# Supplementary material for: Insights into the missing apiosylation step in flavonoid apiosides biosynthesis of Leguminosae plants
Source: Nat Commun. 2023 Oct 20;14:6658. doi: 10.1038/s41467-023-42393-1 (PMC10589286; doi:10.1038/s41467-023-42393-1)
Supplement: Supplementary file 1 — Supplementary Information [file 41467_2023_42393_MOESM1_ESM.pdf]

Supplementary Information

Insights into the missing apiosylation step in flavonoid apiosides biosynthesis of Leguminosae plants

Hao-Tian Wang[1,4], Zi-Long Wang[1,4], Kuan Chen[1], Ming-Ju Yao[1], Meng Zhang[1], Rong-Shen Wang[1], Jia-He Zhang[1], Hans Ågren[2], Fu-Dong Li[3], Junhao Li[2,*], Xue Qiao[1,*] and Min Ye[1,*]

[1] State Key Laboratory of Natural and Biomimetic Drugs, School of Pharmaceutical Sciences, Peking University, 38 Xueyuan Road, Beijing 100191, China

[2] Department of Physics and Astronomy, Uppsala University, SE-751 20, Uppsala, Sweden

[3] National Science Center for Physical Sciences at Microscale Division of Molecular & Cell Biophysics and School of Life Sciences, University of Science and Technology of China, Hefei 230026, China

[4] These authors contributed equally: Hao-Tian Wang and Zi-Long Wang

* Email: junhao.li@physics.uu.se (J. H. Li), qiaoxue@bjmu.edu.cn (X. Qiao), yemin@bjmu.edu.cn (M. Ye)

Table of Contents

1. Supplementary Note 1

HRMS (ESI), ^{1}H and ^{13}C NMR spectral data for apiosylated products 1

2. Supplementary Tables 6

Supplementary Table 1. Accession numbers of plant genes used in this study ... 6

Supplementary Table 2. Compounds used as ApiGT substrates in this study. 8

Supplementary Table 3. HPLC methods used to analyze ApiGT catalyzed products 10

Supplementary Table 4. Data collection and refinement statistics of GuApiGT crystal 11

Supplementary Table 5. Reported crystal structures of plant UGTs 12

Supplementary Table 6. Impact of turning off the atomic charge of outer MM region residues on the activation barriers (ΔE) of the UDP-Api system 15

Supplementary Table 7. Data collection and refinement statistics of Sb3GT1 crystals 16

Supplementary Table 8. A list of 39 plant species with a 45-amino acid PSPG box from Leguminosae 17

3. Supplementary Figures 21

Supplementary Fig. 1 Phylogenetic analysis of GuApiGT (MSTRG.23171.4) with reported UGTs 21

Supplementary Fig. 2 SDS-PAGE analysis of recombinant ApiGTs and GuGT53 purified by Ni affinity chromatography 22

Supplementary Fig. 3 HPLC and LC/MS analyses of GuApiGT catalytic reaction mixture for substrate **1** 23

Supplementary Fig. 4 Effects of reaction time (a), buffer (b), temperature (c), and divalent metal ions (d) on enzyme activity of GuApiGT 24

Supplementary Fig. 5 Determination of kinetic parameters for recombinant GuApiGT (*n*=3) 25

Supplementary Fig. 6 Structures of substrates **38-65** that cannot be catalyzed by GuApiGT 26

Supplementary Figs. 7-42 HPLC/MS analyses of GuApiGT catalytic reaction mixture for substrates **2-37** 27

Supplementary Figs. 43-81 HR-ESI-MS and NMR spectra of apiosylated products 63

Supplementary Fig. 82 The crystal model of GuApiGT predicted by AlphaFold2 83

Supplementary Fig. 83 The PSPG box (W329-H373) in crystal structure of GuApiGT. 84

Supplementary Fig. 84 The initial binding mode of GuApiGT/UDP-Api/**2**. 85

Supplementary Fig. 85 Time evolution of root mean square deviations (RMSD) of protein backbone (CA, Cα atoms), substrate (**2**), and UDP-sugar for MD simulation systems 86

Supplementary Fig. 86 The superimposition of GuApiGT/UDP-Api/**2** snapshots in MD simulations........ 87

Supplementary Fig. 87 HPLC analysis of GuApiGT mutants catalyzed products 88

Supplementary Fig. 88 The complex models of GuApiGT/UDP-Glc/**2** and GuApiGT/UDP-Xyl/**2**........ 89

Supplementary Fig. 89 The MD snapshots of GuApiGT/UDP-Glc/**2**........ 90

Supplementary Fig. 90 The MM/GBSA binding free energy in different systems 91

Supplementary Fig. 91 The function of E272 in the structure of GuApiGT...... 92

Supplementary Figs. 92-93 HPLC and LC/MS analyses of mutants catalyzed product using UDP-Api and UDP-Xyl as sugar donor, respectively........ 93

Supplementary Fig. 94 Superimposition of GuApiGT and GgCGT 96

Supplementary Fig. 95 HPLC analyses of mutants catalyzed products using UDP-Glc as sugar donor 98

Supplementary Fig. 96 Sugar donor preference of GuApiGT mutants. 99

Supplementary Figs. 97-122 HPLC and LC/MS analyses of L369/H373Q and I136T/G370/H373Q catalytic reaction mixtures for substrates **1-3**, **7**, **9**, **12**, **20**, **27**, **31-32**, **34-35** and **37** 100

Supplementary Figs. 123-132 HR-ESI-MS and NMR spectra of glycosylated products **32b** and **3c** 126

Supplementary Fig. 133 The MS spectra of GuApiGT (wild type), L369/H373Q, and I136T/L369/H373Q mutants 131

Supplementary Fig. 134 The peptide segment coverage of GuApiGT in the HDX-MS analysis.. 132

Supplementary Fig. 135 Differential peptides in the model structures of L369/H373Q and I136T/L369/H373Q, by comparing with WT. 133

Supplementary Figs. 136-137 Deuterium uptake plots of peptides 363-372 and 135-156 at different time points 134

Supplementary Fig. 138 Sugar donor selectivity mechanisms of GuApiGT mutants 136

Supplementary Fig. 139 Sequences alignment of Sb3GT1 and GuApiGT. 137

Supplementary Fig. 140 The MS and MS/MS data of **66a** 138

Supplementary Fig. 141 Binding modes of compounds **2**, **2′**, **20** and **38** in GuApiGT. 139

Supplementary Fig. 142 Functional characterization of SsApiGT, PtApiGT, GgApiGT, and GiApiGT 140

Supplementary Fig. 143 Amino acid sequence alignment of GuApiGT, GgApiGT, GiApiGT, SsApiGT and PtApiGT 141

Supplementary Figs. 144-145 LC/MS analyses of **2a** in tabacco 142

Supplementary Fig. 146 Functional characterization of GuGT53 144

Supplementary Figs. 147-155 LC/MS analyses of engineered tabacco. 145

Supplementary Fig. 156 Purification of UDP-Api by HPLC. UV detection wavelength, 262 nm 154

Supplementary Fig. 157 Crystals of GuApiGT, Sb3GT1, and Sb3GT1-375S/Q377H 155

4. Supplementary References 156

Supplementary Note

HRMS (ESI), ^{1}H and ^{13}C NMR spectral data for apiosylated products

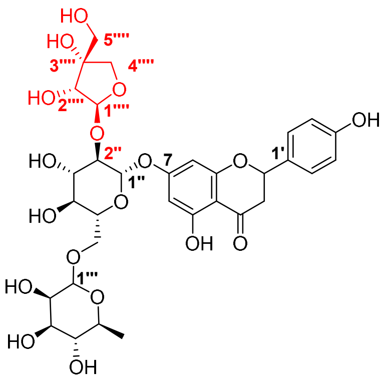

Narirutin 2''-*O*-β-D-apioside (6a)

^{1}H NMR (600 MHz, DMSO-d_6): δ 5.50 (1H, m, H-2), 2.74 (2H, m, H-3), 6.09 (1H, s, H-6), 6.11 (1H, s, H-8), 7.34 (2H, d, J=8.6Hz, H-2′, 6′), 6.79 (2H, d, J=8.6Hz, H-3′, 5′), 5.08 (1H, m, H-1″), 5.30 (1H, d, J=0.8Hz, H-1‴), 4.51 (1H, br s, H-1⁗), 12.02 (1H, s, 5-OH), 9.60 (1H, s, 4′-OH).

^{13}C NMR (150 MHz, DMSO-d_6): δ 78.6 (C-2), 42.0 (C-3), 197.2 (C-4), 163.0 (C-5), 96.3 (C-6), 164.9 (C-7), 95.3 (C-8), 162.7 (C-9), 103.3 (C-10), 128.4 (C-1′), 128.6 (C-2′), 115.2 (C-3′), 157.8 (C-4′), 115.2 (C-5′), 128.6 (C-6′), 97.5 (C-1″), 75.5 (C-2″), 76.6 (C-3″), 69.9 (C-4″), 75.3 (C-5″), 66.0 (C-6″), 108.7 (C-1‴), 76.1 (C-2‴), 79.3 (C-3‴), 74.0 (C-4‴), 64.2 (C-5‴), 17.9 (C-6‴), 100.6 (C-1⁗), 70.3 (C-2⁗), 70.7(C-3⁗), 72.1 (C-4⁗), 68.3 (C-5⁗).

HRMS (ESI) calculated for $C_{32}H_{39}O_{18}$ $[M-H]^-$ m/z 711.2142, found m/z 711.2148.

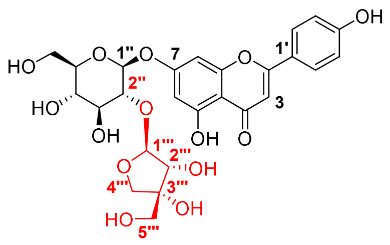

Apiin (15a)

^{1}H NMR (400 MHz, DMSO-*d*$_6$): *δ* 6.87 (1H, s, H-3), 6.43 (1H, d, *J*=2.1Hz, H-6), 6.81 (1H, d, *J*=2.1Hz, H-8), 7.96 (2H, d, *J*=8.8Hz, H-2′, 6′), 6.94 (2H, d, *J*=8.8Hz, H-3′, 5′), 5.16 (1H, d, *J*=5.7Hz, H-1″), 5.35 (1H, d, *J*=1.0Hz, H-1‴), 12.97 (1H, s, 5-OH), 10.41 (1H, s, 4′-OH).

^{13}C NMR (100 MHz, DMSO-*d*$_6$): *δ* 164.3 (C-2), 103.1 (C-3), 182.0 (C-4), 156.9 (C-5), 99.3 (C-6), 162.7 (C-7), 94.8 (C-8), 161.1 (C-9), 105.4 (C-10), 121.0 (C-1′), 128.6 (C-2′), 116.0 (C-3′), 161.1 (C-4′), 116.0 (C-5′), 128.6 (C-6′), 98.1 (C-1″), 75.7 (C-2″), 76.8 (C-3″), 69.8 (C-4″), 77.0 (C-5″), 60.5 (C-6″), 108.7 (C-1‴), 76.1 (C-2‴), 79.3 (C-3‴), 74.0 (C-4‴), 64.2 (C-5‴).

HRMS (ESI) calculated for $C_{26}H_{27}O_{14}$ $[M-H]^-$ *m/z* 563.1406, found *m/z* 563.1408[1].

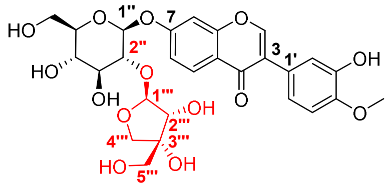

Calycosin 2″-*O*-β-D-apioside (24a)

^{1}H NMR (600 MHz, DMSO-*d*$_6$): *δ* 8.39 (1H, s, H-2), 8.06 (1H, d, *J*=8.7Hz, H-5), 7.10 (1H, dd, *J*=8.7, 2.0Hz, H-6), 7.19 (1H, d, *J*=2.0Hz, H-8), 7.22 (1H, d, *J*=2.3Hz, H-2′), 8.05 (1H, d, *J*=8.9Hz, H-5′), 7.13 (1H, dd, *J*=8.9, 2.3Hz, H-6′), 5.19 (1H, d, *J*=7.7Hz, H-1″), 5.36 (1H, d, *J*=1.0Hz, H-1‴), 9.05 (1H, s, 3'-OH), 3.79 (3H, s, 4′-OCH$_3$).

^{13}C NMR (150 MHz, DMSO-*d*$_6$): *δ* 153.6 (C-2), 123.6 (C-3), 174.6 (C-4), 127.0 (C-5), 115.5 (C-6), 161.3 (C-7), 103.4 (C-8), 157.0 (C-9), 118.6 (C-10), 124.5 (C-1′), 116.4 (C-2′), 146.1 (C-3′), 147.6 (C-4′), 112.0 (C-5′), 119.7 (C-6′), 98.4 (C-1″), 75.7 (C-2″), 76.9 (C-3″), 69.9 (C-4″), 77.1 (C-5″), 60.6 (C-6″), 108.7 (C-1‴), 76.1 (C-2‴), 79.3 (C-3‴), 74.0 (C-4‴), 64.2 (C-5‴), 55.7 (4′- OCH$_3$).

HRMS (ESI) calculated for $C_{27}H_{29}O_{14}$ $[M-H]^-$ *m/z* 577.1563, found *m/z* 577.1562.

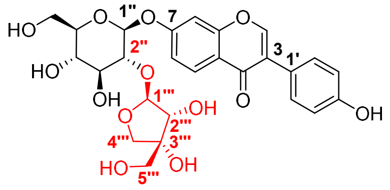

Daidzin 2″-*O*-β-D-apioside (27a)

^{1}H NMR (600 MHz, DMSO-d_6): δ 8.38 (1H, s, H-2), 8.04 (1H, d, J=8.8Hz, H-5), 7.12 (1H, dd, J=8.8, 2.2Hz, H-6), 7.21 (1H, d, J=2.2Hz, H-8), 7.40 (2H, d, J=8.6Hz, H-2′, 6′), 6.81 (2H, d, J=8.6Hz, H-3′, 5′), 5.19 (1H, d, J=7.6Hz, H-1″), 5.35 (1H, d, J=0.8Hz, H-1‴).

^{13}C NMR (150 MHz, DMSO-d_6): δ 153.4 (C-2), 123.8 (C-3), 174.9 (C-4), 127.1 (C-5), 115.6 (C-6), 161.3 (C-7), 103.4 (C-8), 157.3 (C-9), 118.6 (C-10), 122.4 (C-1′), 130.2 (C-2′), 115.1 (C-3′), 157.3 (C-4′), 115.1 (C-5′), 130.2 (C-6′), 98.4 (C-1″), 75.9 (C-2″), 76.9 (C-3″), 69.9 (C-4″), 77.1 (C-5″), 60.6 (C-6″), 108.8 (C-1‴), 76.2 (C-2‴), 79.4 (C-3‴), 74.0 (C-4‴), 64.2 (C-5‴).

HRMS (ESI) calculated for $C_{26}H_{27}O_{13}$ $[M-H]^-$ *m/z* 547.1457, found *m/z* 547.1459.

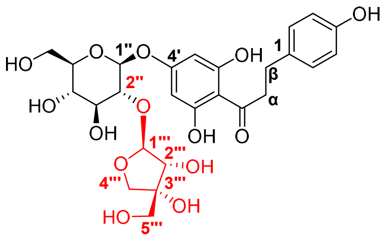

Trilobatin 2″-*O*-β-D-apioside (32a)

^{1}H NMR (600 MHz, DMSO-d_6): δ 7.01 (2H, d, J=8.3Hz, H-2, 6), 6.66 (2H, d, J=8.3Hz, H-3, 5), 6.02 (2H, s, H-3′, 5′), 3.25 (2H, m, H-α), 2.78 (2H, m, H-β), 4.97 (1H, d, J=7.1Hz, H-1″), 5.31 (1H, d, J=0.9Hz, H-1‴), 9.16 (1H, s, 4-OH), 12.29 (2H, s, 2′-OH, 6′-OH).

^{13}C NMR (150 MHz, DMSO-d_6): δ 205.1 (C=O), 131.5 (C-1), 129.2 (C-2), 115.1 (C-3), 155.4 (C-4), 115.1 (C-5), 129.2 (C-6), 105.3 (C-1′), 163.8 (C-2′), 94.9 (C-3′), 163.1 (C-4′), 94.9 (C-5′), 163.8 (C-6′), 97.7 (C-1″), 75.8 (C-2″), 76.7 (C-3″), 69.6 (C-4″), 76.9 (C-5″), 60.4 (C-6″), 108.9 (C-1‴), 76.0 (C-2‴), 79.3 (C-3‴), 74.0 (C-4‴), 64.2 (C-5‴), 45.8 (C-α), 29.3 (C-β).

HRMS (ESI) calculated for $C_{26}H_{31}O_{14}$ $[M-H]^-$ *m/z* 567.1719, found *m/z* 567.1722.

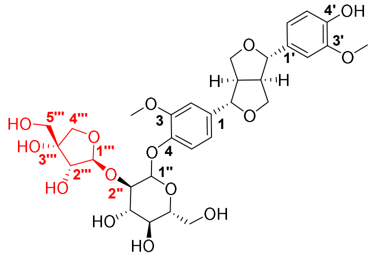

Pinoresinol 4-*O*-β-D-apiosyl(1→2)-β-D-glucoside (35a)

^{1}H NMR (600 MHz, DMSO-d_6): δ 6.94 (1H, d, J=1.8Hz, H-2), 7.01 (1H, d, J=8.5Hz, H-5), 6.84 (1H, dd, J=8.5, 1.8Hz, H-6), 4.67 (1H, d, J=4.6Hz, H-7), 4.61 (1H, d, J=4.6Hz, H-7′), 3.05 (2H, m, H-8, 8′), 4.13 (2H, m, H-9a, 9′a), 3.75 (2H, m, H-9b, 9′b), 6.89 (1H, d, J=1.8Hz, H-2′), 6.72 (1H, d, J=8.0Hz, H-5′), 6.75 (1H, dd, J=8.0,1.8Hz, H-6′), 4.92 (1H, d, J=7.7Hz, H-1″), 5.41 (1H, d, J=0.7Hz, H-1‴), 3.75 (3H, s, 3-OCH_3), 3.76 (3H, s, 3′-OCH_3), 8.90 (1H, s, 4′-OH).

^{13}C NMR (150 MHz, DMSO-d_6): δ 135.2 (C-1), 110.3 (C-2), 145.6 (C-3), 148.8 (C-4), 114.9 (C-5), 118.0 (C-6), 85.0 (C-7), 53.6 (C-8), 71.0 (C-9), 85.2 (C-7′), 53.7 (C-8′), 71.0 (C-9′), 132.2 (C-1′), 110.4 (C-2′), 147.5 (C-3′), 145.9 (C-4′), 115.1 (C-5′), 118.7 (C-6′), 55.6 (3-OCH_3), 55.6 (3′-OCH_3), 98.5 (C-1″), 75.0 (C-2″), 76.9 (C-3″), 70.0 (C-4″), 77.2 (C-5″), 60.6 (C-6″), 108.3 (C-1‴), 76.1 (C-2‴), 79.4 (C-3‴), 73.9 (C-4‴), 64.5 (C-5‴).

HRMS (ESI) calculated for $C_{31}H_{39}O_{15}$ $[M-H]^-$ *m/z* 651.2294, found *m/z* 651.2294.

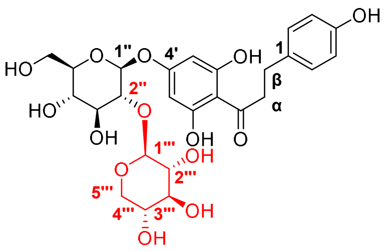

Trilobatin 2″-*O*-β-D-xyloside (32b)

^{1}H NMR (400 MHz, DMSO-d_6): δ 7.02 (2H, d, J=8.5Hz, H-2, 6), 6.66 (2H, d, J=8.5Hz, H-3, 5), 6.03 (2H, overlapped, H-3′, 5′), 3.26 (2H, t, J=7.8Hz, H-α), 2.78 (2H, t, 7.8Hz, H-β), 5.01 (1H, d, J=7.5Hz, H-1″), 4.44 (1H, d, J=7.4Hz, H-1‴), 9.22 (1H, s, 4-OH), 12.38 (2H, s, 2′, 6′-OH).

^{13}C NMR (100 MHz, DMSO-d_6): δ 205.1 (C=O), 131.5 (C-1), 129.2 (C-2), 115.1 (C-3), 155.5 (C-4), 115.1 (C-5), 129.2 (C-6), 105.3 (C-1′), 163.8 (C-2′), 95.2 (C-3′), 163.3 (C-4′), 95.2 (C-5′), 163.8 (C-6′), 98.0 (C-1″), 82.2 (C-2″), 76.0 (C-3″), 69.0 (C-4″), 76.9 (C-5″), 60.3 (C-6″), 105.0 (C-1‴), 74.2 (C-2‴), 76.0 (C-3‴), 69.6 (C-4‴), 65.9 (C-5‴), 45.8 (C-α), 29.4 (C-β).

HRMS (ESI) calculated for $C_{26}H_{31}O_{14}$ $[M-H]^-$ *m/z* 567.1719, found *m/z* 567.1764.

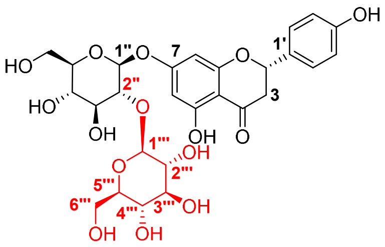

Naringenin 7-*O*-β-D-glucosyl(1→2)-β-D-glucoside (3c)

^{1}H NMR (400 MHz, DMSO-d_6): δ 5.50 (1H, m, H-2), 2.74 (2H, m, H-3), 6.16 (1H, d, *J*=1.8Hz, H-6), 6.18 (1H, d, *J*=1.8Hz, H-8), 7.32 (2H, d, *J*=8.5Hz, H-2′, 6′), 6.80 (2H, d, *J*=8.5Hz, H-3′, 5′), 9.67 (1H, s, 4′-OH), 12.05 (1H, s, 5-OH).

^{13}C NMR (100 MHz, DMSO-d_6): δ 78.7 (C-2), 42.1 (C-3), 197.3 (C-4), 163.0 (C-5), 95.6 (C-6), 165.1 (C-7), 95.5 (C-8), 162.9 (C-9), 103.3 (C-10), 128.6 (C-1′), 128.4 (C-2′), 115.2 (C-3′), 157.8 (C-4′), 115.2 (C-5′), 128.4 (C-6′), 97.9 (C-1″), 82.5 (C-2″), 78.7(C-3″), 69.1 (C-4″), 75.7 (C-5″), 60.5 (C-6″), 104.5 (C-1‴), 74.7 (C-2‴), 76.8 (C-3‴), 69.5 (C-4‴), 76.2 (C-5‴), 60.4 (C-6‴).

HRMS (ESI) calculated for $C_{27}H_{31}O_{15}$ $[M\text{-}H]^-$ *m*/*z* 595.1668, found *m*/*z* 595.1665.

Supplementary Tables

Supplementary Table 1. Accession numbers of plant genes used in this study.

Name	Organism	Genbank accession number
**GuApiGT**	***Glycyrrhiza uralensis***	OQ201607
**GgApiGT**	***Glycyrrhiza glabra***	OQ230797
**GiApiGT**	***Glycyrrhiza inflata***	OQ230796
**SsApiGT**	***Spatholobus suberectus***	OQ230794
**PtApiGT**	***Pueraria thomsonii***	OQ230795
**GuGT53**	***Glycyrrhiza uralensis***	OQ266890
AtUGT79B6	*Arabidopsis thaliana*	Q9FN26
UGT79B1	*Arabidopsis thaliana*	Q9LVW3
ZjOGT38	*Ziziphus jujuba*	UNP42106
TcOGT4	*Trollius chinensis*	MK947398
In3GGT	*Ipomoea nil*	Q53UH4
UGT79B30	*Glycine max*	BAR88078
UGT79B31	*Petunia hybrida*	BBE29003
Cs1-6RhaT	*Citrus sinensis*	ABA18631
LeABRT2	*Lobelia erinus*	BAU68118
Ph3RT	*Petunia hybrida*	CAA81057
MaF3GRT	*Morus alba*	ALD83609
GmUGT79A7	*Glycine max*	BAV56172
GmUGT79A6	*Glycine max*	BAN91401
UGT91L1	*Zea mays*	NP_001347041
Cm1-2RhaT1	*Citrus maxima*	AAL06646
VpUGT94F1	*Veronica persica*	BAI44133
CaUGT3	*Catharanthus roseus*	BAH80312
BpUGAT	*Bellis perennis*	BAD77944
PdUGT85A19	*Prunus dulcis*	ABV68925
CsUGT707B1	*Crocus sativus*	CCG85331
Ih3GT	*Iris hollandica*	BAD83701
Sb3GT1	*Scutellaria baicalensis*	QBL54224
VvGT1	*Vitis vinifera*	AAB81683
UGT78D2	*Arabidopsis thaliana*	NP_197207
UGT78K1	*Glycine max*	NP_001304377
UGT78G1	*Medicago truncatula*	XP_003610163
Pf5GT	*Perilla frutescens*	BAA36421
Vh5GT	*Glandularia hybrida*	BAA36423
Gt5GT7	*Gentiana triflora*	BAG32255
UGT75C1	*Arabidopsis thaliana*	Q0WW21
Ih5GT	*Iris hollandica*	BAD06874
UGT89C1	*Arabidopsis thaliana*	Q9LNE6
UGT73C6	*Arabidopsis thaliana*	NP_181217

UGT73A10	*Lycium barbarum*	BAG80536
SbUF7GT	*Scutellaria baicalensis*	BAA83484
TcCGT1	*Trollius chinensis*	MK644229
GgCGT	*Glycyrrhiza glabra*	QGL05036
SbCGTa	*Scutellaria baicalensis*	QLF98861
SbCGTb	*Scutellaria baicalensis*	QLF98862
AtPAL	*Arabidopsis thaliana*	NM_129260.3
AtC4H	*Arabidopsis thaliana*	NM_128601.3
At4CL	*Arabidopsis thaliana*	DQ062385.1
AtCHS	*Arabidopsis thaliana*	DQ062415
GuCHR	*Glycyrrhiza uralensis*	MK341786
GuCHI	*Glycyrrhiza uralensis*	MK348532.1
AtCHI	*Arabidopsis thaliana*	NM_180439
PcFNSI	*Petroselinum crispum*	Q7XZQ8.1
GuGT14	*Glycyrrhiza uralensis*	MK534521
pgm	*Nocardia farcinica*	BAD59304.1
GalU	*Escherichia coli*	WP_262018987.1
CalS8	*Micromonospora echinospora*	AAM70332.1
UAXS	*Arabidopsis thaliana*	NM_128345

Supplementary Table 2. Compounds used as ApiGT substrates in this study.

No.	Name	CAS number	Molecular formula	Molecular weight
**1**	Liquiritin	551-15-5	$C_{21}H_{22}O_9$	418
**2**	Isoliquiritin	5041-81-6	$C_{21}H_{22}O_9$	418
**3**	Naringenin 7-*O*-glucoside	529-55-5	$C_{21}H_{22}O_{10}$	434
**4**	Neoliquiritin	5088-75-5	$C_{21}H_{22}O_9$	418
**5**	Eriodictyol 7-*O*-glucoside	38965-51-4	$C_{21}H_{22}O_{11}$	450
**6**	Narirutin	14259-46-2	$C_{27}H_{32}O_{14}$	580
**7**	Neoisoliquiritin	59122-93-9	$C_{21}H_{22}O_9$	418
**8**	Isoliquiritigenin 4,4'-*O*-diglucoside	69262-36-8	$C_{27}H_{32}O_{14}$	580
**9**	Sophoraflavone B	22052-75-1	$C_{21}H_{20}O_9$	416
**10**	Isosaponarin	19416-87-6	$C_{27}H_{30}O_{15}$	594
**11**	Flavocommelin	16049-42-6	$C_{28}H_{32}O_{15}$	608
**12**	7,4'-Dihydroxyflavone 7-*O*-glucoside	20633-86-7	$C_{21}H_{20}O_9$	416
**13**	Cynaroside	5373-11-5	$C_{21}H_{20}O_{11}$	448
**14**	Diosmetin 7-*O*-glucoside	20126-59-4	$C_{22}H_{22}O_{11}$	462
**15**	Apigenin 7-*O*-glucoside	578-74-5	$C_{21}H_{20}O_{10}$	432
**16**	Oroxylin A 7-*O*-glucoside	36948-77-3	$C_{22}H_{22}O_{10}$	446
**17**	Wogonin 7-*O*-glucoside	866621-11-6	$C_{22}H_{22}O_{10}$	446
**18**	7,2'-Dihydroxyflavone 7,2'-*O*-diglucoside	From our lab.	$C_{27}H_{30}O_{14}$	578
**19**	Luteolin 7,4'-*O*-diglucoside	70404-47-6	$C_{27}H_{30}O_{16}$	610
**20**	Wogonin 5-*O*-glucoside	80366-14-9	$C_{22}H_{22}O_{10}$	446
**21**	Isovitexin	29702-25-8	$C_{21}H_{20}O_{10}$	432
**22**	Isoorientin	4261-42-1	$C_{21}H_{20}O_{11}$	448
**23**	Genistin	529-59-9	$C_{21}H_{20}O_{10}$	432
**24**	Calycosin 7-*O*-glucoside	20633-67-4	$C_{22}H_{22}O_{10}$	446
**25**	Tectoridin	611-40-5	$C_{22}H_{22}O_{11}$	462
**26**	Ononin	486-62-4	$C_{22}H_{22}O_9$	430
**27**	Daidzin	552-66-9	$C_{21}H_{20}O_9$	416
**28**	Spireoside	20229-56-5	$C_{21}H_{20}O_{12}$	464
**29**	Quercetin 7,4'-*O*-diglucoside	42900-82-3	$C_{27}H_{30}O_{17}$	626
**30**	Quercetin 7-*O*-glucoside	491-50-9	$C_{21}H_{20}O_{12}$	464
**31**	Kaempferol 7-*O*-glucoside	16290-07-6	$C_{21}H_{20}O_{11}$	448
**32**	Trilobatin	4192-90-9	$C_{21}H_{24}O_{10}$	436
**33**	Mangiferin	4773-96-0	$C_{19}H_{18}O_{11}$	422
**34**	Isomangiferin	24699-16-9	$C_{19}H_{18}O_{11}$	422
**35**	Pinoresinol 4-*O*-glucoside	69251-96-3	$C_{26}H_{32}O_{11}$	520
**36**	Forsythin	487-41-2	$C_{27}H_{34}O_{11}$	534
**37**	Skimmin	93-39-0	$C_{15}H_{16}O_8$	324
**38**	Astragalin	480-10-4	$C_{21}H_{20}O_{11}$	448
**39**	Kaempferol 3-*O*-galactoside	23627-87-4	$C_{21}H_{20}O_{11}$	448

**40**	Kaempferol 3-*O*-*N*-acetylglucosamine	2358715-08-7	$C_{23}H_{23}NO_{11}$	489
**41**	Isoquercetin	482-35-9	$C_{21}H_{20}O_{12}$	464
**42**	Hyperoside	482-36-0	$C_{21}H_{20}O_{12}$	464
**43**	Quercetin 3-*O*-L-arabinoside	22255-13-6	$C_{20}H_{18}O_{11}$	434
**44**	Reinutrin	549-32-6	$C_{20}H_{18}O_{11}$	434
**45**	Quercetin 3-*O*-rhamnoside	522-12-3	$C_{21}H_{20}O_{11}$	448
**46**	Quercetin 3-*O*-rutinoside	949926-49-2	$C_{27}H_{30}O_{16}$	610
**47**	Puerarin	3681-99-0	$C_{21}H_{20}O_{9}$	416
**48**	Vicenin-2	23666-13-9	$C_{27}H_{30}O_{15}$	594
**49**	Schaftoside	51938-32-0	$C_{26}H_{28}O_{14}$	564
**50**	Isoschaftoside	52012-29-0	$C_{26}H_{28}O_{14}$	564
**51**	Isoviolanthin	40788-84-9	$C_{27}H_{30}O_{14}$	578
**52**	Chrysin	480-40-0	$C_{15}H_{10}O_{4}$	254
**53**	Wogonin	632-85-9	$C_{16}H_{12}O_{5}$	284
**54**	Oroxylin A	480-11-5	$C_{16}H_{12}O_{5}$	284
**55**	Luteolin	491-70-3	$C_{15}H_{10}O_{6}$	286
**56**	Calycosin	20575-57-9	$C_{16}H_{12}O_{5}$	284
**57**	Isoliquiritigenin	961-29-5	$C_{15}H_{12}O_{4}$	256
**58**	Trilobatin	60-81-1	$C_{21}H_{24}O_{10}$	436
**59**	Wogonoside	51059-44-0	$C_{22}H_{20}O_{11}$	460
**60**	Loganin	18524-94-2	$C_{17}H_{26}O_{10}$	390
**61**	Secoxyloganin	58822-47-2	$C_{17}H_{24}O_{11}$	404
**62**	Chlorogenic acid	327-97-9	$C_{16}H_{18}O_{9}$	354
**63**	Glucosyringic acid	33228-65-8	$C_{15}H_{20}O_{10}$	360
**64**	Forsythoside A	79916-77-1	$C_{29}H_{36}O_{15}$	624
**65**	Glycyrrhetic acid 3-*O*-glucuronide	34096-83-8	$C_{36}H_{54}O_{10}$	646
**66**	Kaempferol	520-18-3	$C_{15}H_{10}O_{6}$	286

Supplementary Table 3. HPLC methods used to analyze ApiGT catalyzed products.

Method	Solvent A	Solvent B	Elution gradient	Substrates
A	Water containing 0.1% formic acid	ACN	25-34% B, 8.5min;<br>34%-25% B, 0.5min;<br>25% B, 5min	**1, 2**
B	Water containing 0.1% formic acid	ACN	5-30% B, 20min;<br>30-100% B, 5min;<br>100-5% B, 1min;<br>5% B, 6min	**1-7, 9, 12-32, 35-66, 1′-2′**
C	Water containing 0.1% formic acid	ACN	5-15% B,15min;<br>15-100% B, 10min;<br>100-5% B, 1min;<br>5% B, 6min	**8, 10-11**
D	Water containing 0.1% formic acid	ACN	13-28% B, 20min;<br>28-100% B, 5min;<br>100-13% B, 1min;<br>13% B, 6min	**27**
E	Water containing 0.1% formic acid	MeOH	20-45% B, 16min;<br>45-20% B, 1min;<br>20% B, 6min	**33-34**
F	Water containing 0.1% formic acid	ACN	10-25% B, 20min;<br>25-100% B, 5min;<br>100-10% B, 1min;<br>10% B, 6min	**37**

Supplementary Table 4. Data collection and refinement statistics of GuApiGT crystal.

	**GuApiGT**
Wavelength(Å)	0.9785
Space group	*C*2
Cell parameters	
a, b, c (Å)	161.16, 128.69, 47.56
α, β, γ (°)	90, 91.07, 90
Resolution(Å)	19.66-2.20(2.27-2.20)[a]
R_{merge} (%)	16.5(92.5)
$CC_{1/2}$(%)	99.0(82.5)
$I/\sigma I$	7.4(2.0)
Completeness (%)	99.3(98.3)
Average redundancy	6.8(6.4)
**Refinement**	
No. reflections (overall)	48728
No. reflections (test set)	2332
R_{work}/R_{free}(%)	19.14/23.72
Number of atoms	
Protein	7028
H_2O	254
SO_4	45
UDP	
UDPGlc	
B factors (Å^2)	
Protein	38.96
H_2O	37.73
SO_4	55.55
UDP	
UDPGlc	
r.m.s. deviations	
Bond lengths (Å)	0.008
Bond angles (°)	0.959
Rampage plot % residues	
Favored	97.31
Allowed	2.69
Outliers	0

[a] Values in parentheses are for highest-resolution shell.

Supplementary Table 5. Reported crystal structures of plant UGTs.

Name	Source	Structure	Resolution	PDB ID
UGT71G1[2]	*Medicago truncatula*	UGT71G1/UDP	2.0 Å	2ACV
		UGT71G1/UDP-Glc	2.6 Å	2ACW
VvGT1[3]	*Vitis vinifera*	VvGT1/ UDP	1.9 Å	2C1X
		VvGT1/UDP-2FGlc/kaempferol	1.9 Å	2C1Z
		VvGT1/UDP/quercetin	2.1 Å	2C9Z
UGT85H2[4]	*Medicago truncatula*	UGT85H2 apo	2.1 Å	2PQ6
UGT72B1[5]	*Arabidopsis thaliana*	UGT72B1/UDP	1.45 Å	2VCH
		UGT72B1/UDP/Tris	1.75 Å	2VG8
		UGT72B1/UDP-2FGlc/TCP	1.9 Å	2VCE
UGT78G1[6]	*Medicago truncatula*	UGT78G1/UDP	2.1 Å	3HBJ
		UGT78G1/UDP/myricetin	2.1 Å	3HBF
UGT78K6[7]	*Clitoria ternatea*	UGT78K6 apo	1.85 Å	3WC4
		UGT78K6/UDP	1.85 Å	4WHM
		UGT78K6/Delphinidin	2.55 Å	4REM
		UGT78K6/Petunidin	2.7 Å	4REN
		UGT78K6/Kaempferol	1.75 Å	4REL
Os79[8,9]	*Oryza sativa*	Os79/UDP (open conformation)	1.78 Å	5TME
		Os79/UDP (closed conformation)	2.34 Å	5TMB
		Os79/UDP-2FGlc/trichothecene	2.19 Å	5TMD
		Os79 (Q202A)/UDP	1.47 Å	6BK0
		Os79 (H122A/L123A)/UDP	1.29 Å	6BK1
		Os79 (T291A)/UDP	1.58 Å	6BK2
		Os79/UDP/D3G	2.17 Å	6BK3
UGT74F2[10]	*Arabidopsis thaliana*	UGT74F2/UDP/SA	2.56 Å	5U6M
		UGT74F2/UDP/2-bromobenzoic acid	2 Å	5U6S
		UGT74F2$_{T15S}$/UDP/SA	2 Å	5U6N
		UGT74F2$_{T15S}$/UDP/2-bromobenzoic acid	1.8 Å	5V2J

		$UGT74F2_{T15A}$/UDP/2-bromobenzoic acid	2 Å	5V2K
PtUGT1[11]	*Polygonum tinctorium*	PtUGT1	2.14 Å	5NLM
UGT89C1[12]	*Arabidopsis thaliana*	UGT89C1	2.7 Å	6IJ7
		UGT89C1/UDP	3 Å	6IJ9
		UGT89C1/UDP-Rha	3.21 Å	6IJA
		UGT89C1/Quercetin	3.2 Å	6IJD
UGT76G1[13-15]	*Stevia rebaudiana*	UGT76G1/UDP (SeMet)	1.8 Å	6O86
		UGT76G1/UDP	1.75 Å	6O8Q
		UGT76G1/UDP/rebaudioside A	1.99 Å	6O88
		UGT76G1/UDP	1.69 Å	6INF
		UGT76G1/UDP/Reb A	2.1 Å	6INH
		UGT76G1/UDP/Rubu	1.7 Å	6INI
		$UGT76G1_{H25A}$/UDP	1.7 Å	6ING
TcCGT1[16]	*Trollius chinensis*	TcCGT1/UDP	1.85 Å	6JTD
UGT74AC1[17]	*Siraitia grosvenorii*	UGT74AC1	2.02 Å	6L90
		UGT74AC1/UDPG	2.1 Å	6L8Z
GgCGT[18]	*Glycyrrhiza glabra*	GgCGT/UDP-Glc	2.6 Å	6L5P
		GgCGT/UDP-Gal	2.9 Å	6L5Q
		GgCGT/UDP/phloretin	1.9/2.9 Å	6L5S/6L5R
		GgCGT/UDP/nothofagin	1.8 Å	6L7H
UGT708C1[19]	*Fagopyrum esculentum*	UGT708C1 apo	2.1 Å	6LLG
		UGT708C1/UDP	2.25 Å	6LLW
		UGT708C1/UDPG	2 Å	6LLZ
MiCGT[20]	*Mangifera indica*	MiCGT/UDPG	2.85 Å	7VA8
		$MiCGT_{VFAH}$/UDP	3.1 Å	7VAA
PaGT2[21,22]	*Phytolacca americana*	PaGT2 apo	2.3 Å	6JEL
		PaGT2/UDP-2Flc/resveratrol	2.6 Å	6JEM
		PaGT2/UDP-2FGlc/pterostilbene	2.65 Å	6JEN

PaGT3[21,22]	*Phytolacca americana*	PaGT3/18-crown-6	2.4 Å	6lzy
		PaGT3/15-crown-5	3.1 Å	6lzx
UGT74AC2[23]	*Siraitia grosvenorii*	UGT74AC2/UDP	1.85 Å	7BV3
SbCGTa[24]	*Scutellaria baicalensis*	SbCGTa/UDP	3.0 Å	6LG0
SbCGTb[24]	*Scutellaria baicalensis*	SbCGTb/UDP-Glc	2.85 Å	6LFZ
LpCGTa[24]	*Landoltia punctata*	LpCGTa/UDP	3.0 Å	6LG1
LpCGTb[24]	*Landoltia punctata*	LpCGTb	2.36 Å	6LFN
ZmCGTa[24]	*Zea mays*	ZmCGTa/UDP	2.05 Å	6LF6
OsUGT91C[25]	*Oryza sativa*	OsUGT91C1 apo	1.77 Å	7ERY
		OsUGT91C1/UDP/Reb E	1.39 Å	7ES0
		OsUGT91C1/UDP/ST	1.66 Å	7ES1
		OsUGT91C1/UDP/STB	1.92 Å	7ERX
		OsUGT91C1H27A/UDP/Reb D	2.34 Å	7ES2
UGT74AN2[26]	*Calotropis gigantea*	UGTT4AN2 apo	1.95 Å	7W09
		UGT74AN2/UDP	2.04 Å	7W0K
		UGT74AN2/UDP-Glc	2.15 Å	7W1H
		UGT74AN2/UDP/ resibufogenin	2.10 Å	7W0Z
		UGT74AN2/UDP/bufalin	2.15 Å	7W10
		UGT74AN2/UDP/ digitoxigenin	2.3 Å	7W1B
		UGT74AN2/UDP/ digitoxigenin 3-*O*-glucoside	2.45 Å	7W11

Supplementary Table 6. Impact of turning off the atomic charge of outer MM region residues on activation barrier (ΔE) of the UDP-Api system.

Modifications[a]	ΔE (kcal/mol)[b]	ΔΔE (kcal/mol)[c]
Original	14.49	
P12	13.07	1.42
W13	12.73	1.75
A15	14.96	0.48
G17	18.45	3.97
L19	11.71	2.77
P21	13.77	0.72
Y22	7.44	7.04
T78	14.14	0.34
F86	14.38	0.11
F114	17.35	2.87
F116	10.23	4.25
Q117	14.56	0.07
Y134	15.57	1.08
L135	16.08	1.59
V137	12.23	2.26
N138	14.85	0.37
T141	12.49	2.00
F189	14.27	0.21
F195	15.02	0.53
**E272**	-7.19	**21.67**
H347	15.90	1.41
C348	15.12	0.63
G349	14.07	0.41
A350	12.71	1.78
A351	16.28	1.79
S352	15.34	0.85
P367	14.06	0.43
**R368**	26.55	**12.06**
G370	14.19	0.29
I374	15.32	0.84
N376	13.72	0.76

[a.] The atomic charge of a specific residue was set to zero for the single point energy calculations.

[b.] The activation barrier for each system was derived from the difference of ONIOM energy extrapolation + zero-point energy correction between RC and TS.

[c.] ΔΔE is the absolute value of the difference of ΔE between a charge-modified system and the original UDP-Api system.

[d.] The important residues that has an impact of more 10 kcal/mol on the original activation energy are highlighted in bold font.

Supplementary Table 7. Data collection and refinement statistics of Sb3GT1 crystals.

	**Sb3GT1/UDP**	**Sb3GT1 375S/Q377H complex with UDP-Glc**
Wavelength(Å)	0.97918	0.97918
Space group	*P 21 21 2*	*P 21 21 21*
Cell parameters		
a, b, c (Å)	101.65, 61.28, 68.64	47.47, 74.52, 128.98
α, β, γ (°)	90, 90, 90	90, 90, 90
Resolution(Å)	45.71-1.9(1.94-1.9)[a]	47.47-1.43(1.45-1.43)[a]
R_{merge} (%)	10.6 (133)	4.3(103)
$CC_{1/2}$(%)	99.9(75.3)	100(78)
$I/\sigma I$	18.6(2.2)	24.8(2.1)
Completeness (%)	99.0 (96.8)	100(100)
Average redundancy	13 (10.8)	12.8(10.5)
**Refinement**		
No. reflections (overall)	34678	85370
No. reflections (test set)	1706	4352
R_{work}/R_{free}(%)	19.99/23.89	14.59/18.43
Number of atoms		
Protein	3425	3514
H_2O	96	222
SO_4		
UDP	25	
UDPGlc		36
B factors (Å^2)		
Protein	35.81	29.92
H_2O	35.02	36.37
SO_4		
UDP	36.64	
UDPGlc		34.33
r.m.s. deviations		
Bond lengths (Å)	0.0063	0.0155
Bond angles (°)	1.4130	1.9494
Rampage plot % residues		
Favored	97.21	97.49
Allowed	2.79	2.28
Outliers	0	0.23

[a] Values in parentheses are for highest-resolution shell.

Supplementary Table 8. A list of 39 plant species with a 45-amino acid PSPG box from Leguminosae.

No.	Species	Name	Genbank accession number	Number of genes
1	*Glycyrrhiza uralensis*	UGT79B74 (GuApiGT)	OQ201607	1
2	*Glycyrrhiza glabra*	UGT79B75 (GgApiGT)	OQ230797	3
		UGT79B79	OR372699	
		UGT79B80	OR372700	
3	*Glycyrrhiza inflata*	UGT79B76 (GiApiGT)	OQ230796	1
4	*Pueraria thomsonii*	UGT79B77 (PtApiGT)	OQ230795	1
5	*Abrus precatorius*	UGT79B81	OR372676	9
		UGT79B82	OR372677	
		UGT79B83	OR372678	
		UGT79B84	OR372679	
		UGT79B85	OR372680	
		UGT79B86	OR372681	
		UGT79B87	OR372682	
		UGT79B88	OR372683	
		UGT79B89	OR372684	
6	*Cicer arietinum*	UGT79B23	OR372685	6
		UGT79B24	OR372687	
		UGT79B25	OR372686	
		UGT79B26	OR372688	
		UGT79B90	OR372689	
		UGT79B91	OR372735	
7	*Lupinus angustifolius*	UGT79B92	OR372736	1
8	*Cajanus cajan*	UGT79B93	OR372690	5
		UGT79B94	OR372691	
		UGT79B95	OR372692	
		UGT79B96	OR372693	
		UGT79B97	OR372694	
9	*Trifolium pratense*	UGT79B98	OR372751	5
		UGT79B99	OR372752	
		UGT79B104	OR372753	

		UGT79B105	OR372754	
		UGT79B106	OR372755	
		UGT79B103	OR372696	
10	*Codariocalyx motorius*	UGT79B107	OR372697	3
		UGT79B108	OR372698	
11	*Spatholobus suberectus*	UGT79B78 (SsApiGT)	OQ230794	2
		UGT79B109	OR372756	
		UGT79B110	OR372713	
		UGT79B111	OR372714	
12	*Medicago truncatula*	UGT79B112	OR372715	6
		UGT79B113	OR372716	
		UGT79B114	OR372717	
		UGT79B115	OR372718	
		UGT79B116	OR372757	
13	*Trifolium subterraneum*	UGT79B117	OR372758	4
		UGT79B118	OR372759	
		UGT79B119	OR372760	
		UGT79B120	OR372706	
		UGT79B121	OR372707	
		UGT79B122	OR372708	
14	*Glycine soja*	UGT79B123	OR372709	7
		UGT79B124	OR372710	
		UGT79B125	OR372761	
		UGT79B126	OR372711	
		UGT79B127	OR372701	
		UGT79B128	OR372702	
15	*Glycine max*	UGT79B129	OR372703	5
		UGT79B130	OR372704	
		UGT79B131	OR372763	
16	*Cladrastis lutea*	UGT79B132	OR372695	1
		UGT79B133	OR372744	
		UGT79B134	OR372745	
		UGT79B135	OR372746	
17	*Vigna unguiculata*	UGT79B136	OR372750	7
		UGT79B137	OR372749	
		UGT79B138	OR372747	
		UGT79B139	OR372748	

18	*Arachis duranensis*	UGT79B140	OR372660	3
		UGT79B141	OR372661	
		UGT79B142	OR372662	
19	*Astragalus propinquus*	UGT79B143	OR372733	2
		UGT79B144	OR372734	
20	*Vigna angularis*	UGT79B145	OR372738	6
		UGT79B146	OR372743	
		UGT79B147	OR372739	
		UGT79B148	OR372740	
		UGT79B149	OR372741	
		UGT79B150	OR372742	
21	*Vigna radiata* var. *radiata*	UGT79B151	OR372728	5
		UGT79B152	OR372729	
		UGT79B153	OR372730	
		UGT79B154	OR372731	
		UGT79B155	OR372732	
22	*Gleditsia sinensis*	UGT79B156	OR372762	1
23	*Arachis hypogaea*	UGT79B157	OR372668	10
		UGT79B158	OR372669	
		UGT79B159	OR372664	
		UGT79B160	OR372665	
		UGT79B161	OR372764	
		UGT79B162	OR372666	
		UGT79B163	OR372670	
		UGT79B164	OR372667	
		UGT79B165	OR372765	
		UGT79B166	OR372663	
24	*Arachis ipaensis*	UGT79B167	OR372671	4
		UGT79B168	OR372672	
		UGT79B169	OR372673	
		UGT79B170	OR372674	
25	*Astragalus membranaceus*	UGT79B171	OR372675	2
		UGT79B172	OR372775	
26	*Gleditsia triacanthos*	UGT79B173	OR372766	1
27	*Mucuna pruriens*	UGT79B174	OR372767	2
		UGT79B175	OR372768	
28	*Phaseolus vulgaris*	UGT79B176	OR372723	5
		UGT79B177	OR372724	

		UGT79B178	OR372725	
		UGT79B179	OR372726	
		UGT79B180	OR372727	
29	*Bituminaria bituminosa*	UGT79B181	OR372769	1
30	*Lupinus albus*	UGT79B182	OR372770	1
31	*Prosopis alba*	UGT79B183	OR372719	1
32	*Copaifera officianalis*	UGT79B184	OR372771	1
33	*Glycyrrhiza lepidota*	UGT79B185	OR372772	1
34	*Senna tora*	UGT79B186	OR372773	1
35	*Lathyrus sativus*	UGT79B187	OR372712	1
36	*Gompholobium polymorphum*	UGT79B188	OR372705	1
37	*Lotus japonicus*	UGT79B189	OR372737	1
38	*Xanthicercis zambesiaca*	UGT79B190	OR372774	1
39	*Pueraria montana* var. *lobata*	UGT79B100<br>UGT79B101<br>UGT79B102	OR372720<br>OR372721<br>OR372722	3

Supplementary Figures

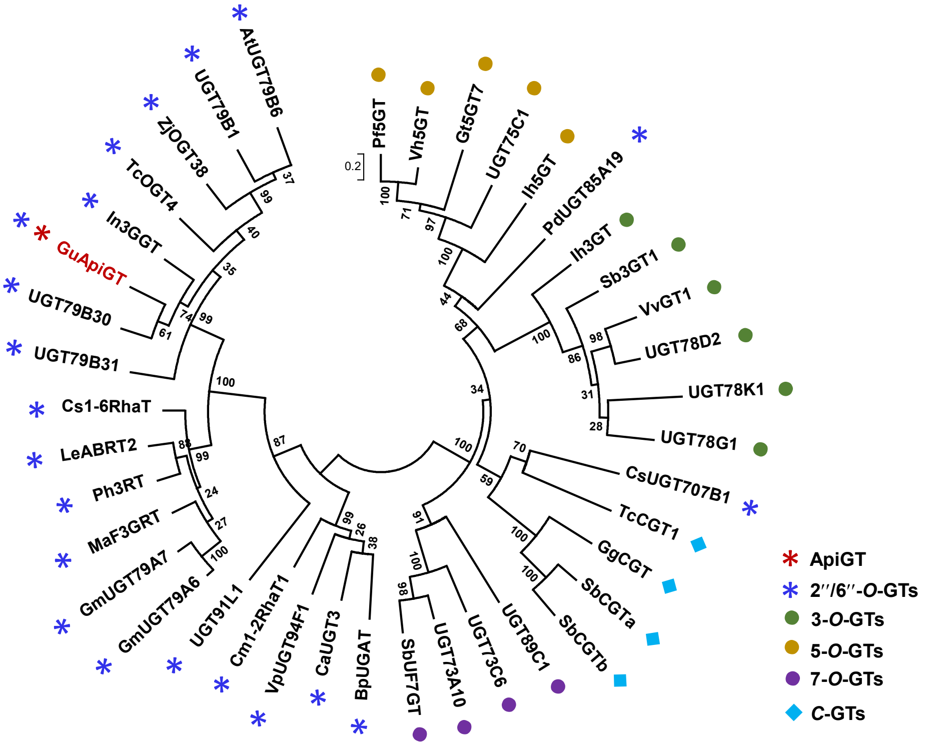

Supplementary Fig. 1 Phylogenetic analysis of GuApiGT (MSTRG.23171.4) with reported UGTs using MEGA6 software with the maximum likelihood method. The bootstrap consensus tree inferred from 1,000 replicates was taken to represent the evolutionary history of the taxa analyzed. All the GenBank accession numbers used in this study are listed in **Supplementary Table 1**.

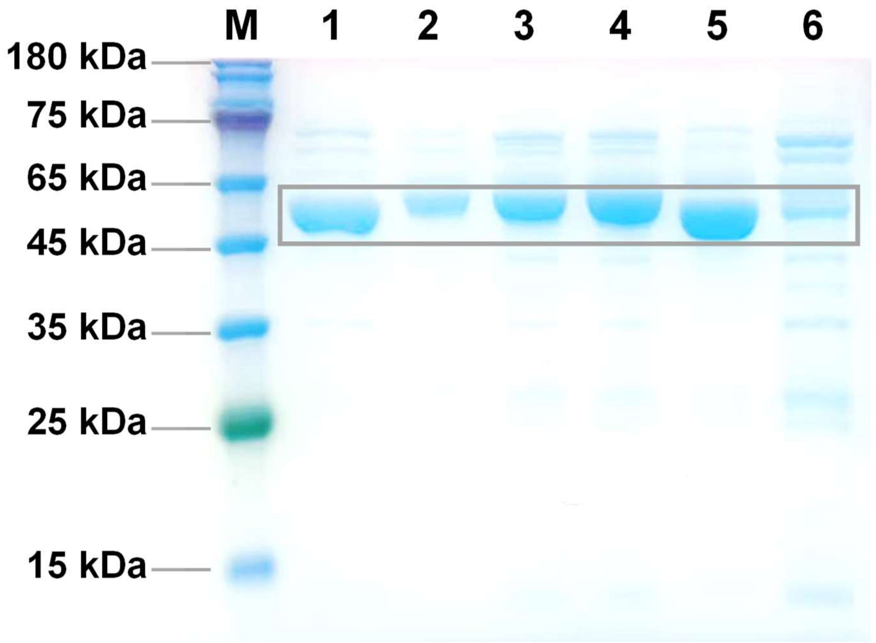

Supplementary Fig. 2 SDS-PAGE analysis of recombinant ApiGTs and GuGT53 purified by Ni affinity chromatography. Lane M: protein marker; Lane 1, purified GuApiGT; Lane 2, purified GuGT53; Lane 3, purified GgApiGT; Lane 4, purified GiApiGT; Lane 5, purified SsApiGT; Lane 6, purified PtApiGT.

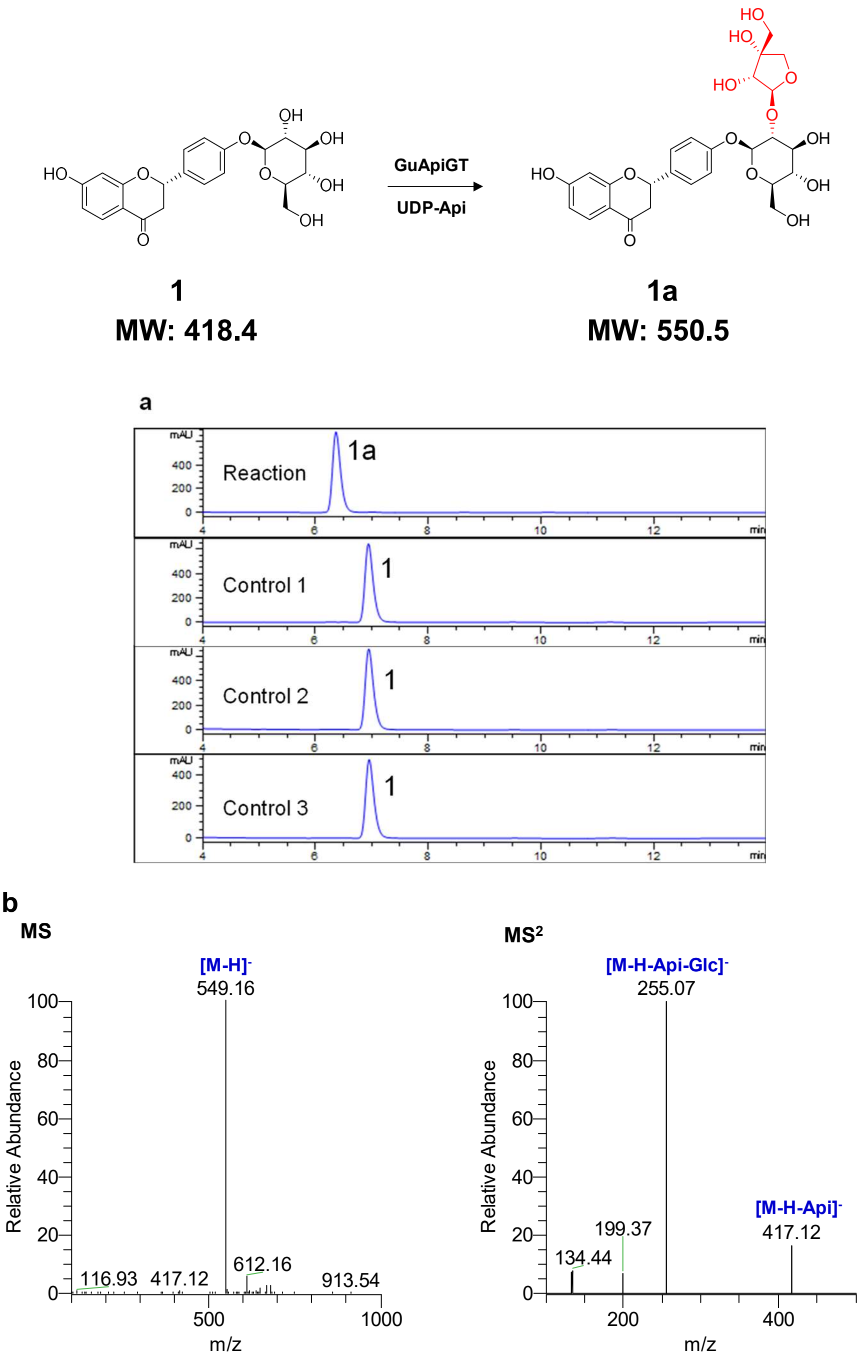

Supplementary Fig. 3 HPLC and LC/MS analyses of GuApiGT catalytic reaction mixture for substrate **1**. **a**, HPLC analysis of GuApiGT catalyzed product using **1** as the substrate. **b**, (-)-ESI-MS and MS^2 spectra of product **1a**. UDP-Api was produced by adding UDP-GlcA, purified UAXS, and NAD$^+$ to the mixed system. Control 1, UDP-GlcA-free. Control 2, UAXS-free. Control 3, UDP-Xyl to replace UDP-Api supply system. The analytical conditions are given in **Supplementary Table 3**.

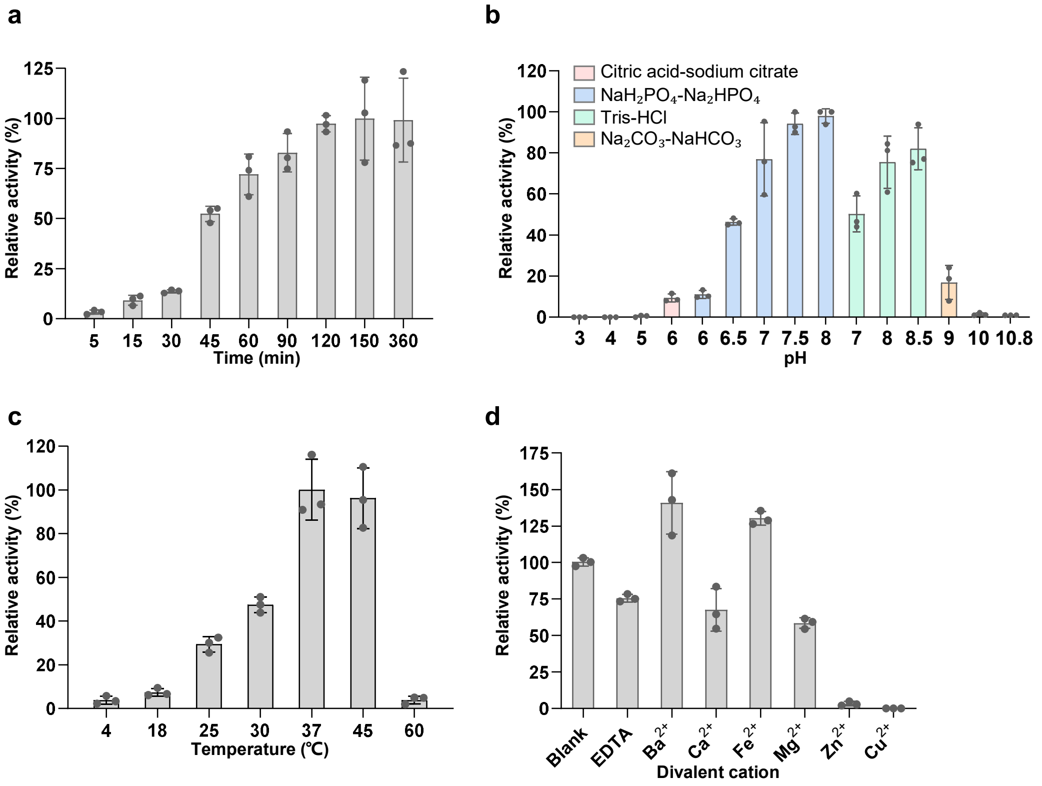

Supplementary Fig. 4 Effects of reaction time (**a**), reaction buffer (**b**), temperature (**c**), and divalent metal ions (**d**) on enzyme activity of GuApiGT. UDP-Api was used as the sugar donor and **2** as the acceptor. GuApiGT showed the optimal reaction temperature at 37°C, and the maximum activity at pH 8.0. Some divalent cations could suppress the catalytic activities. Data are presented as mean values ± SD (*n*=3 biologically independent samples). The source data underlying figures (**a-d**) are provided in a Source Data file.

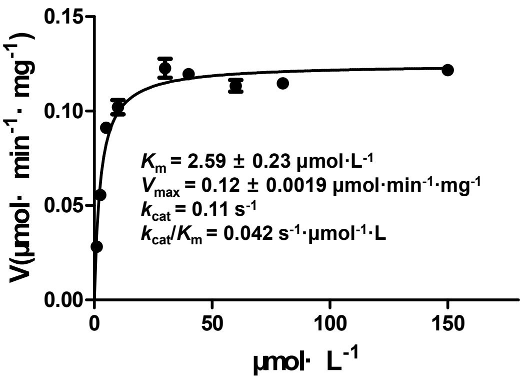

Supplementary Fig. 5 Determination of kinetic parameters for recombinant GuApiGT (n=3). The apparent K_m value was calculated from Michaelis-Menten plot with varying concentrations of compound **2** (isoliquiritin). Data are presented as mean values ± SD (n=3 biologically independent samples). The source data underlying figure are provided in a Source Data file.

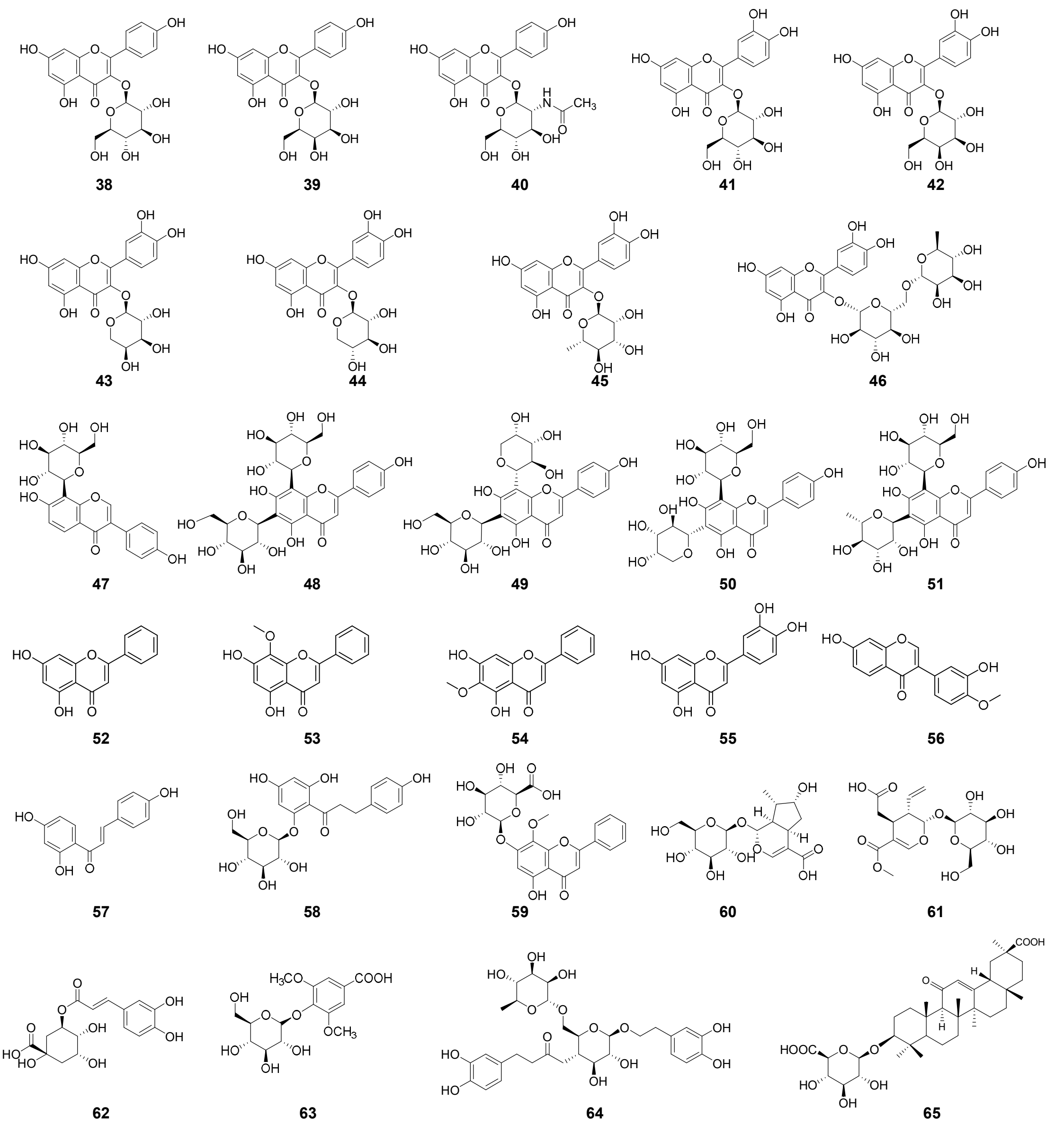

Supplementary Fig. 6 Structures of substrates **38-65** that cannot be catalyzed by GuApiGT. (**38-46**, flavonoid 3-*O*-glycosides; **47-51**, flavonoid *C*-glycosides; **52-57**, free flavonoids; **58-65**, other substrates).

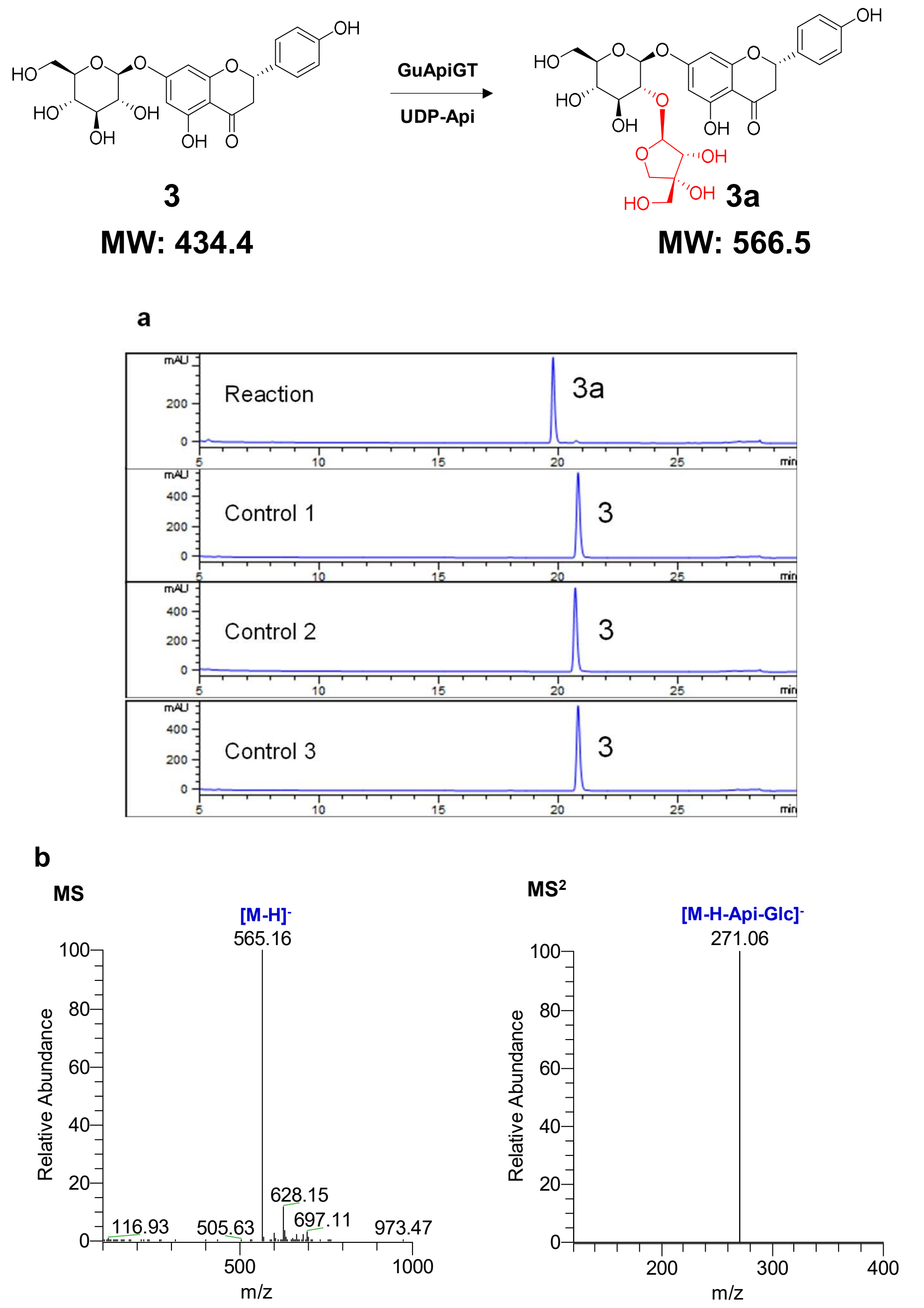

Supplementary Fig. 7 HPLC and LC/MS analyses of GuApiGT catalytic reaction mixture for substrate **3**. **a**, HPLC analysis of GuApiGT catalyzed product using **3** as the substrate. **b**, (-)-ESI-MS and MS^2 spectra of product **3a**. UDP-Api was produced by adding UDP-GlcA, purified UAXS and NAD^+ to the mixed system. Control 1, UDP-GlcA-free. Control 2, UAXS-free. Control 3, UDP-Xyl to replace UDP-Api supply system. The analysis conditions are given in **Supplementary Table 3**.

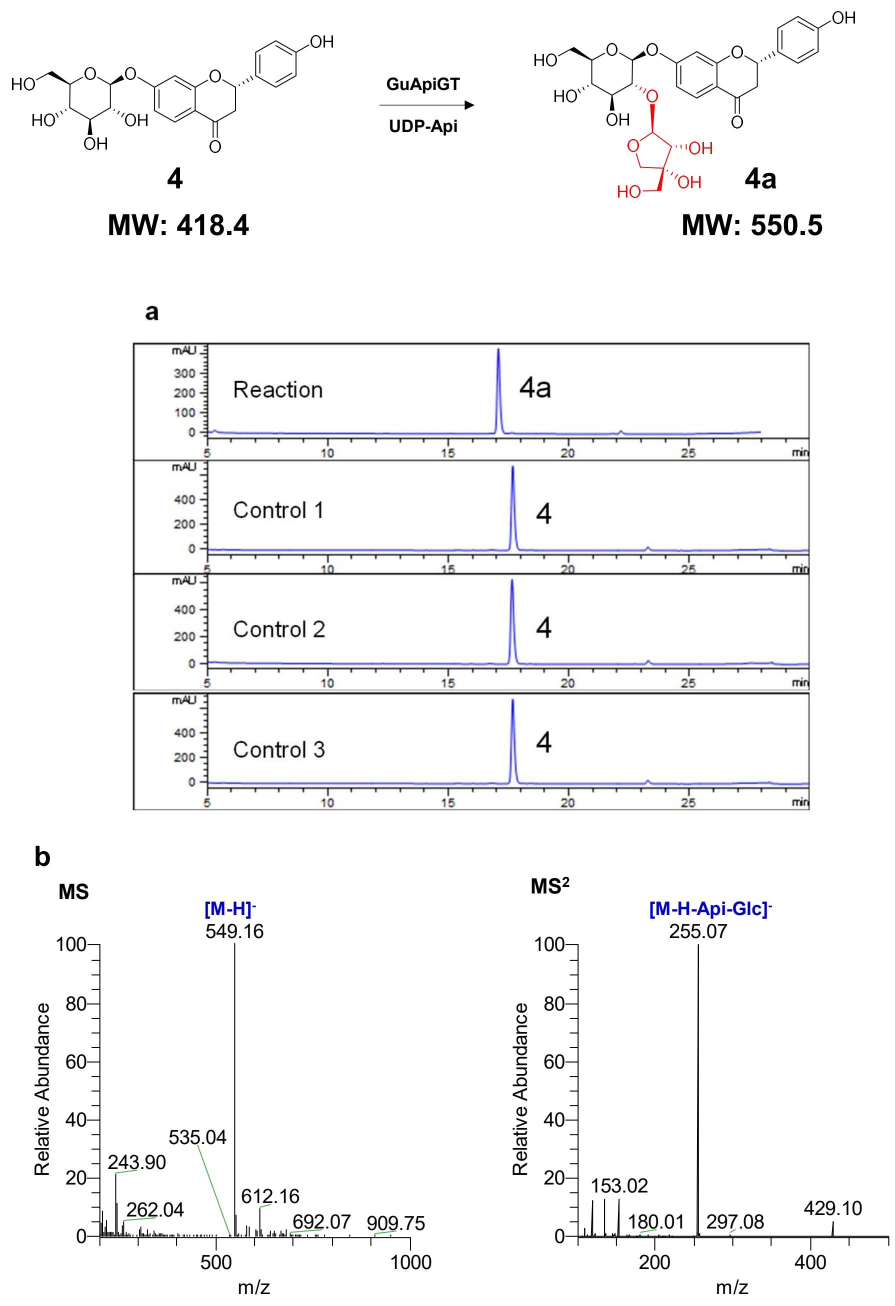

Supplementary Fig. 8 HPLC and LC/MS analyses of GuApiGT catalytic reaction mixture for substrate **4**. **a**, HPLC analysis of GuApiGT catalyzed product using **4** as the substrate. **b**, (-)-ESI-MS and MS^2 spectra of product **4a**. UDP-Api was produced by adding UDP-GlcA, purified UAXS and NAD^+ to the mixed system. Control 1, UDP-GlcA-free. Control 2, UAXS-free. Control 3, UDP-Xyl to replace UDP-Api supply system. The analysis conditions are given in **Supplementary Table 3**.

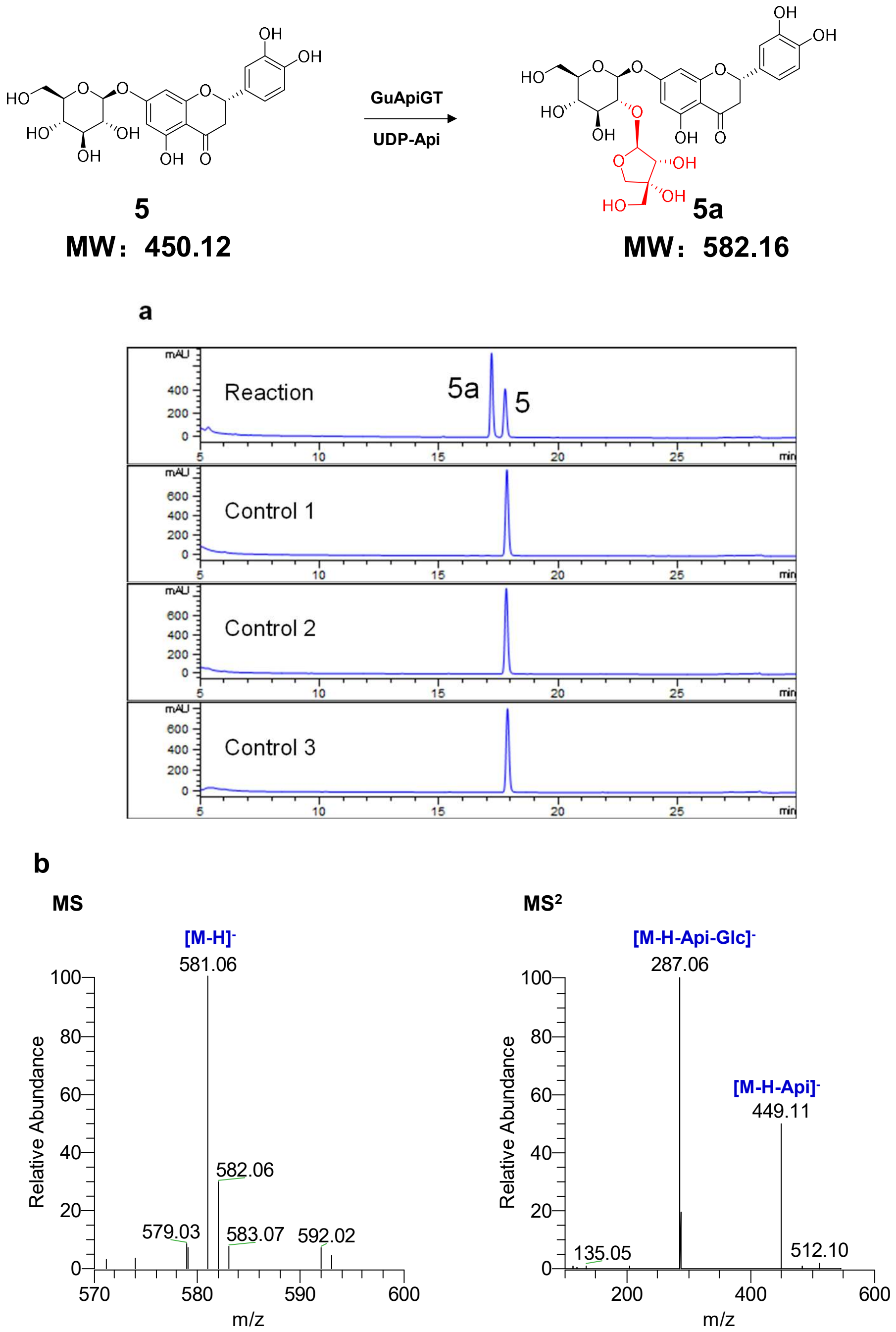

Supplementary Fig. 9 HPLC and LC/MS analyses of GuApiGT catalytic reaction mixture for substrate **5**. **a**, HPLC analysis of GuApiGT catalyzed product using **5** as the substrate. **b**, (-)-ESI-MS and MS^2 spectra of product **5a**. UDP-Api was produced by adding UDP-GlcA, purified UAXS and NAD^+ to the mixed system. Control 1, UDP-GlcA-free. Control 2, UAXS-free. Control 3, UDP-Xyl to replace UDP-Api supply system. The analysis conditions are given in **Supplementary Table 3**.

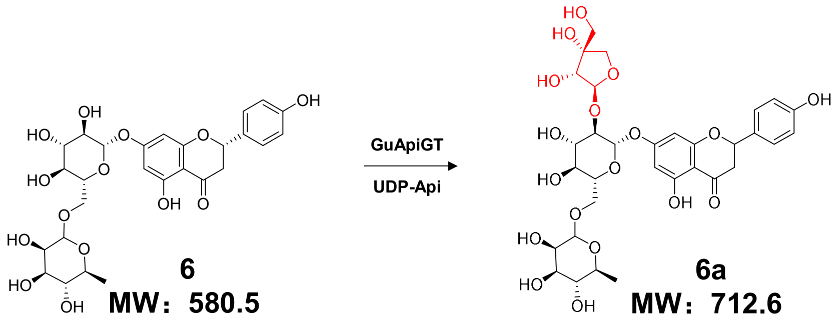

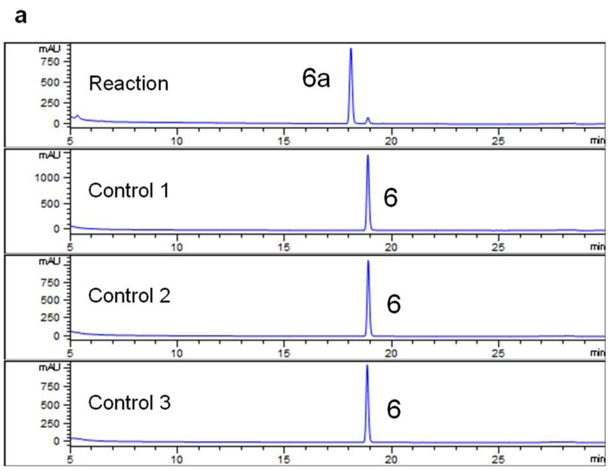

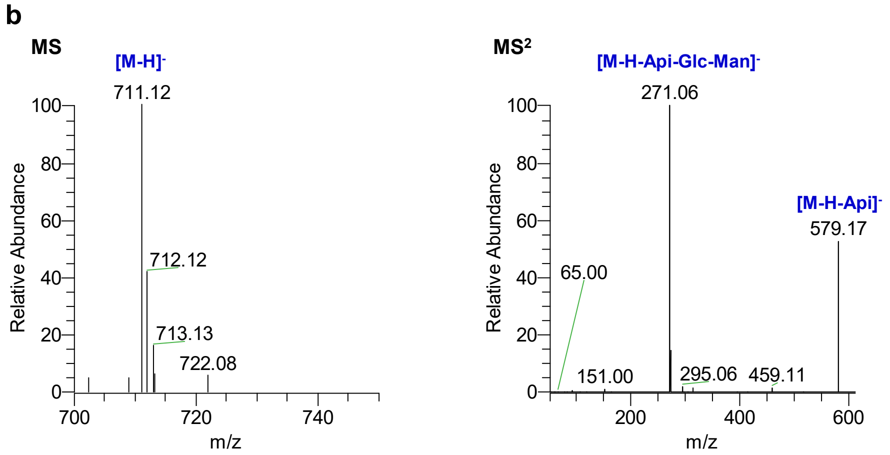

Supplementary Fig. 10 HPLC and LC/MS analyses of GuApiGT catalytic reaction mixture for substrate **6**. **a**, HPLC analysis of GuApiGT catalyzed product using **6** as the substrate. **b**, (-)-ESI-MS and MS^2 spectra of product **6a**. UDP-Api was produced by adding UDP-GlcA, purified UAXS and NAD^+ to the mixed system. Control 1, UDP-GlcA-free. Control 2, UAXS-free. Control 3, UDP-Xyl to replace UDP-Api supply system. The analysis conditions are given in **Supplementary Table 3**.

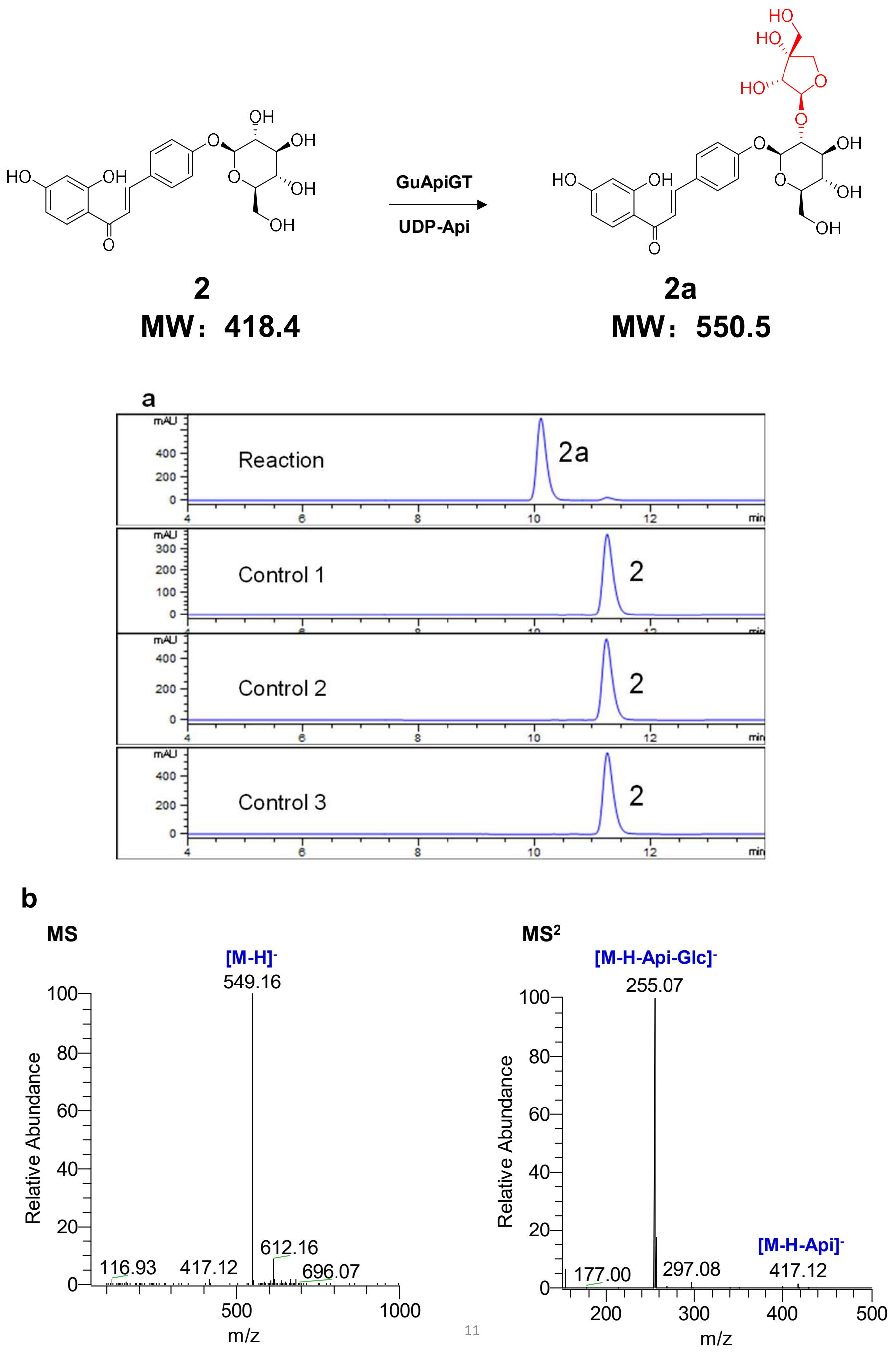

Supplementary Fig. 11 HPLC and LC/MS analyses of GuApiGT catalytic reaction mixture for substrate **2**. **a**, HPLC analysis of GuApiGT catalyzed product using **2** as the substrate. **b**, (-)-ESI-MS and MS^2 spectra of product **2a**. UDP-Api was produced by adding UDP-GlcA, purified UAXS and NAD^+ to the mixed system. Control 1, UDP-GlcA-free. Control 2, UAXS-free. Control 3, UDP-Xyl to replace UDP-Api supply system. The analysis conditions are given in **Supplementary Table 3**.

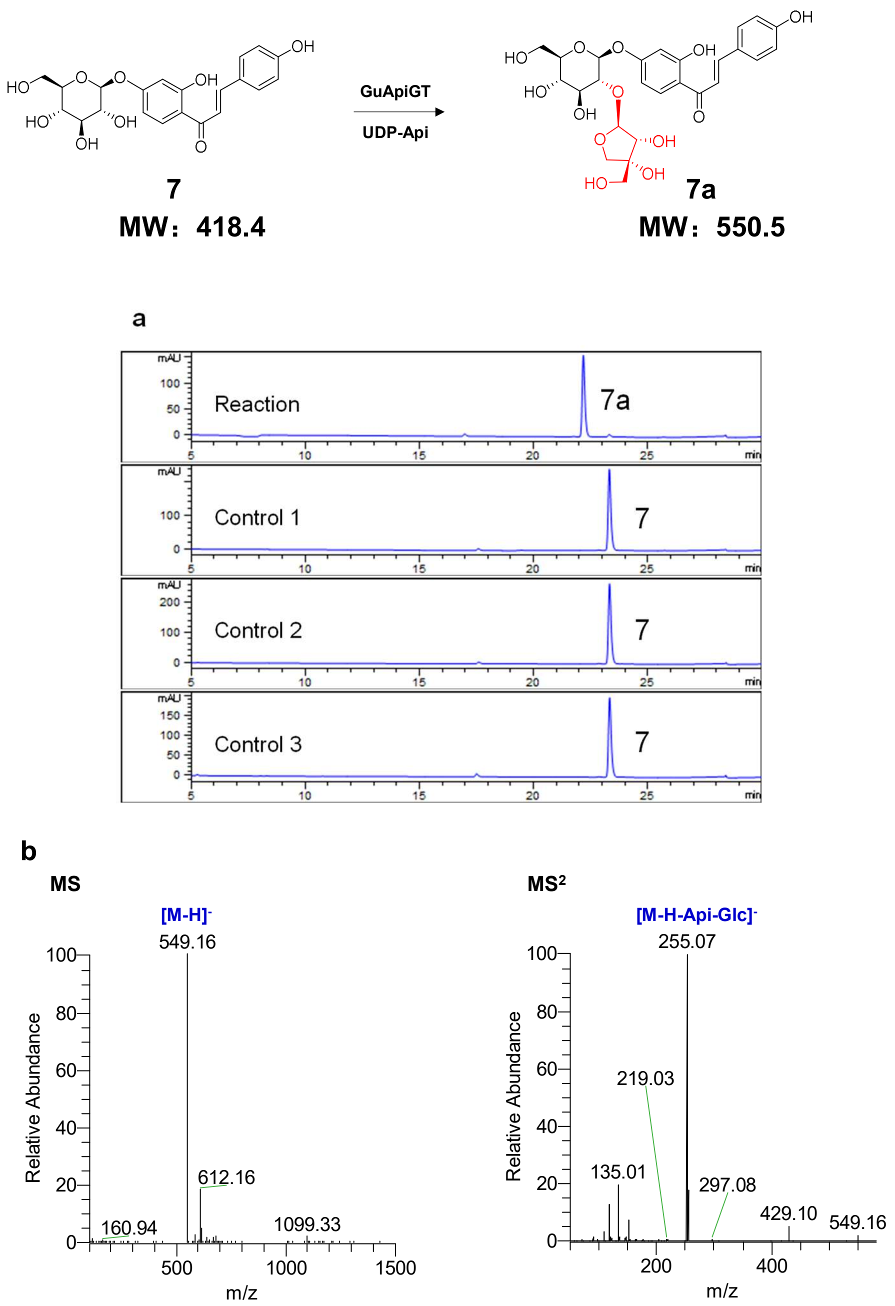

Supplementary Fig. 12 HPLC and LC/MS analyses of GuApiGT catalytic reaction mixture for substrate **7**. **a**, HPLC analysis of GuApiGT catalyzed product using **7** as the substrate. **b**, (-)-ESI-MS and MS^2 spectra of product **7a**. UDP-Api was produced by adding UDP-GlcA, purified UAXS and NAD$^+$ to the mixed system. Control 1, UDP-GlcA-free. Control 2, UAXS-free. Control 3, UDP-Xyl to replace UDP-Api supply system. The analysis conditions are given in **Supplementary Table 3**.

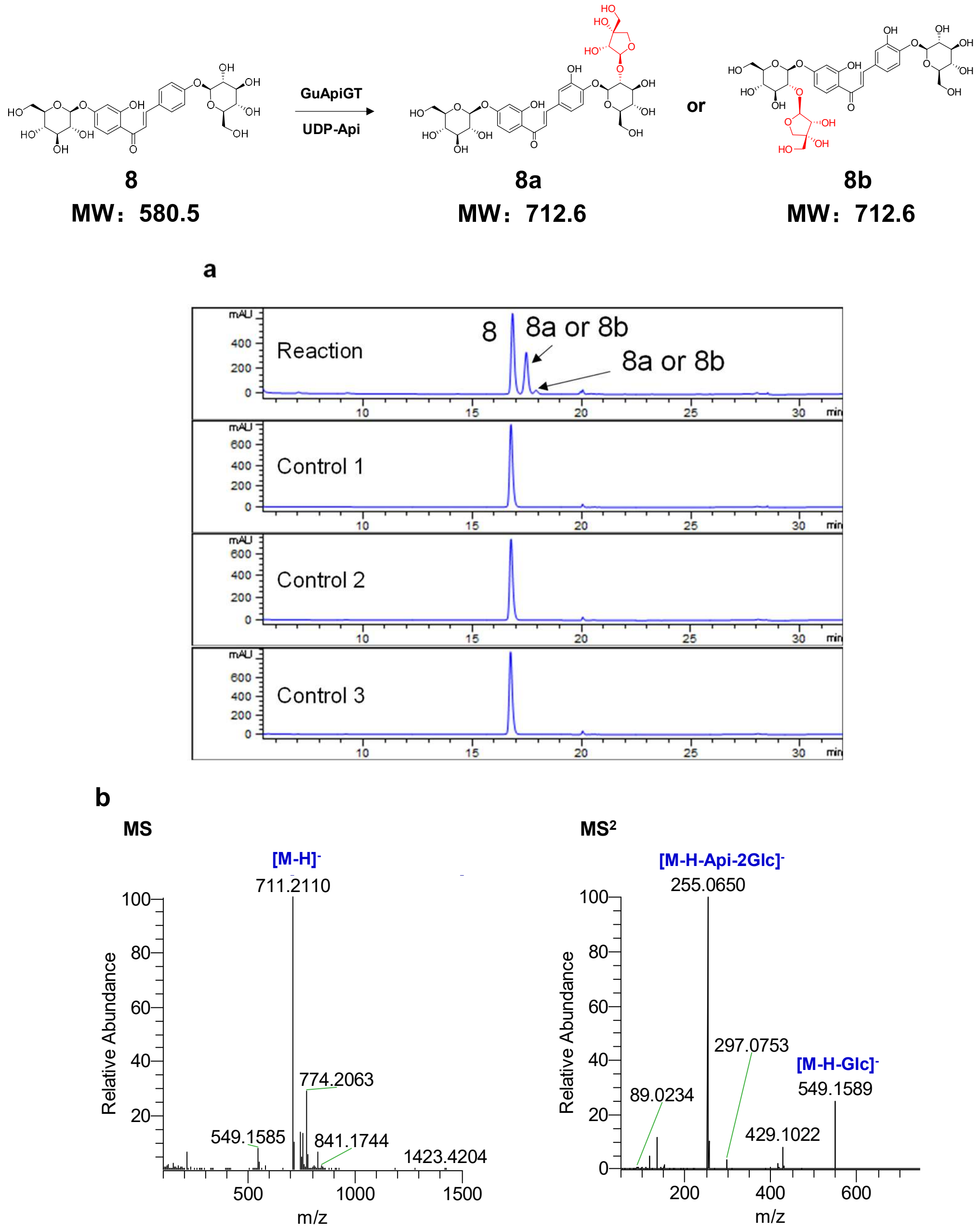

Supplementary Fig. 13 HPLC and LC/MS analyses of GuApiGT catalytic reaction mixture for substrate **8**. **a**, HPLC analysis of GuApiGT catalyzed product using **8** as the substrate. **b**, (-)-ESI-MS and MS^2 spectra of product **8a** or **8b**. UDP-Api was produced by adding UDP-GlcA, purified UAXS and NAD^+ to the mixed system. Control 1, UDP-GlcA-free. Control 2, UAXS-free. Control 3, UDP-Xyl to replace UDP-Api supply system. The analysis conditions are given in **Supplementary Table 3**.

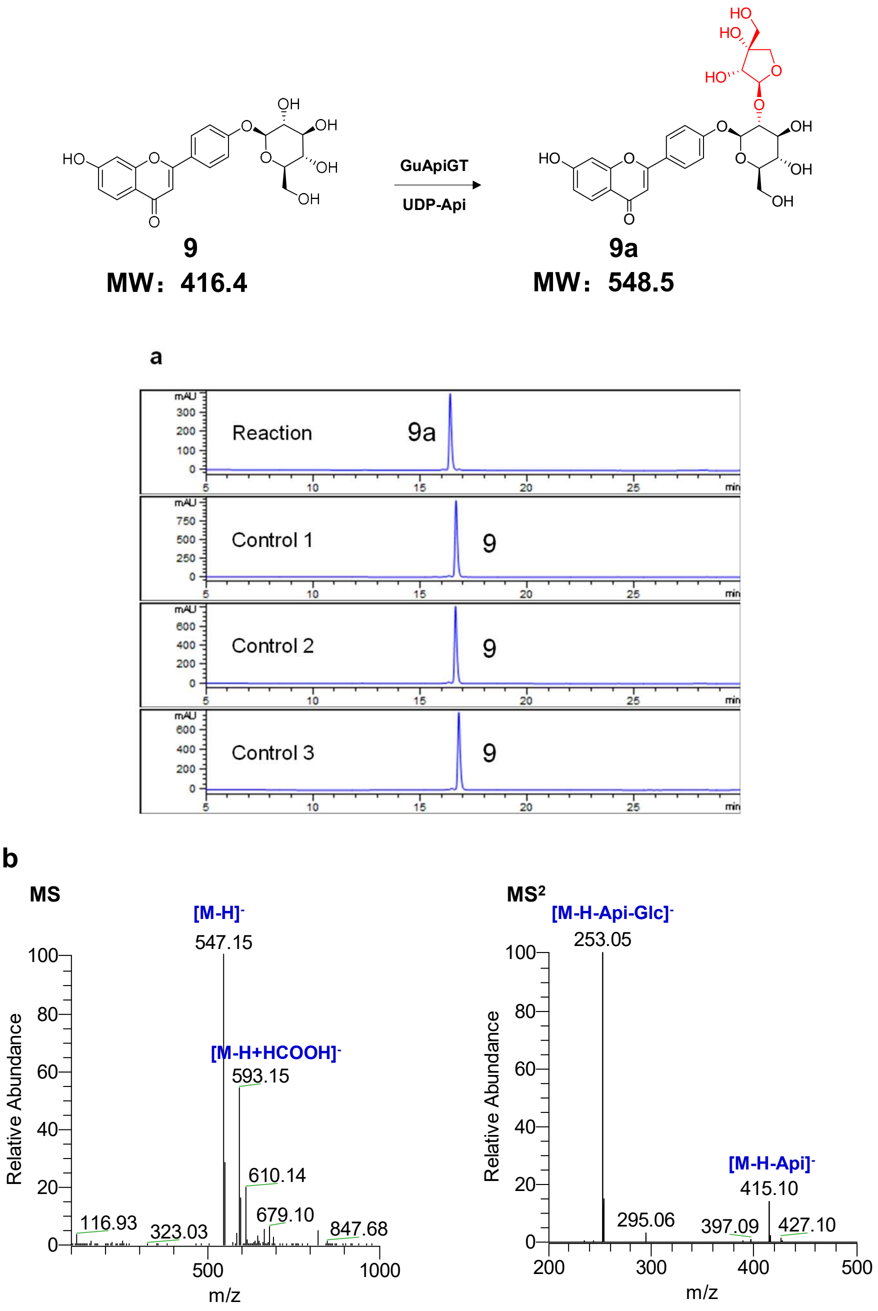

Supplementary Fig. 14 HPLC and LC/MS analyses of GuApiGT catalytic reaction mixture for substrate **9**. **a**, HPLC analysis of GuApiGT catalyzed product using **9** as the substrate. **b**, (-)-ESI-MS and MS^2 spectra of product **9a**. UDP-Api was produced by adding UDP-GlcA, purified UAXS and NAD^+ to the mixed system. Control 1, UDP-GlcA-free. Control 2, UAXS-free. Control 3, UDP-Xyl to replace UDP-Api supply system. The analysis conditions are given in **Supplementary Table 3**.

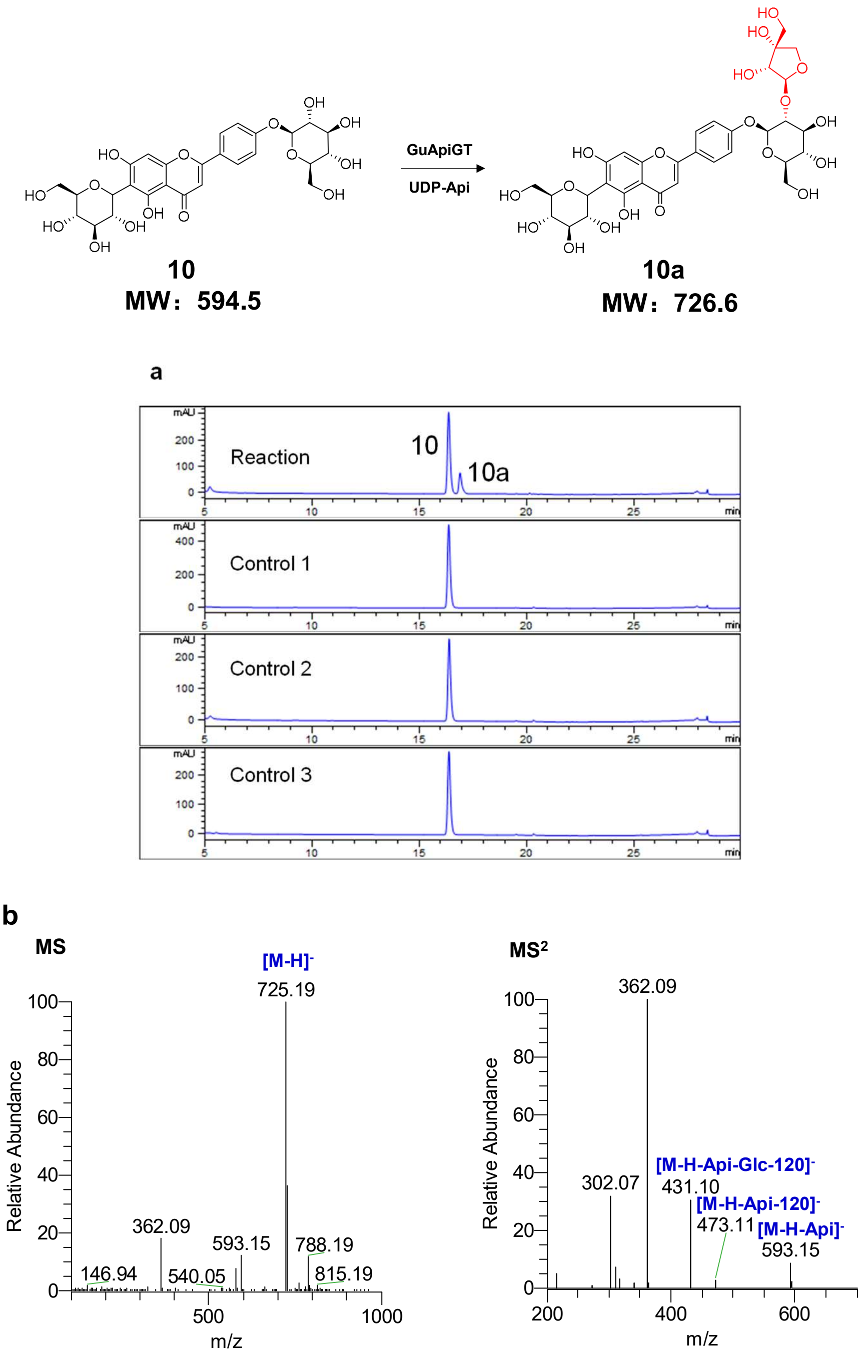

Supplementary Fig. 15 HPLC and LC/MS analyses of GuApiGT catalytic reaction mixture for substrate **10**. **a**, HPLC analysis of GuApiGT catalyzed product using **10** as the substrate. **b**, (-)-ESI-MS and MS^2 spectra of product **10a**. UDP-Api was produced by adding UDP-GlcA, purified UAXS and NAD^+ to the mixed system. Control 1, UDP-GlcA-free. Control 2, UAXS-free. Control 3, UDP-Xyl to replace UDP-Api supply system. The analysis conditions are given in **Supplementary Table 3**.

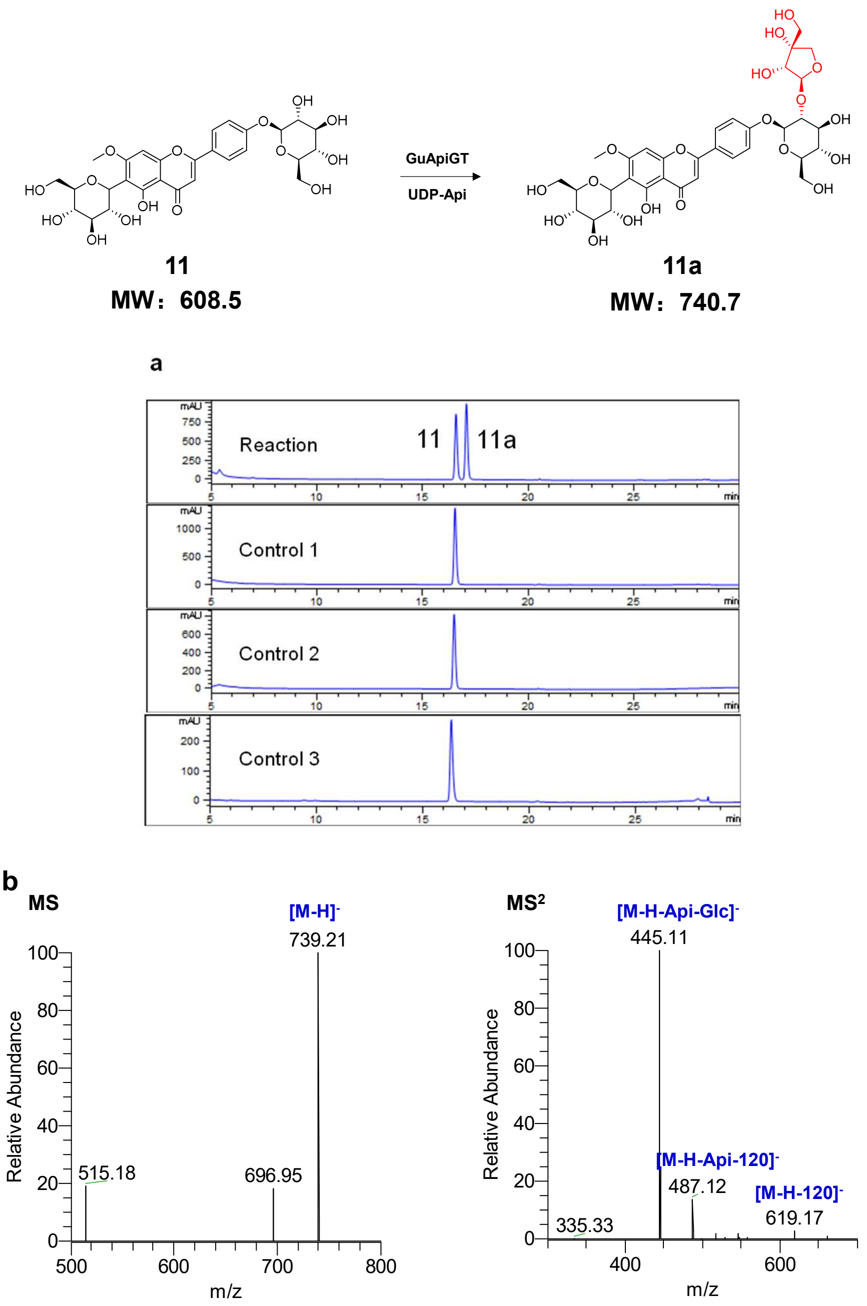

Supplementary Fig. 16 HPLC and LC/MS analyses of GuApiGT catalytic reaction mixture for substrate **11**. **a**, HPLC analysis of GuApiGT catalyzed product using **11** as the substrate. **b**, (-)-ESI-MS and MS^2 spectra of product **11a**. UDP-Api was produced by adding UDP-GlcA, purified UAXS and NAD$^+$ to the mixed system. Control 1, UDP-GlcA-free. Control 2, UAXS-free. Control 3, UDP-Xyl to replace UDP-Api supply system. The analysis conditions are given in **Supplementary Table 3**.

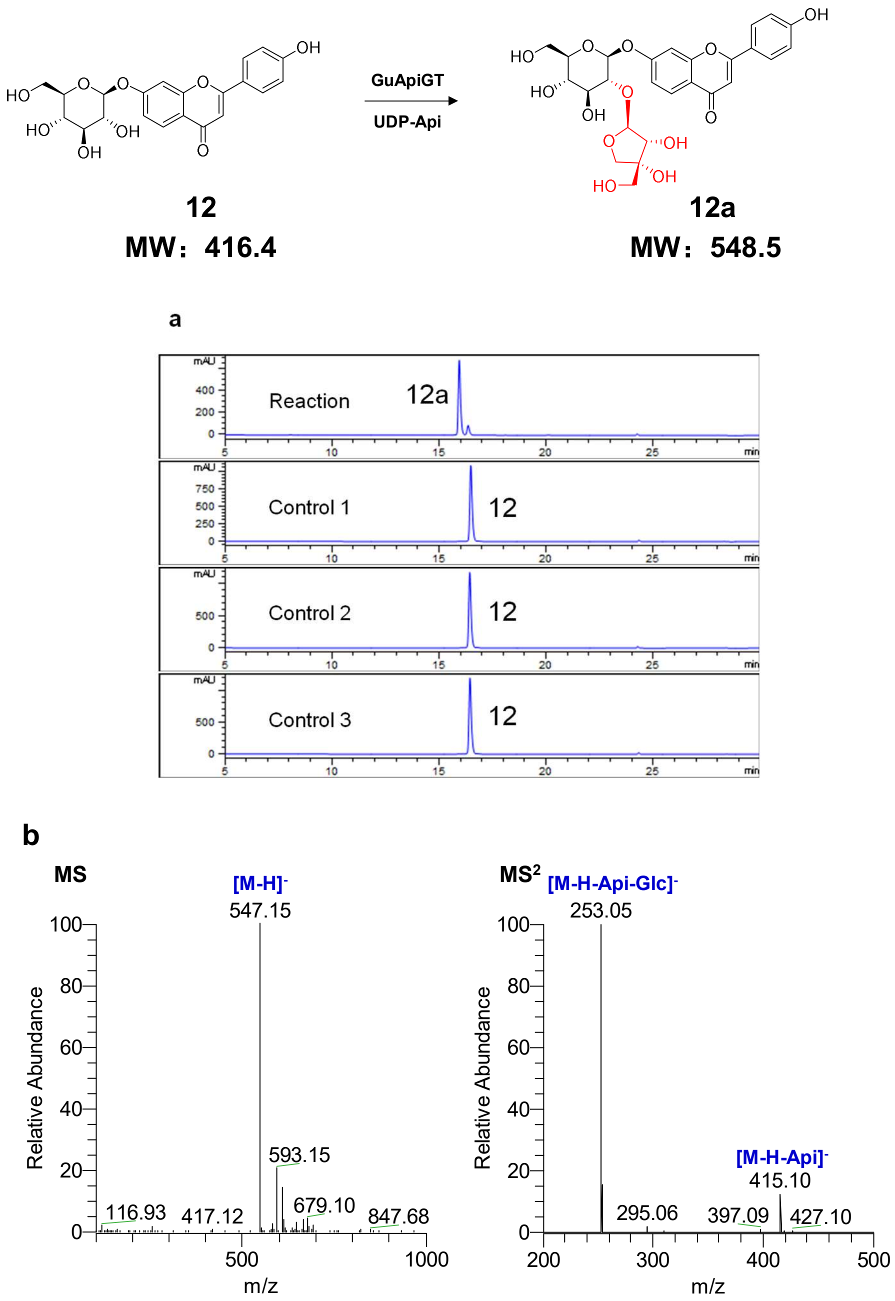

Supplementary Fig. 17 HPLC and LC/MS analyses of GuApiGT catalytic reaction mixture for substrate **12**. **a**, HPLC analysis of GuApiGT catalyzed product using **12** as the substrate. **b**, (-)-ESI-MS and MS^2 spectra of product **12a**. UDP-Api was produced by adding UDP-GlcA, purified UAXS and NAD$^+$ to the mixed system. Control 1, UDP-GlcA-free. Control 2, UAXS-free. Control 3, UDP-Xyl to replace UDP-Api supply system. The analysis conditions are given in **Supplementary Table 3**.

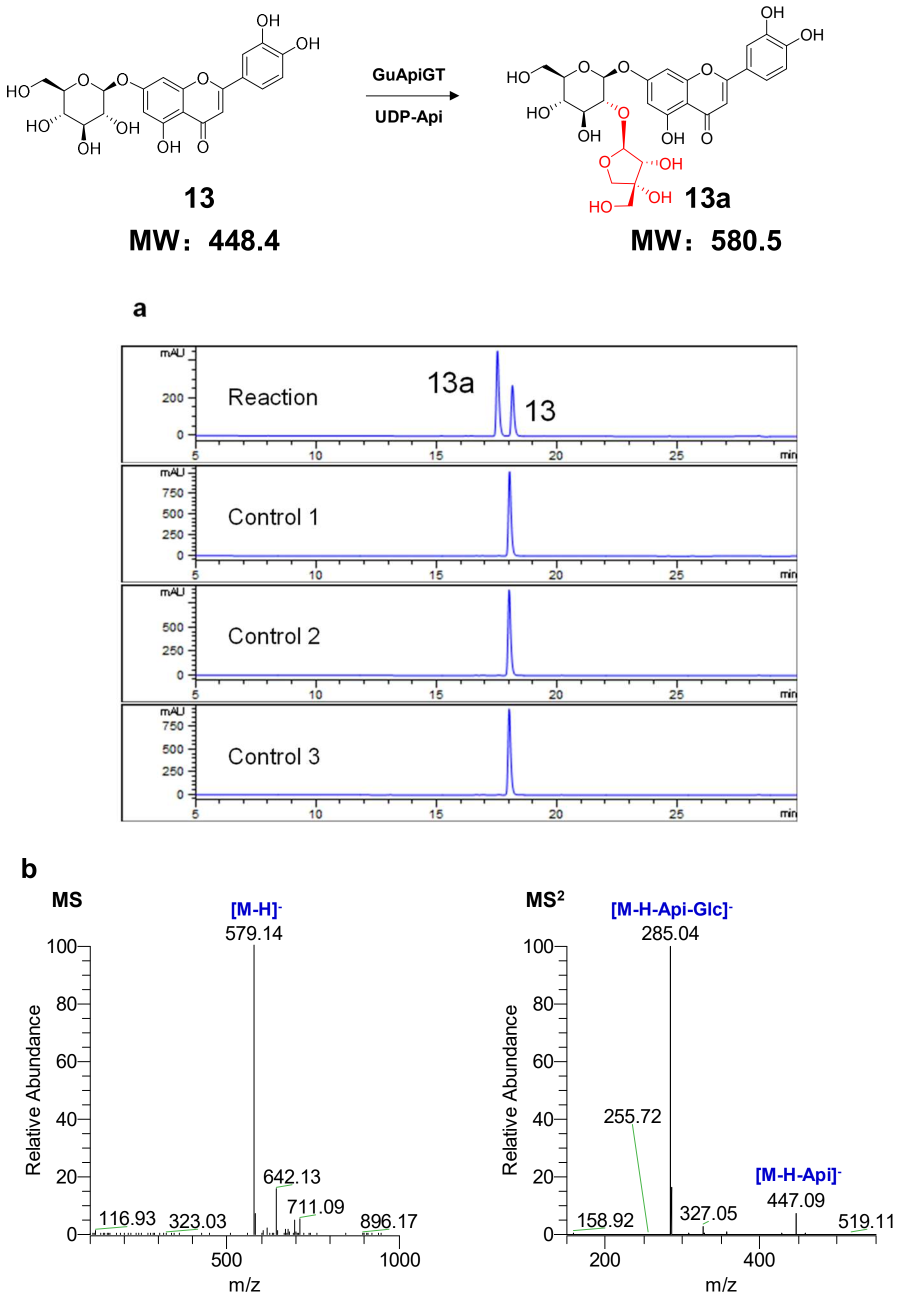

Supplementary Fig. 18 HPLC and LC/MS analyses of GuApiGT catalytic reaction mixture for substrate **13**. **a**, HPLC analysis of GuApiGT catalyzed product using **13** as the substrate. **b**, (-)-ESI-MS and MS^2 spectra of product **13a**. UDP-Api was produced by adding UDP-GlcA, purified UAXS and NAD^+ to the mixed system. Control 1, UDP-GlcA-free. Control 2, UAXS-free. Control 3, UDP-Xyl to replace UDP-Api supply system. The analysis conditions are given in **Supplementary Table 3**.

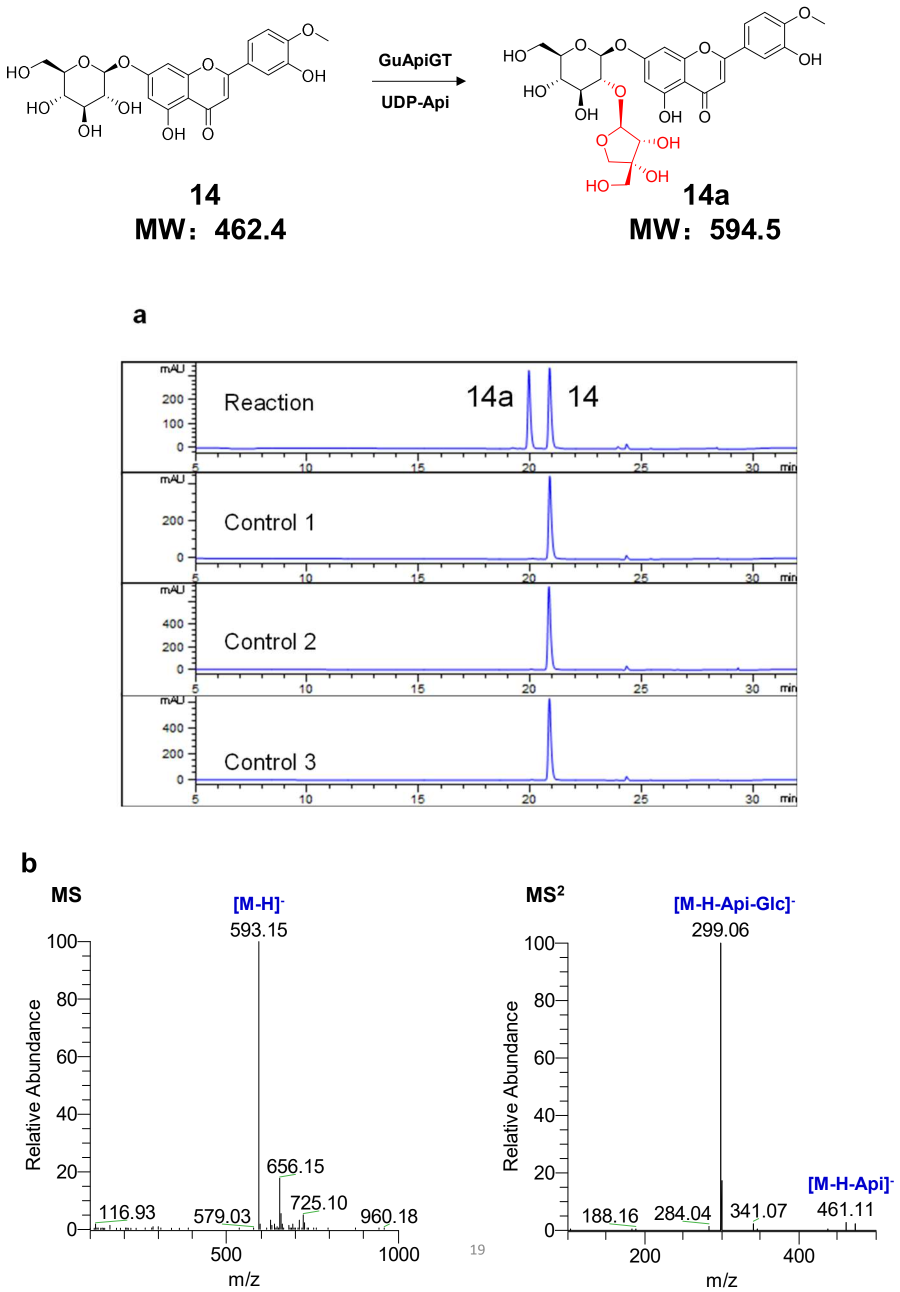

Supplementary Fig. 19 HPLC and LC/MS analyses of GuApiGT catalytic reaction mixture for substrate **14**. **a**, HPLC analysis of GuApiGT catalyzed product using **14** as the substrate. **b**, (-)-ESI-MS and MS^2 spectra of product **14a**. UDP-Api was produced by adding UDP-GlcA, purified UAXS and NAD^+ to the mixed system. Control 1, UDP-GlcA-free. Control 2, UAXS-free. Control 3, UDP-Xyl to replace UDP-Api supply system. The analysis conditions are given in **Supplementary Table 3**.

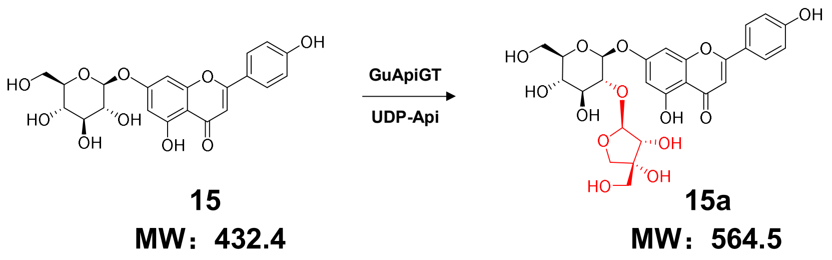

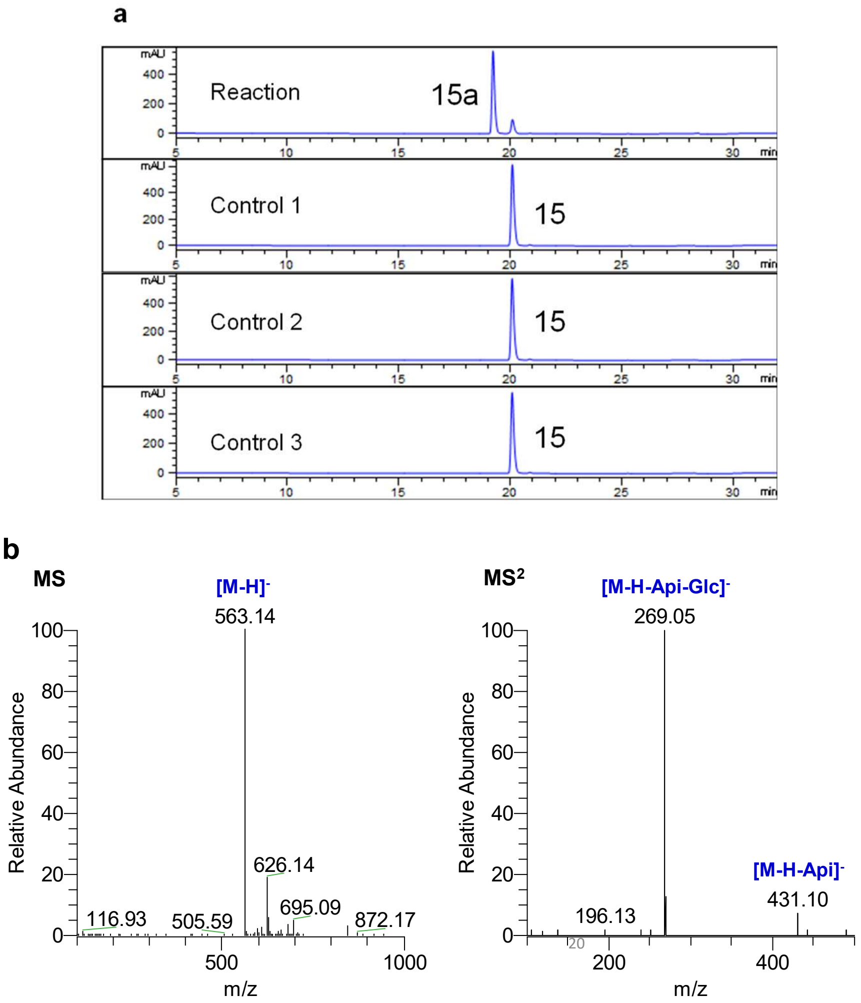

Supplementary Fig. 20 HPLC and LC/MS analyses of GuApiGT catalytic reaction mixture for substrate **15**. **a**, HPLC analysis of GuApiGT catalyzed product using **15** as the substrate. **b**, (-)-ESI-MS and MS^2 spectra of product **15a**. UDP-Api was produced by adding UDP-GlcA, purified UAXS and NAD$^+$ to the mixed system. Control 1, UDP-GlcA-free. Control 2, UAXS-free. Control 3, UDP-Xyl to replace UDP-Api supply system. The analysis conditions are given in **Supplementary Table 3**.

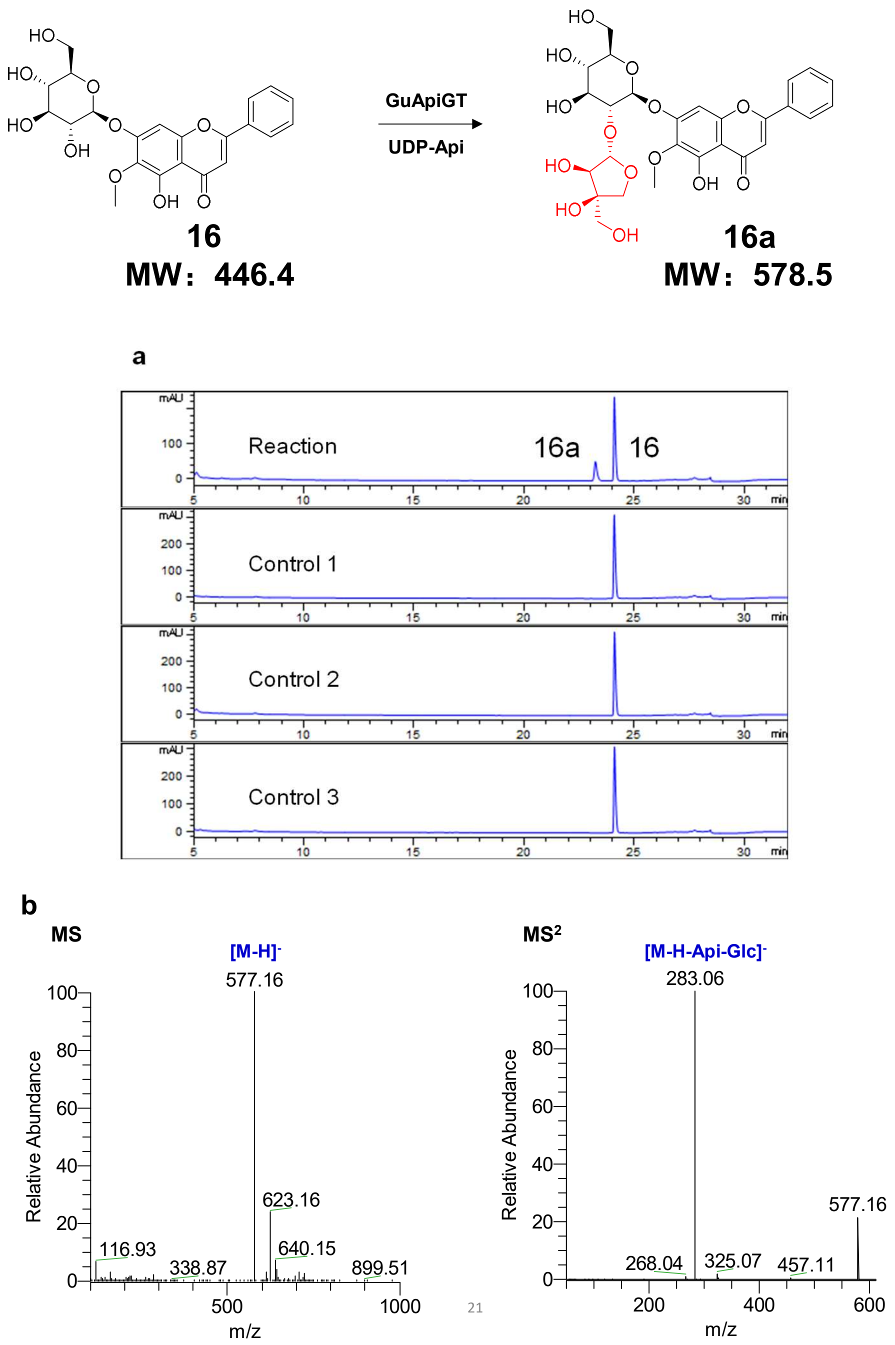

Supplementary Fig. 21 HPLC and LC/MS analyses of GuApiGT catalytic reaction mixture for substrate **16**. **a**, HPLC analysis of GuApiGT catalyzed product using **16** as the substrate. **b**, (-)-ESI-MS and MS^2 spectra of product **16a**. UDP-Api was produced by adding UDP-GlcA, purified UAXS and NAD^+ to the mixed system. Control 1, UDP-GlcA-free. Control 2, UAXS-free. Control 3, UDP-Xyl to replace UDP-Api supply system. The analysis conditions are given in **Supplementary Table 3**.

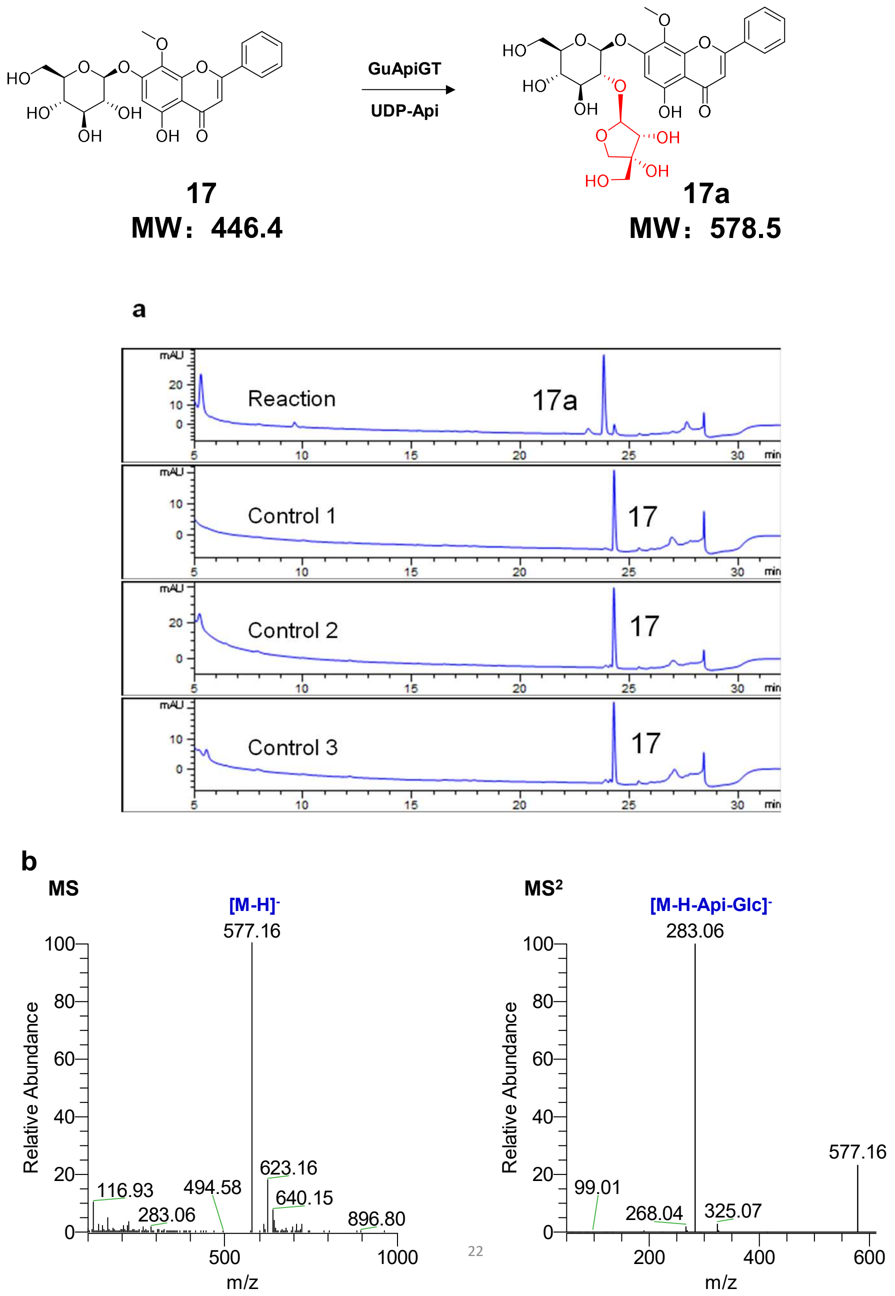

Supplementary Fig. 22 HPLC and LC/MS analyses of GuApiGT catalytic reaction mixture for substrate **17**. **a**, HPLC analysis of GuApiGT catalyzed product using **17** as the substrate. **b**, (-)-ESI-MS and MS^2 spectra of product **17a**. UDP-Api was produced by adding UDP-GlcA, purified UAXS and NAD^+ to the mixed system. Control 1, UDP-GlcA-free. Control 2, UAXS-free. Control 3, UDP-Xyl to replace UDP-Api supply system. The analysis conditions are given in **Supplementary Table 3**.

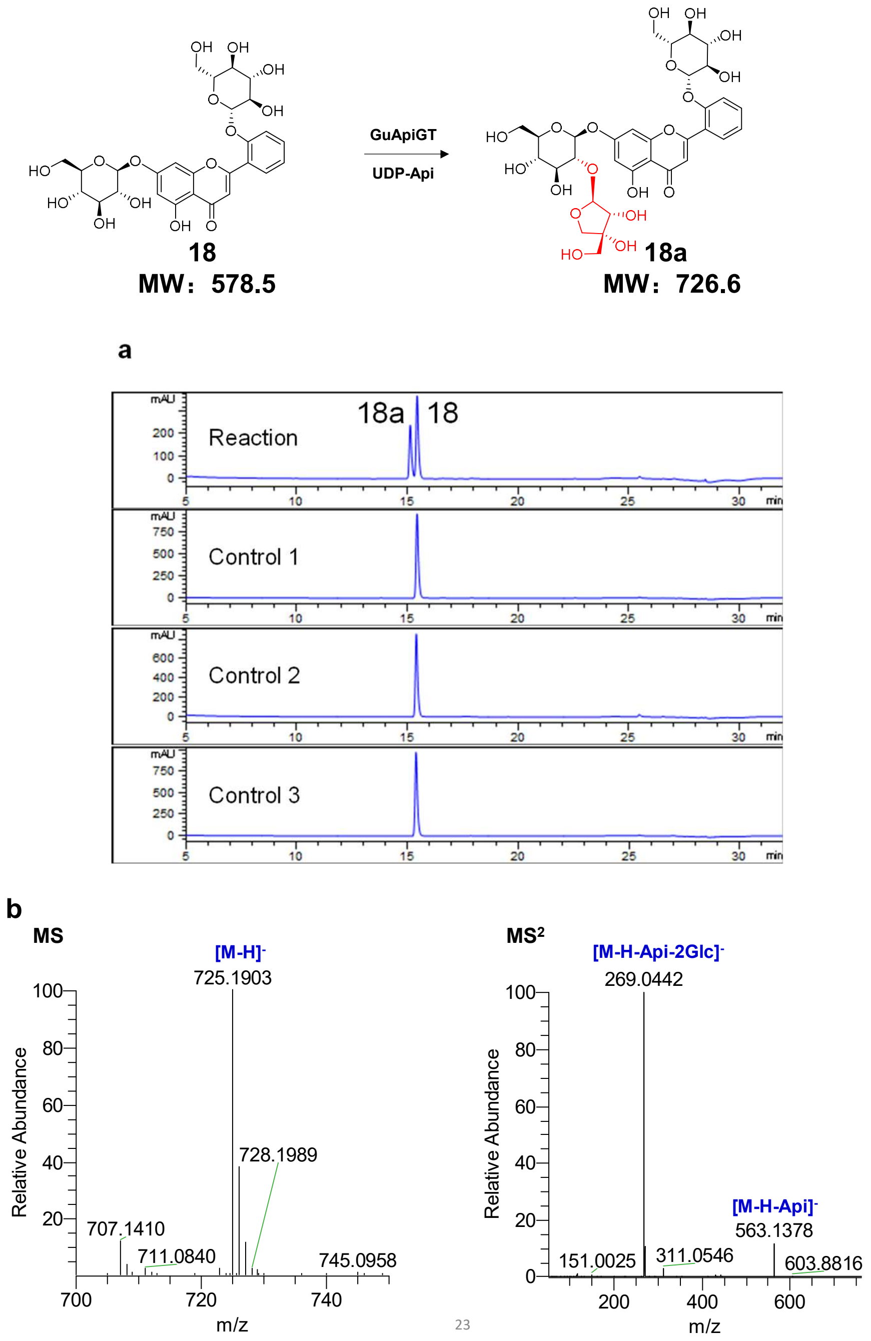

Supplementary Fig. 23 HPLC and LC/MS analyses of GuApiGT catalytic reaction mixture for substrate **18**. **a**, HPLC analysis of GuApiGT catalyzed product using **18** as the substrate. **b**, (-)-ESI-MS and MS^2 spectra of product **18a**. UDP-Api was produced by adding UDP-GlcA, purified UAXS and NAD^+ to the mixed system. Control 1, UDP-GlcA-free. Control 2, UAXS-free. Control 3, UDP-Xyl to replace UDP-Api supply system. The analysis conditions are given in **Supplementary Table 3**.

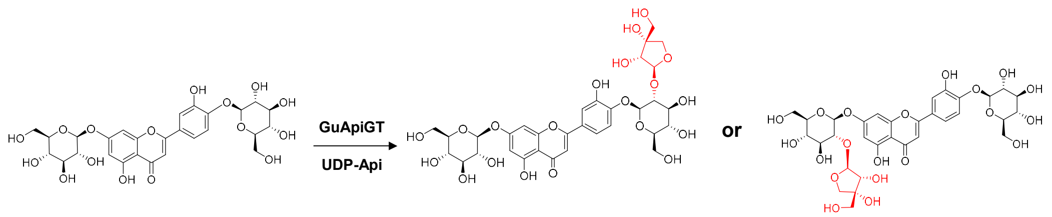

19 MW：610.15 **19a** MW：742.6 **19a** MW：742.6

a

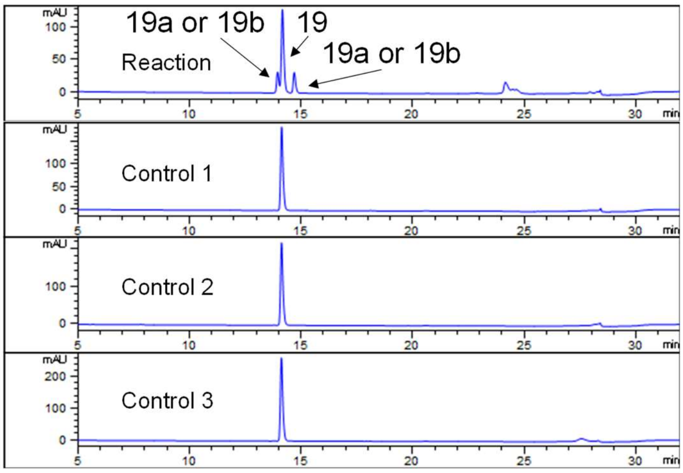

b

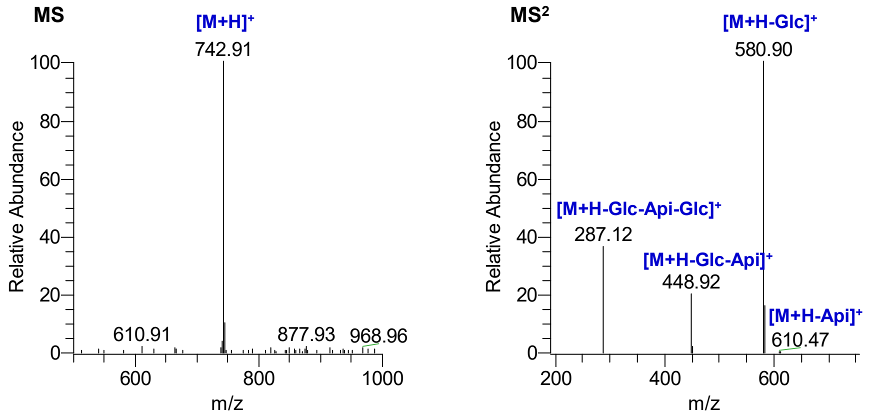

Supplementary Fig. 24 HPLC and LC/MS analyses of GuApiGT catalytic reaction mixture for substrate **19**. **a**, HPLC analysis of GuApiGT catalyzed product using **19** as the substrate. **b**, (+)-ESI-MS and MS^2 spectra of product **19a** or **19b**. UDP-Api was produced by adding UDP-GlcA, purified UAXS and NAD^+ to the mixed system. Control 1, UDP-GlcA-free. Control 2, UAXS-free. Control 3, UDP-Xyl to replace UDP-Api supply system. The analysis conditions are given in **Supplementary Table 3**.

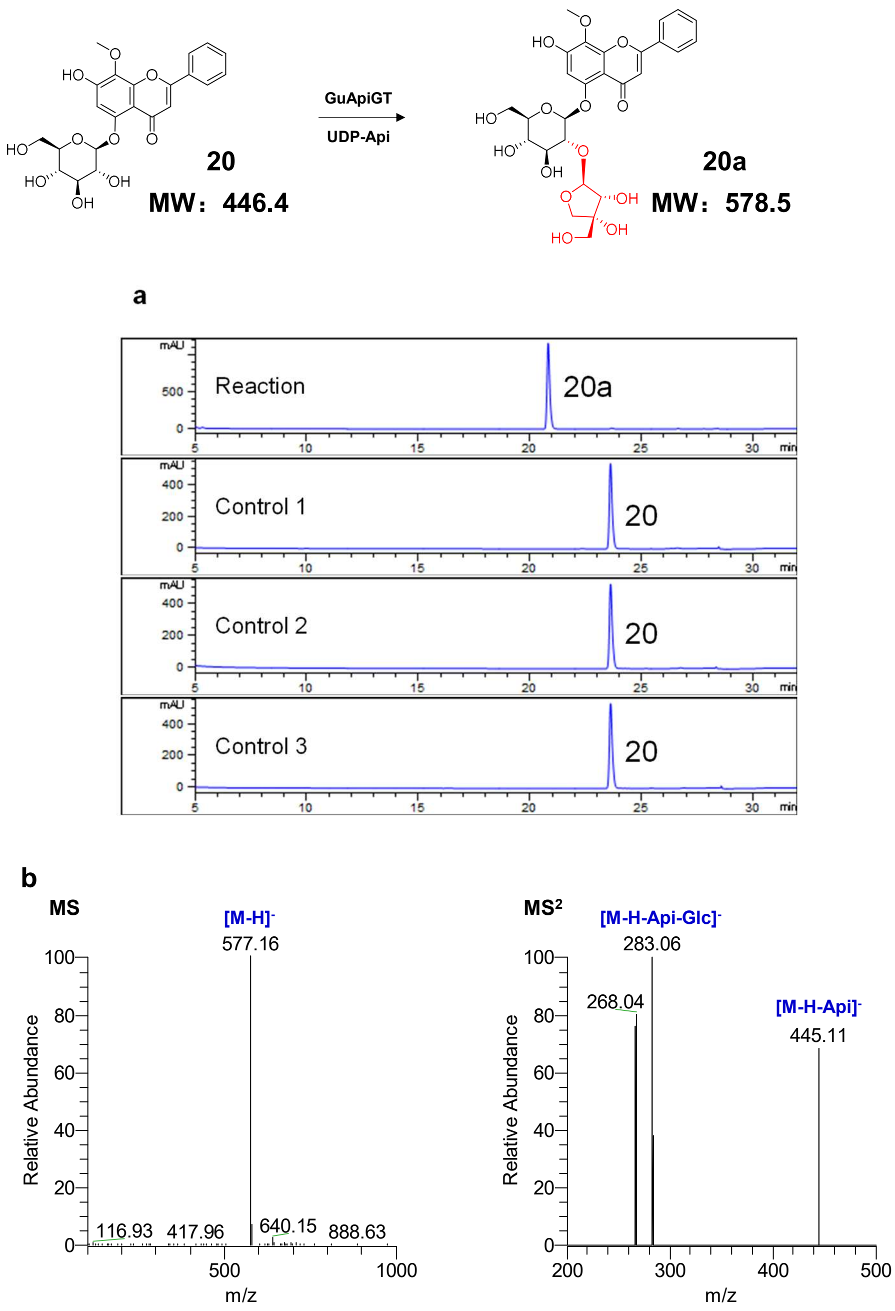

Supplementary Fig. 25 HPLC and LC/MS analyses of GuApiGT catalytic reaction mixture for substrate **20**. **a**, HPLC analysis of GuApiGT catalyzed product using **20** as the substrate. **b**, (-)-ESI-MS and MS^2 spectra of product **20a**. UDP-Api was produced by adding UDP-GlcA, purified UAXS and NAD^+ to the mixed system. Control 1, UDP-GlcA-free. Control 2, UAXS-free. Control 3, UDP-Xyl to replace UDP-Api supply system. The analysis conditions are given in **Supplementary Table 3**.

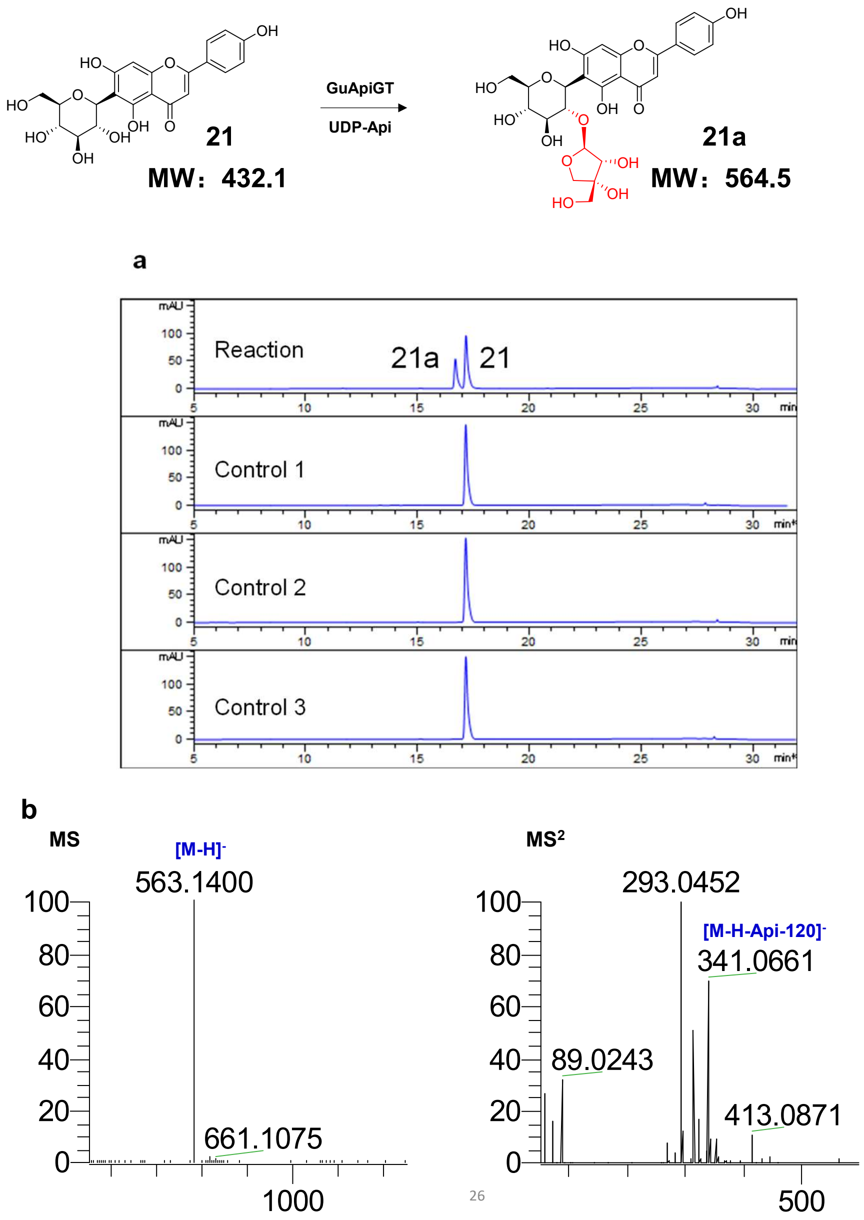

Supplementary Fig. 26 HPLC and LC/MS analyses of GuApiGT catalytic reaction mixture for substrate **21**. **a**, HPLC analysis of GuApiGT catalyzed product using **21** as the substrate. **b**, (-)-ESI-MS and MS^2 spectra of product **21a**. UDP-Api was produced by adding UDP-GlcA, purified UAXS and NAD^+ to the mixed system. Control 1, UDP-GlcA-free. Control 2, UAXS-free. Control 3, UDP-Xyl to replace UDP-Api supply system. The analysis conditions are given in **Supplementary Table 3**.

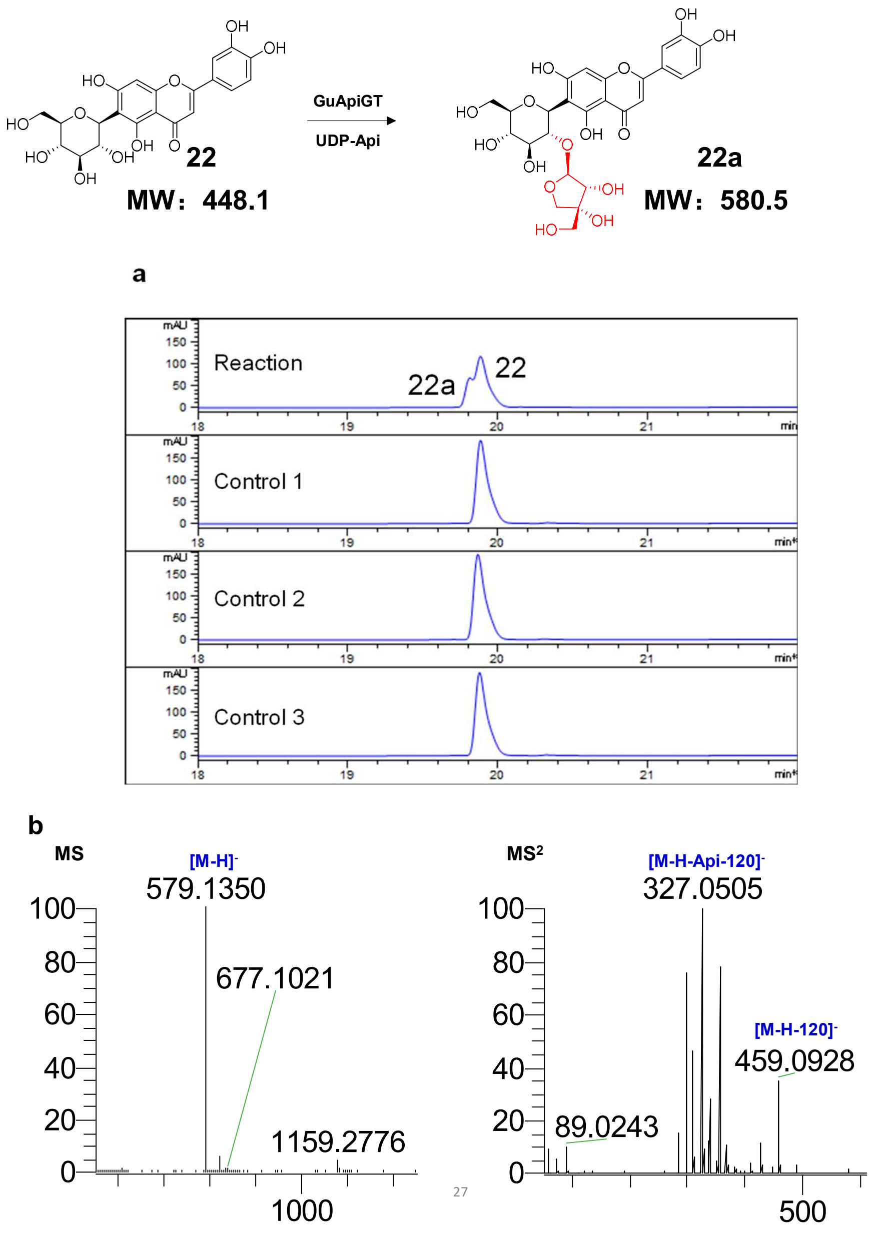

Supplementary Fig. 27 HPLC and LC/MS analyses of GuApiGT catalytic reaction mixture for substrate **22**. **a**, HPLC analysis of GuApiGT catalyzed product using **22** as the substrate. **b**, (-)-ESI-MS and MS^2 spectra of product **22a**. UDP-Api was produced by adding UDP-GlcA, purified UAXS and NAD^+ to the mixed system. Control 1, UDP-GlcA-free. Control 2, UAXS-free. Control 3, UDP-Xyl to replace UDP-Api supply system. The analysis conditions are given in **Supplementary Table 3**.

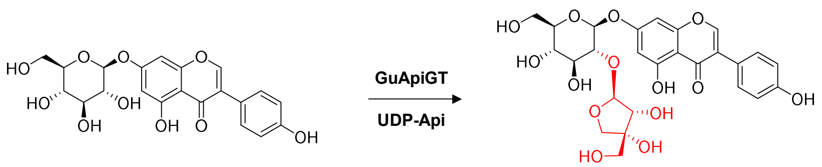

23
MW：432.4

23a
MW：564.5

a

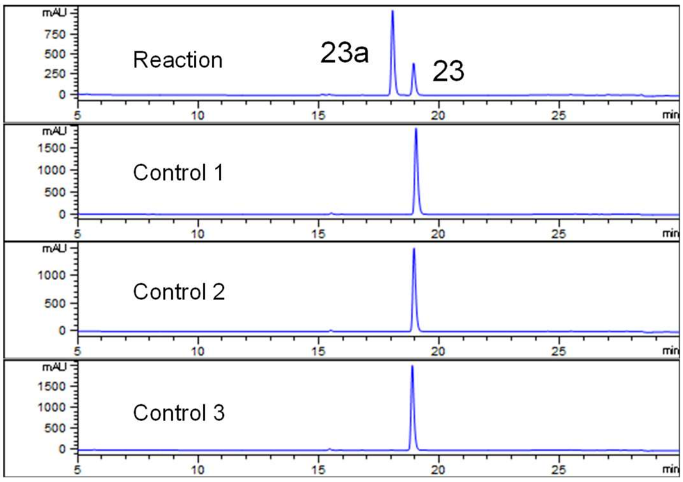

b

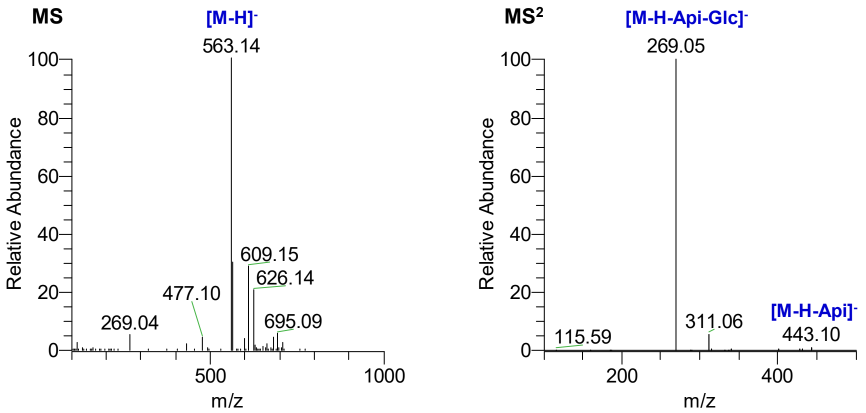

Supplementary Fig. 28 HPLC and LC/MS analyses of GuApiGT catalytic reaction mixture for substrate **23**. **a**, HPLC analysis of GuApiGT catalyzed product using **23** as the substrate. **b**, (-)-ESI-MS and MS^2 spectra of product **23a**. UDP-Api was produced by adding UDP-GlcA, purified UAXS and NAD^+ to the mixed system. Control 1, UDP-GlcA-free. Control 2, UAXS-free. Control 3, UDP-Xyl to replace UDP-Api supply system. The analysis conditions are given in **Supplementary Table 3**.

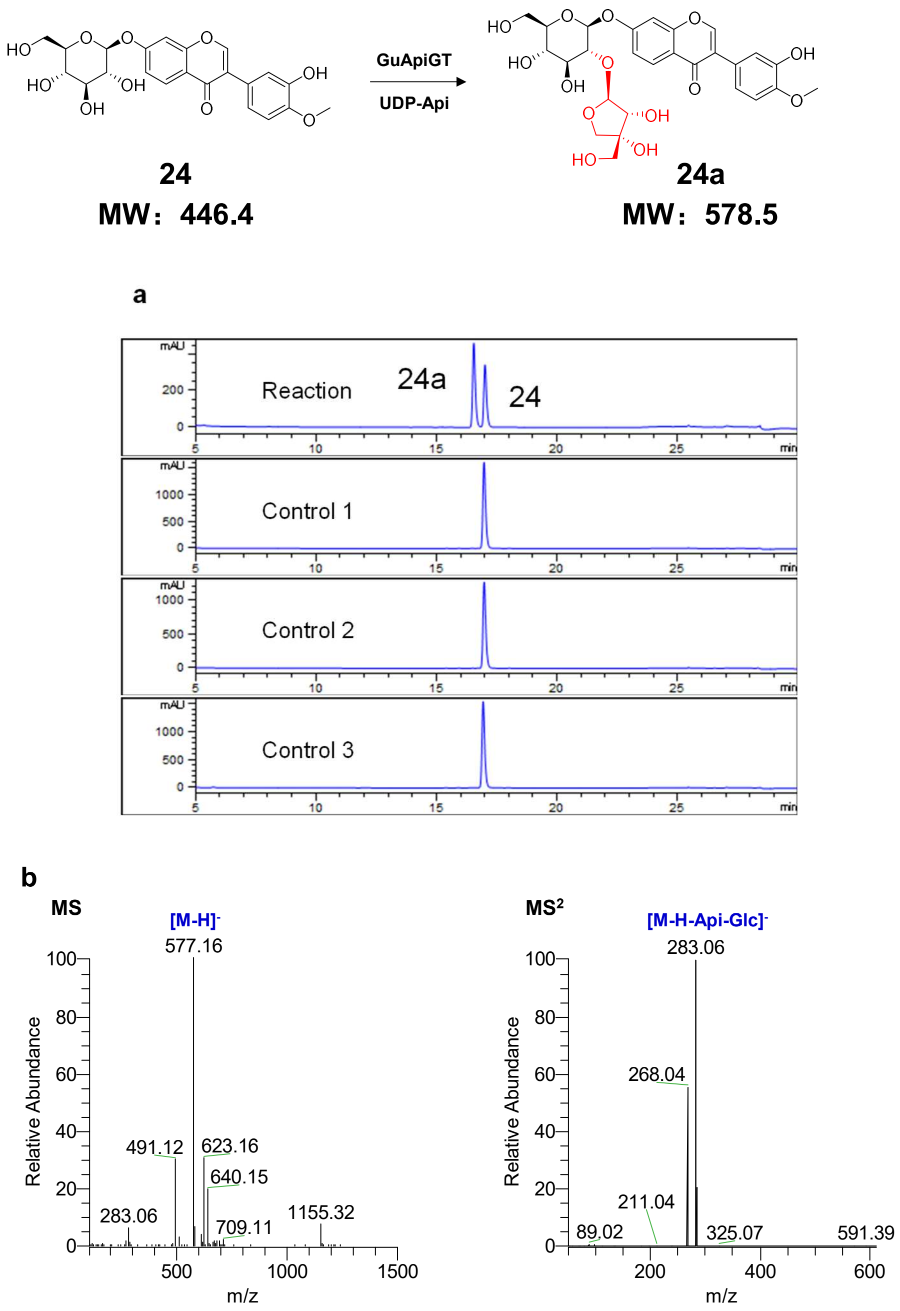

Supplementary Fig. 29 HPLC and LC/MS analyses of GuApiGT catalytic reaction mixture for substrate **24**. **a**, HPLC analysis of GuApiGT catalyzed product using **24** as the substrate. **b**, (-)-ESI-MS and MS^2 spectra of product **24a**. UDP-Api was produced by adding UDP-GlcA, purified UAXS and NAD^+ to the mixed system. Control 1, UDP-GlcA-free. Control 2, UAXS-free. Control 3, UDP-Xyl to replace UDP-Api supply system. The analysis conditions are given in **Supplementary Table 3**.

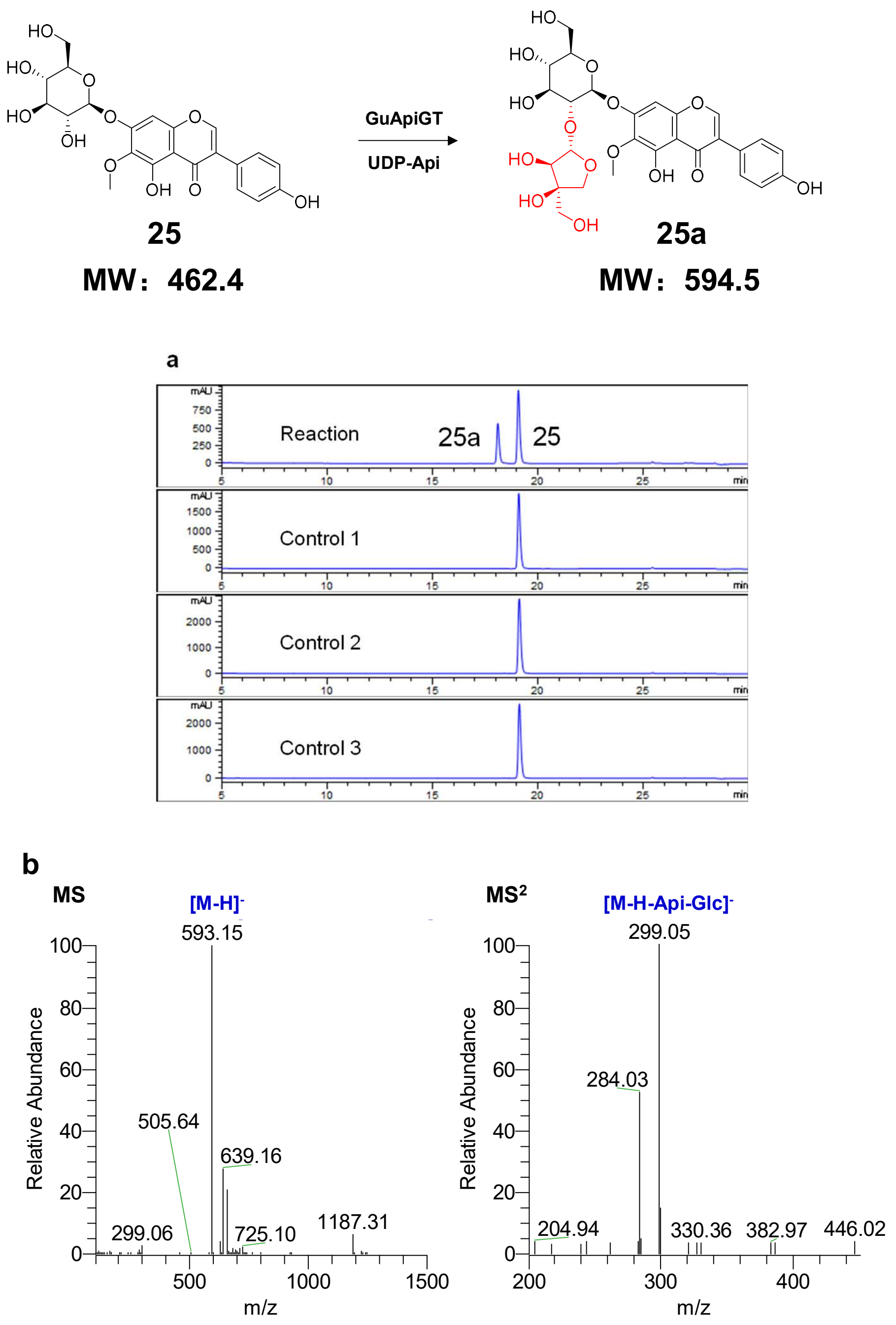

Supplementary Fig. 30 HPLC and LC/MS analyses of GuApiGT catalytic reaction mixture for substrate **25**. **a**, HPLC analysis of GuApiGT catalyzed product using **25** as the substrate. **b**, (-)-ESI-MS and MS^2 spectra of product **25a**. UDP-Api was produced by adding UDP-GlcA, purified UAXS and NAD^+ to the mixed system. Control 1, UDP-GlcA-free. Control 2, UAXS-free. Control 3, UDP-Xyl to replace UDP-Api supply system. The analysis conditions are given in **Supplementary Table 3**.

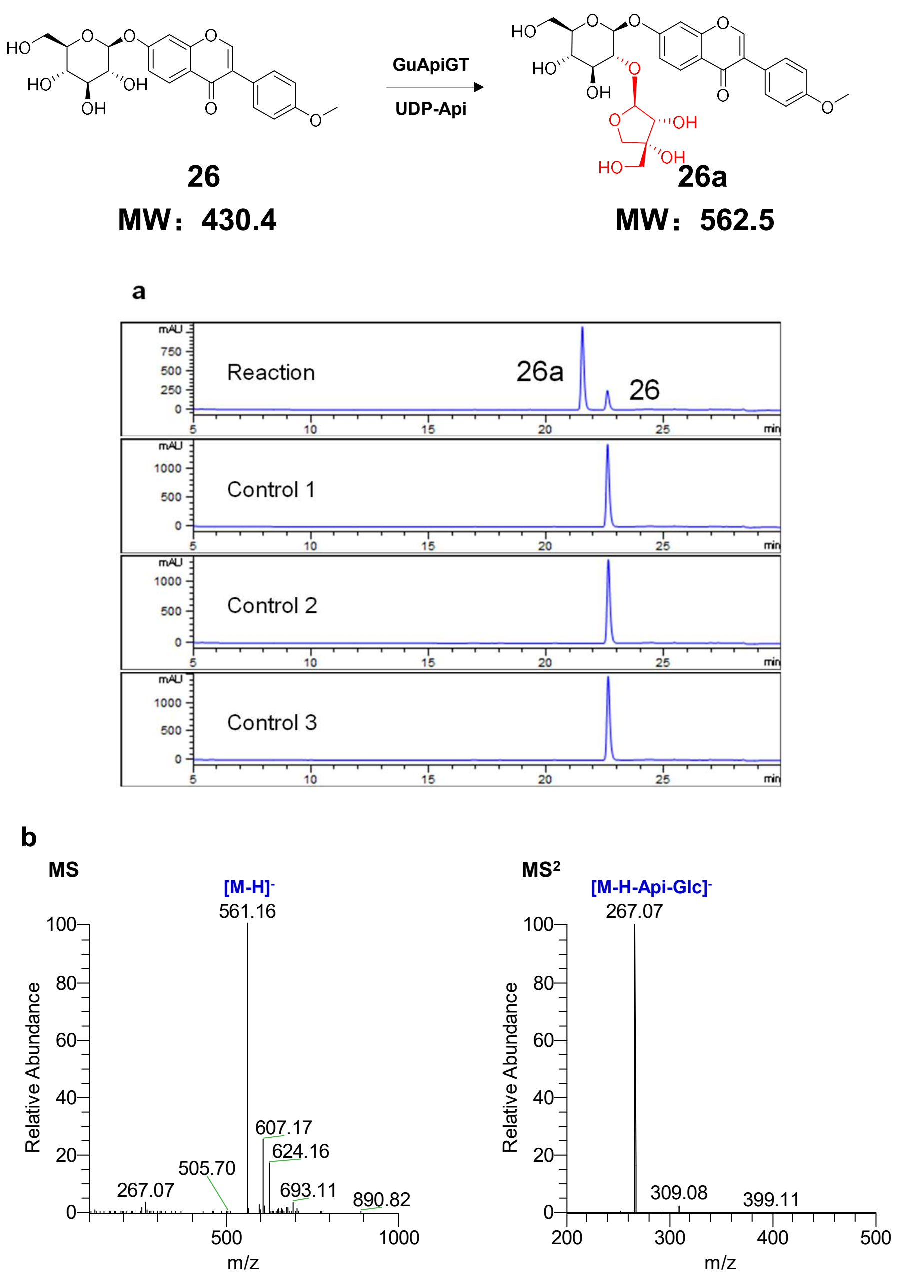

Supplementary Fig. 31 HPLC and LC/MS analyses of GuApiGT catalytic reaction mixture for substrate **26**. **a**, HPLC analysis of GuApiGT catalyzed product using **26** as the substrate. **b**, (-)-ESI-MS and MS^2 spectra of product **26a**. UDP-Api was produced by adding UDP-GlcA, purified UAXS and NAD^+ to the mixed system. Control 1, UDP-GlcA-free. Control 2, UAXS-free. Control 3, UDP-Xyl to replace UDP-Api supply system. The analysis conditions are given in **Supplementary Table 3**.

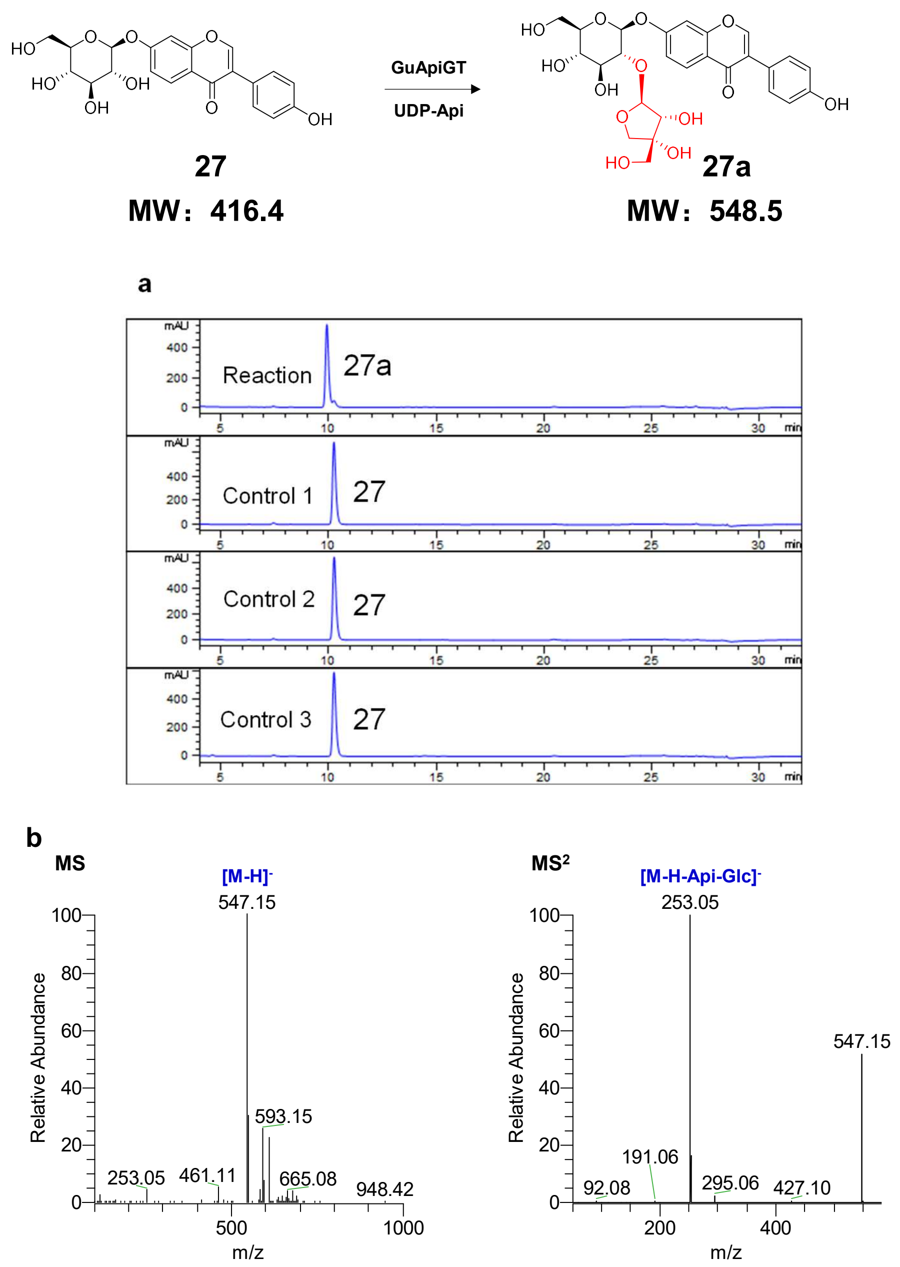

Supplementary Fig. 32 HPLC and LC/MS analyses of GuApiGT catalytic reaction mixture for substrate **27**. **a**, HPLC analysis of GuApiGT catalyzed product using **27** as the substrate. **b**, (-)-ESI-MS and MS^2 spectra of product **27a**. UDP-Api was produced by adding UDP-GlcA, purified UAXS and NAD^+ to the mixed system. Control 1, UDP-GlcA-free. Control 2, UAXS-free. Control 3, UDP-Xyl to replace UDP-Api supply system. The analysis conditions are given in **Supplementary Table 3**.

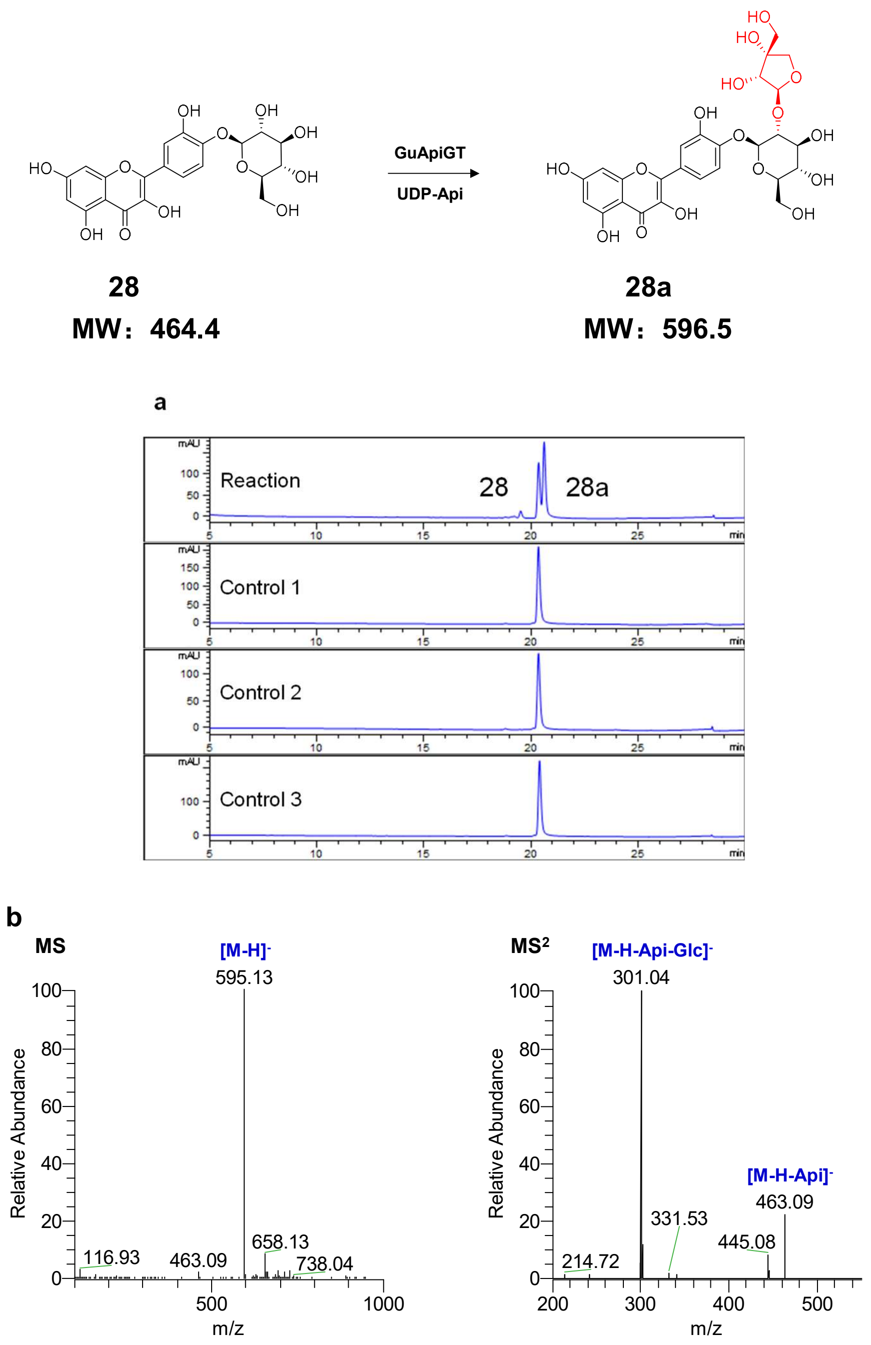

Supplementary Fig. 33 HPLC and LC/MS analyses of GuApiGT catalytic reaction mixture for substrate **28**. **a**, HPLC analysis of GuApiGT catalyzed product using **28** as the substrate. **b**, (-)-ESI-MS and MS^2 spectra of product **28a**. UDP-Api was produced by adding UDP-GlcA, purified UAXS and NAD^+ to the mixed system. Control 1, UDP-GlcA-free. Control 2, UAXS-free. Control 3, UDP-Xyl to replace UDP-Api supply system. The analysis conditions are given in **Supplementary Table 3**.

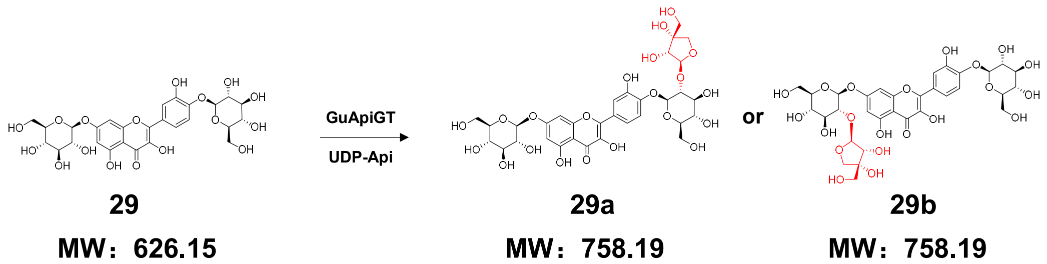

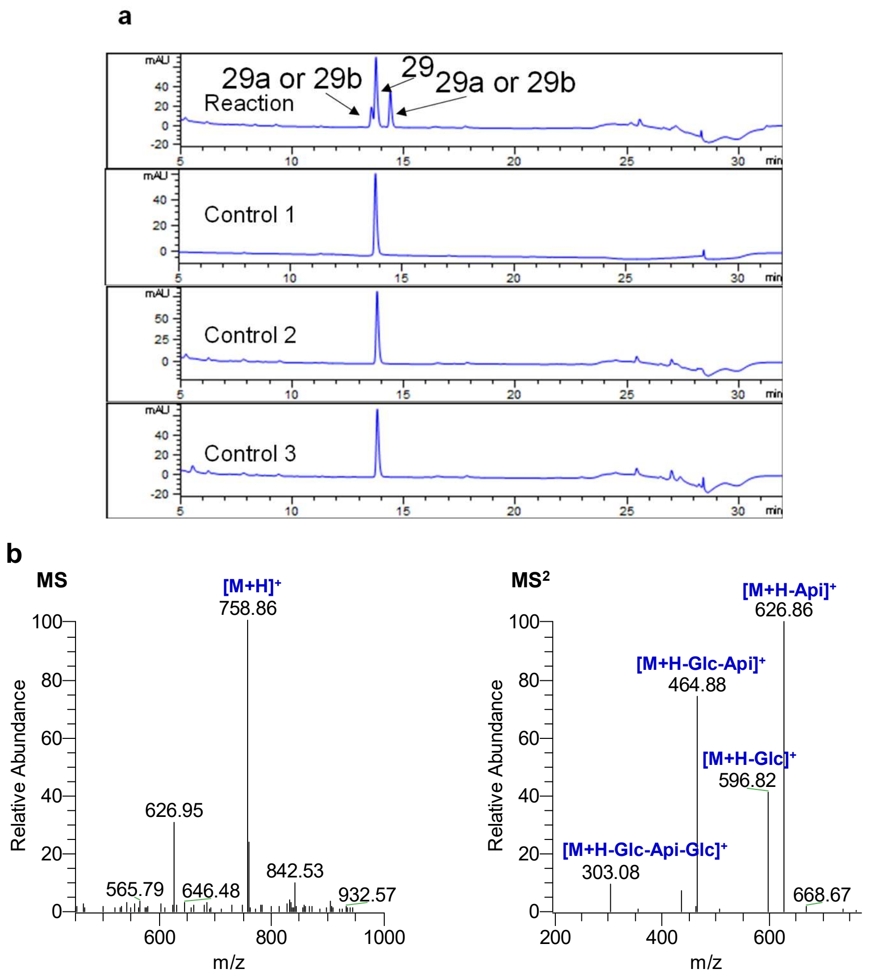

Supplementary Fig. 34 HPLC and LC/MS analyses of GuApiGT catalytic reaction mixture for substrate **29**. **a**, HPLC analysis of GuApiGT catalyzed product using **29** as the substrate. **b**, (+)-ESI-MS and MS^2 spectra of product **29a** or **29b**. UDP-Api was produced by adding UDP-GlcA, purified UAXS and NAD^+ to the mixed system. Control 1, UDP-GlcA-free. Control 2, UAXS-free. Control 3, UDP-Xyl to replace UDP-Api supply system. The analysis conditions are given in **Supplementary Table 3**.

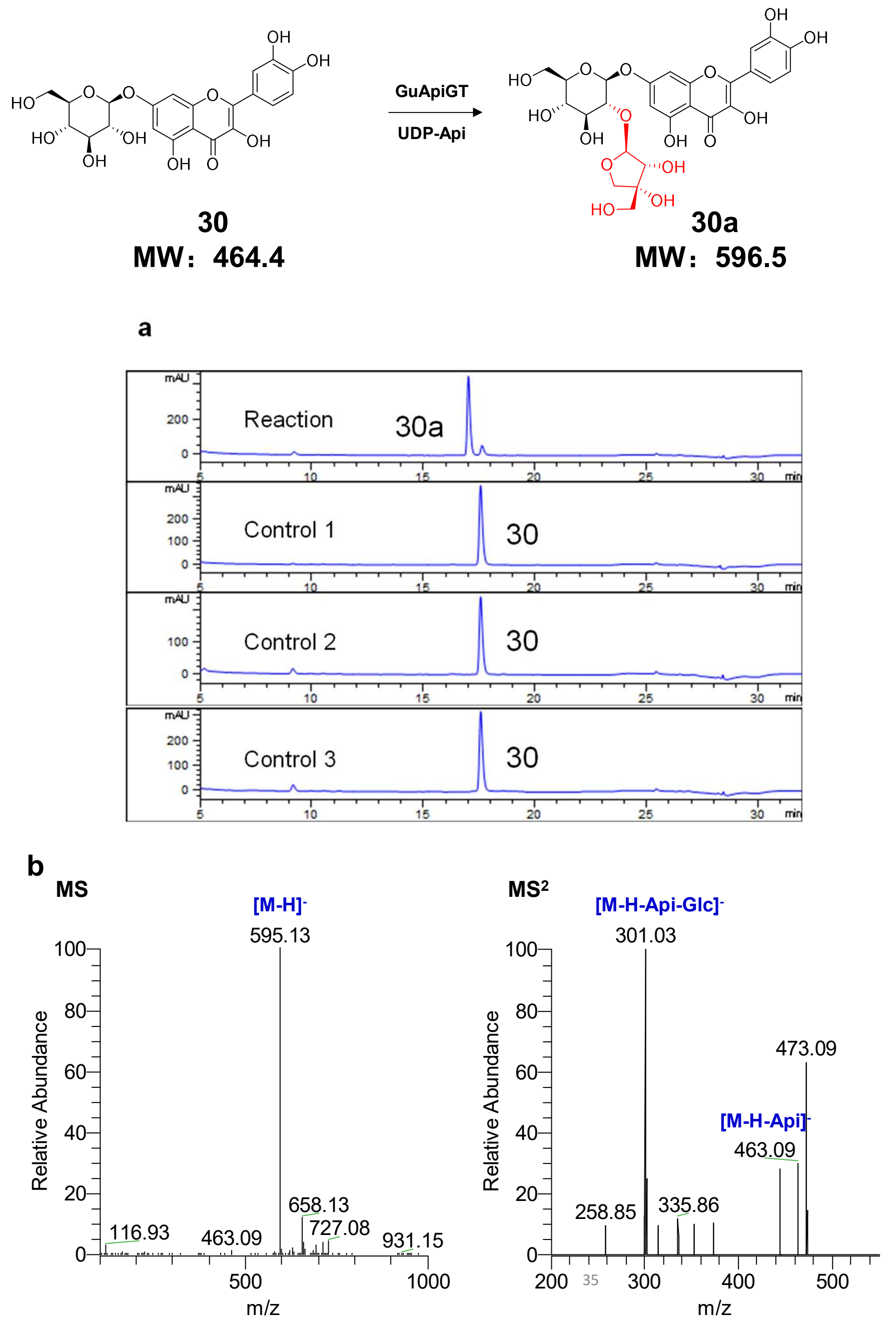

Supplementary Fig. 35 HPLC and LC/MS analyses of GuApiGT catalytic reaction mixture for substrate **30**. **a**, HPLC analysis of GuApiGT catalyzed product using **30** as the substrate. **b**, (-)-ESI-MS and MS^2 spectra of product **30a**. UDP-Api was produced by adding UDP-GlcA, purified UAXS and NAD^+ to the mixed system. Control 1, UDP-GlcA-free. Control 2, UAXS-free. Control 3, UDP-Xyl to replace UDP-Api supply system. The analysis conditions are given in **Supplementary Table 3**.

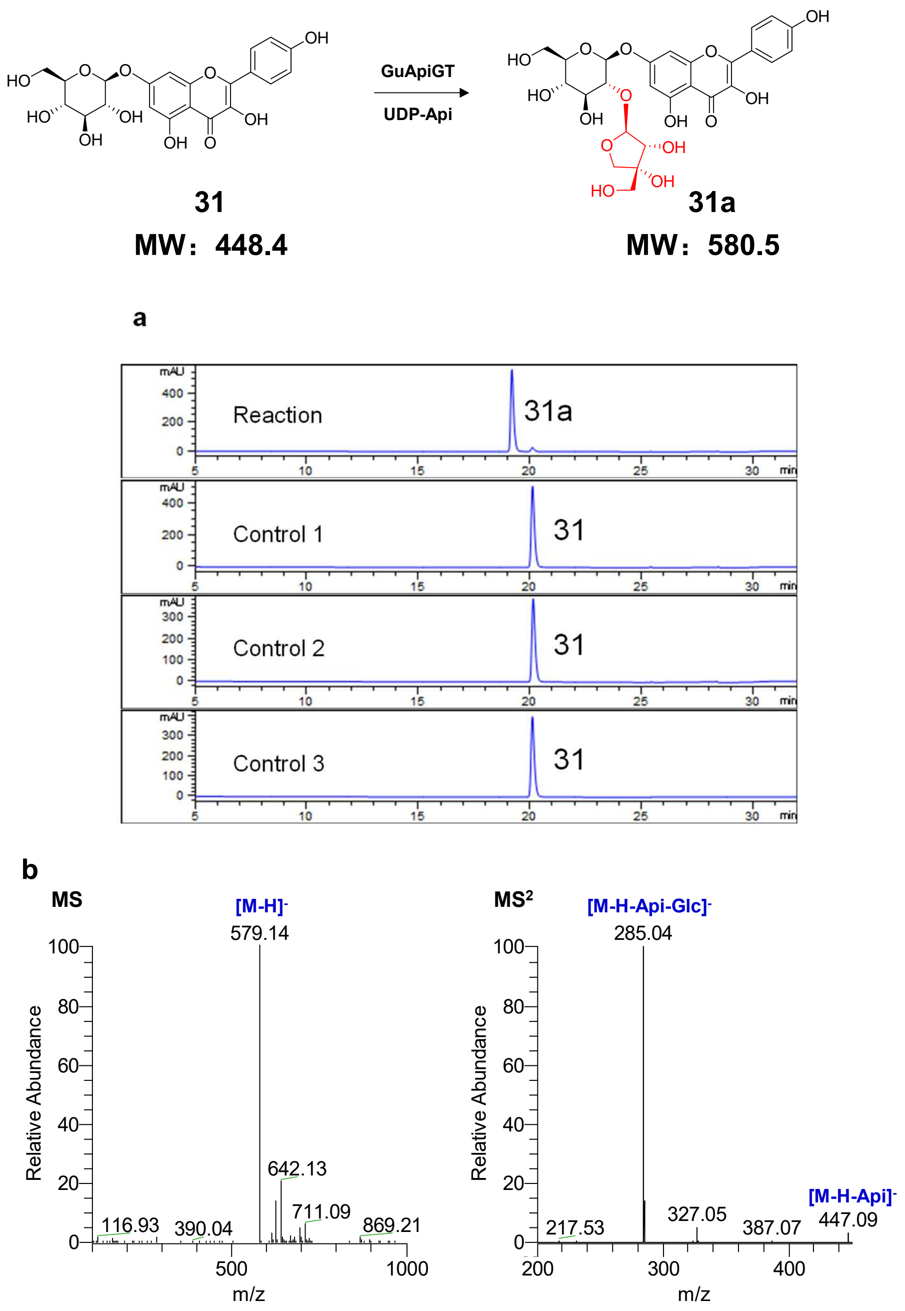

Supplementary Fig. 36 HPLC and LC/MS analyses of GuApiGT catalytic reaction mixture for substrate **31**. **a**, HPLC analysis of GuApiGT catalyzed product using **31** as the substrate. **b**, (-)-ESI-MS and MS^2 spectra of product **31a**. UDP-Api was produced by adding UDP-GlcA, purified UAXS and NAD$^+$ to the mixed system. Control 1, UDP-GlcA-free. Control 2, UAXS-free. Control 3, UDP-Xyl to replace UDP-Api supply system. The analysis conditions are given in **Supplementary Table 3**.

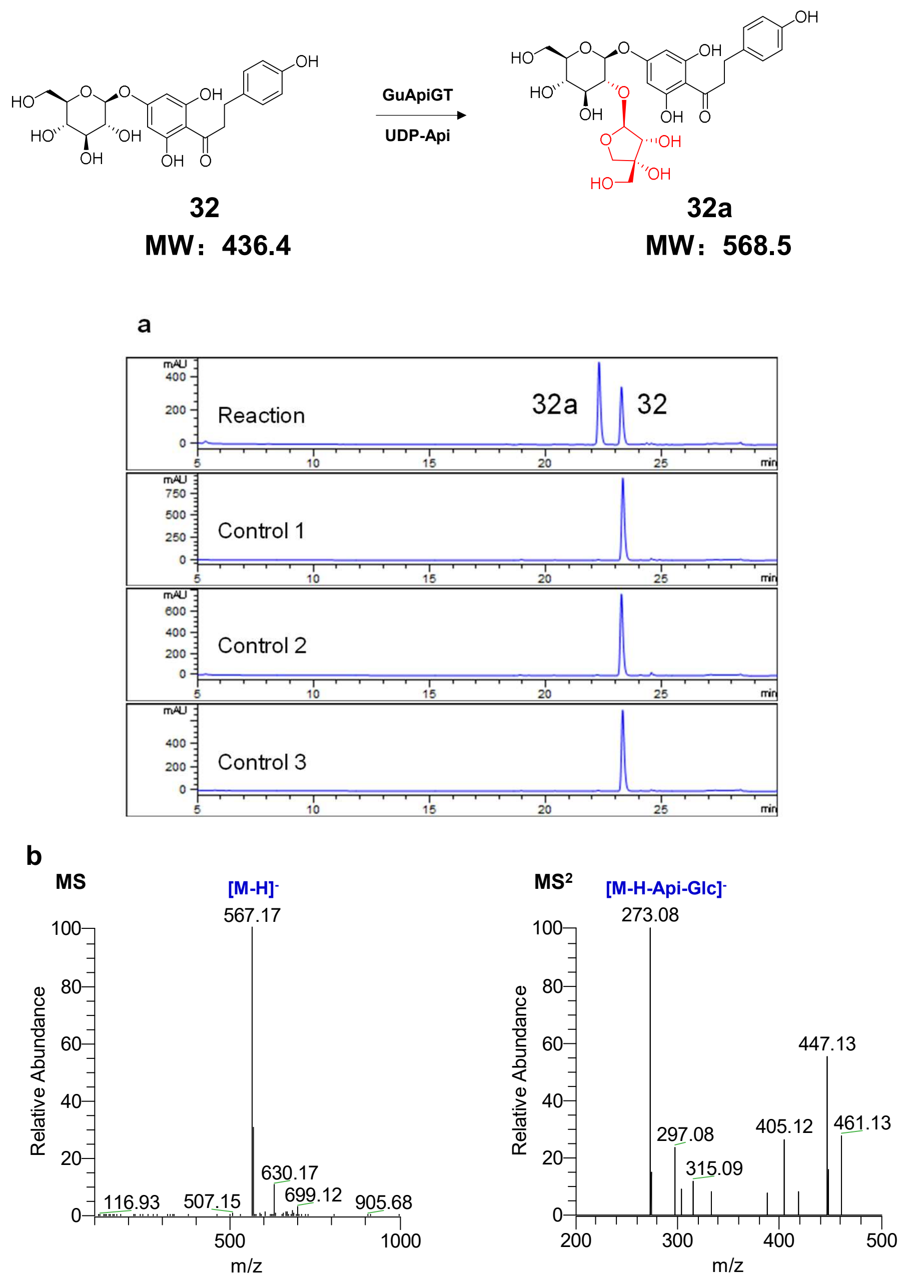

Supplementary Fig. 37 HPLC and LC/MS analyses of GuApiGT catalytic reaction mixture for substrate **32**. **a**, HPLC analysis of GuApiGT catalyzed product using **32** as the substrate. **b**, (-)-ESI-MS and MS^2 spectra of product **32a**. UDP-Api was produced by adding UDP-GlcA, purified UAXS and NAD$^+$ to the mixed system. Control 1, UDP-GlcA-free. Control 2, UAXS-free. Control 3, UDP-Xyl to replace UDP-Api supply system. The analysis conditions are given in **Supplementary Table 3**.

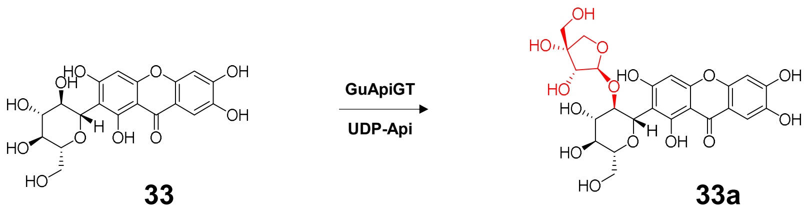

33
MW：422.3

33a
MW：554.5

a

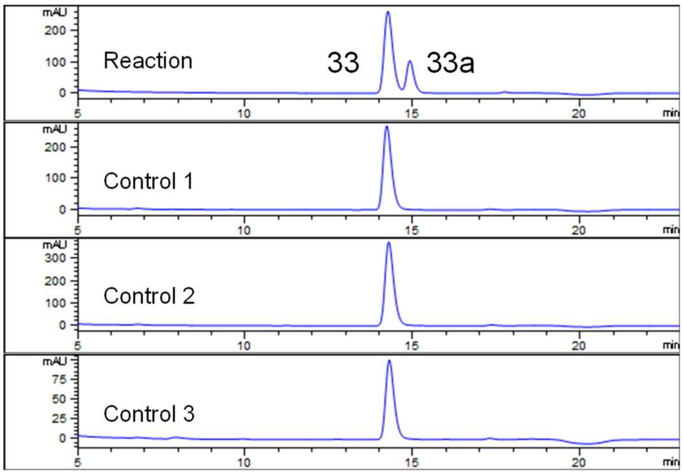

b

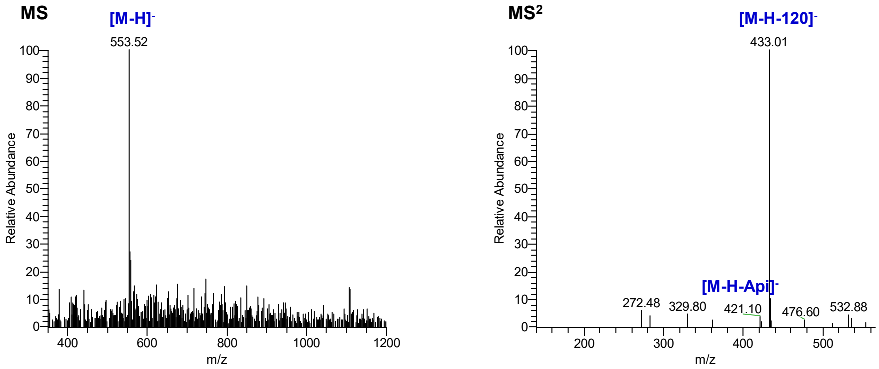

Supplementary Fig. 38 HPLC and LC/MS analyses of GuApiGT catalytic reaction mixture for substrate **33**. **a**, HPLC analysis of GuApiGT catalyzed product using **33** as the substrate. **b**, (-)-ESI-MS and MS^2 spectra of product **33a**. UDP-Api was produced by adding UDP-GlcA, purified UAXS and NAD^+ to the mixed system. Control 1, UDP-GlcA-free. Control 2, UAXS-free. Control 3, UDP-Xyl to replace UDP-Api supply system. The analysis conditions are given in **Supplementary Table 3**.

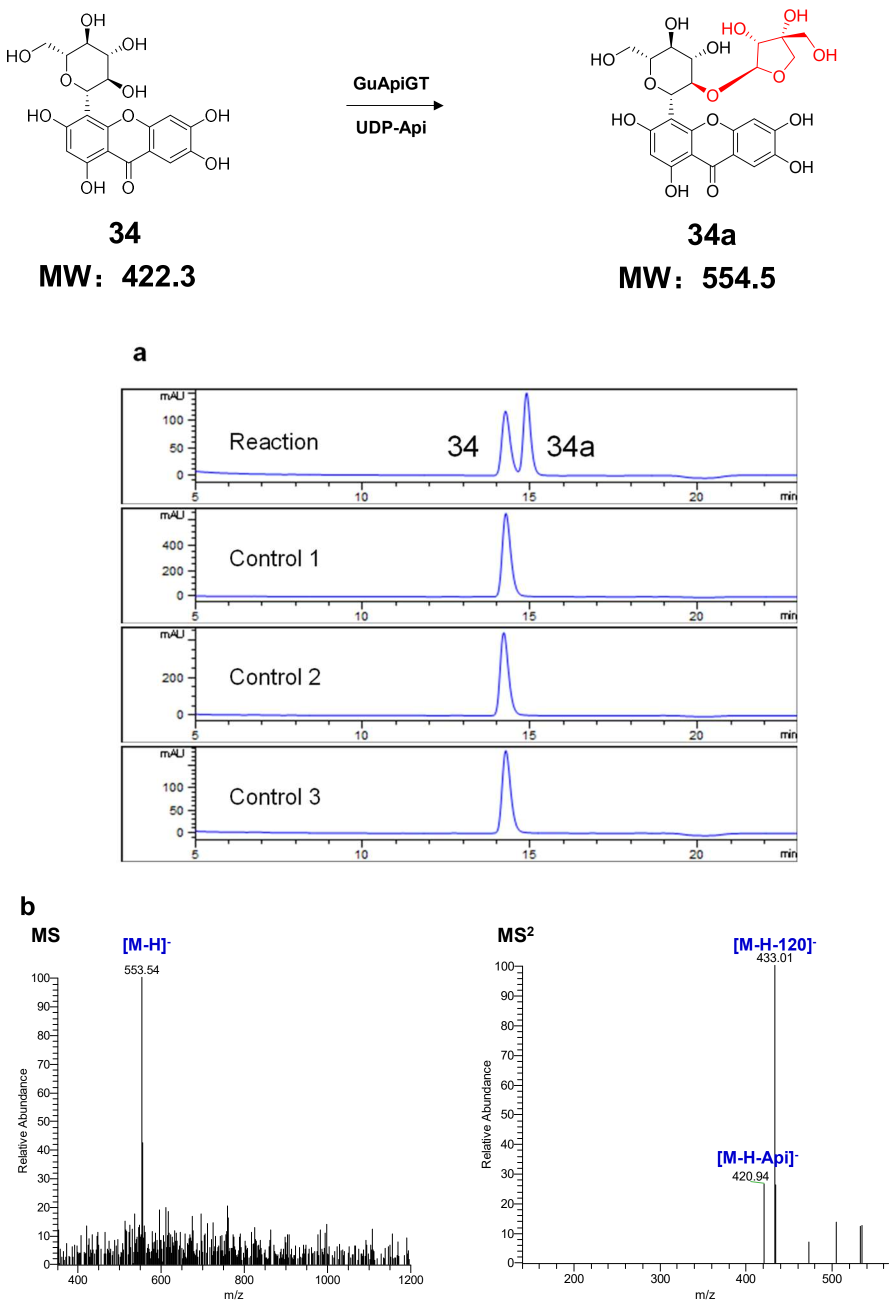

Supplementary Fig. 39 HPLC and LC/MS analyses of GuApiGT catalytic reaction mixture for substrate **34**. **a**, HPLC analysis of GuApiGT catalyzed product using **34** as the substrate. **b**, (-)-ESI-MS and MS^2 spectra of product **34a**. UDP-Api was produced by adding UDP-GlcA, purified UAXS and NAD^+ to the mixed system. Control 1, UDP-GlcA-free. Control 2, UAXS-free. Control 3, UDP-Xyl to replace UDP-Api supply system. The analysis conditions are given in **Supplementary Table 3**.

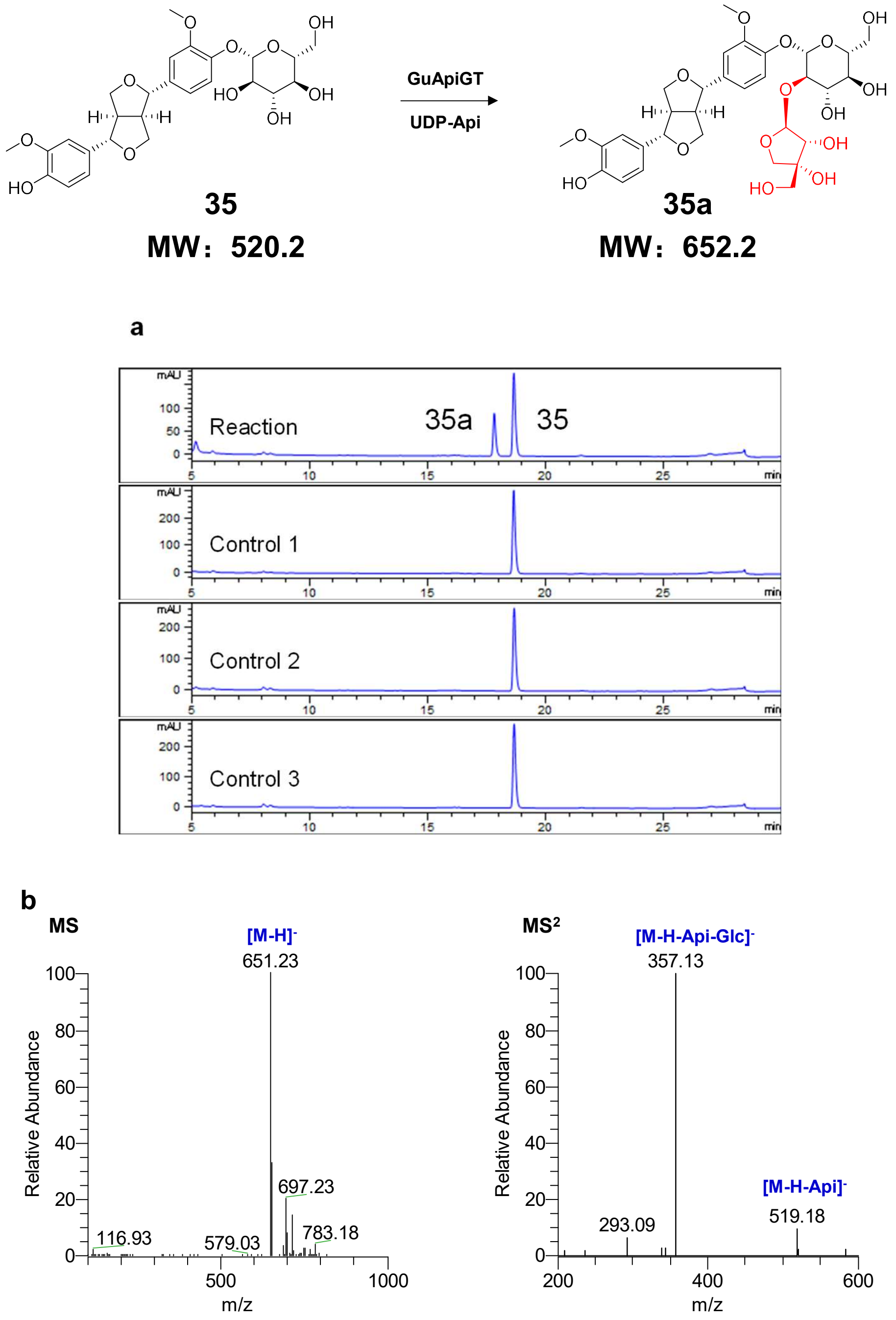

Supplementary Fig. 40 HPLC and LC/MS analyses of GuApiGT catalytic reaction mixture for substrate **35**. **a**, HPLC analysis of GuApiGT catalyzed product using **35** as the substrate. **b**, (-)-ESI-MS and MS^2 spectra of product **35a**. UDP-Api was produced by adding UDP-GlcA, purified UAXS and NAD$^+$ to the mixed system. Control 1, UDP-GlcA-free. Control 2, UAXS-free. Control 3, UDP-Xyl to replace UDP-Api supply system. The analysis conditions are given in **Supplementary Table 3**.

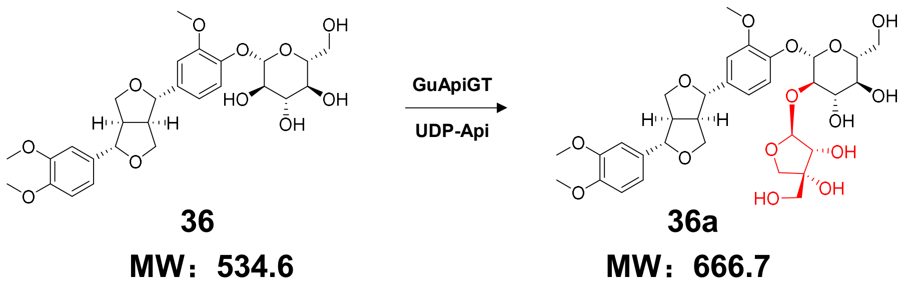

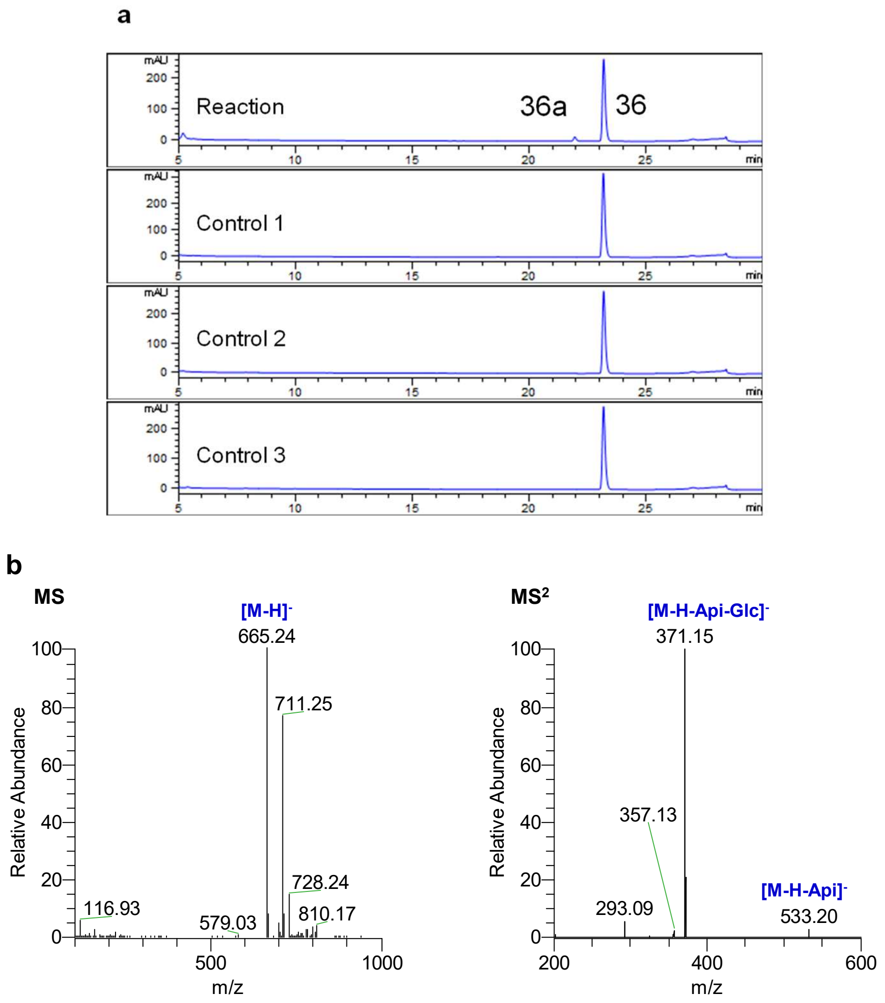

Supplementary Fig. 41 HPLC and LC/MS analyses of GuApiGT catalytic reaction mixture for substrate **36**. **a**, HPLC analysis of GuApiGT catalyzed product using **36** as the substrate. **b**, (-)-ESI-MS and MS^2 spectra of product **36a**. UDP-Api was produced by adding UDP-GlcA, purified UAXS and NAD^+ to the mixed system. Control 1, UDP-GlcA-free. Control 2, UAXS-free. Control 3, UDP-Xyl to replace UDP-Api supply system. The analysis conditions are given in **Supplementary Table 3**.

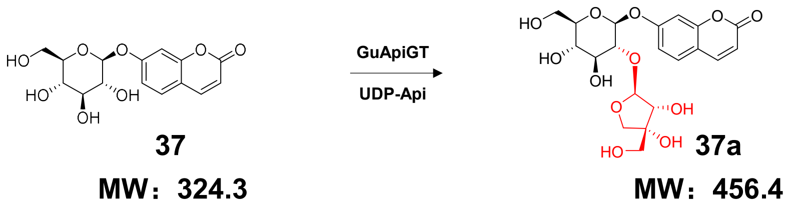

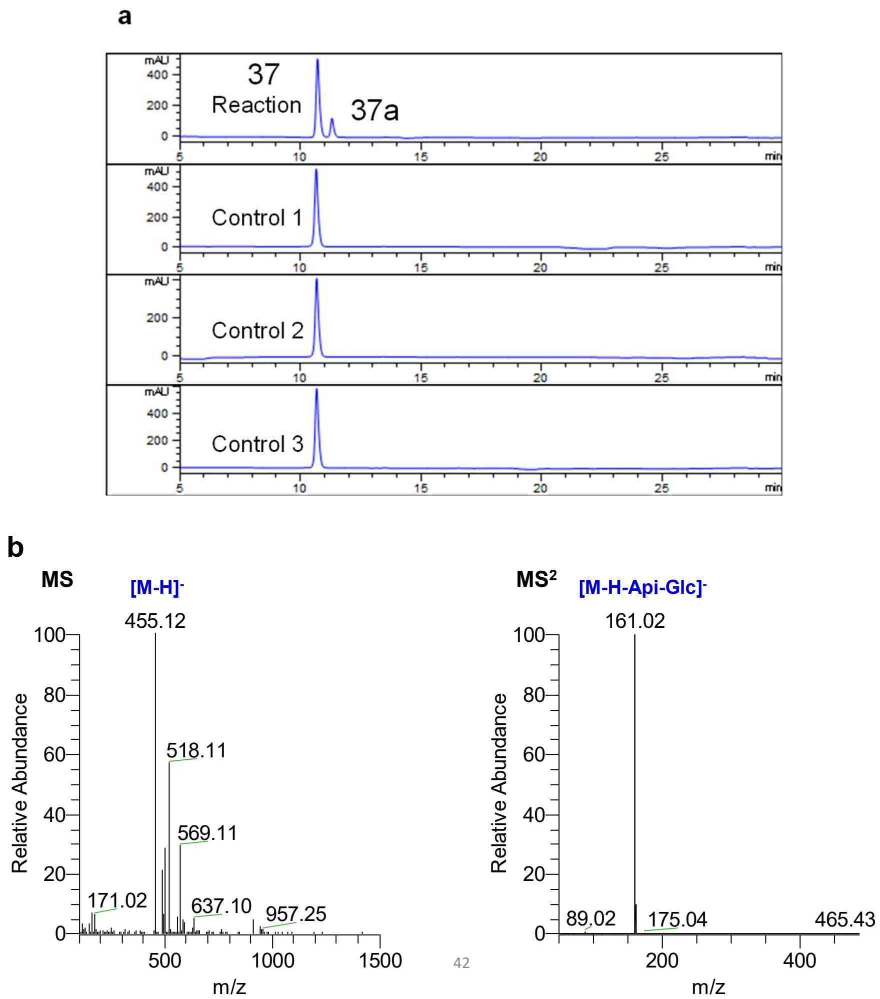

Supplementary Fig. 42 HPLC and LC/MS analyses of GuApiGT catalytic reaction mixture for substrate **37**. **a**, HPLC analysis of GuApiGT catalyzed product using **37** as the substrate. **b**, (-)-ESI-MS and MS^2 spectra of product **37a**. UDP-Api was produced by adding UDP-GlcA, purified UAXS and NAD^+ to the mixed system. Control 1, UDP-GlcA-free. Control 2, UAXS-free. Control 3, UDP-Xyl to replace UDP-Api supply system. The analysis conditions are given in **Supplementary Table 3**.

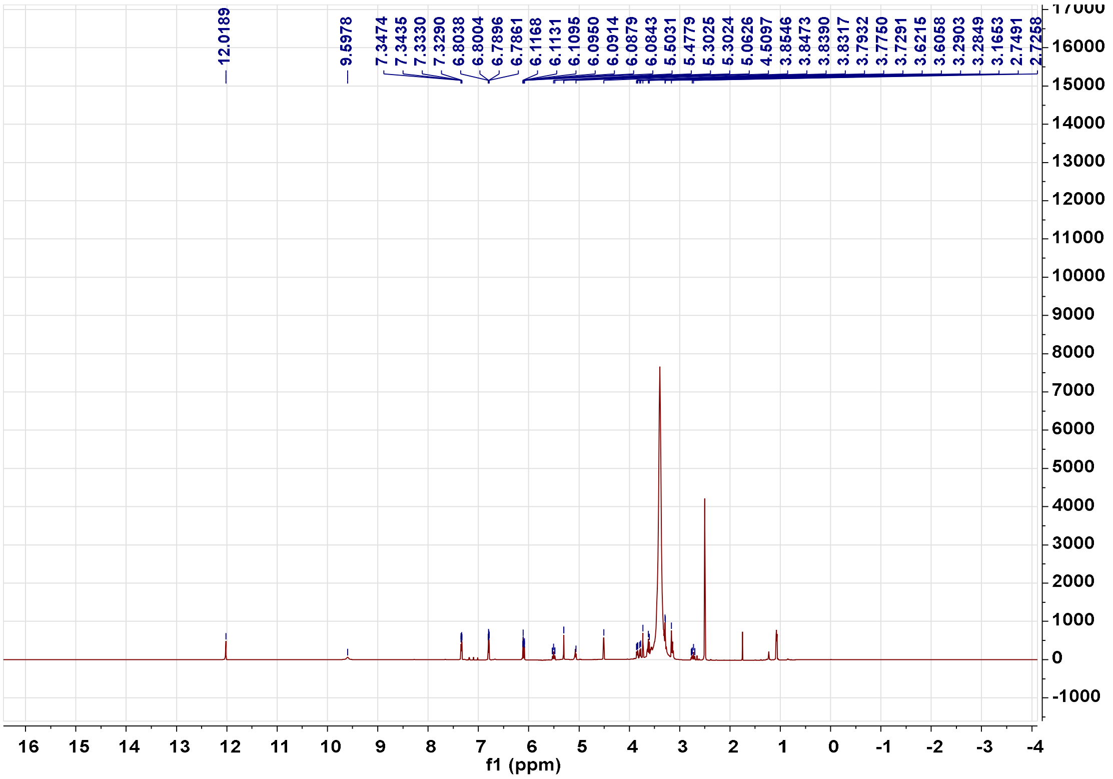

Supplementary Fig. 43 The ^{1}H NMR spectrum of **6a** in DMSO-d_6 (600 MHz).

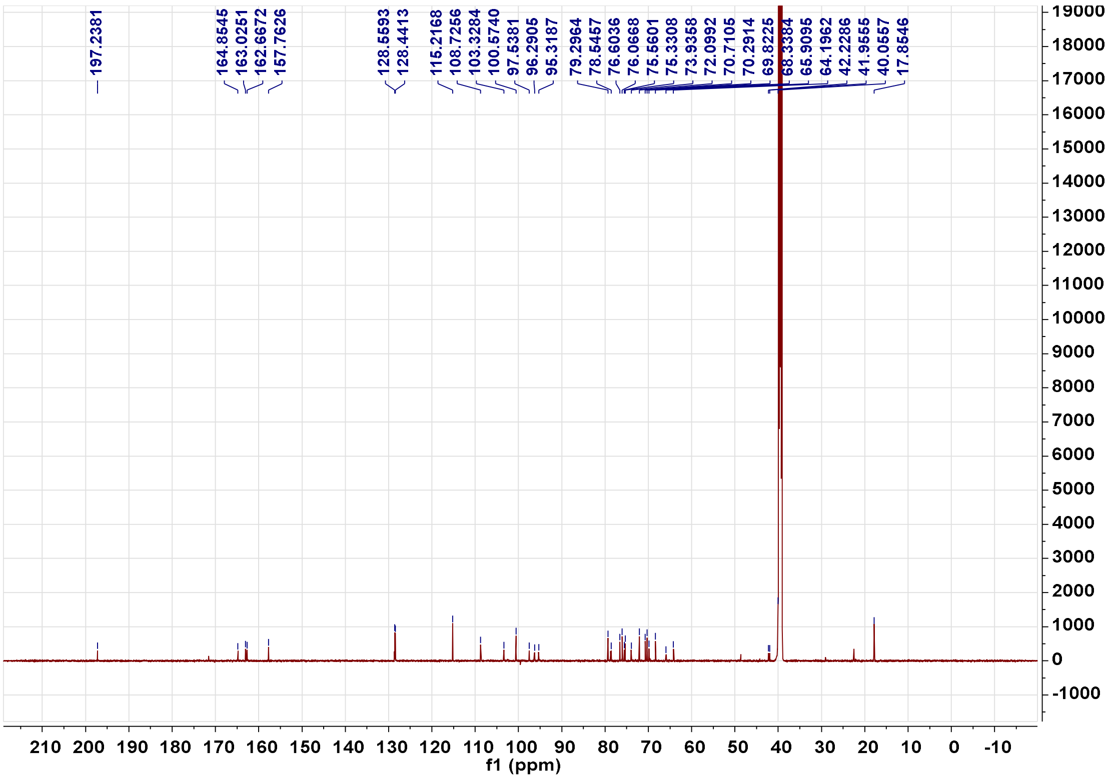

Supplementary Fig. 44 The ^{13}C NMR spectrum of **6a** in DMSO-d_6 (150 MHz).

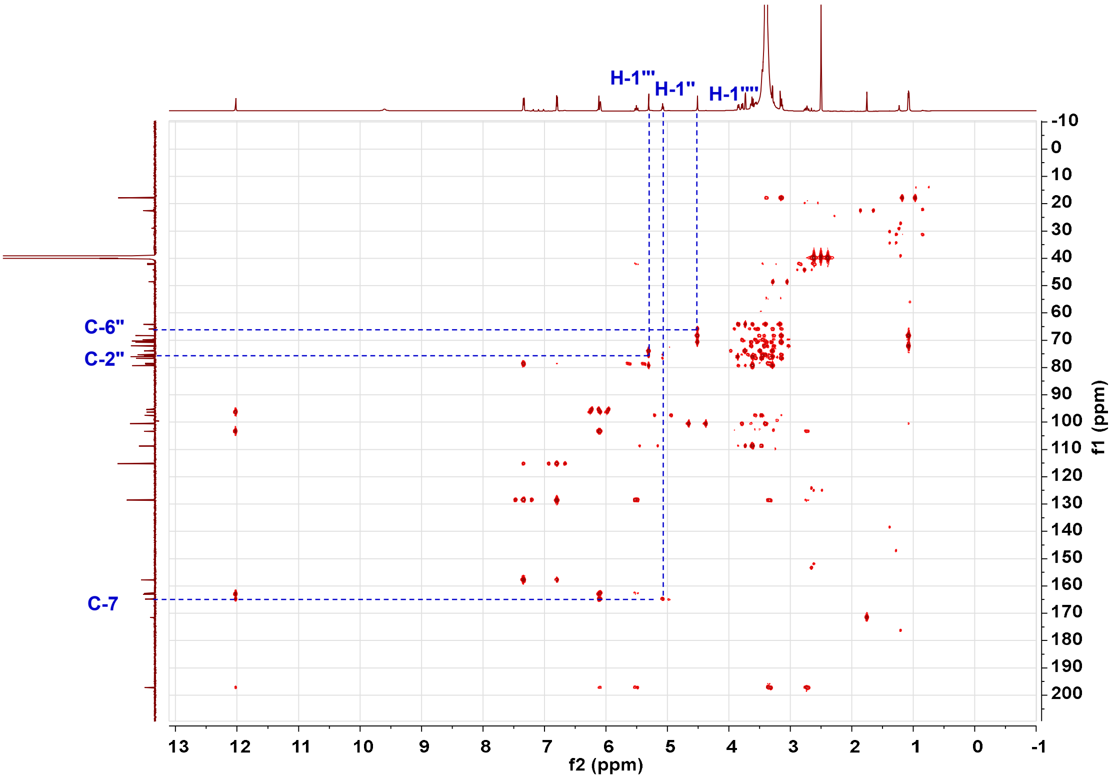

Supplementary Fig. 45 The HMBC spectrum of **6a** in DMSO-d_6 (600 MHz).

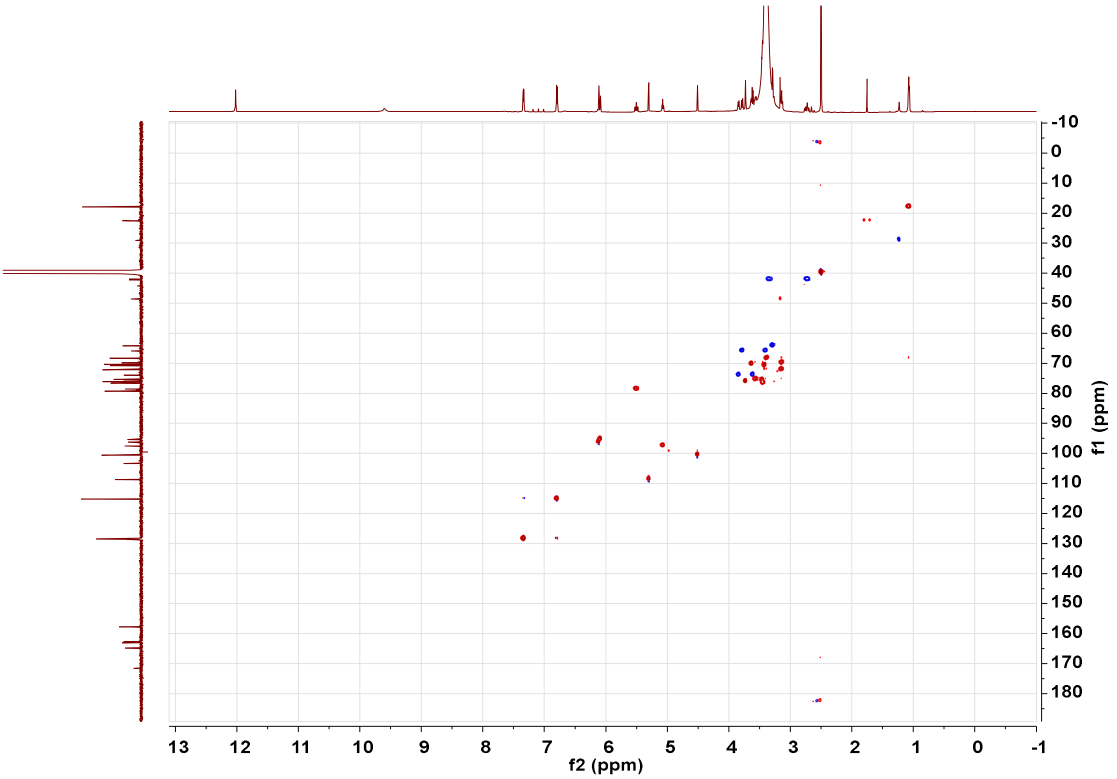

Supplementary Fig. 46 The HSQC spectrum of **6a** in DMSO-d_6 (600 MHz).

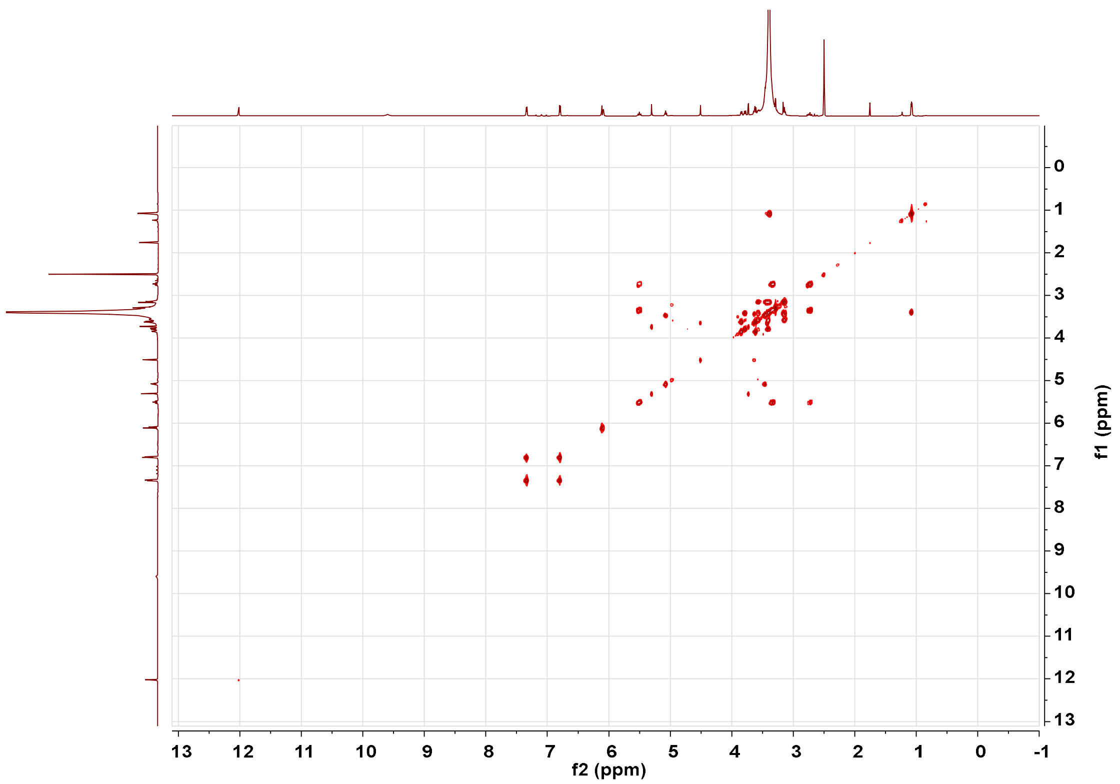

Supplementary Fig. 47 The ^{1}H-^{1}H COSY spectrum of **6a** in DMSO-d_6 (600 MHz).

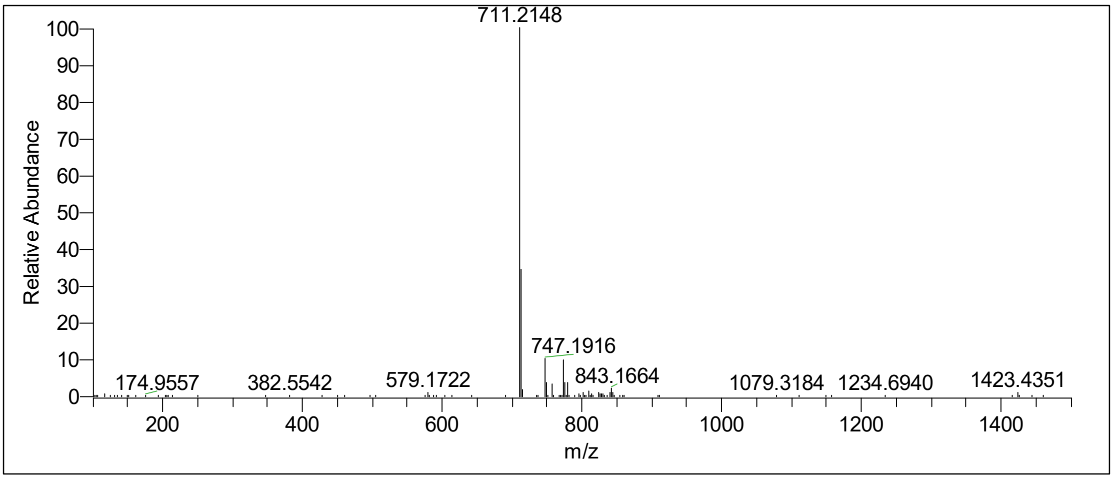

Supplementary Fig. 48 (-)-ESI-HRMS spectrum of **6a**.

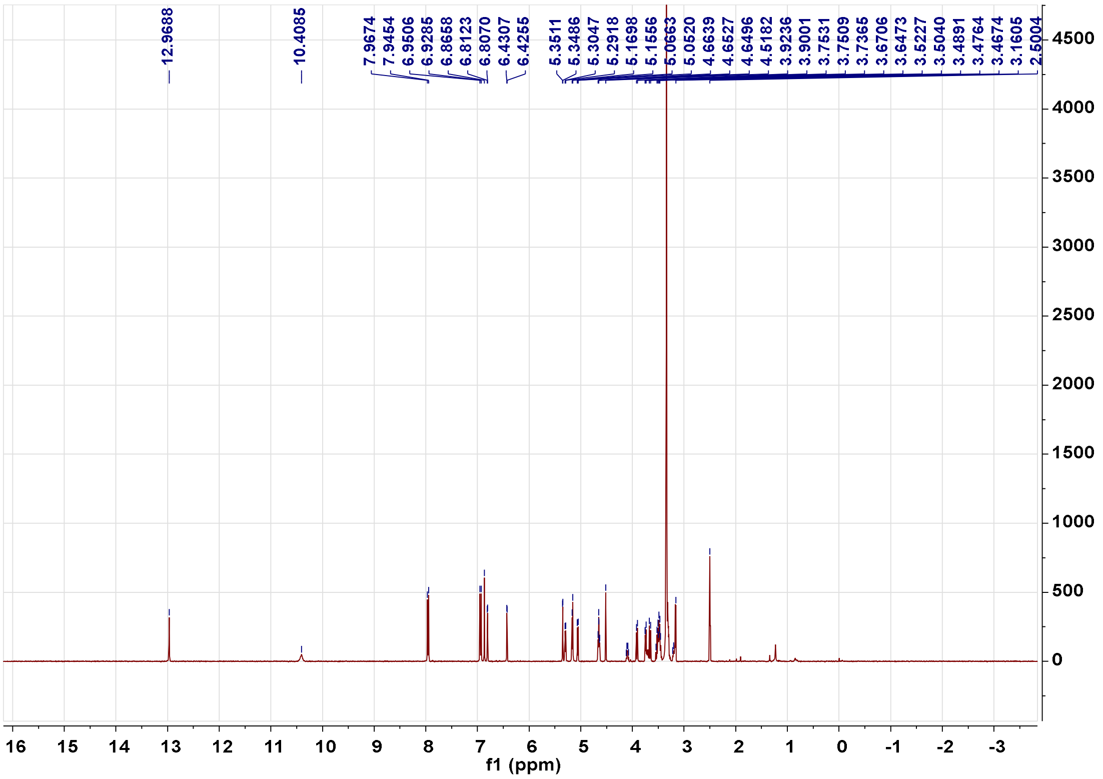

Supplementary Fig. 49 The ^{1}H NMR spectrum of **15a** in DMSO-d_6 (400 MHz).

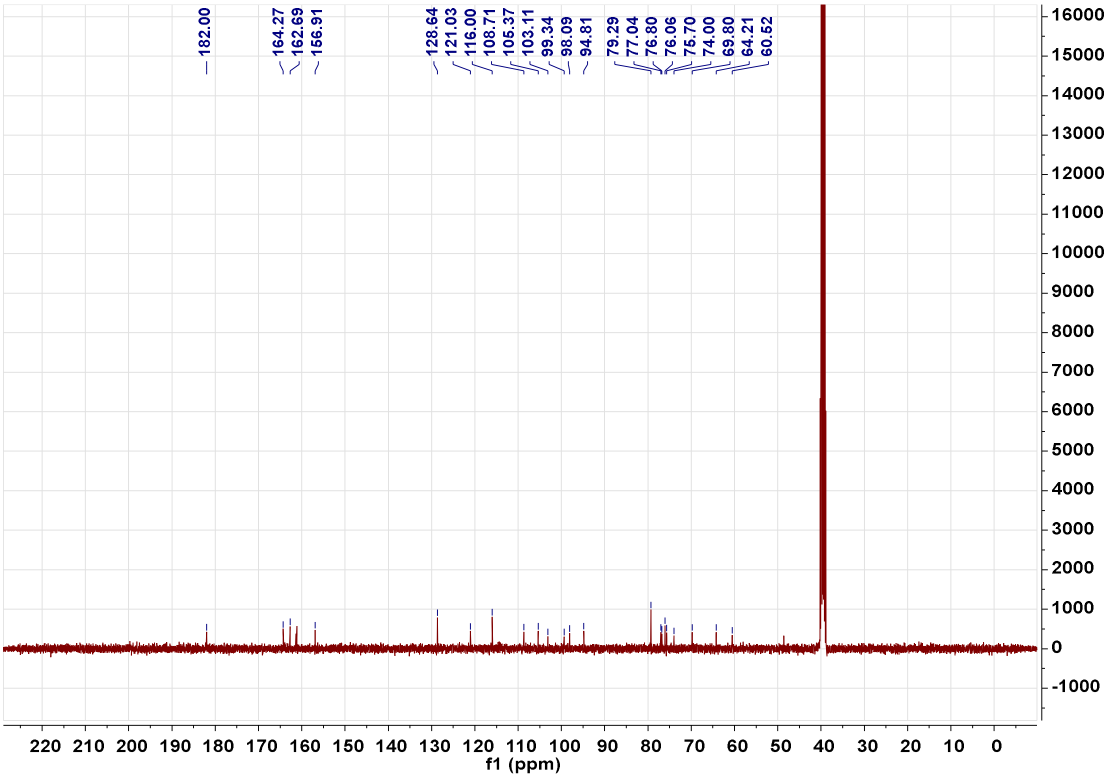

Supplementary Fig. 50 The ^{13}C NMR spectrum of **15a** in DMSO-d_6 (100 MHz).

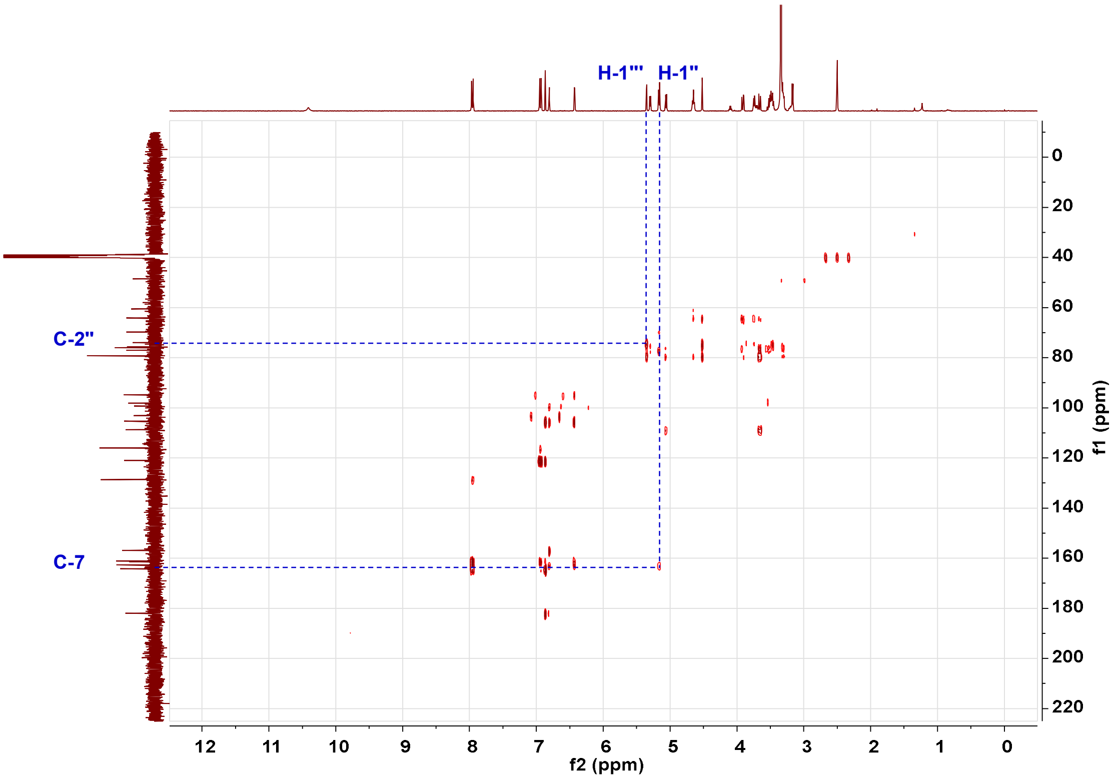

Supplementary Fig. 51 The HMBC spectrum of **15a** in DMSO-d_6 (400 MHz).

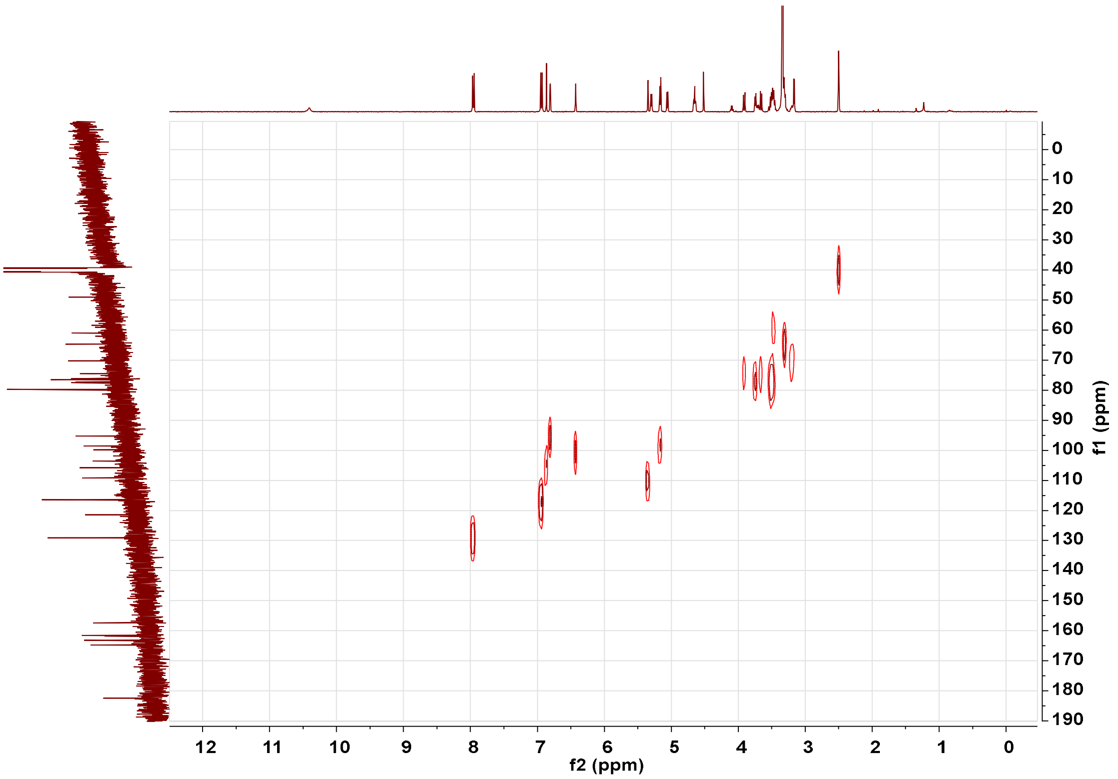

Supplementary Fig. 52 The HSQC spectrum of **15a** in DMSO-d_6 (400 MHz).

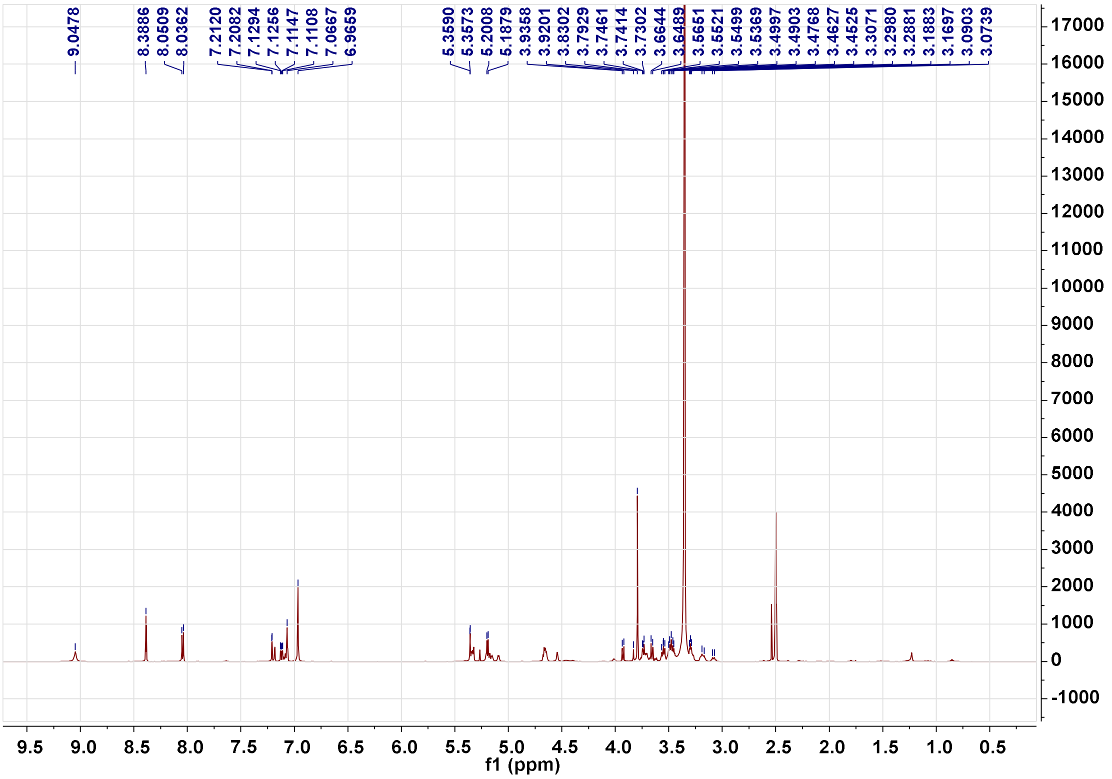

Supplementary Fig. 53 The ^{1}H NMR spectrum of **24a** in DMSO-d_6 (600 MHz).

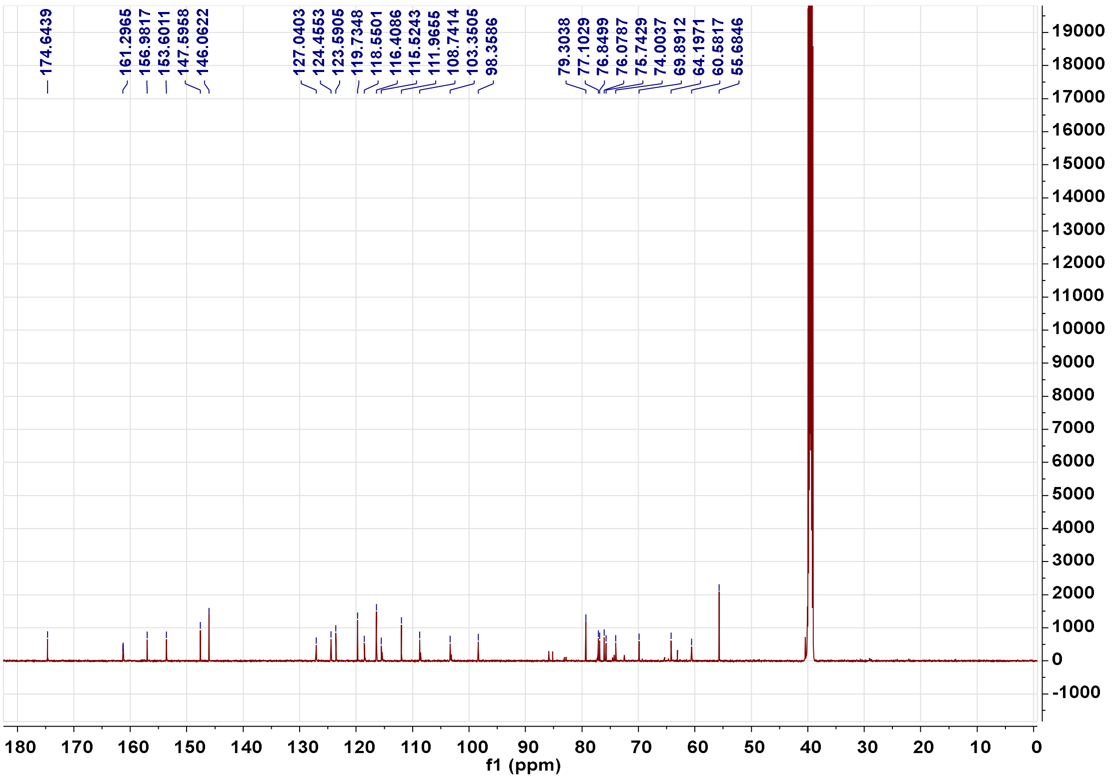

Supplementary Fig. 54 The ^{13}C NMR spectrum of **24a** in DMSO-d_6 (150 MHz).

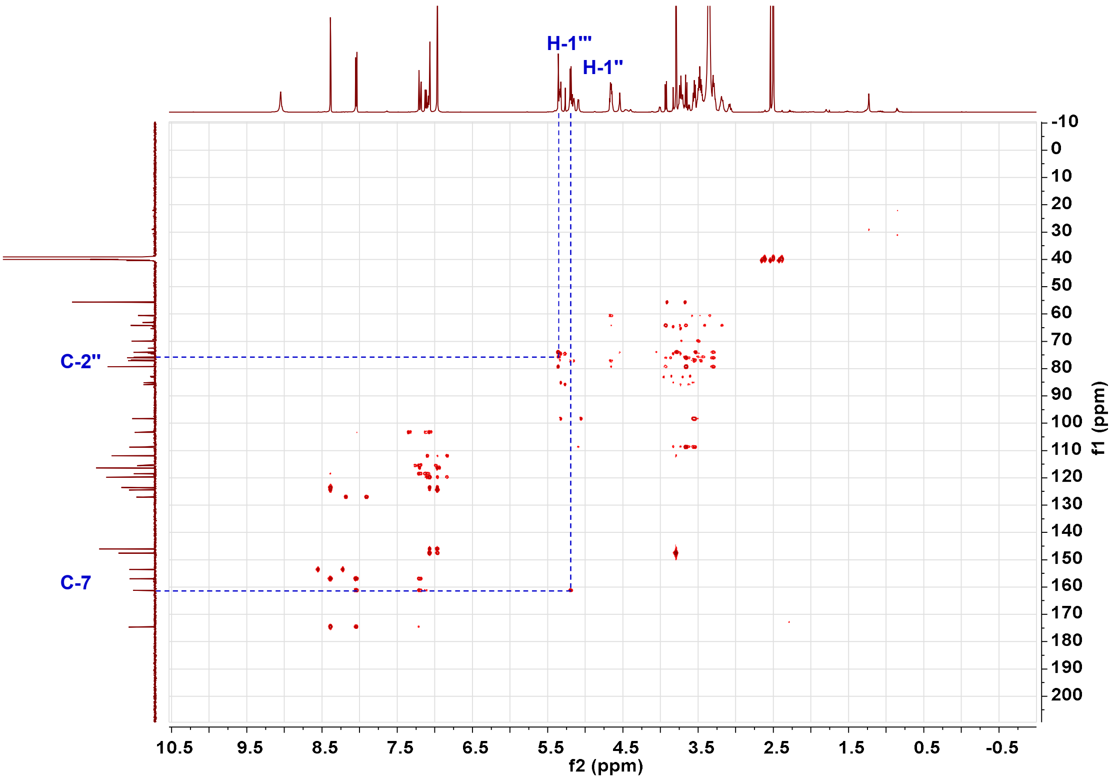

Supplementary Fig. 55 The HMBC spectrum of **24a** in DMSO-d_6 (600 MHz).

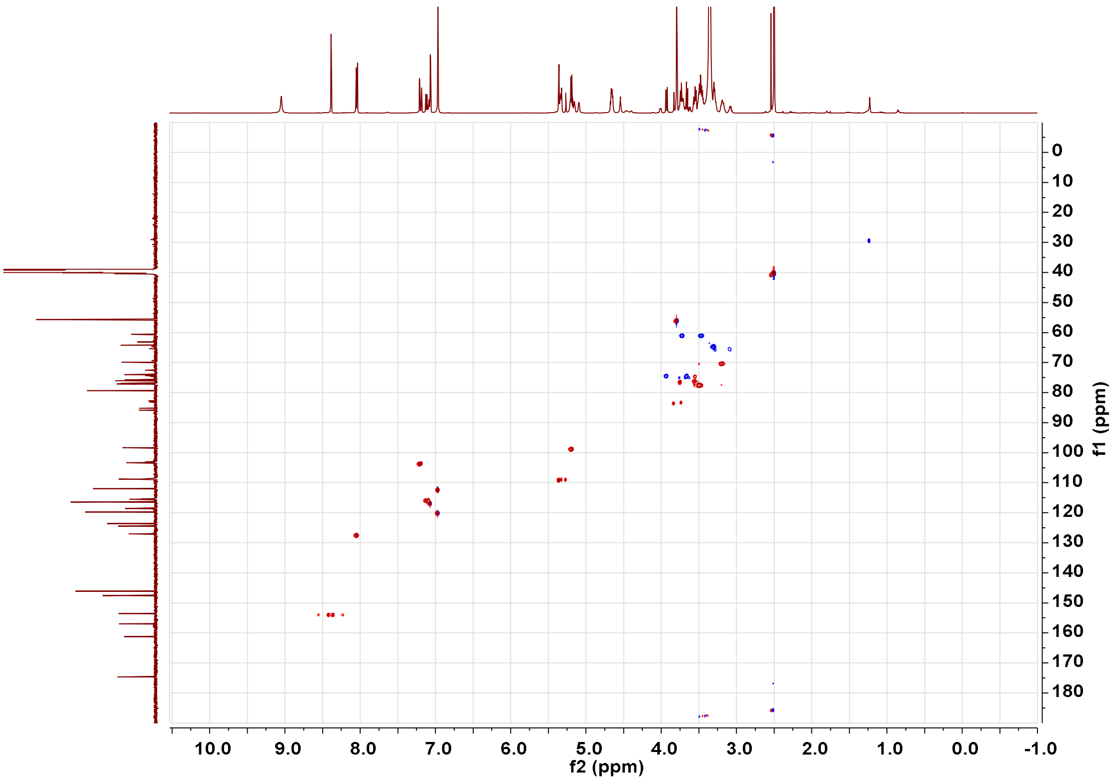

Supplementary Fig. 56 The HSQC spectrum of **24a** in DMSO-d_6 (600 MHz).

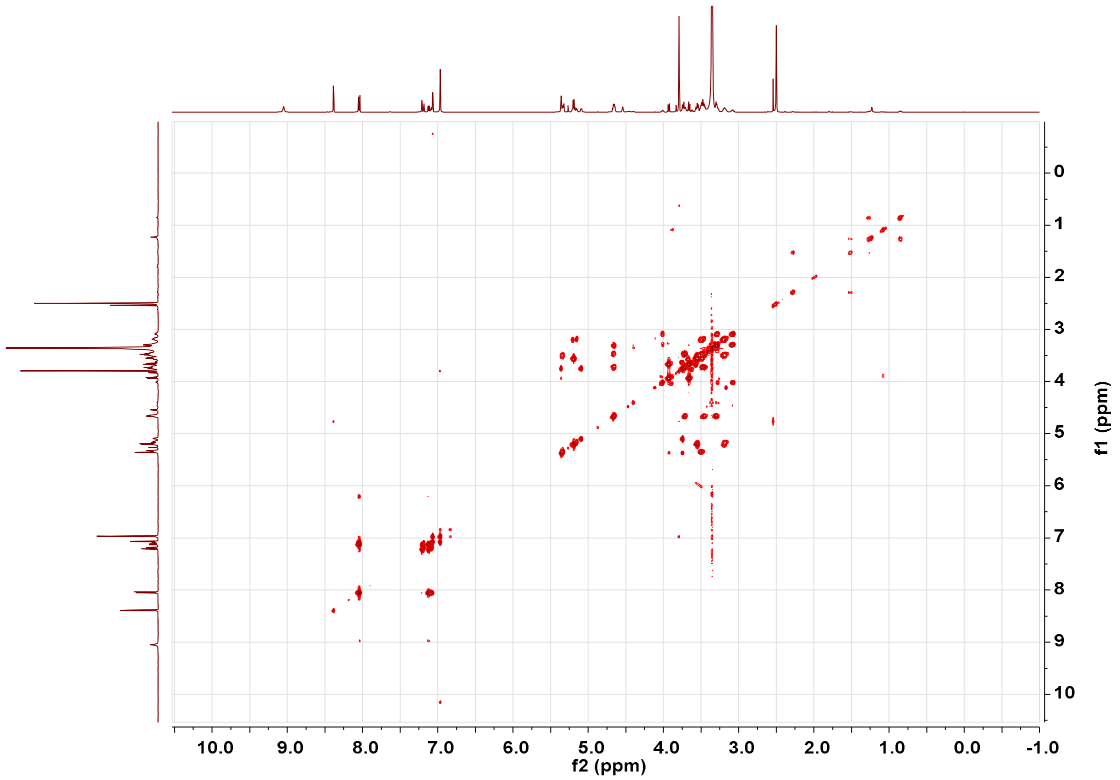

Supplementary Fig. 57 The ^{1}H-^{1}H COSY spectrum of **24a** in DMSO-d_6 (600 MHz).

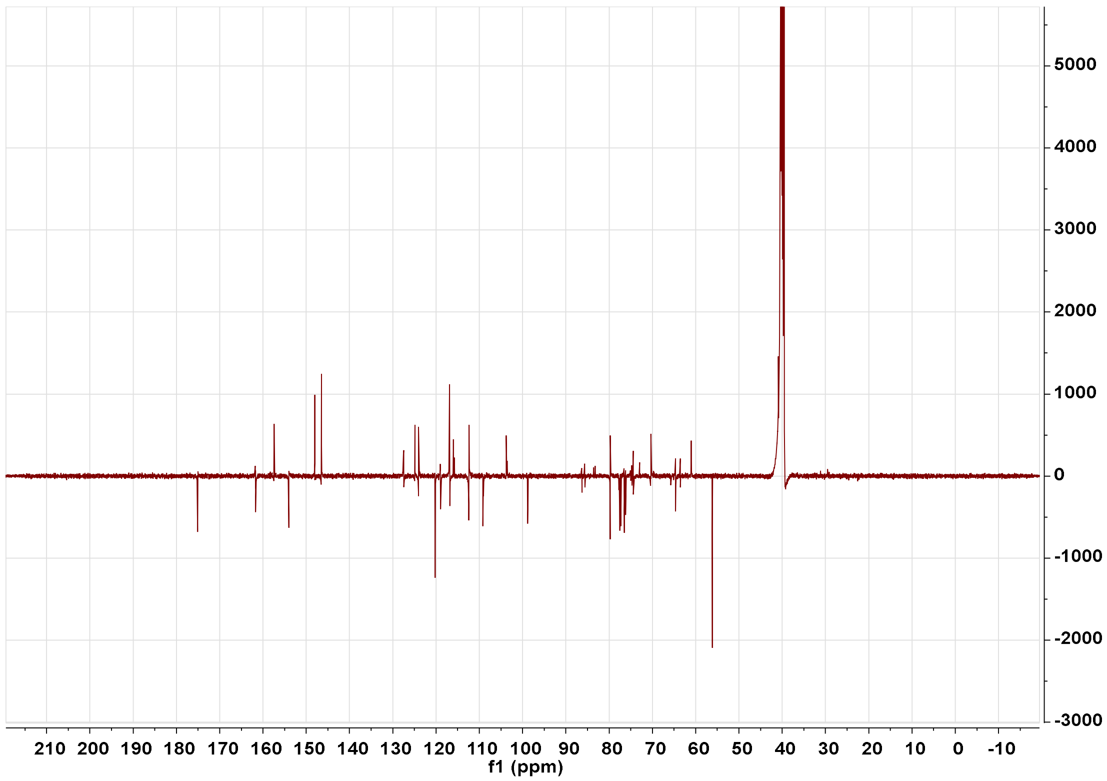

Supplementary Fig. 58 The DEPT 135 spectrum of **24a** in DMSO-d_6 (150 MHz).

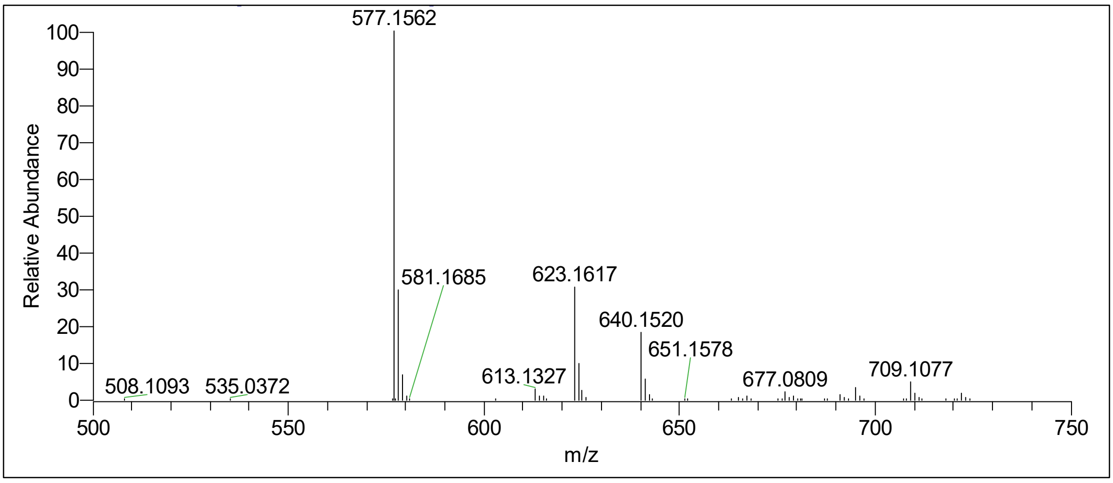

Supplementary Fig. 59 (-)-ESI-HRMS spectrum of **24a**.

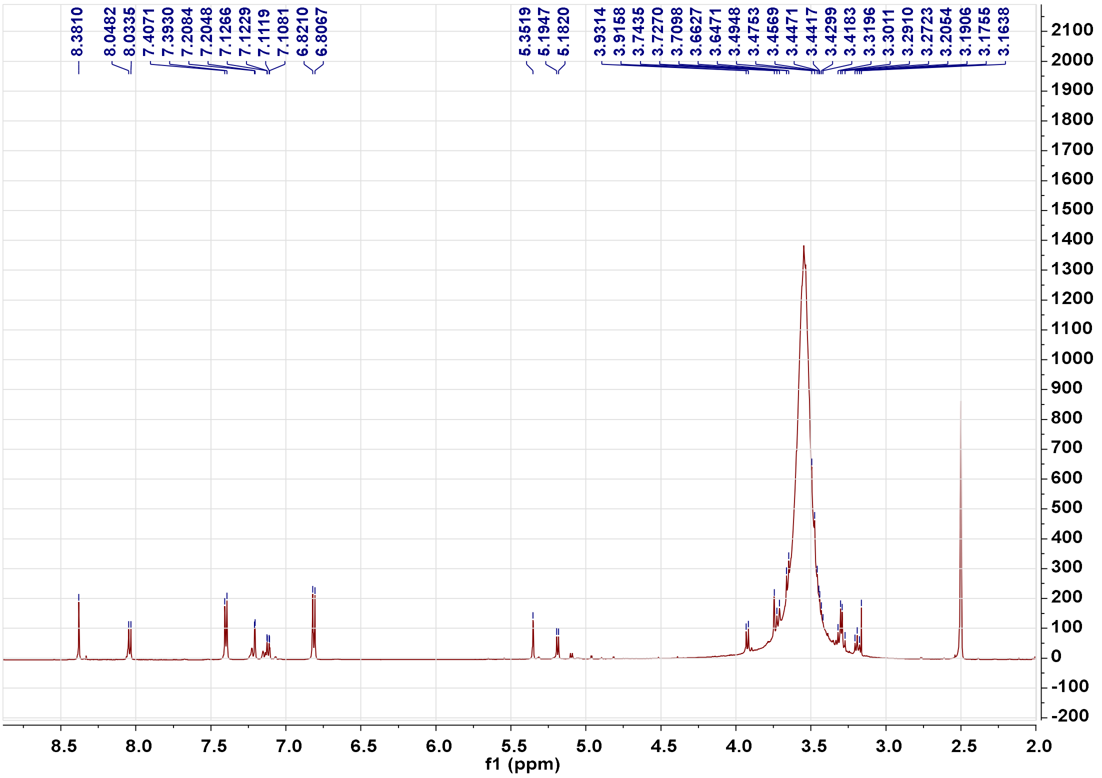

Supplementary Fig. 60 The ^{1}H NMR spectrum of **27a** in DMSO-d_6 (600 MHz).

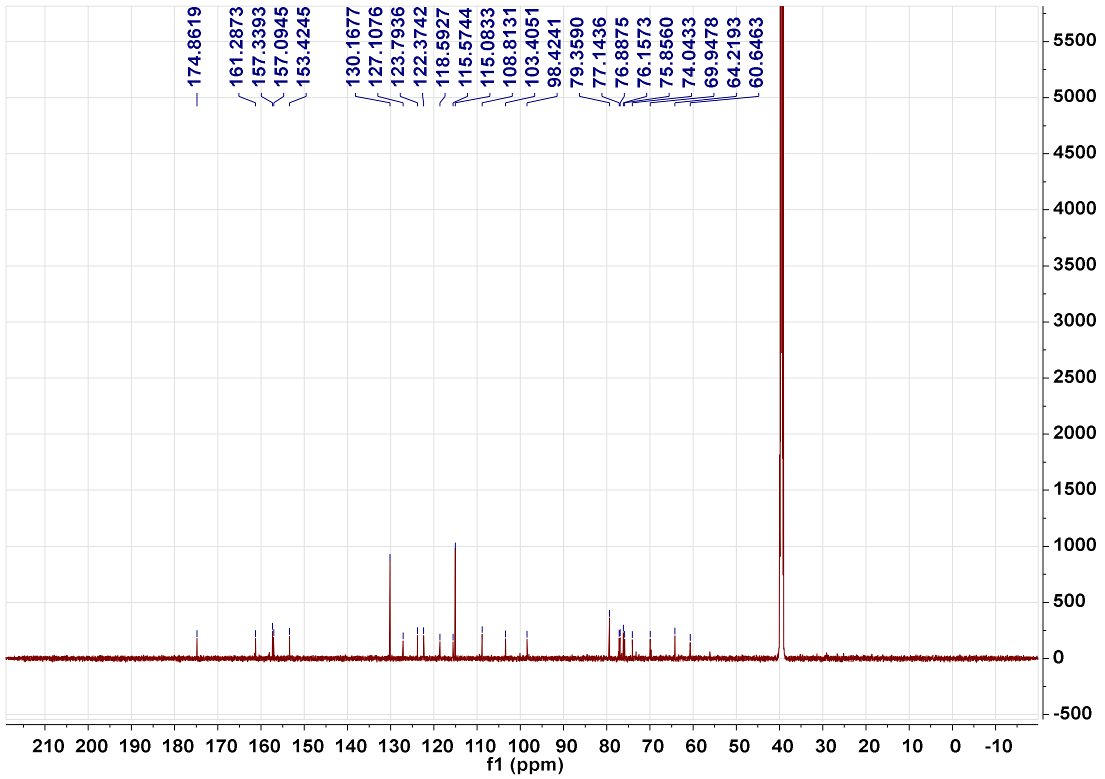

Supplementary Fig. 61 The ^{13}C NMR spectrum of **27a** in DMSO-d_6 (150 MHz).

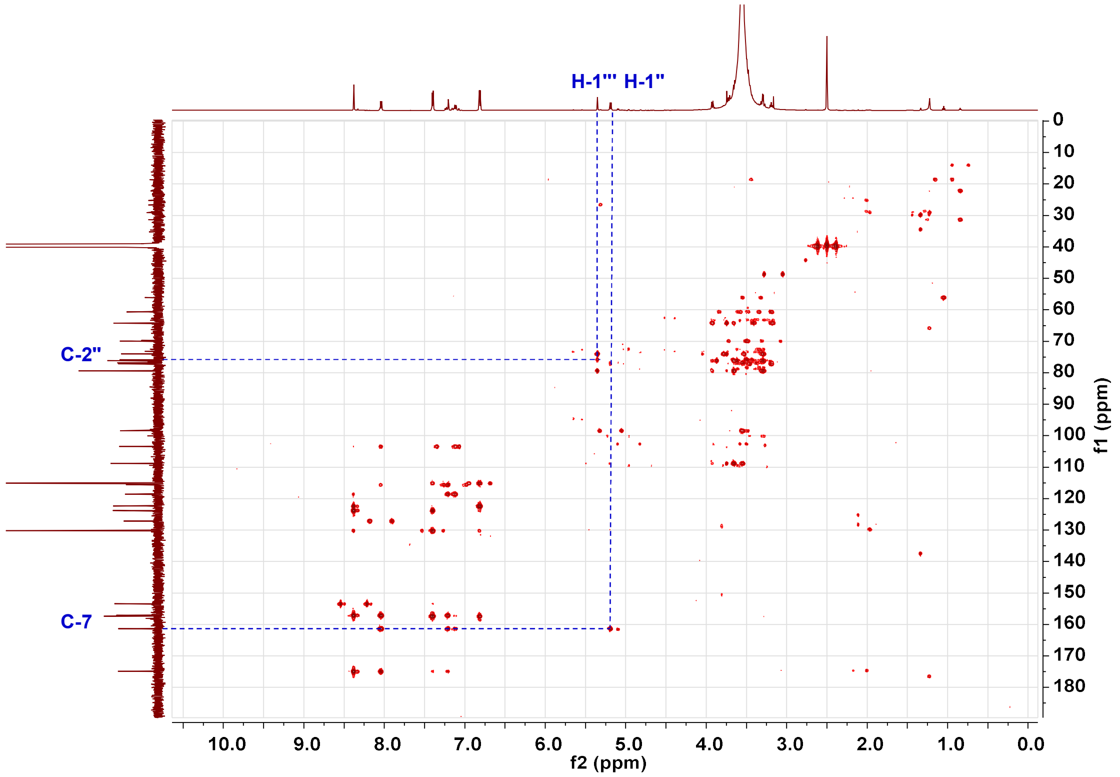

Supplementary Fig. 62 The HMBC spectrum of **27a** in DMSO-d_6 (600 MHz).

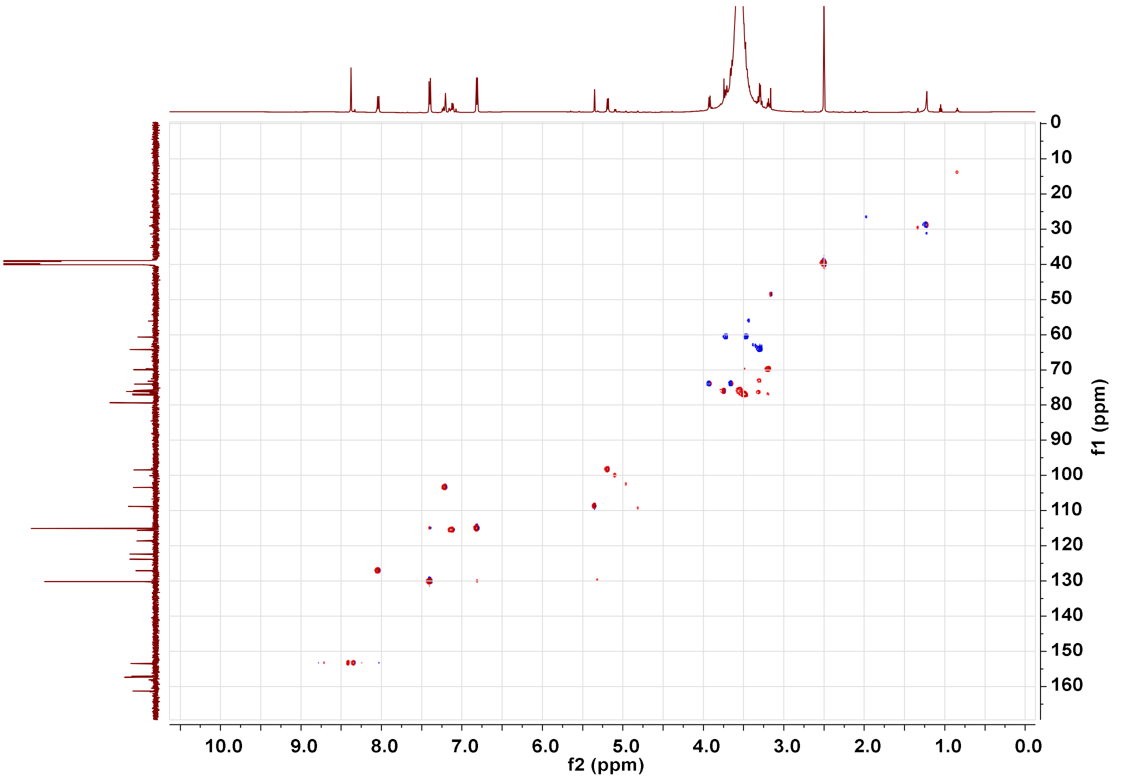

Supplementary Fig. 63 The HSQC spectrum of **27a** in DMSO-d_6 (600 MHz).

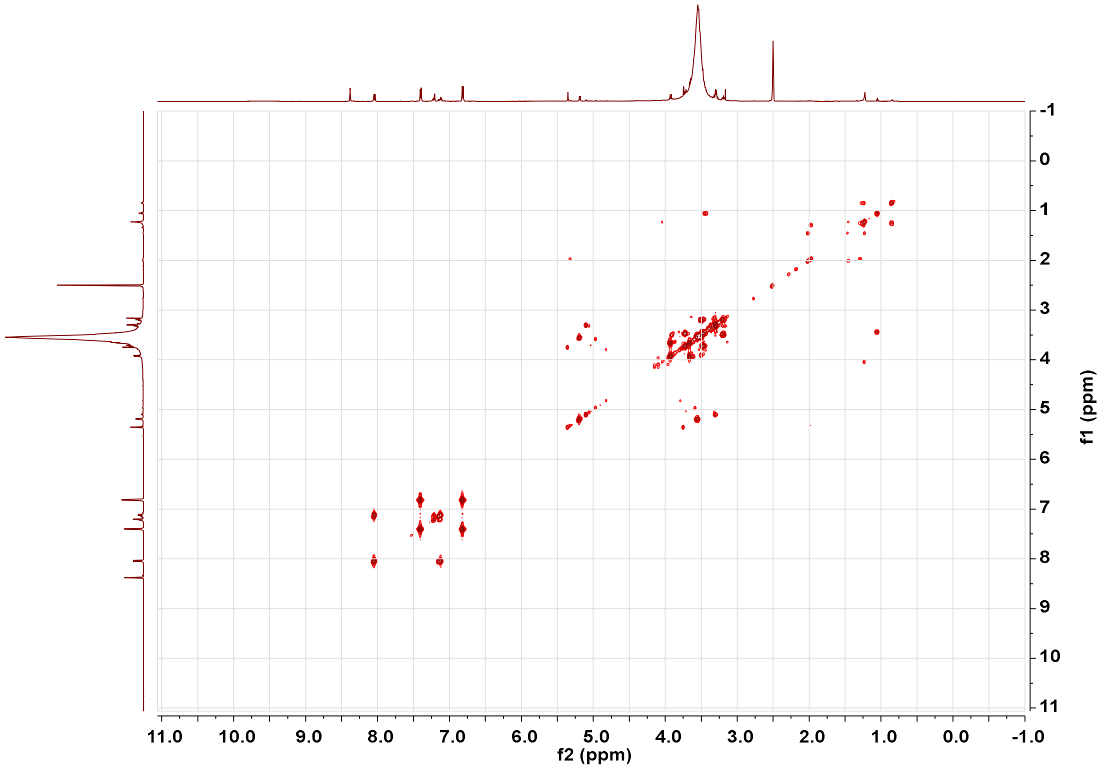

Supplementary Fig. 64 The ^{1}H-^{1}H COSY spectrum of **27a** in DMSO-d_6 (600 MHz).

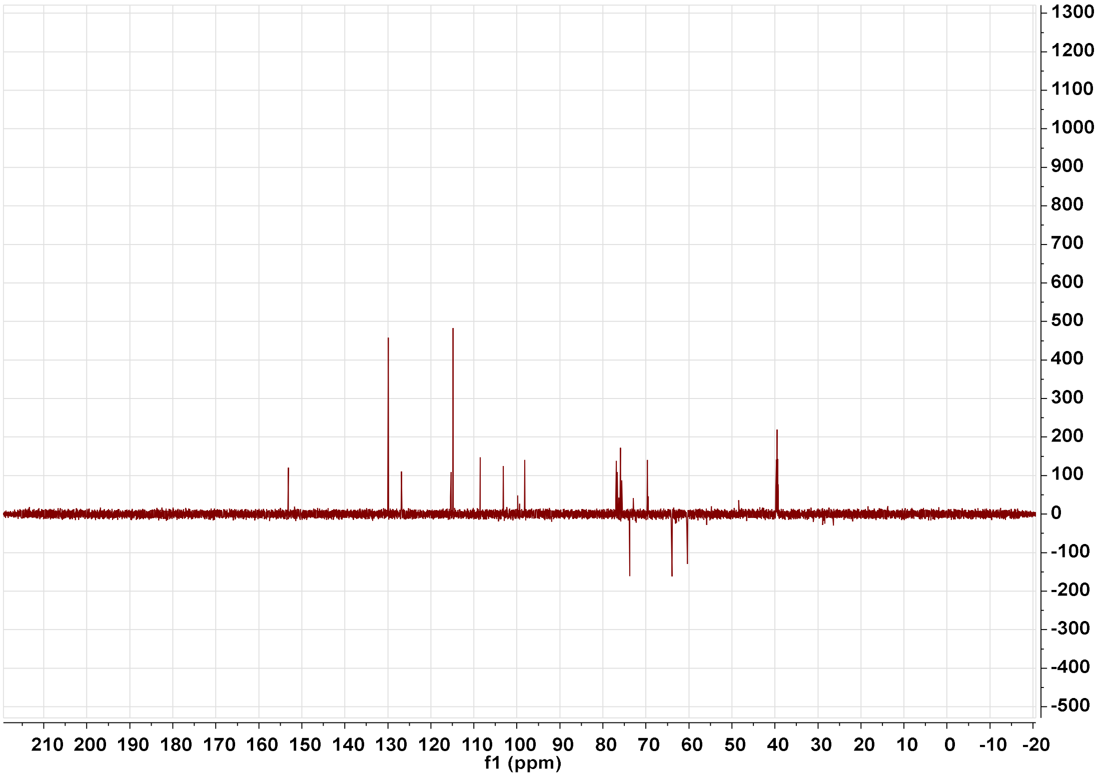

Supplementary Fig. 65 The DEPT 135 spectrum of **27a** in DMSO-d_6 (150 MHz).

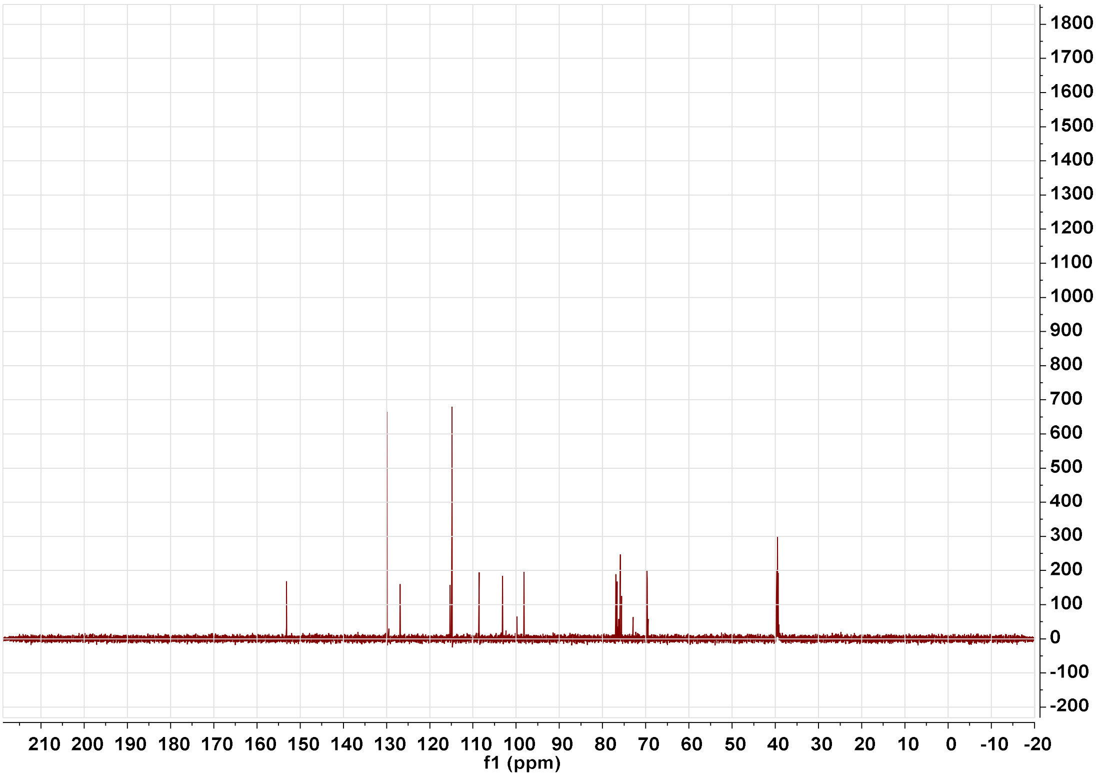

Supplementary Fig. 66 The DEPT 90 spectrum of **27a** in DMSO-d_6 (150 MHz).

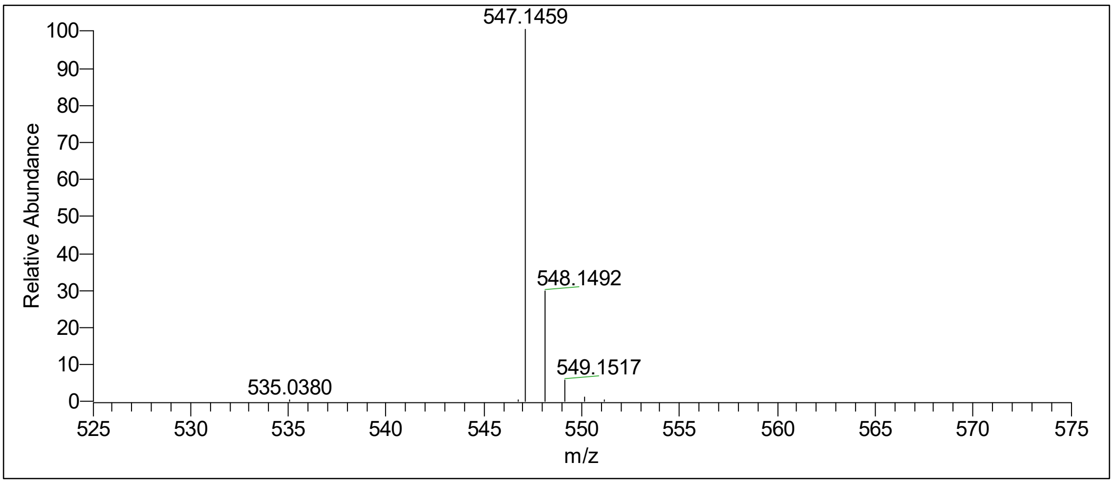

Supplementary Fig. 67 (-)-ESI-HRMS spectrum of **27a**.

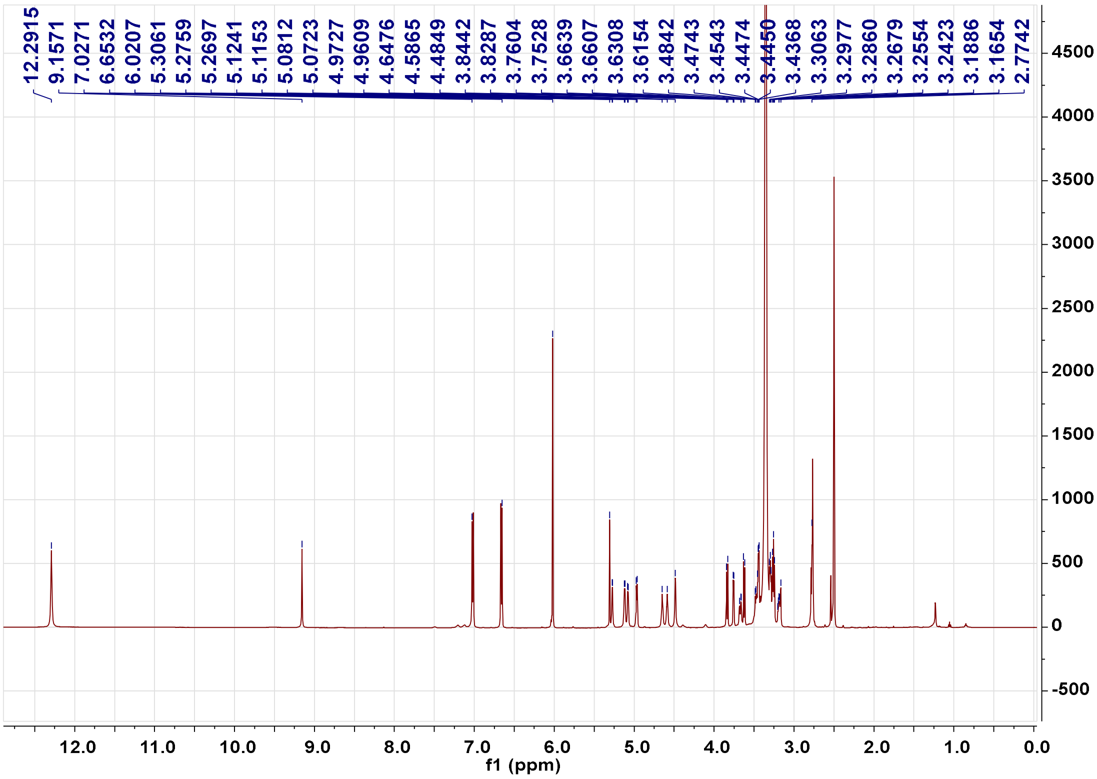

Supplementary Fig. 68 The ^{1}H NMR spectrum of **32a** in DMSO-d_6 (600 MHz).

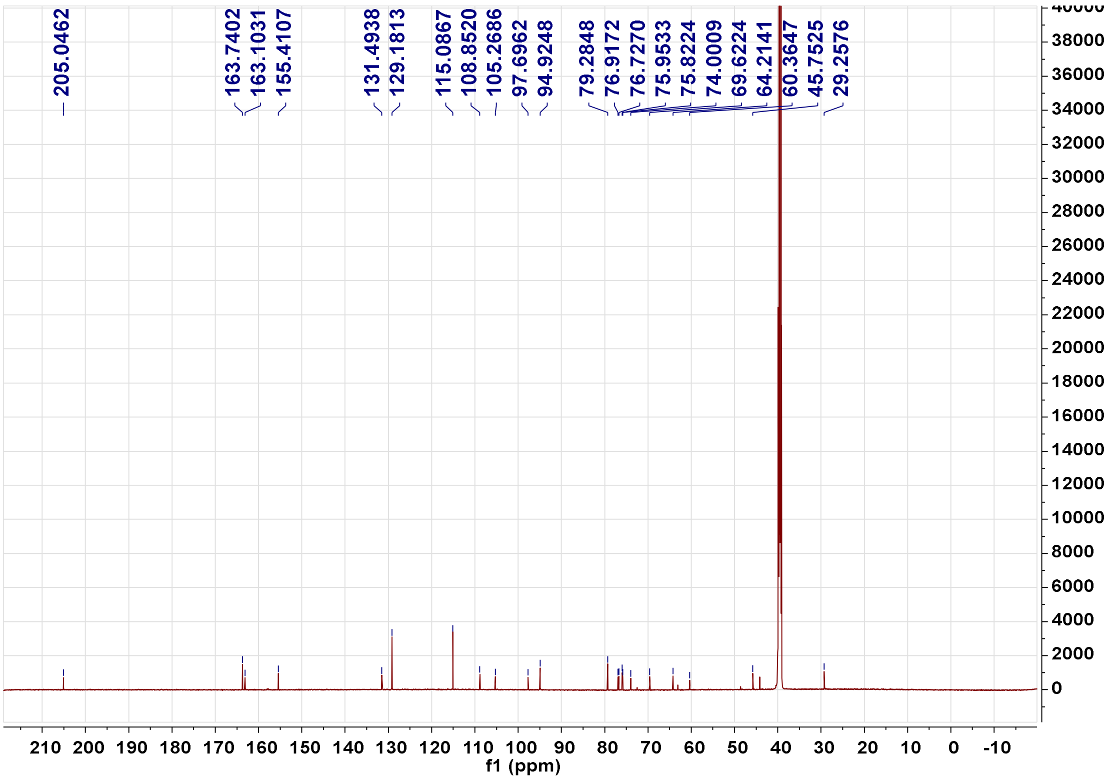

Supplementary Fig. 69 The ^{13}C NMR spectrum of **32a** in DMSO-d_6 (150 MHz).

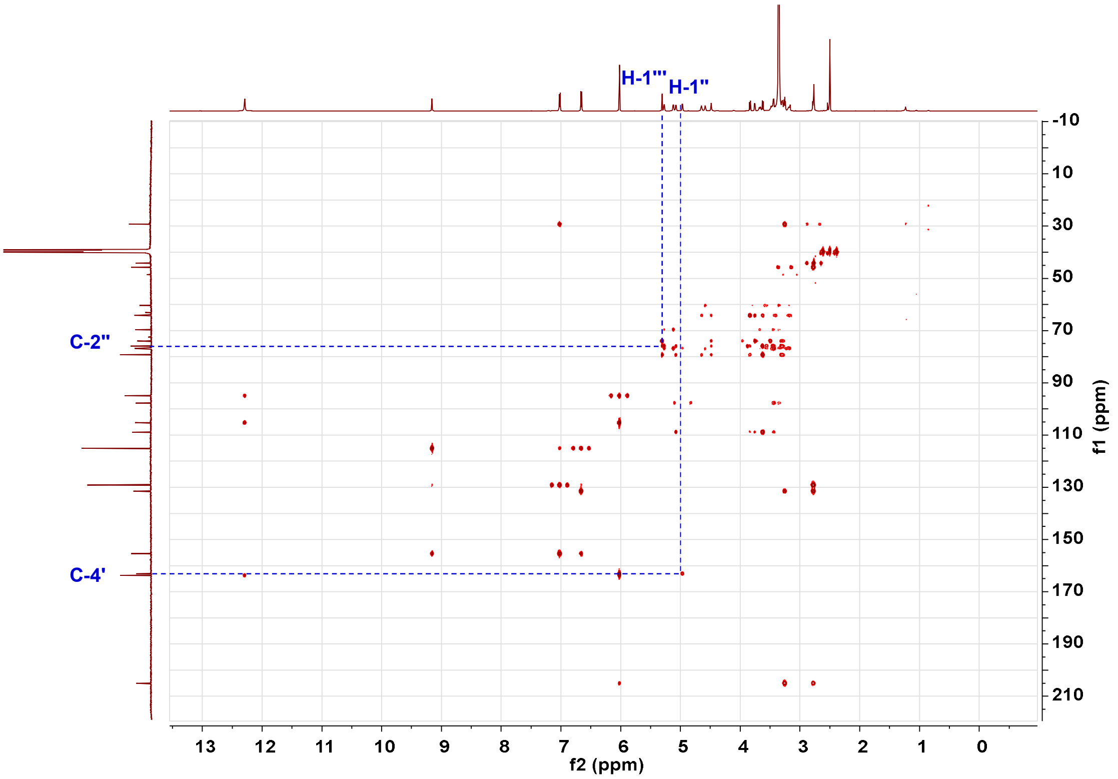

Supplementary Fig. 70 The HMBC spectrum of **32a** in DMSO-d_6 (600 MHz).

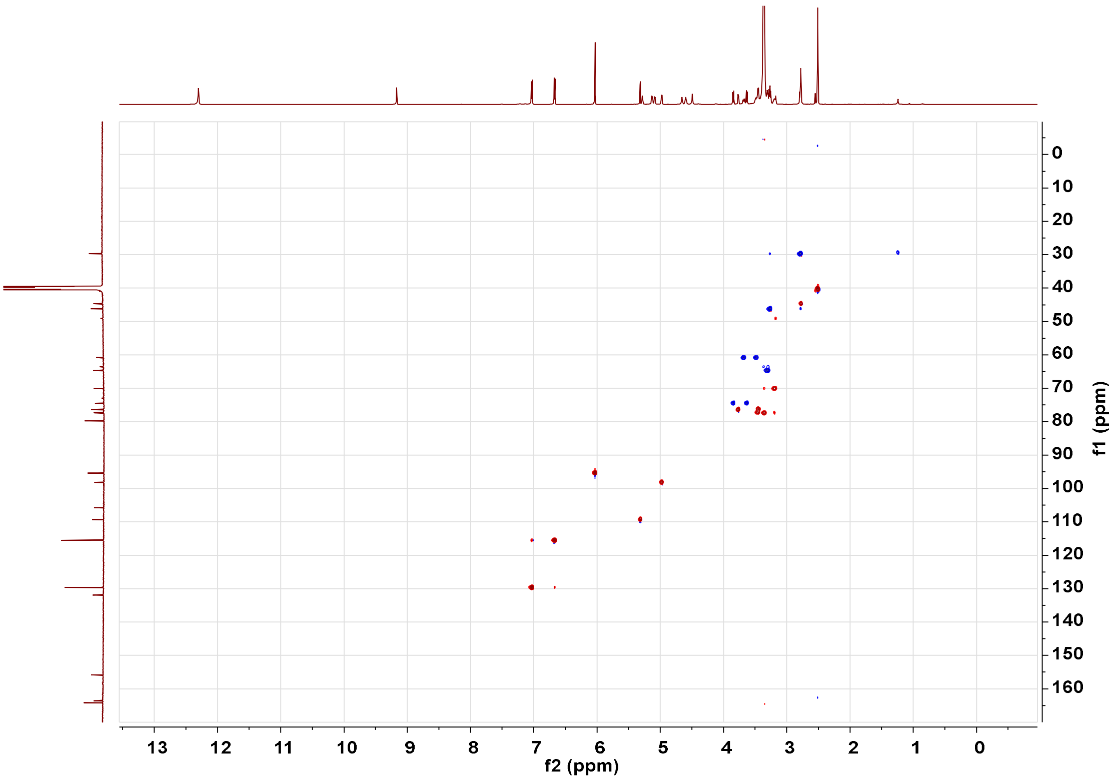

Supplementary Fig. 71 The HSQC spectrum of **32a** in DMSO-d_6 (600 MHz).

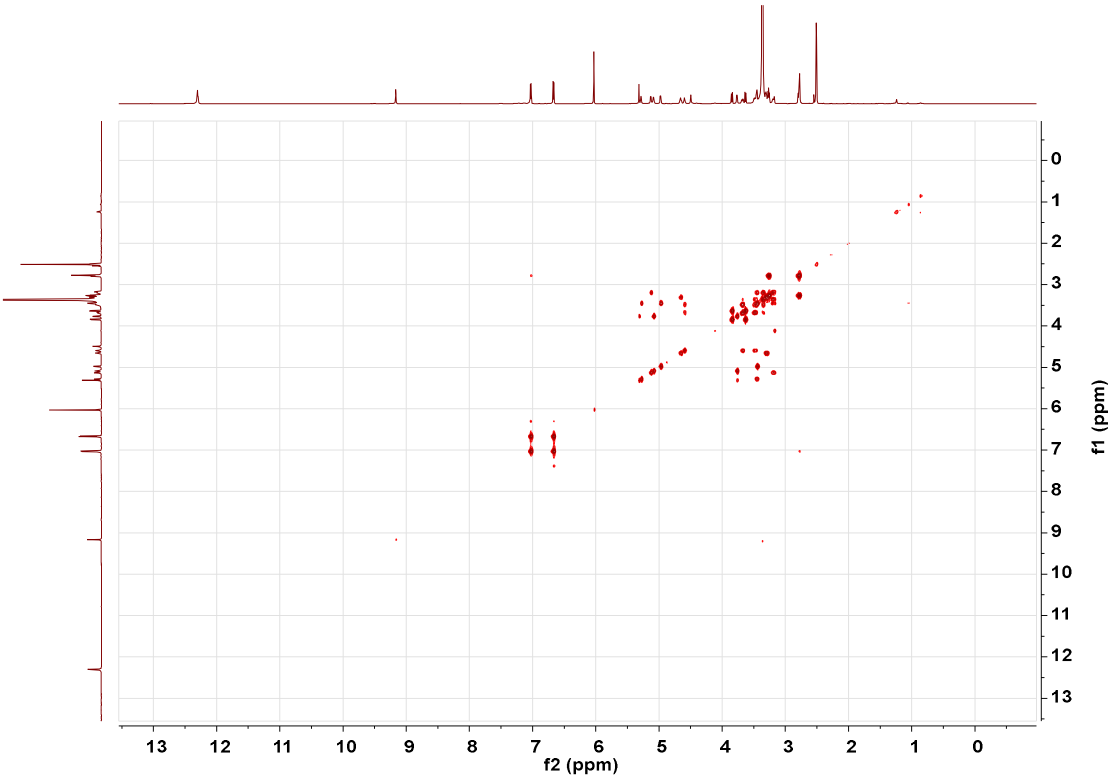

Supplementary Fig. 72 The ^{1}H-^{1}H COSY spectrum of **32a** in DMSO-d_6 (600 MHz).

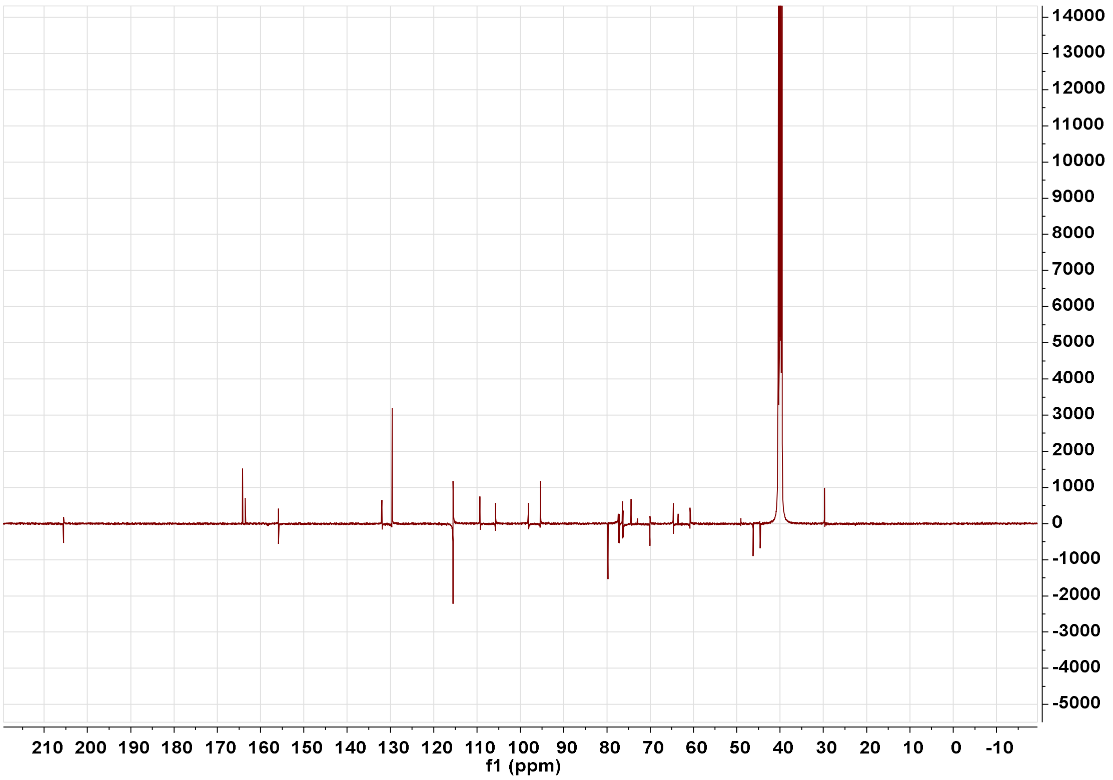

Supplementary Fig. 73 The DEPT 135 spectrum of **32a** in DMSO-d_6 (150 MHz).

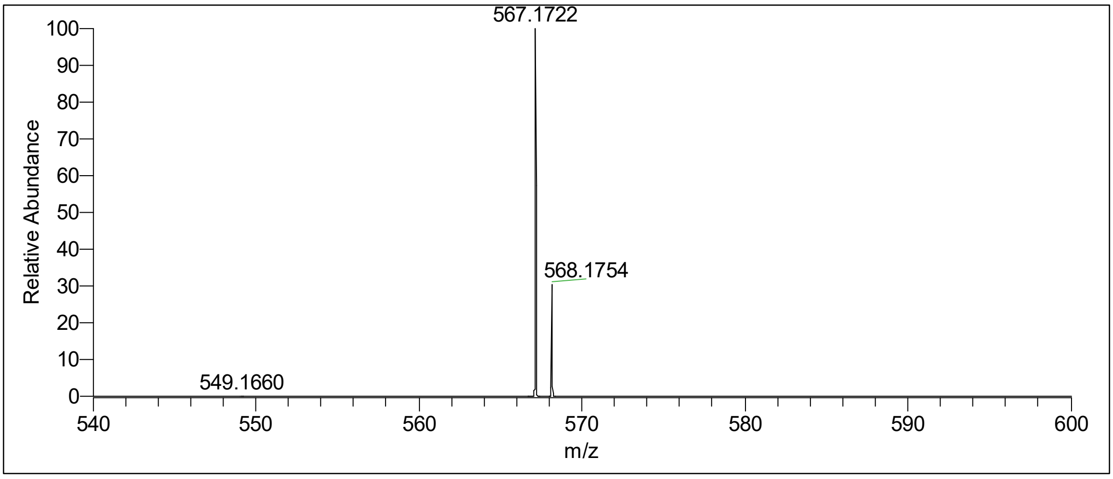

Supplementary Fig. 74 (-)-ESI-HRMS spectrum of **32a**.

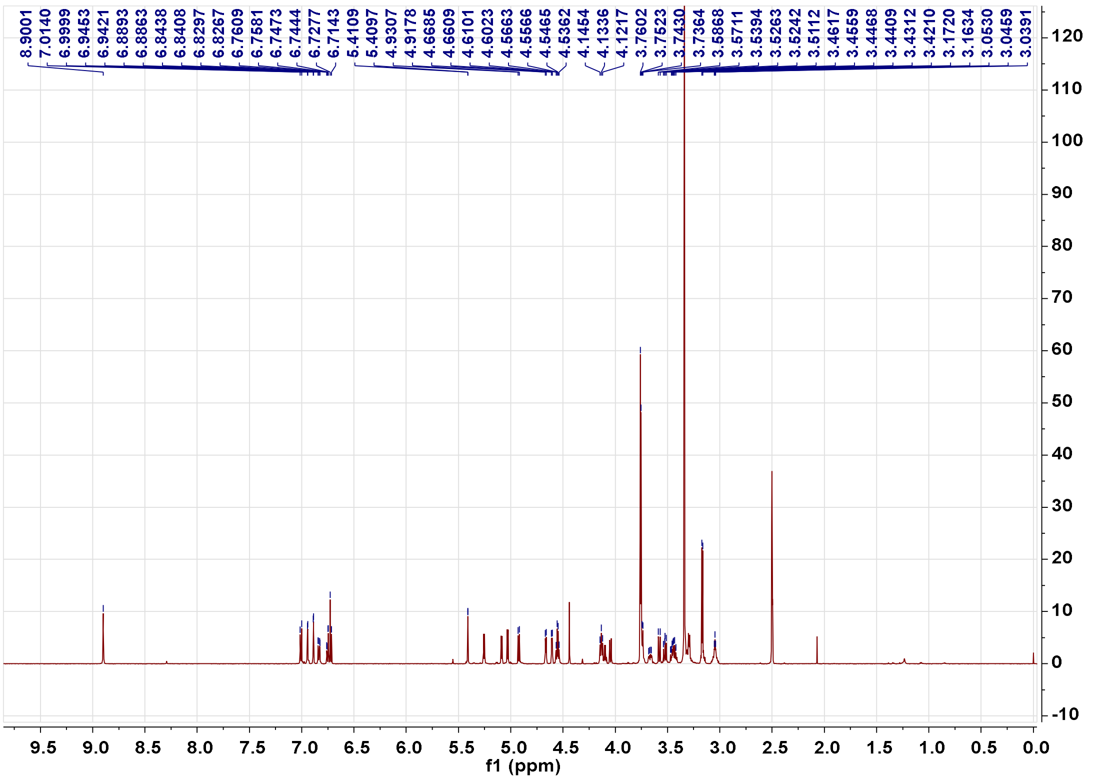

Supplementary Fig. 75 The ^{1}H NMR spectrum of **35a** in DMSO-d_6 (600 MHz).

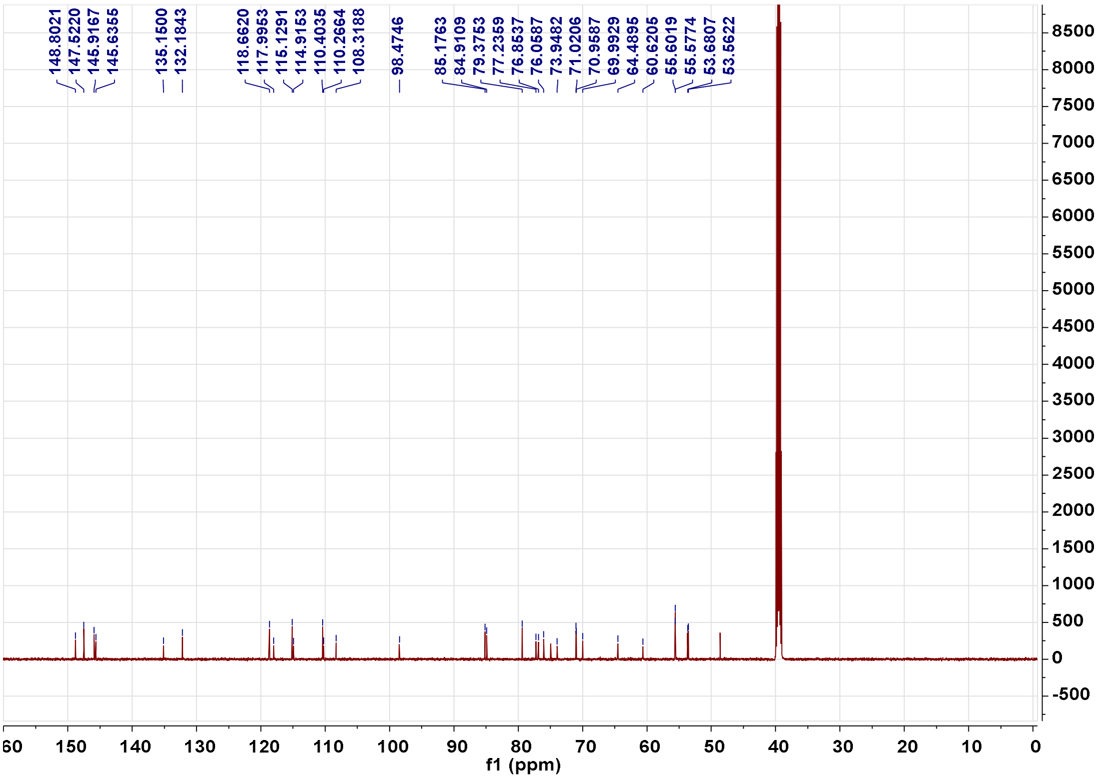

Supplementary Fig. 76 The ^{13}C NMR spectrum of **35a** in DMSO-d_6 (150 MHz).

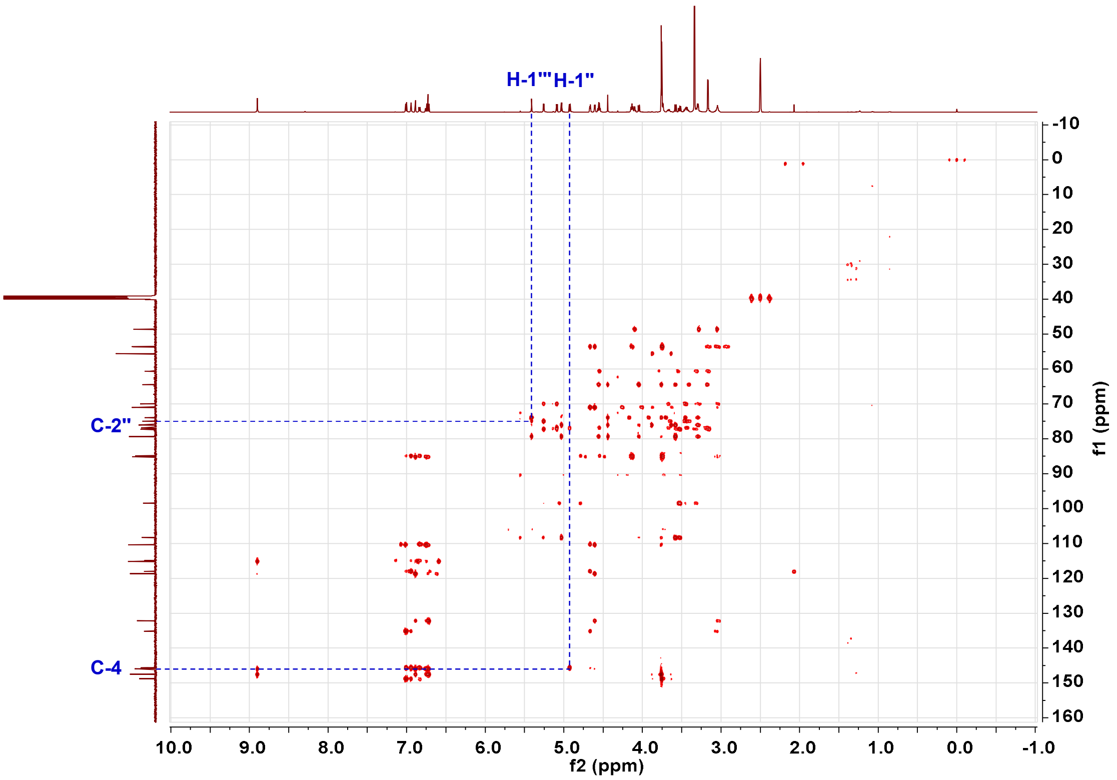

Supplementary Fig. 77 The HMBC spectrum of **35a** in DMSO-d_6 (600 MHz).

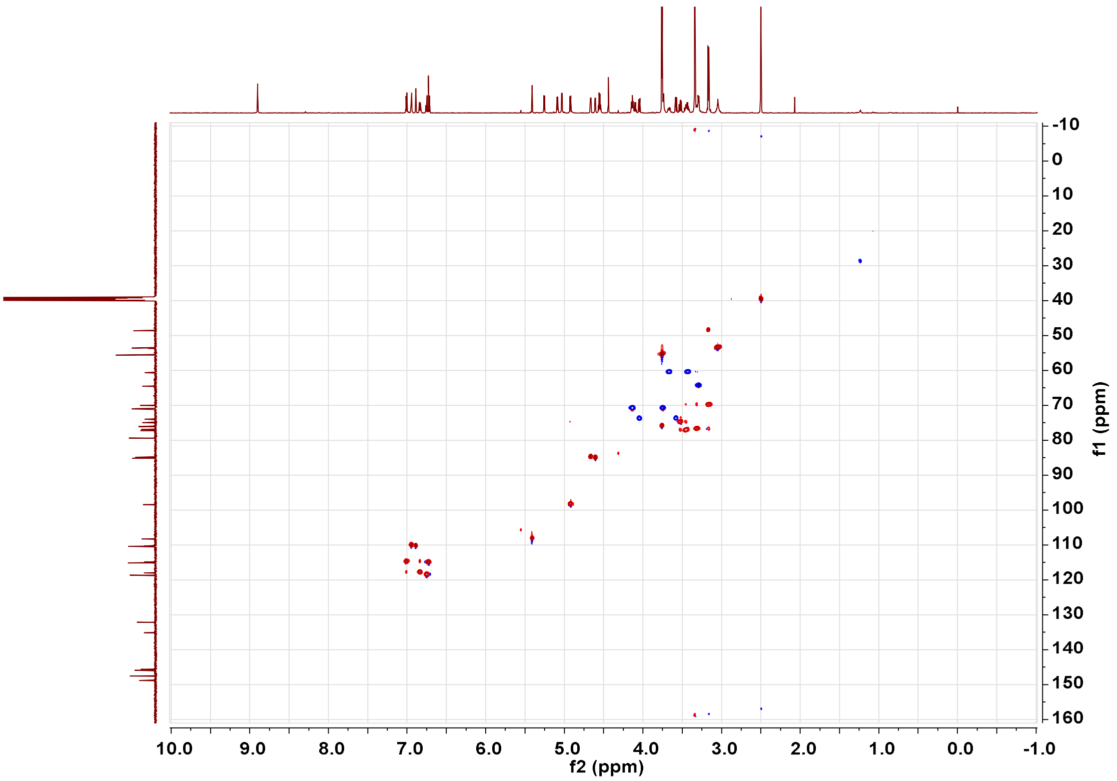

Supplementary Fig. 78 The HSQC spectrum of **35a** in DMSO-d_6 (600 MHz).

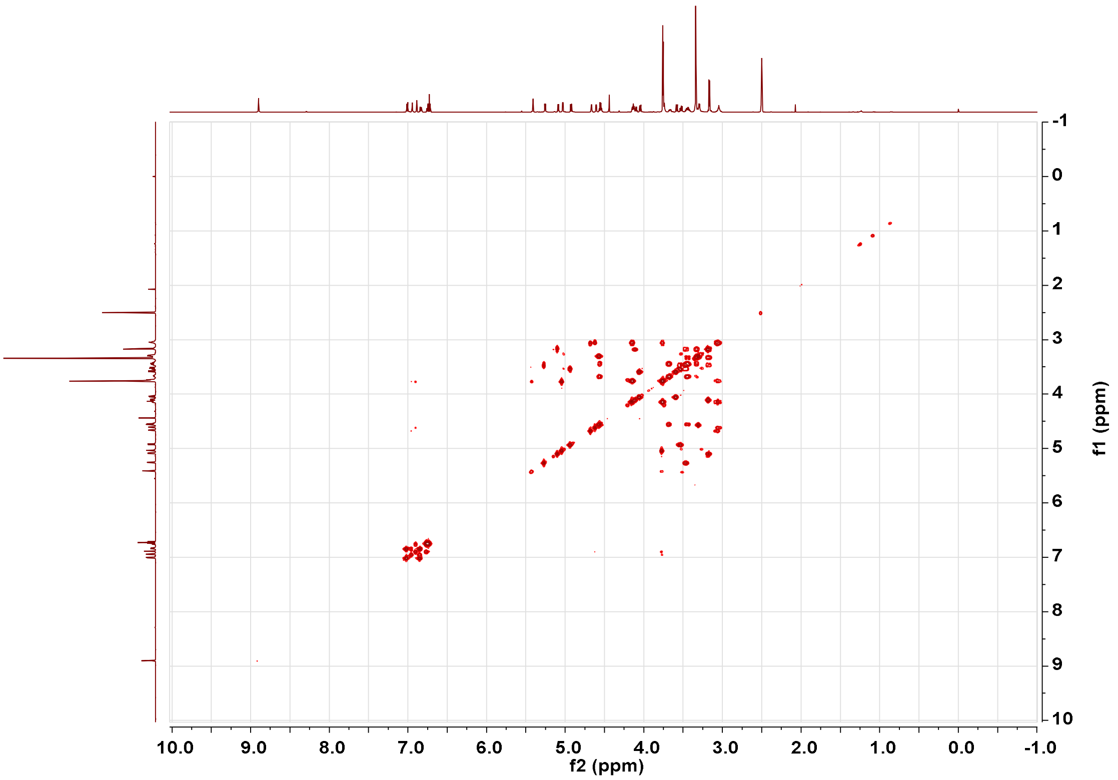

Supplementary Fig. 79 The ^{1}H-^{1}H COSY spectrum of **35a** in DMSO-d_6 (600 MHz).

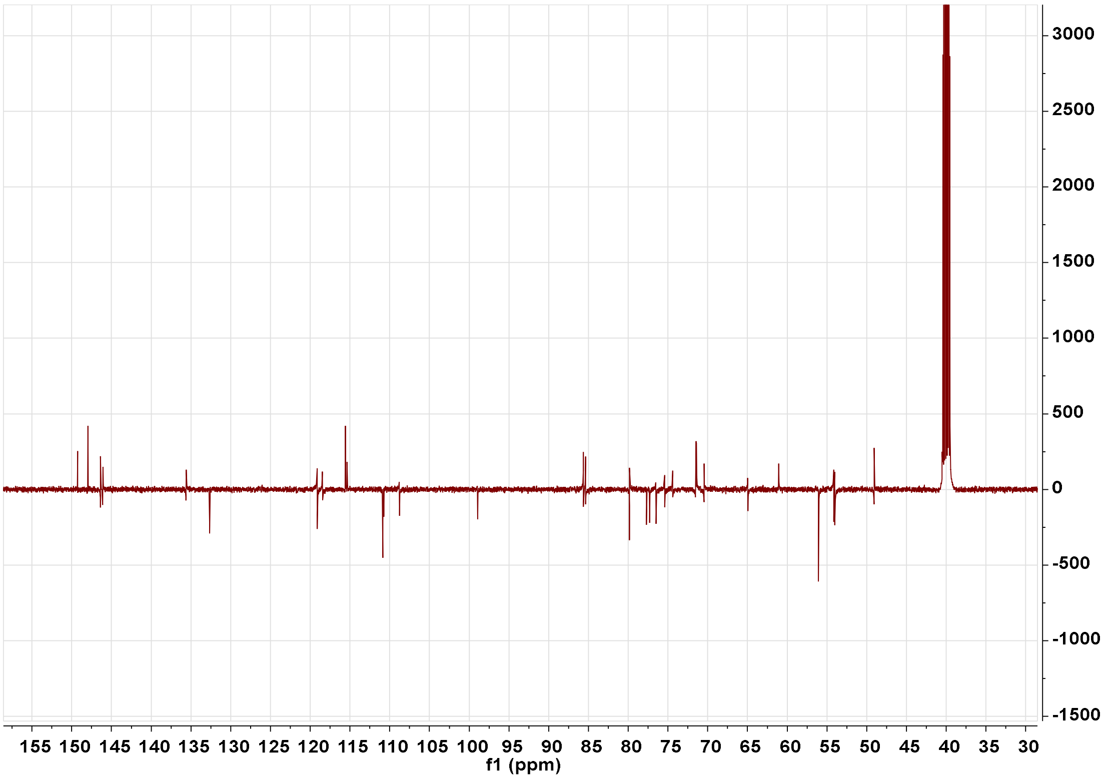

Supplementary Fig. 80 The DEPT 135 spectrum of **35a** in DMSO-d_6 (150 MHz).

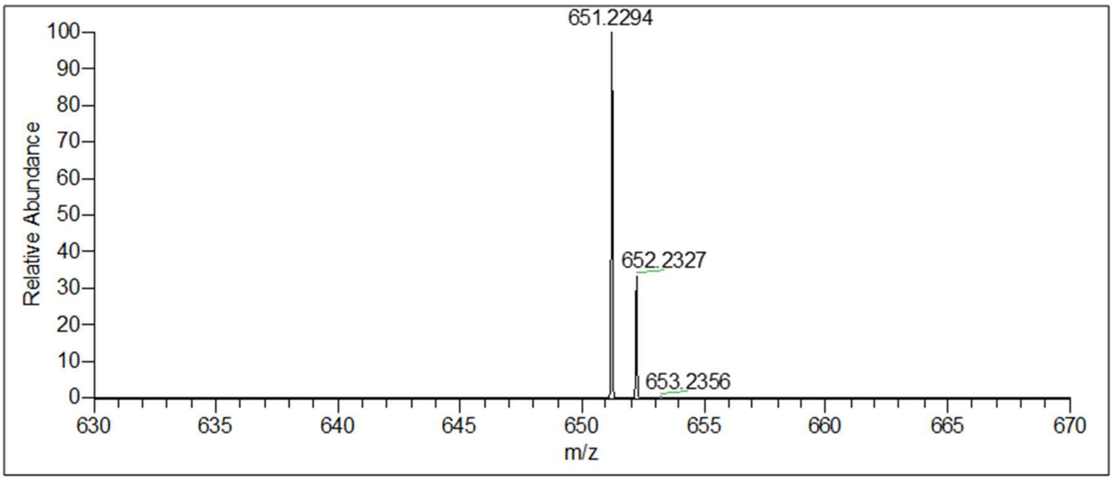

Supplementary Fig. 81 (-)-ESI-HRMS spectrum of **35a**.

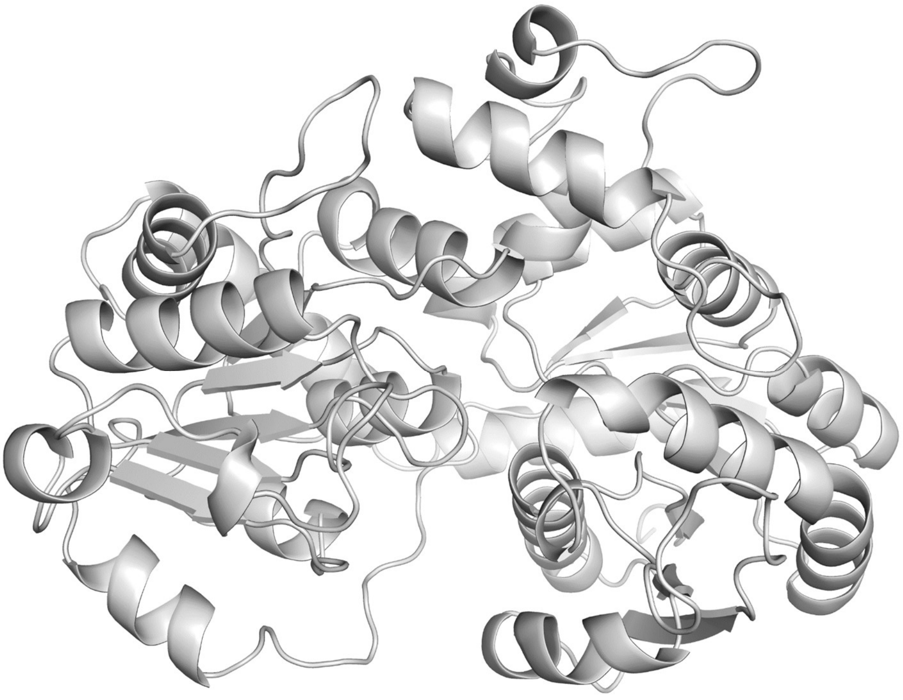

Supplementary Fig. 82 The crystal model of GuApiGT predicted by AlphaFold2.

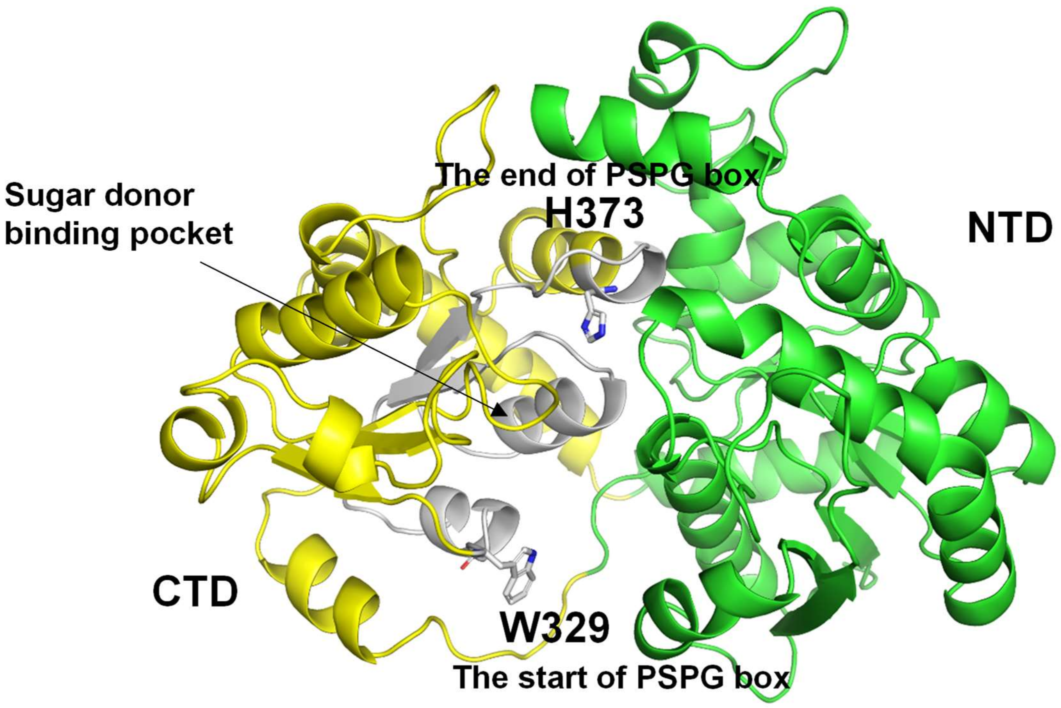

Supplementary Fig. 83 The PSPG box (W329-H373, colored in gray) in crystal structure of GuApiGT.

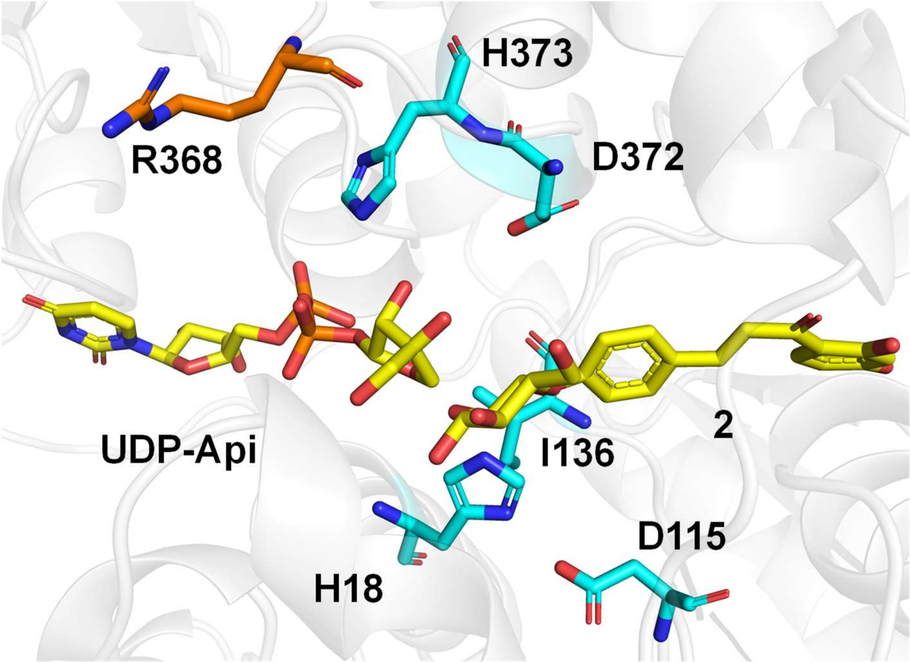

Supplementary Fig. 84 The initial binding mode of GuApiGT/UDP-Api/**2**.

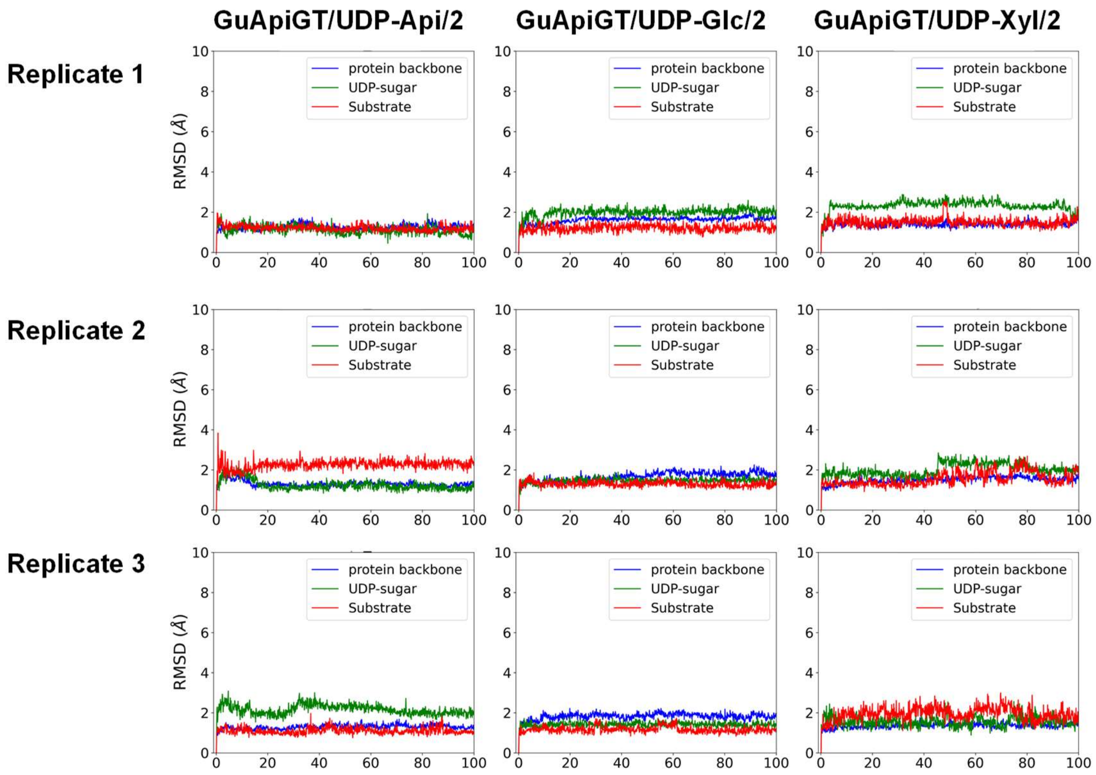

Supplementary Fig. 85 Time evolution of root mean square deviations (RMSD) of protein backbone (CA, Cα atoms), substrate (**2**), and UDP-sugar for MD simulation systems (three independent 100-ns MD simulations for each system). The RMSD was smaller than 5 Å, indicating the binding mode was stable.

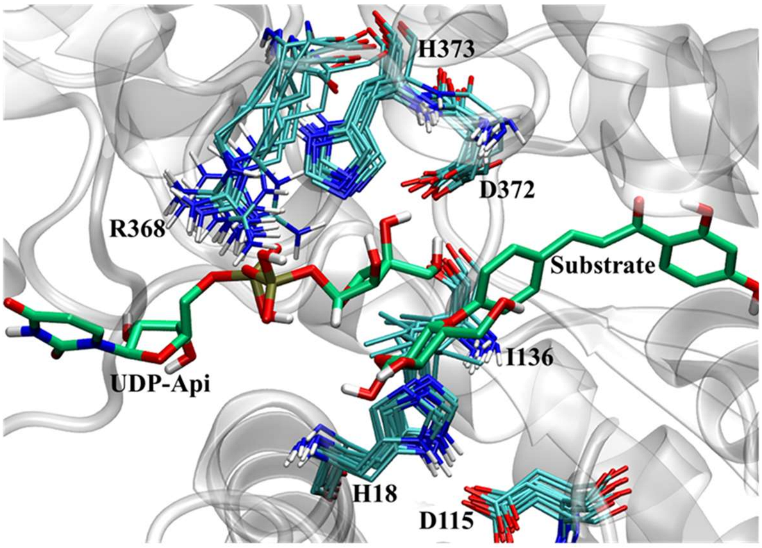

Supplementary Fig. 86 The superimposition of GuApiGT/UDP-Api/**2** snapshots in MD simulations.

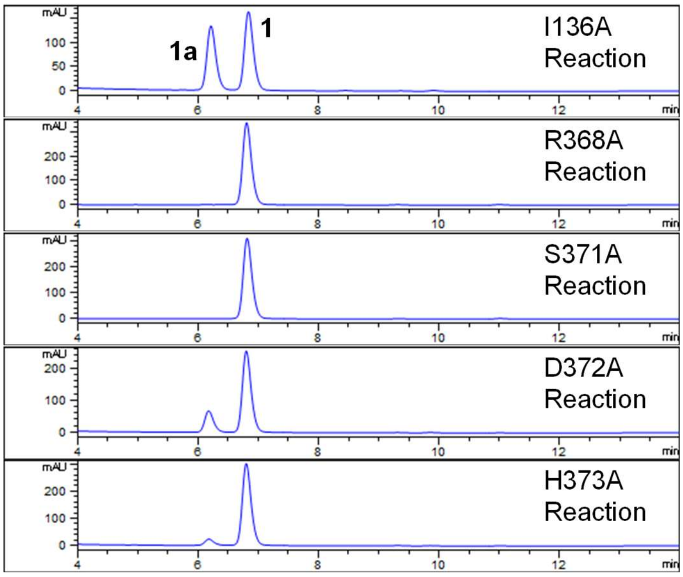

Supplementary Fig. 87 HPLC analysis of GuApiGT mutants catalyzed products.

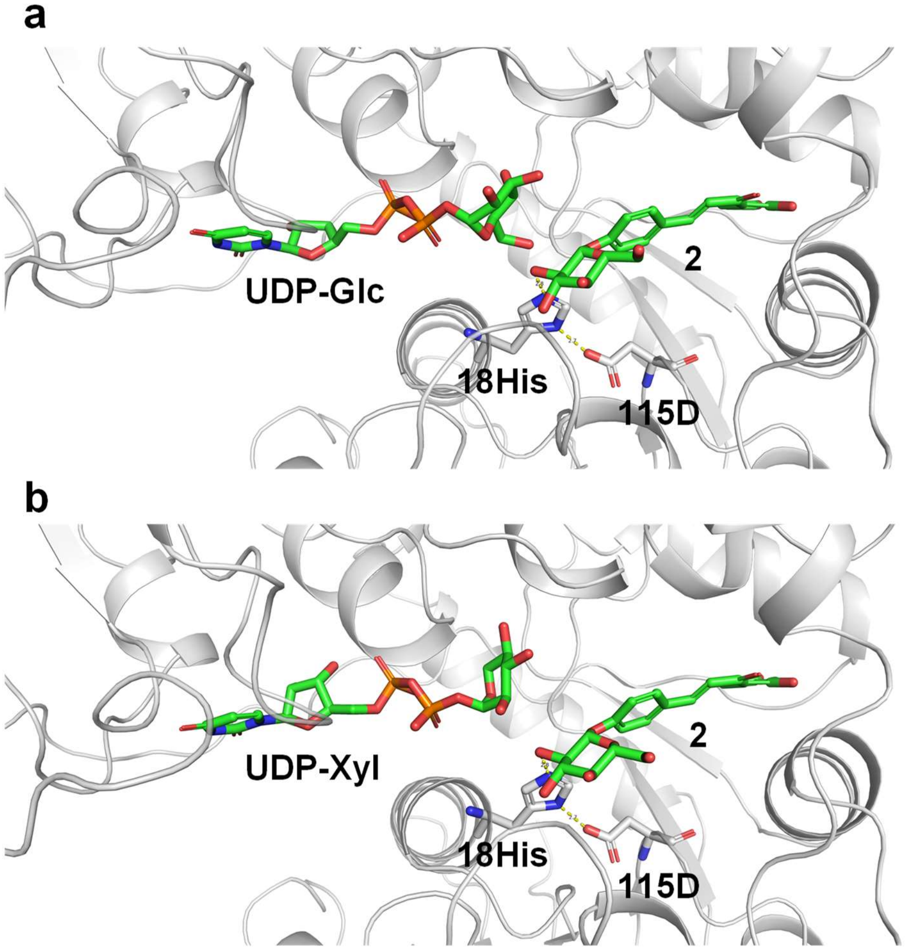

Supplementary Fig. 88 The complex models of GuApiGT/UDP-Glc/**2** (**a**) and GuApiGT/UDP-Xyl/**2** (**b**).

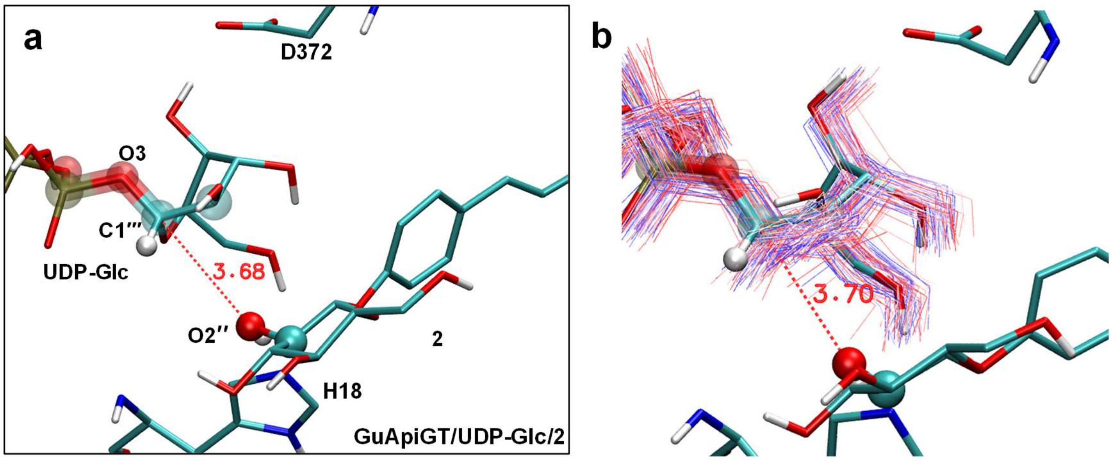

Supplementary Fig. 89 The MD snapshots of GuApiGT/UDP-Glc/**2**. **a**, Representative MD snapshots of GuApiGT/UDP-Glc/**2**. **b**, The superimposition of snapshots.

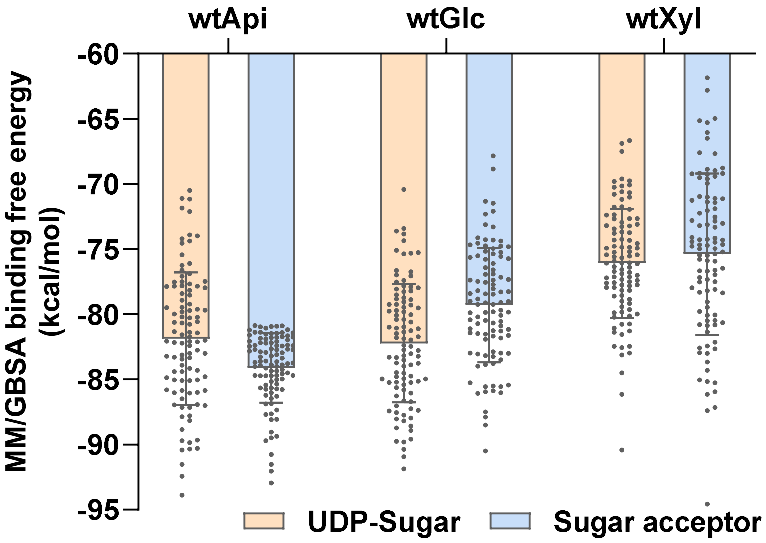

Supplementary Fig. 90 The MM/GBSA binding free energy in different systems (wtApi, GuApiGT/UDP-Api/2; wtGlc, GuApiGT/UDP-Glc/**2**; wtXyl, GuApiGT/UDP-Xyl/**2**). Data are presented as mean values ± SD (*n*=100 biologically independent samples). The source data underlying figure are provided in a Source Data file.

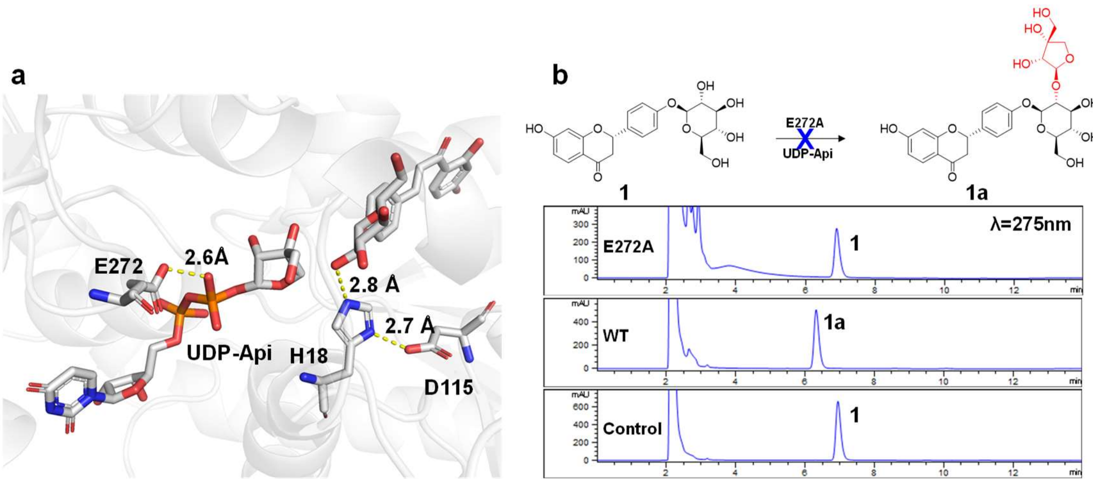

Supplementary Fig. 91 The function of E272 in the structure of GuApiGT. **a**, The binding conformation of UDP-Api during apiosylation in GuApiGT. The hydrogen-bond interactions are shown as yellow dashes. **b**, HPLC analysis of E272A catalyzed product using **1** as the substrate. UDP-Api was produced by adding UDP-GlcA, purified UAXS, and NAD^+ to the mixed system. The chromatographic peak with a retention time of around 2 min is the protein peak.

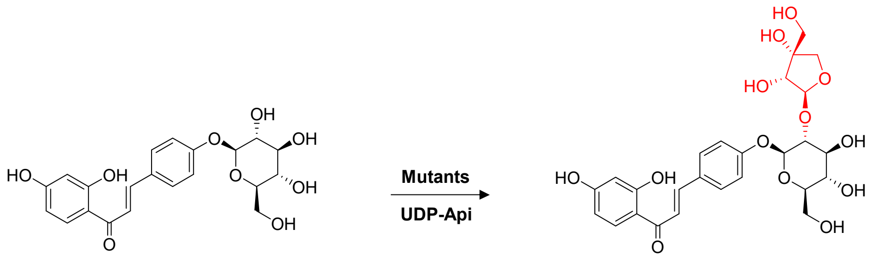

2
MW：418.4

2a
MW：550.5

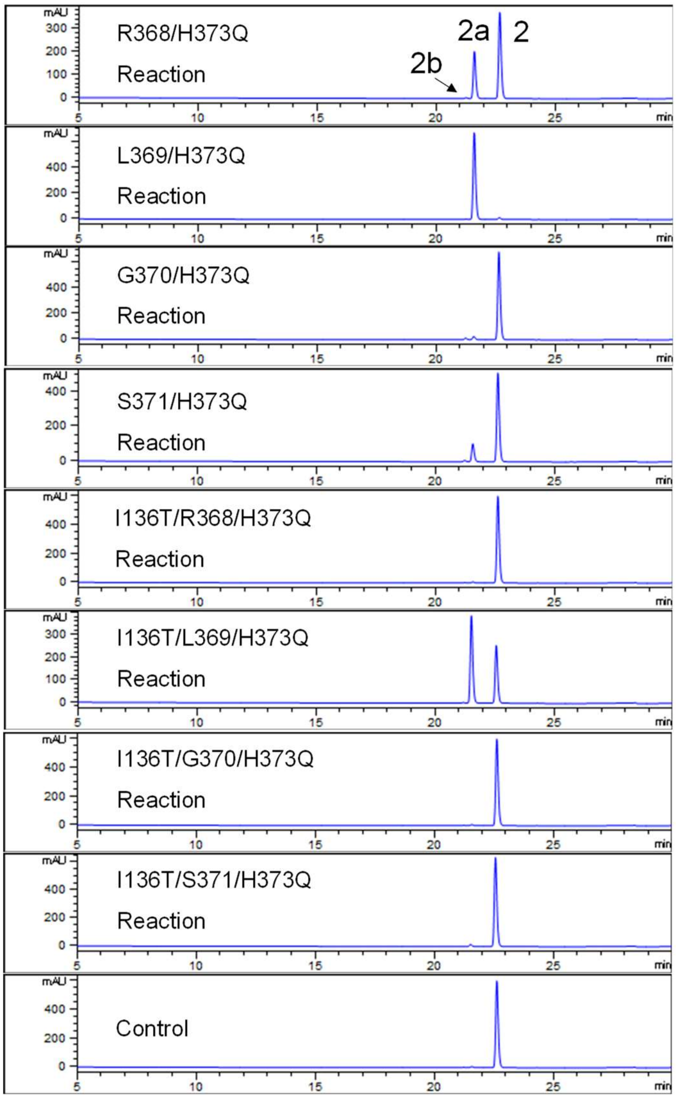

Supplementary Fig. 92 HPLC analysis of mutants catalyzed product using **2** as the substrate and UDP-Api as sugar donor. UDP-Api was produced by adding UDP-GlcA, purified UAXS, and NAD^+ to the mixed system. The analytical conditions are given in **Supplementary Table 3**.

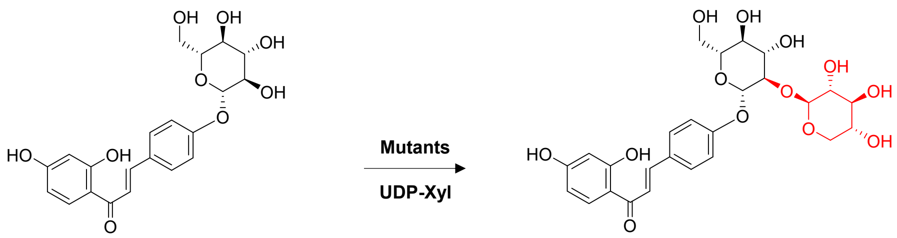

2
MW：418.4

2b
MW：550.5

a

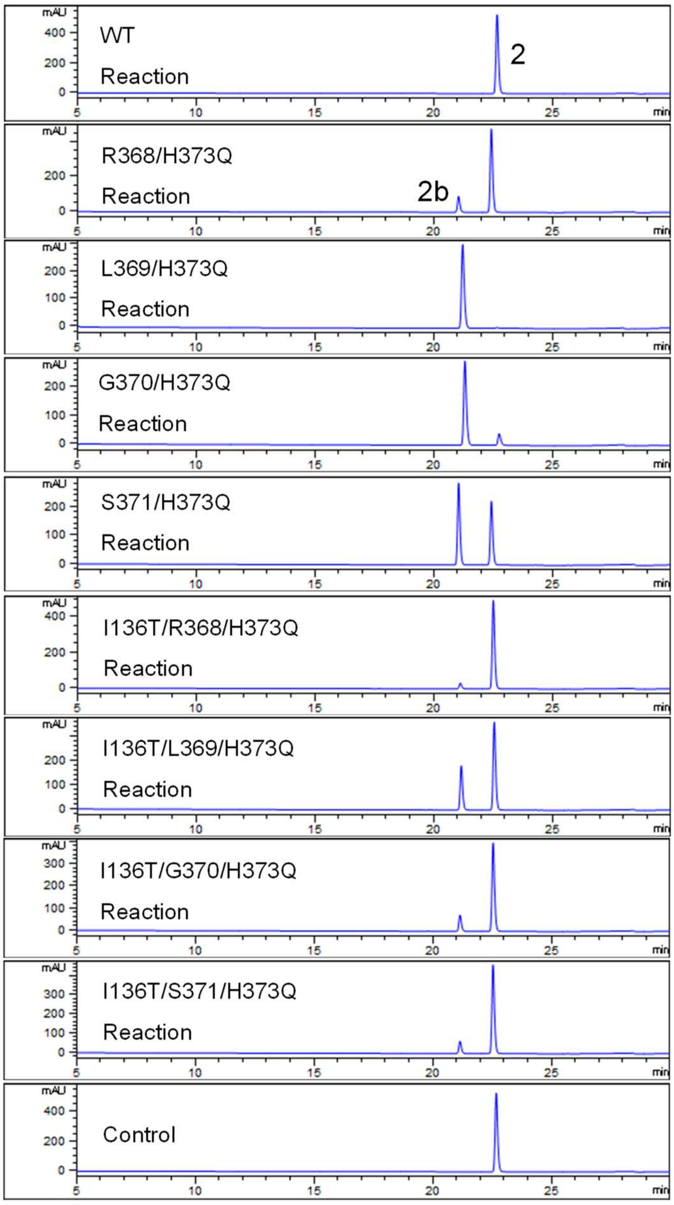

b

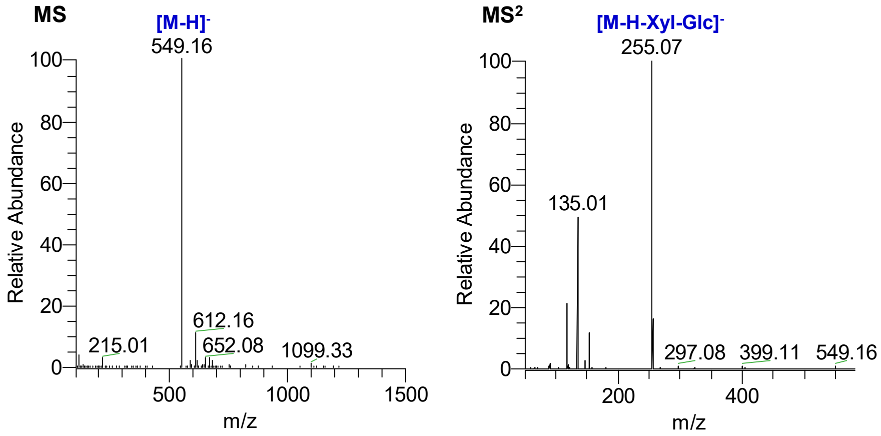

Supplementary Fig. 93 HPLC and LC/MS analyses of mutants catalyzed product using **2** as the substrate and UDP-Xyl as the sugar donor. **a**, HPLC chromatograms. **b**, (-)-ESI-MS and MS^2 spectra of product **2b**. The analytical conditions are given in **Supplementary Table 3**.

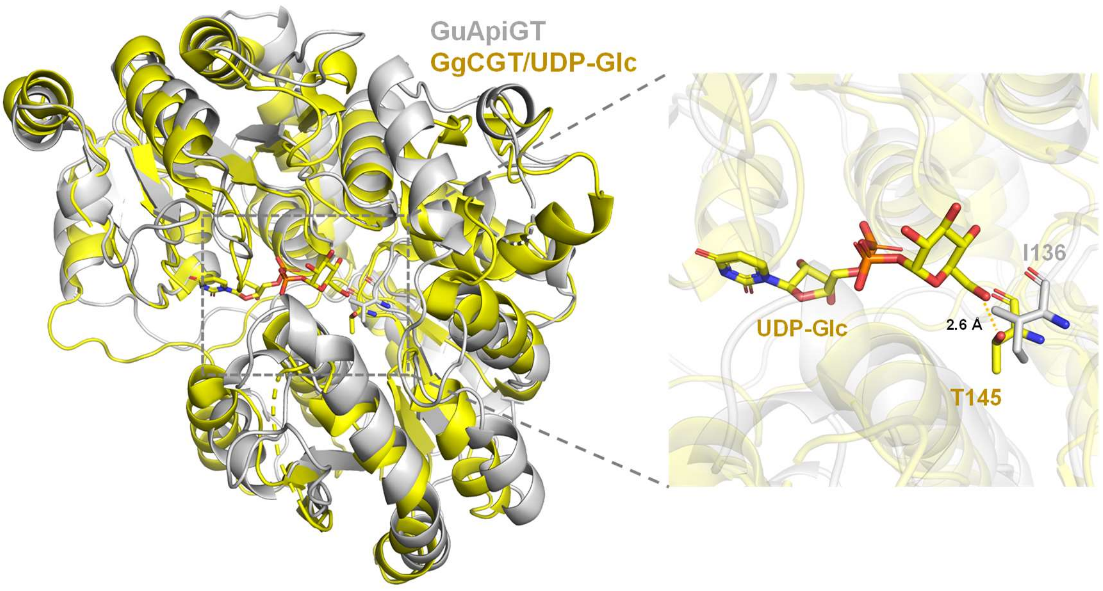

Supplementary Fig. 94 Superimposition of GuApiGT and GgCGT.

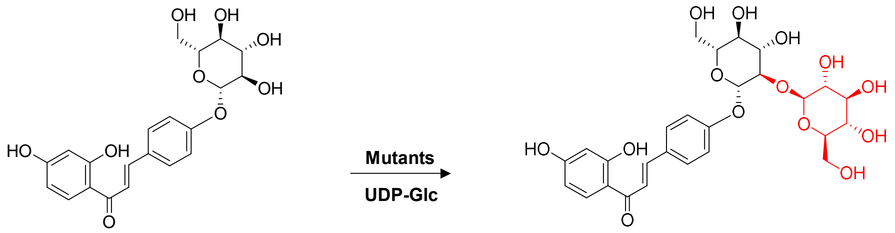

2
MW：418.4

2c
MW：580.5

a

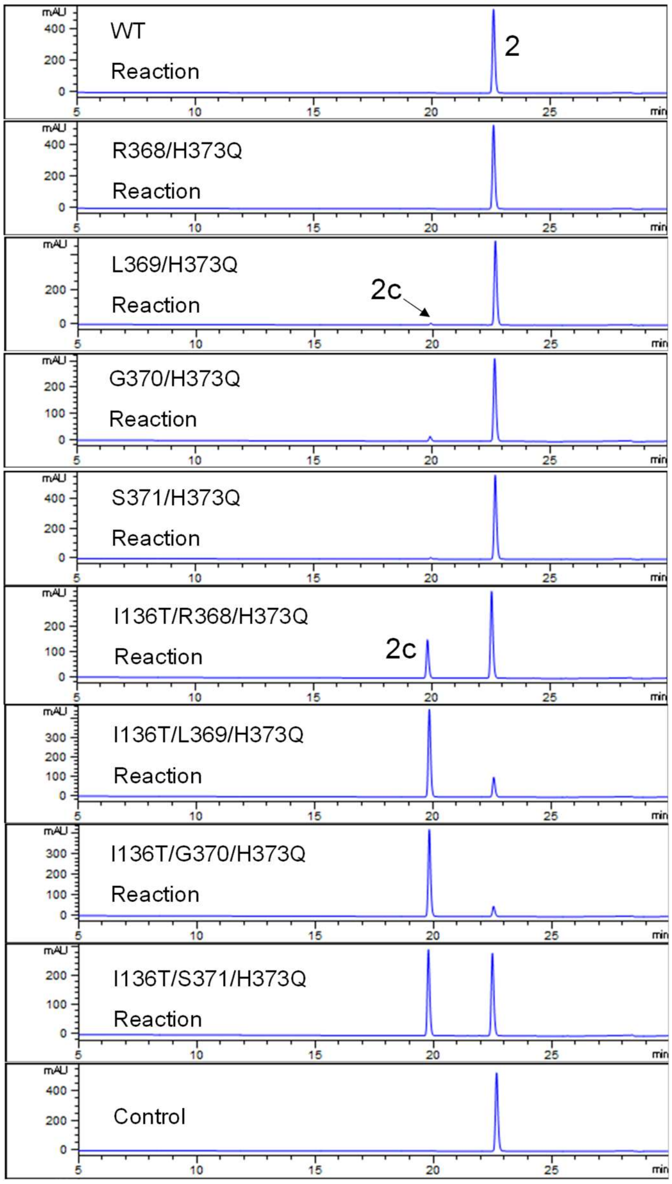

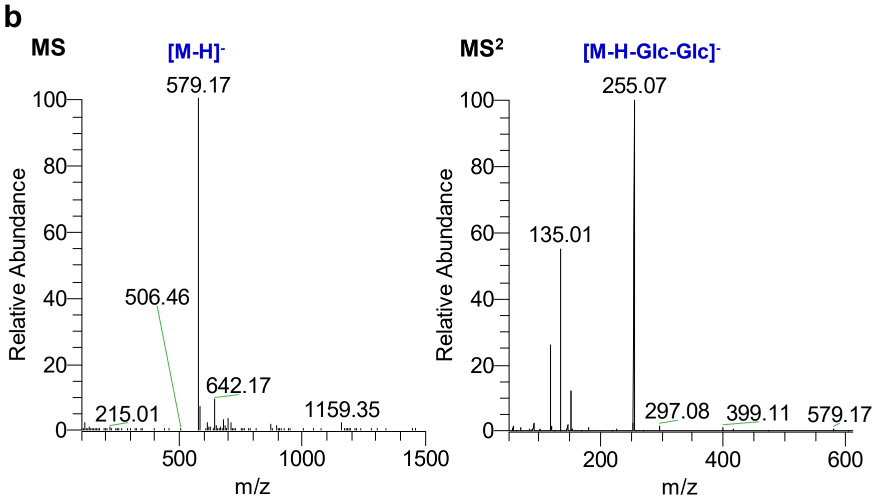

Supplementary Fig. 95 HPLC and LC/MS analyses of mutants catalyzed products using **2** as the substrate and UDP-Glc as sugar donor. **a**, HPLC chromatograms. **b**, (-)-ESI-MS and MS^2 spectra of product **2c**. The analytical conditions are given in **Supplementary Table 3**.

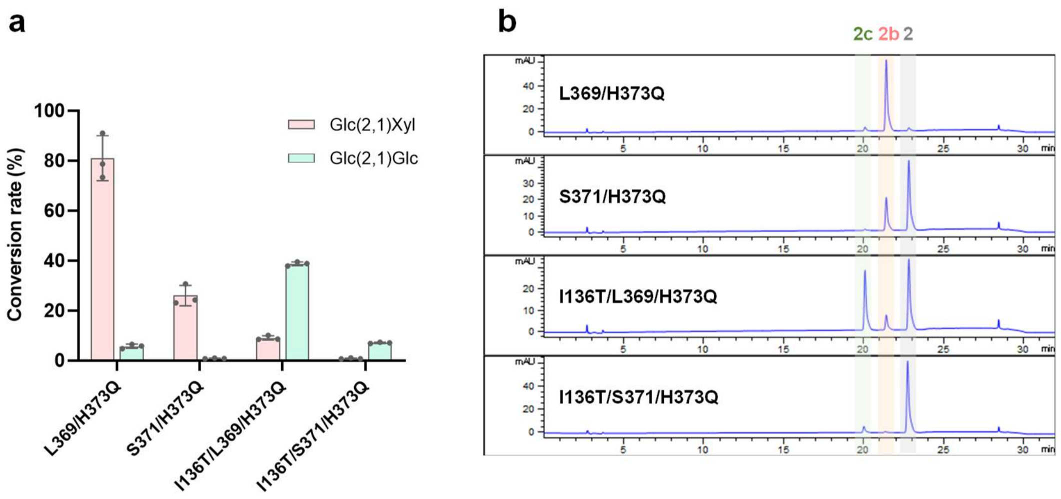

Supplementary Fig. 96 Sugar donor preference of GuApiGT mutants. **a**, conversion rates of four representative mutants. Compound **2** was used as the sugar acceptor, and both UDP-Xyl and UDP-Glc were added into the catalysis system as sugar donor. **b**, HPLC chromatograms of the enzyme catalysis products. For compounds identification of **2b** and **2c**, please see **Supplementary Fig. 93** and **Supplementary Fig. 95**. Data are presented as mean values ± SD (n=3 biologically independent samples) (**a**). The source data underlying figure (**a**) are provided in a Source Data file.

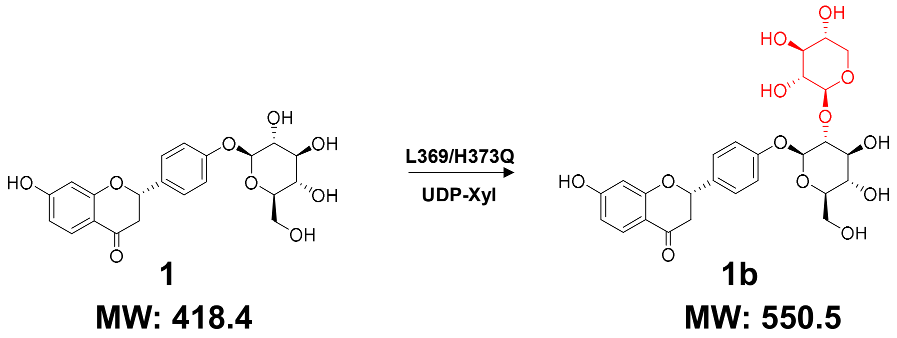

a

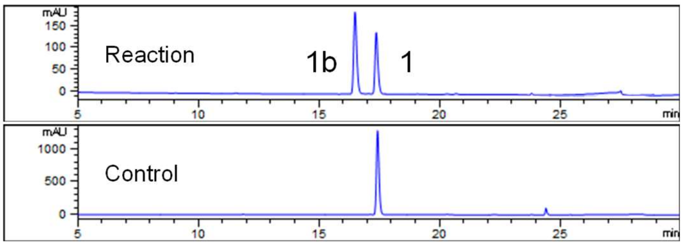

b

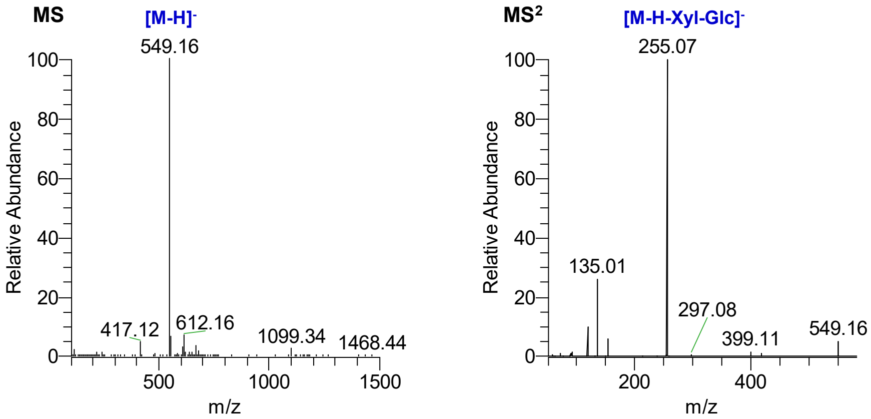

Supplementary Fig. 97 HPLC and LC/MS analyses of L369/H373Q catalytic reaction mixture for substrate **1**. **a**, HPLC analysis of L369/H373Q catalyzed product using **1** as the substrate. **b**, (-)-ESI-MS and MS^2 spectra of product **1b**. The analysis conditions are given in **Supplementary Table 3**.

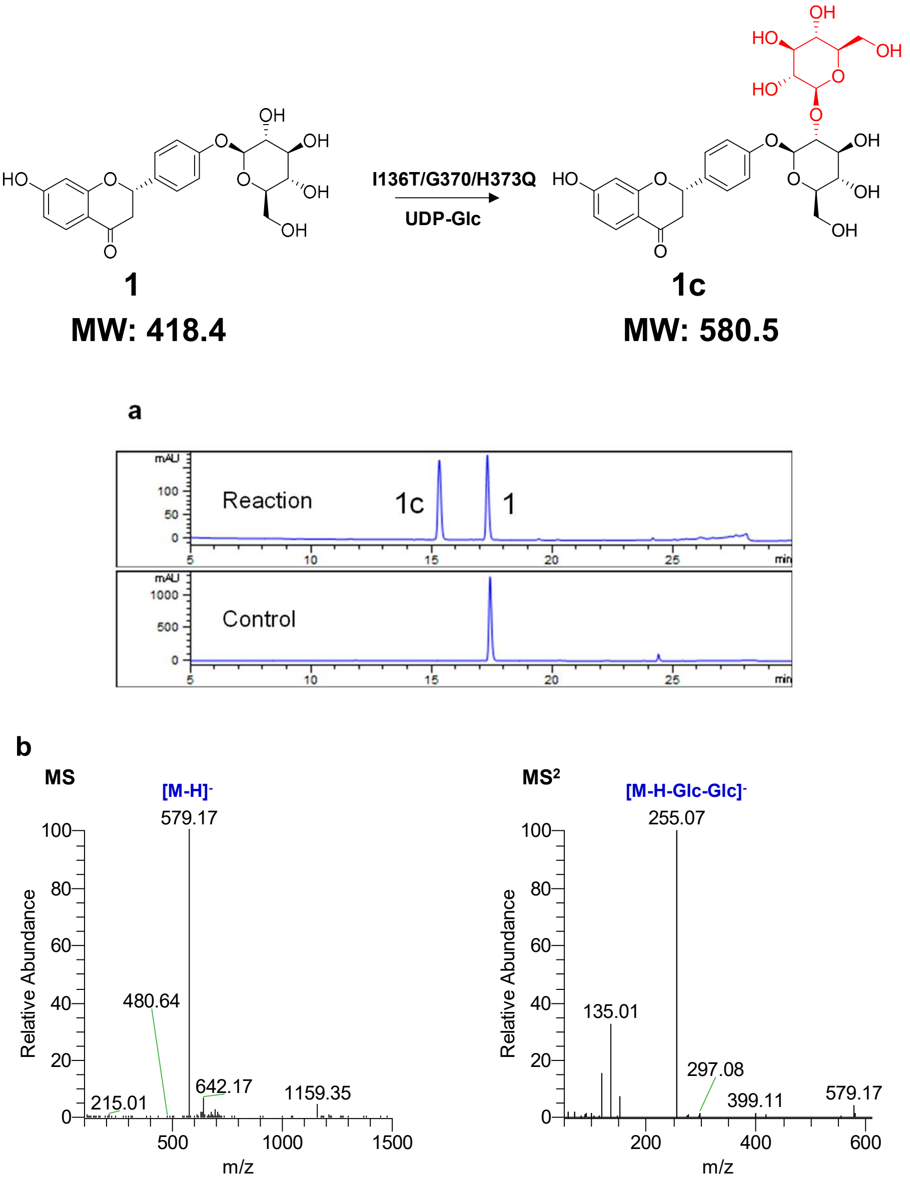

Supplementary Fig. 98 HPLC and LC/MS analyses of I136T/G370/H373Q catalytic reaction mixture for substrate **1**. **a**, HPLC analysis of I136T/G370/H373Q catalyzed product using **1** as the substrate. **b**, (-)-ESI-MS and MS^2 spectra of product **1c**. The analysis conditions are given in **Supplementary Table 3**.

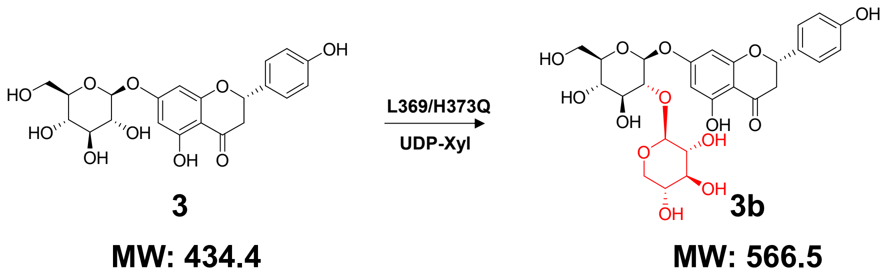

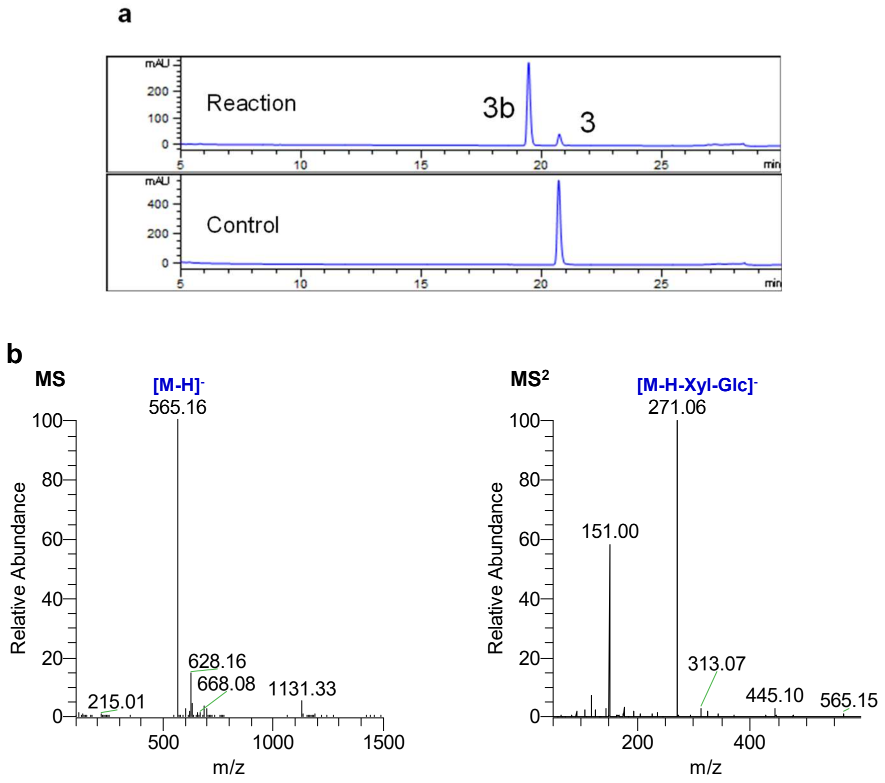

Supplementary Fig. 99 HPLC and LC/MS analyses of L369/H373Q catalytic reaction mixture for substrate **3**. **a**, HPLC analysis of L369/H373Q catalyzed product using **3** as the substrate. **b**, (-)-ESI-MS and MS^2 spectra of product **3b**. The analytical conditions are given in **Supplementary Table 3**.

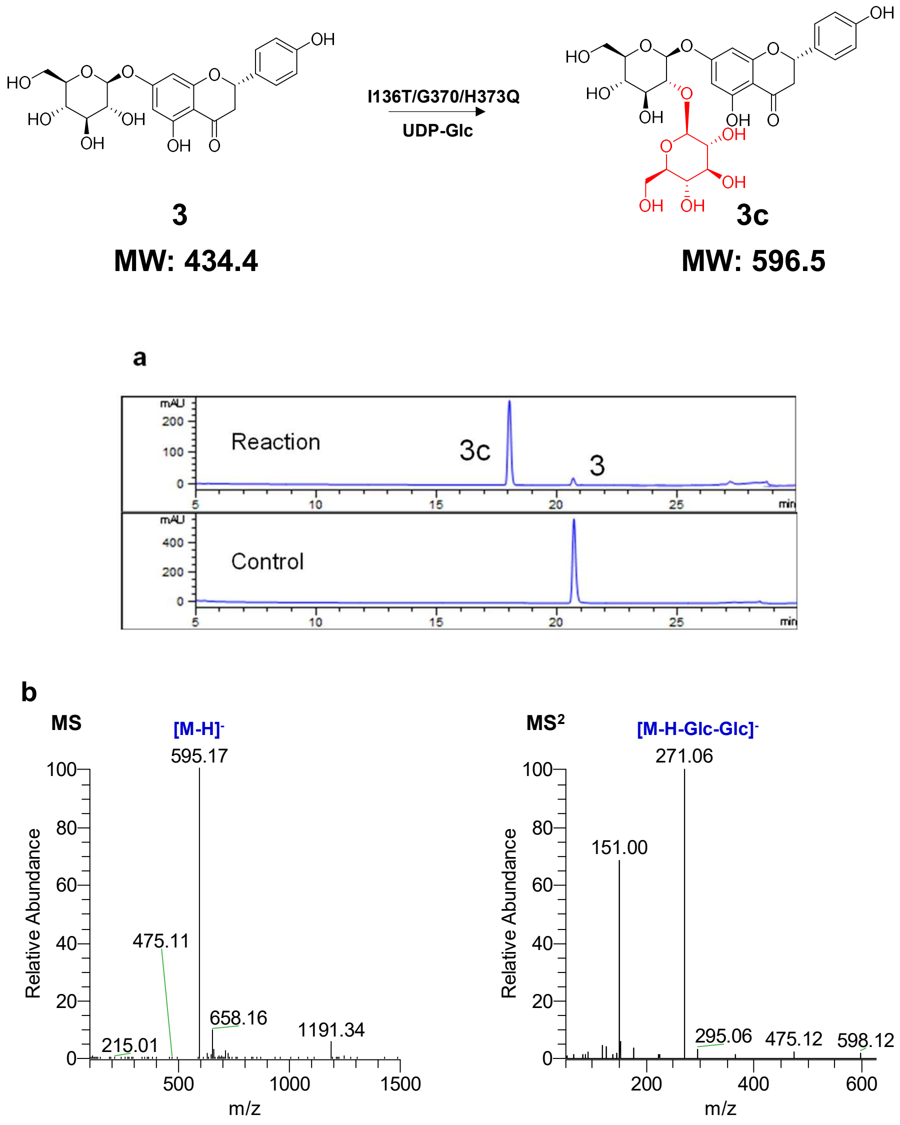

Supplementary Fig. 100 HPLC and LC/MS analyses of I136T/G370/H373Q catalytic reaction mixture for substrate **3**. **a**, HPLC analysis of I136T/G370/H373Q catalyzed product using **3** as the substrate. **b**, (-)-ESI-MS and MS^2 spectra of product **3c**. The analytical conditions are given in **Supplementary Table 3**.

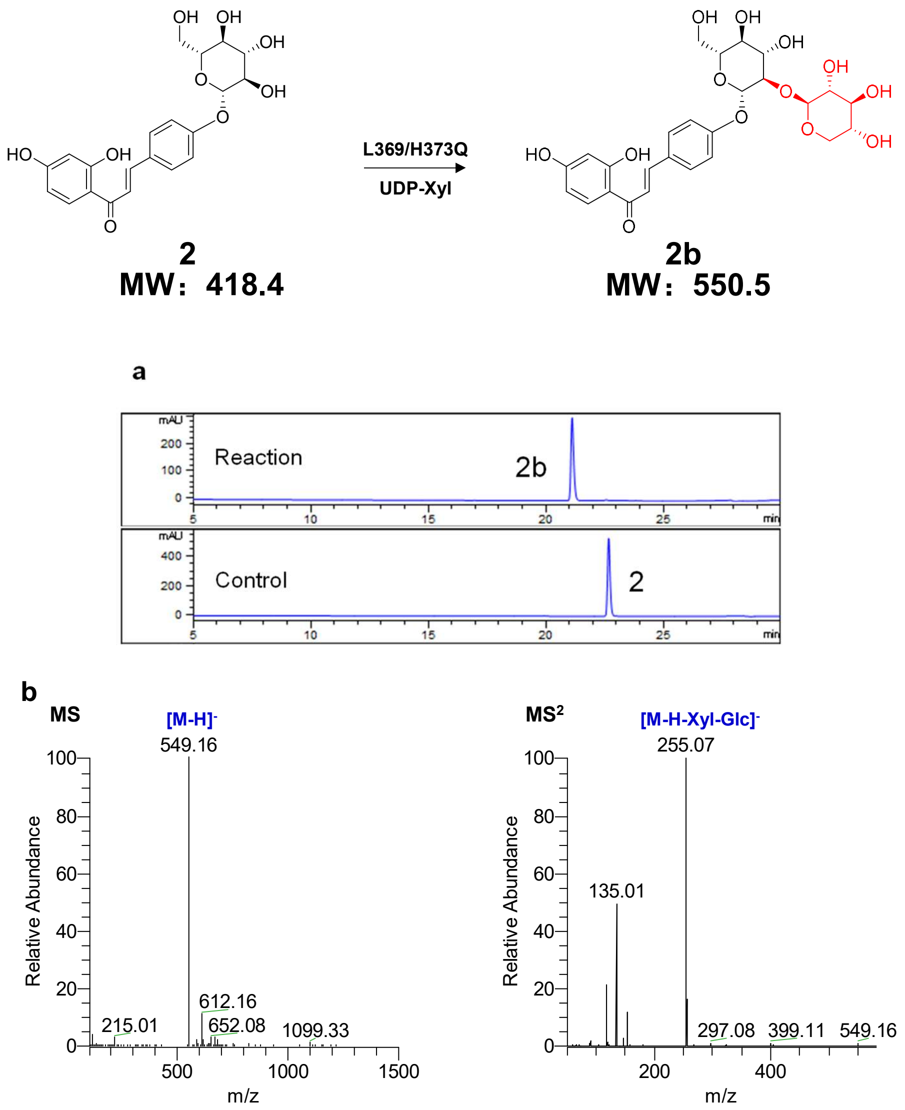

Supplementary Fig. 101 HPLC and LC/MS analyses of L369/H373Q catalytic reaction mixture for substrate **2**. **a**, HPLC analysis of L369/H373Q catalyzed product using **2** as the substrate. **b**, (-)-ESI-MS and MS^2 spectra of product **2b**. The analytical conditions are given in **Supplementary Table 3**.

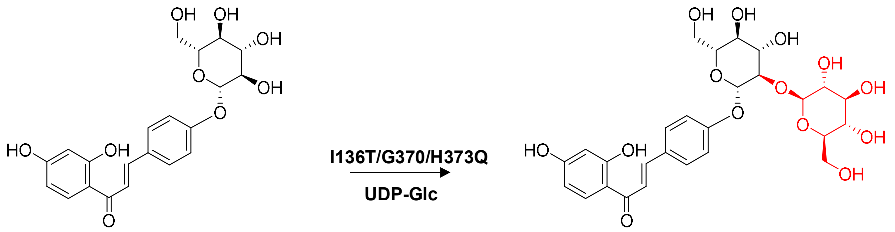

2
MW：418.4

2c
MW：580.5

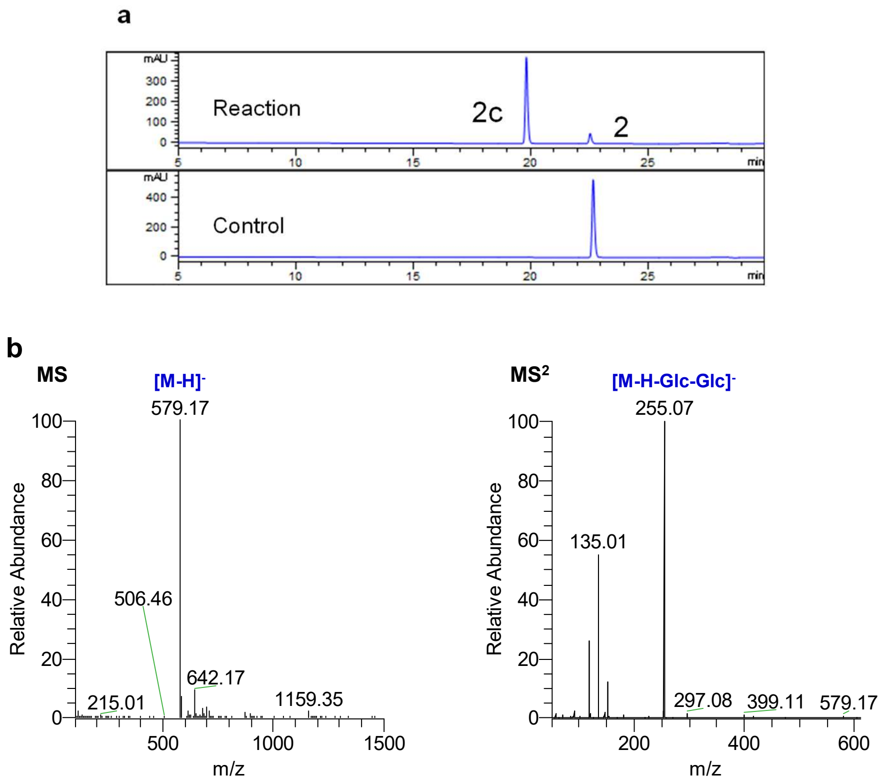

Supplementary Fig. 102 HPLC and LC/MS analyses of I136T/G370/H373Q catalytic reaction mixture for substrate **2**. **a**, HPLC analysis of I136T/G370/H373Q catalyzed product using **2** as the substrate. **b**, (-)-ESI-MS and MS^2 spectra of product **2c**. The analytical conditions are given in **Supplementary Table 3**.

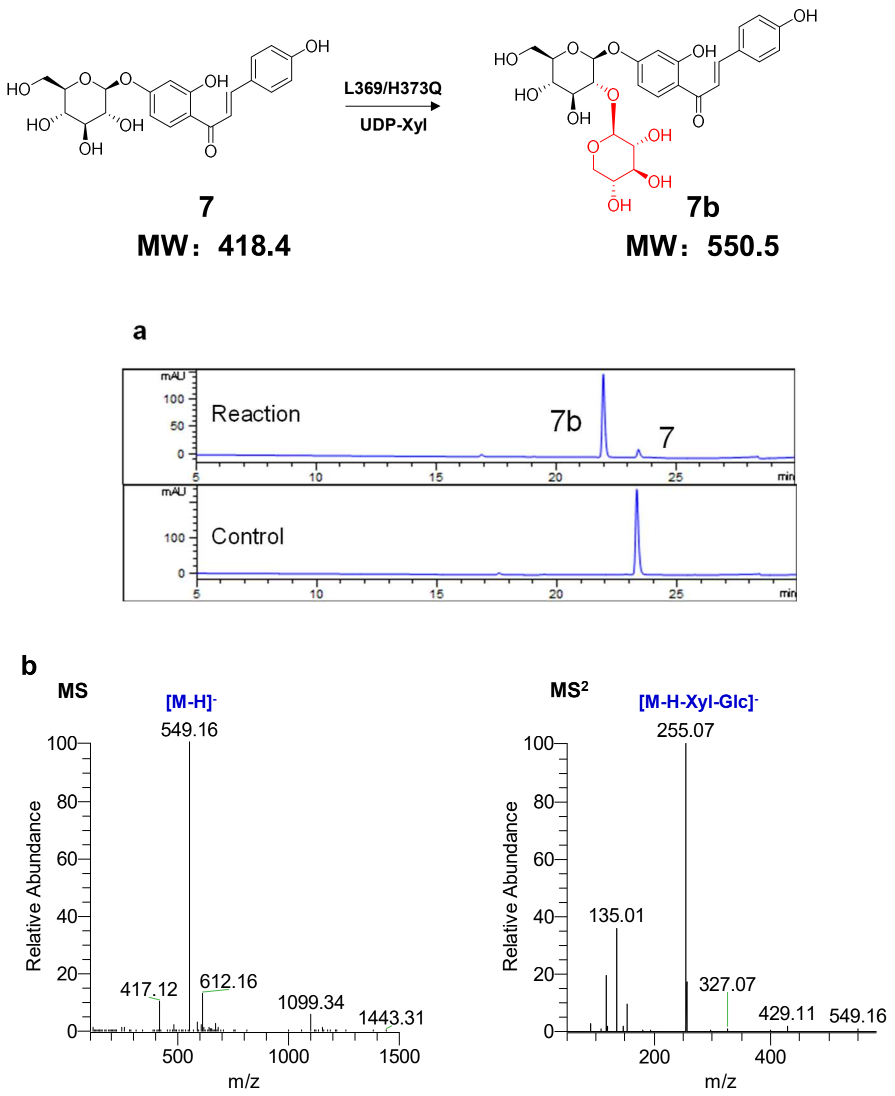

Supplementary Fig. 103 HPLC and LC/MS analyses of L369/H373Q catalytic reaction mixture for substrate **7**. **a**, HPLC analysis of L369/H373Q catalyzed product using **7** as the substrate. **b**, (-)-ESI-MS and MS^2 spectra of product **7b**. The analytical conditions are given in **Supplementary Table 3**.

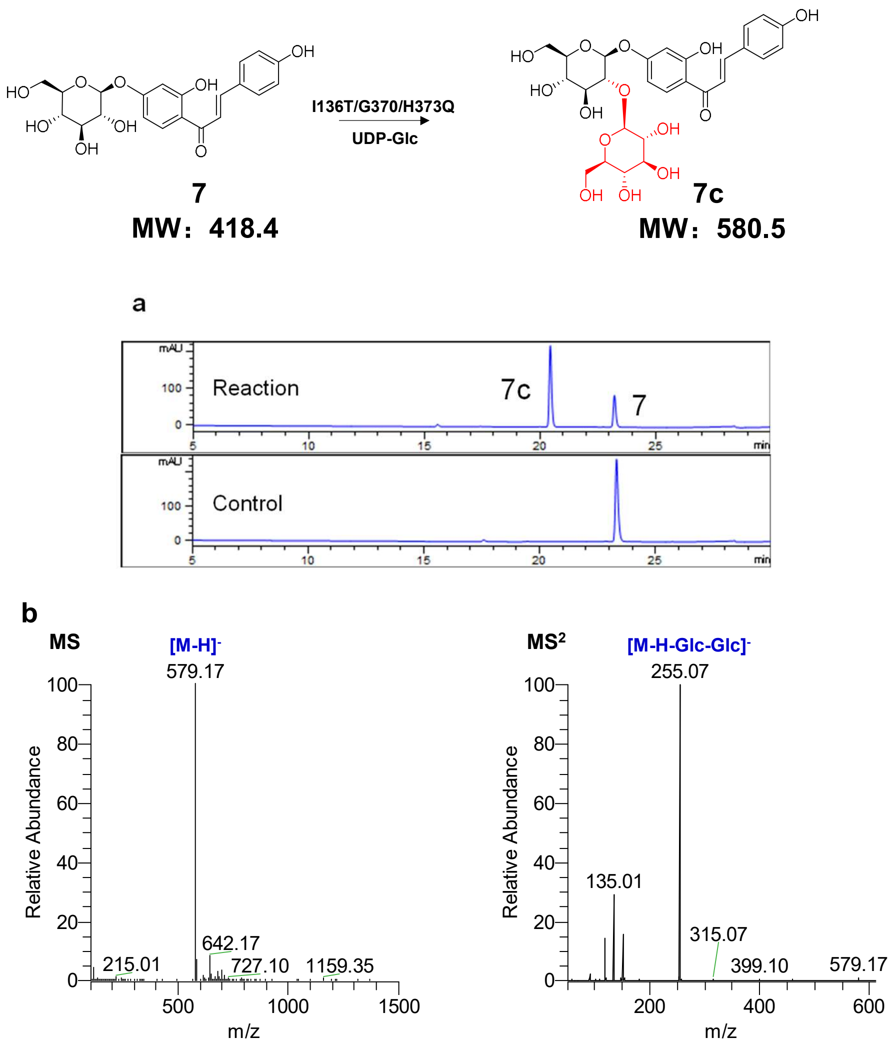

Supplementary Fig. 104 HPLC and LC/MS analyses of I136T/G370/H373Q catalytic reaction mixture for substrate **7**. **a**, HPLC analysis of I136T/G370/H373Q catalyzed product using **7** as the substrate. **b**, (-)-ESI-MS and MS^2 spectra of product **7c**. The analytical conditions are given in **Supplementary Table 3**.

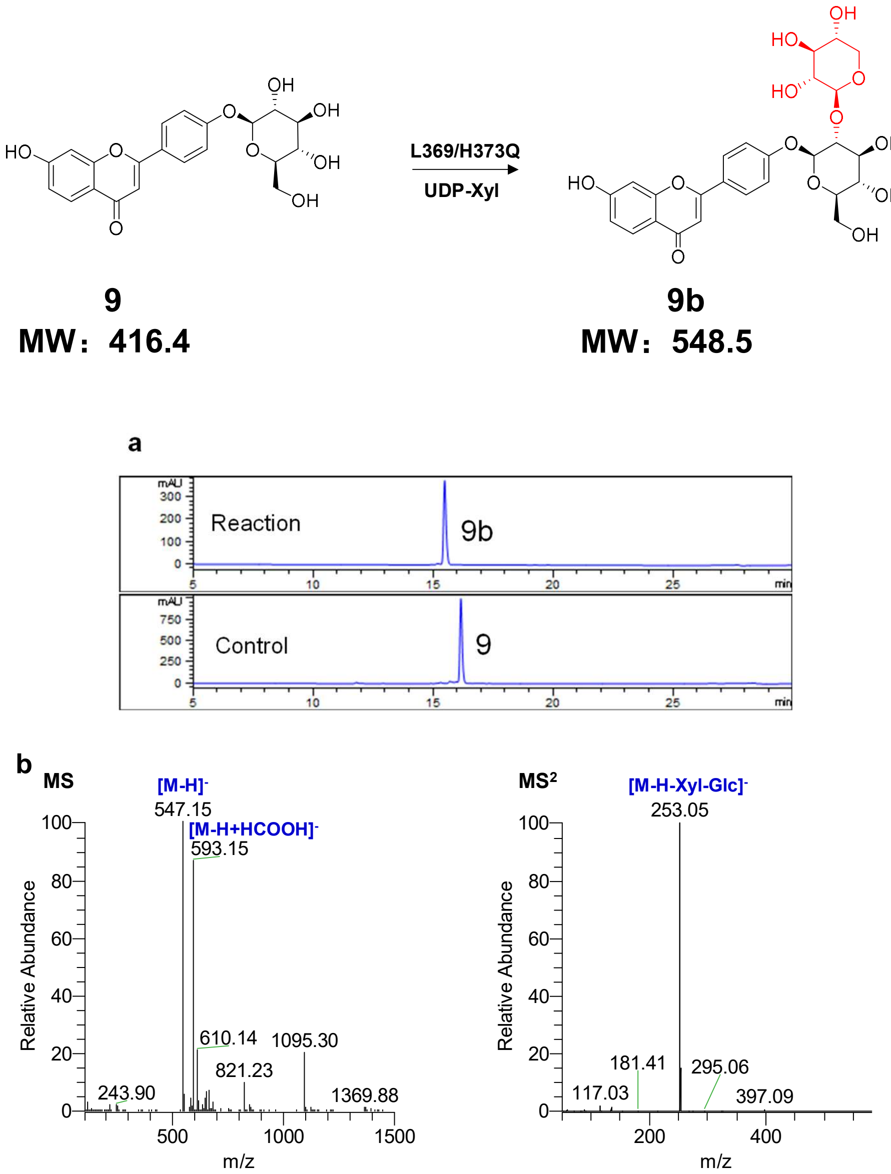

Supplementary Fig. 105 HPLC and LC/MS analyses of L369/H373Q catalytic reaction mixture for substrate **9**. **a**, HPLC analysis of L369/H373Q catalyzed product using **9** as the substrate. **b**, (-)-ESI-MS and MS^2 spectra of product **9b**. The analytical conditions are given in **Supplementary Table 3**.

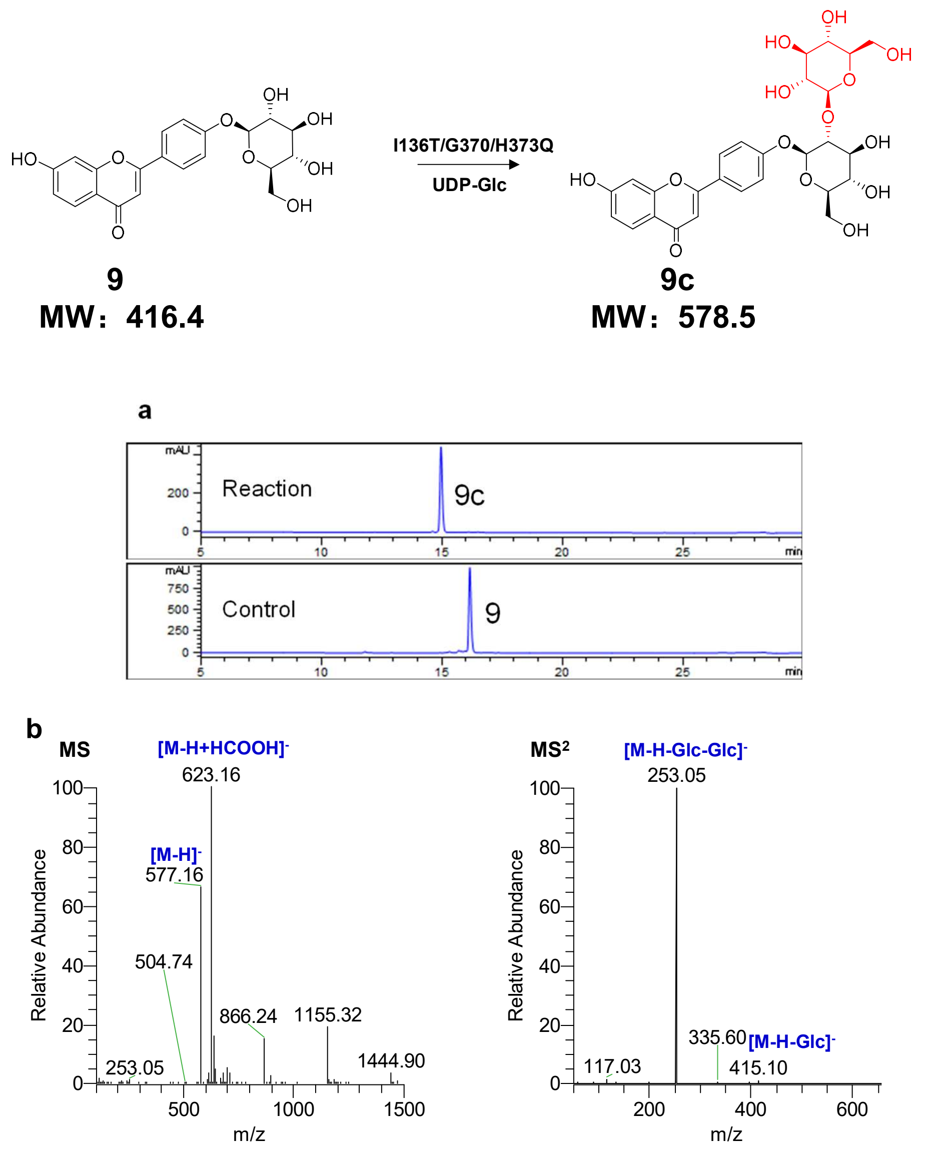

Supplementary Fig. 106 HPLC and LC/MS analyses of I136T/G370/H373Q catalytic reaction mixture for substrate **9**. **a**, HPLC analysis of I136T/G370/H373Q catalyzed product using **9** as the substrate. **b**, (-)-ESI-MS and MS^2 spectra of product **9c**. The analytical conditions are given in **Supplementary Table 3**.

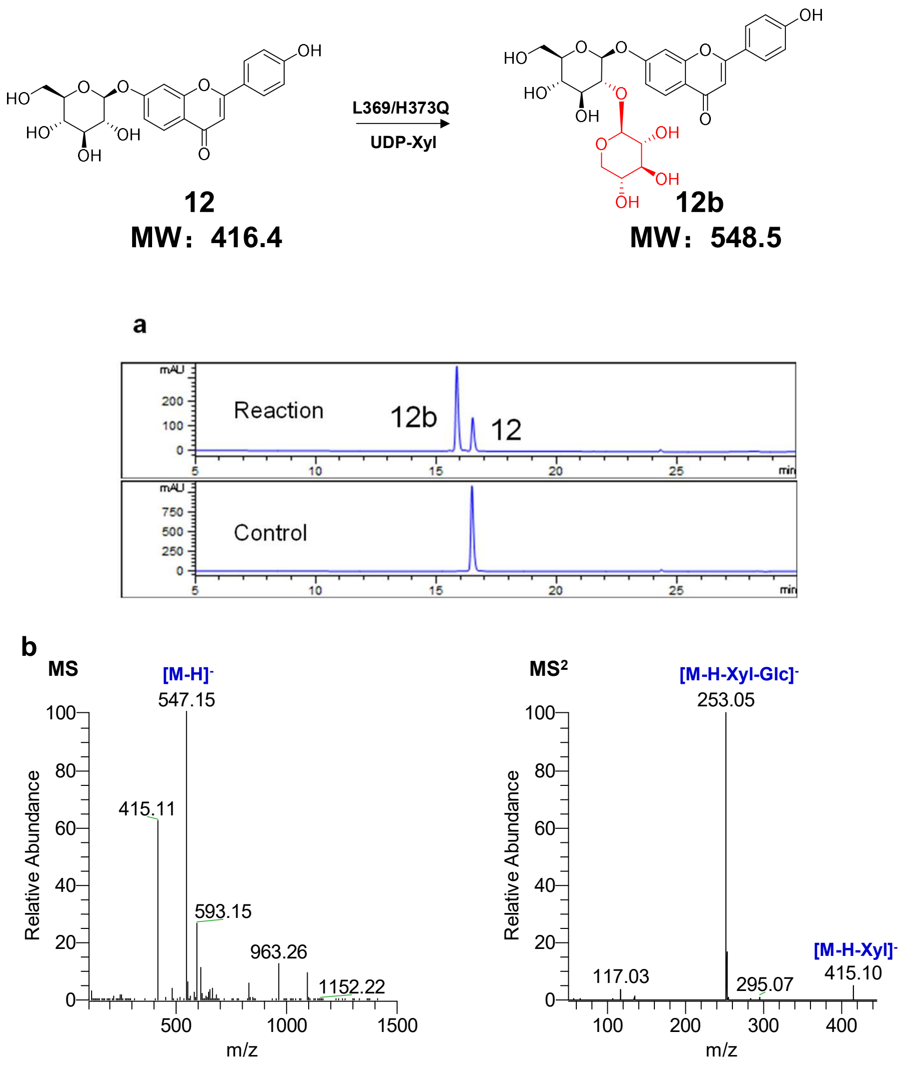

Supplementary Fig. 107 HPLC and LC/MS analyses of L369/H373Q catalytic reaction mixture for substrate **12**. **a**, HPLC analysis of L369/H373Q catalyzed product using **12** as the substrate. **b**, (-)-ESI-MS and MS^2 spectra of product **12b**. The analytical conditions are given in **Supplementary Table 3**.

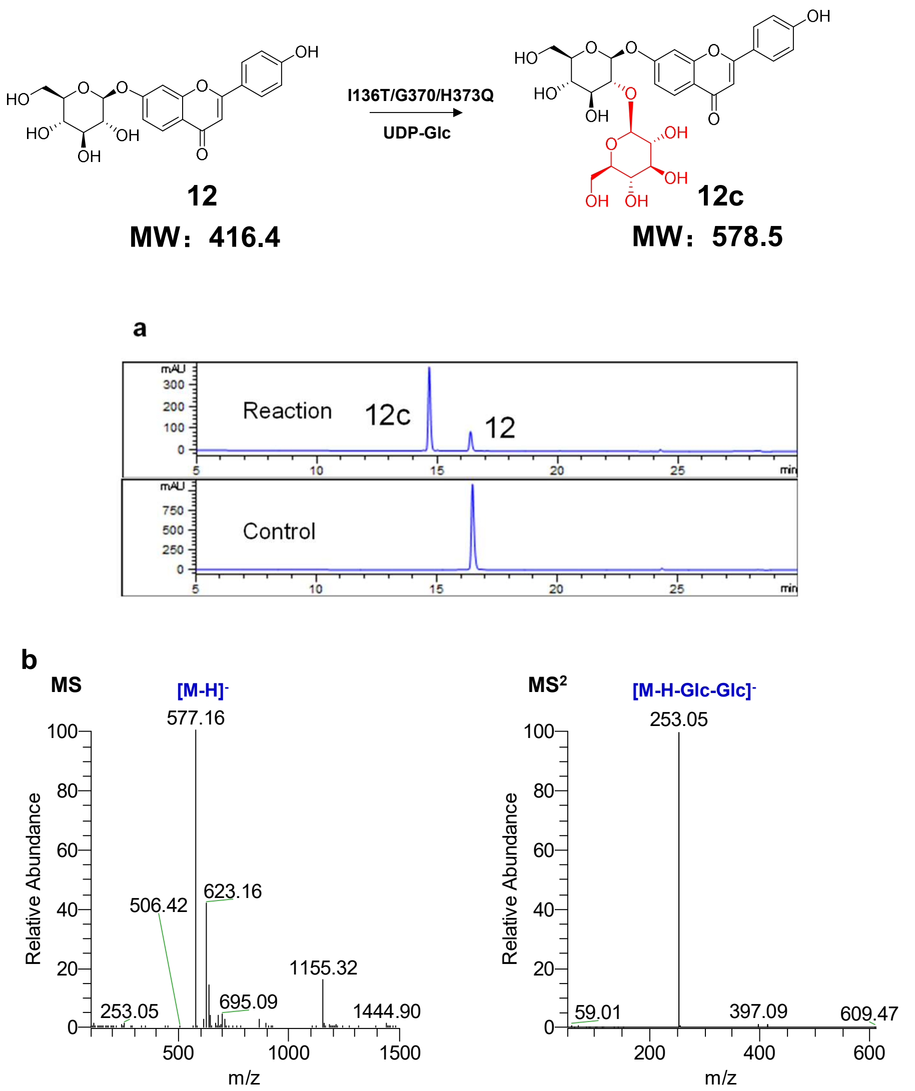

Supplementary Fig. 108 HPLC and LC/MS analyses of I136T/G370/H373Q catalytic reaction mixture for substrate **12**. **a**, HPLC analysis of I136T/G370/H373Q catalyzed product using **12** as the substrate. **b**, (-)-ESI-MS and MS^2 spectra of product **12c**. The analytical conditions are given in **Supplementary Table 3**.

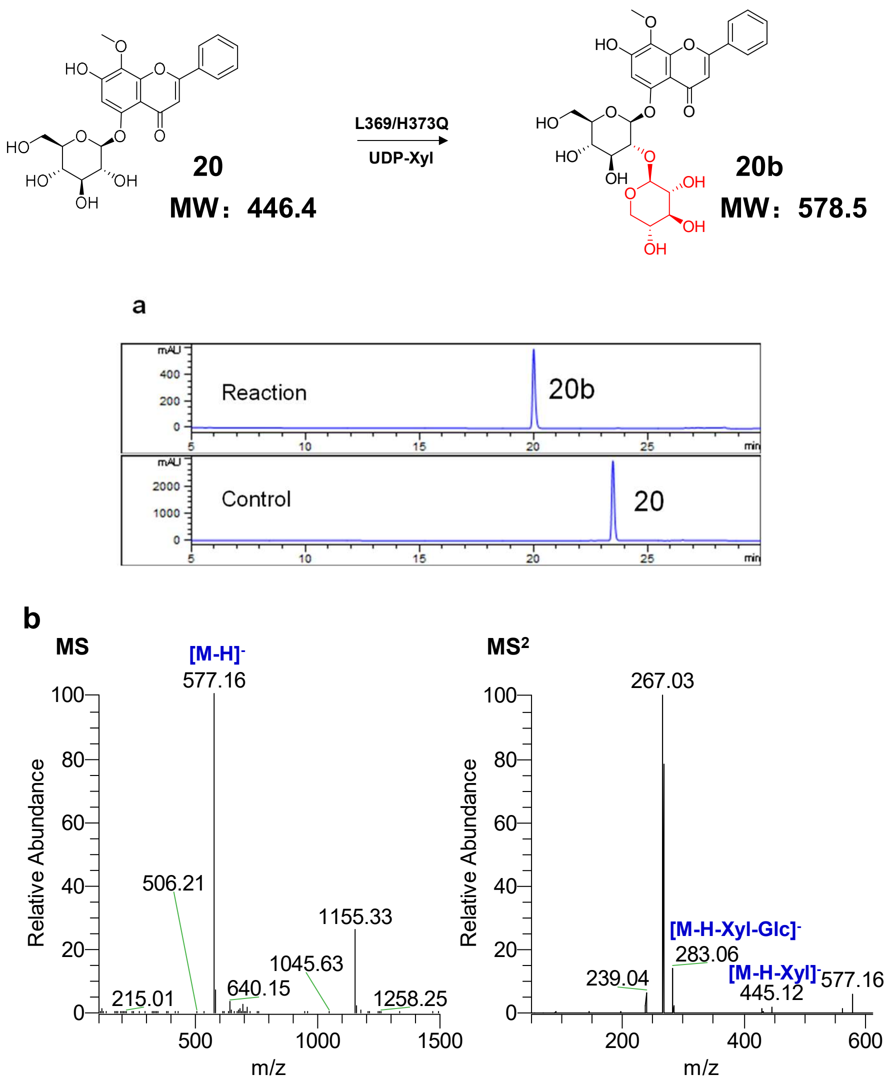

Supplementary Fig. 109 HPLC and LC/MS analyses of L369/H373Q catalytic reaction mixture for substrate **20**. **a**, HPLC analysis of L369/H373Q catalyzed product using **20** as the substrate. **b**, (-)-ESI-MS and MS^2 spectra of product **20b**. The analytical conditions are given in **Supplementary Table 3**.

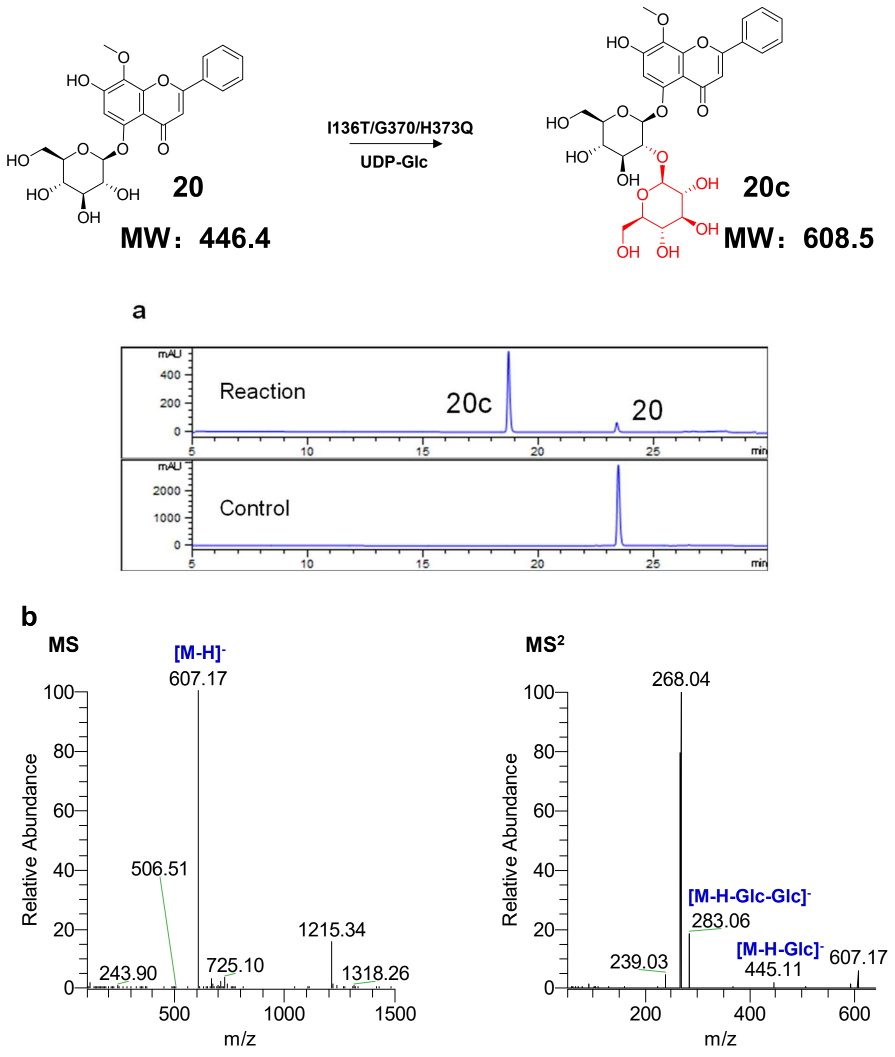

Supplementary Fig. 110 HPLC and LC/MS analyses of I136T/G370/H373Q catalytic reaction mixture for substrate **20**. **a**, HPLC analysis of I136T/G370/H373Q catalyzed product using **20** as the substrate. **b**, (-)-ESI-MS and MS^2 spectra of product **20c**. The analytical conditions are given in **Supplementary Table 3**.

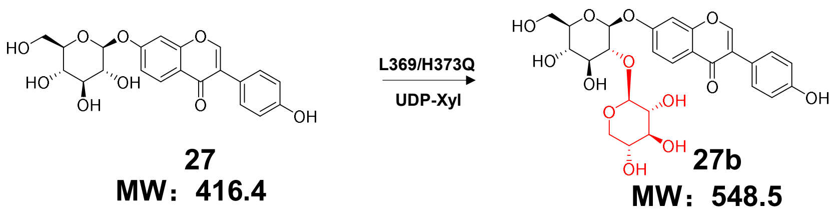

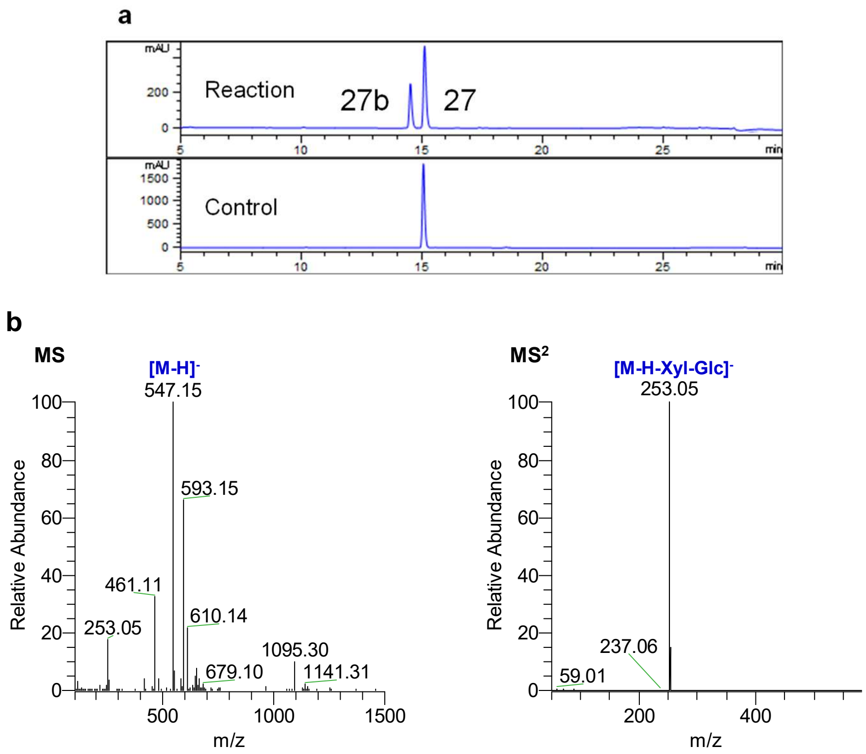

Supplementary Fig. 111 HPLC and LC/MS analyses of L369/H373Q catalytic reaction mixture for substrate **27**. **a**, HPLC analysis of L369/H373Q catalyzed product using **27** as the substrate. **b**, (-)-ESI-MS and MS^2 spectra of product **27b**. The analytical conditions are given in **Supplementary Table 3**.

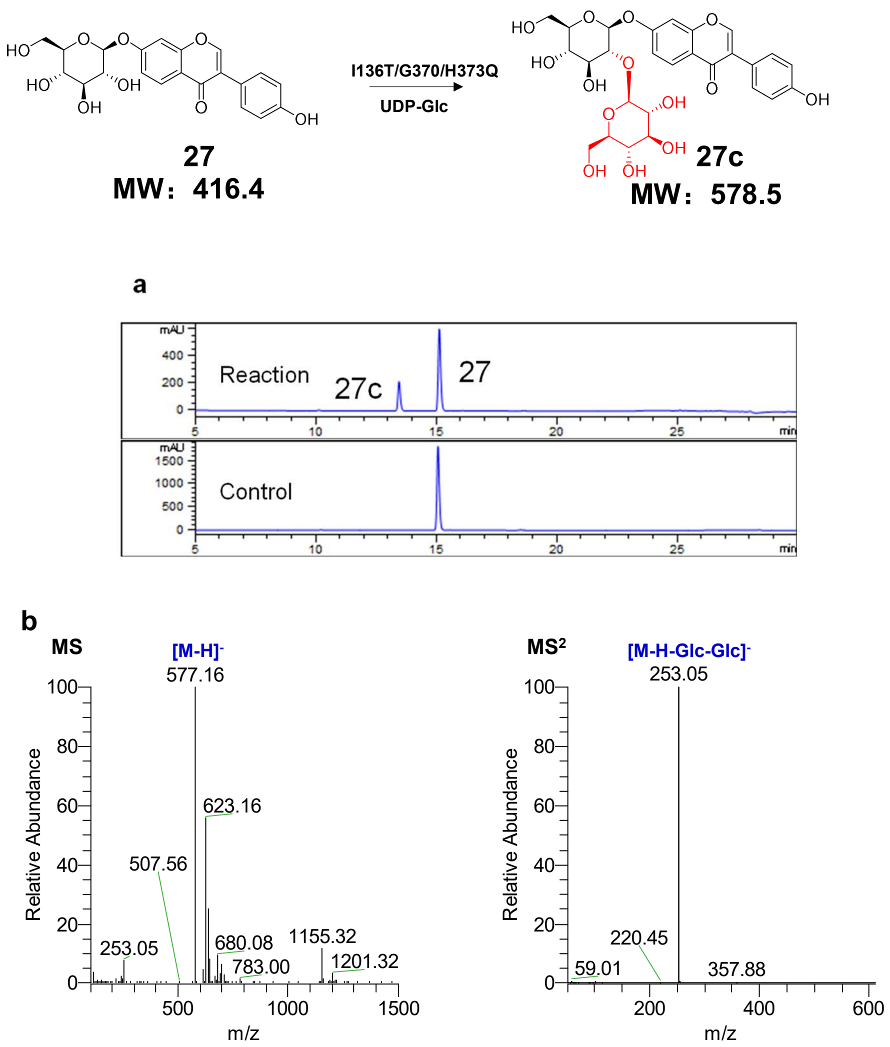

Supplementary Fig. 112 HPLC and LC/MS analyses of I136T/G370/H373Q catalytic reaction mixture for substrate **27**. **a**, HPLC analysis of I136T/G370/H373Q catalyzed product using **27** as the substrate. **b**, (-)-ESI-MS and MS^2 spectra of product **27c**. The analytical conditions are given in **Supplementary Table 3**.

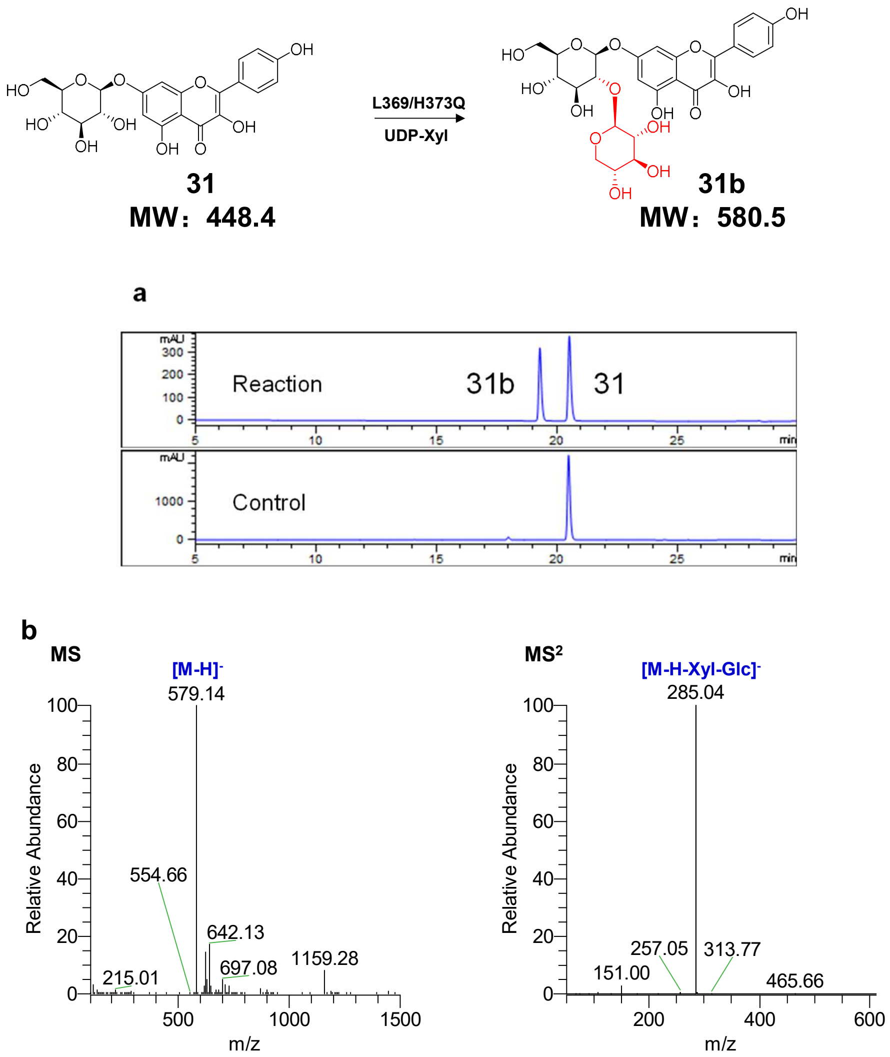

Supplementary Fig. 113 HPLC and LC/MS analyses of L369/H373Q catalytic reaction mixture for substrate **31**. **a**, HPLC analysis of L369/H373Q catalyzed product using **31** as the substrate. **b**, (-)-ESI-MS and MS^2 spectra of product **31b**. The analytical conditions are given in **Supplementary Table 3**.

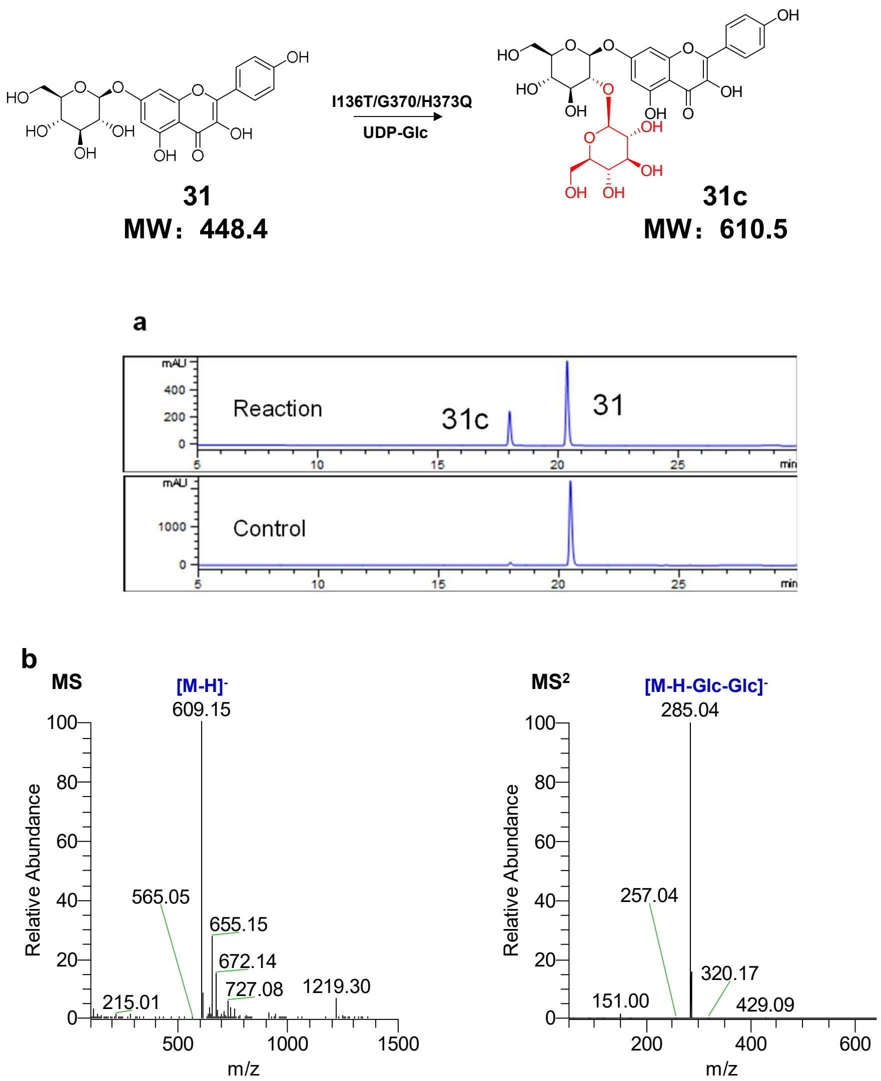

Supplementary Fig. 114 HPLC and LC/MS analyses of I136T/G370/H373Q catalytic reaction mixture for substrate **31**. **a**, HPLC analysis of I136T/G370/H373Q catalyzed product using **31** as the substrate. **b**, (-)-ESI-MS and MS^2 spectra of product **31c**. The analytical conditions are given in **Supplementary Table 3**.

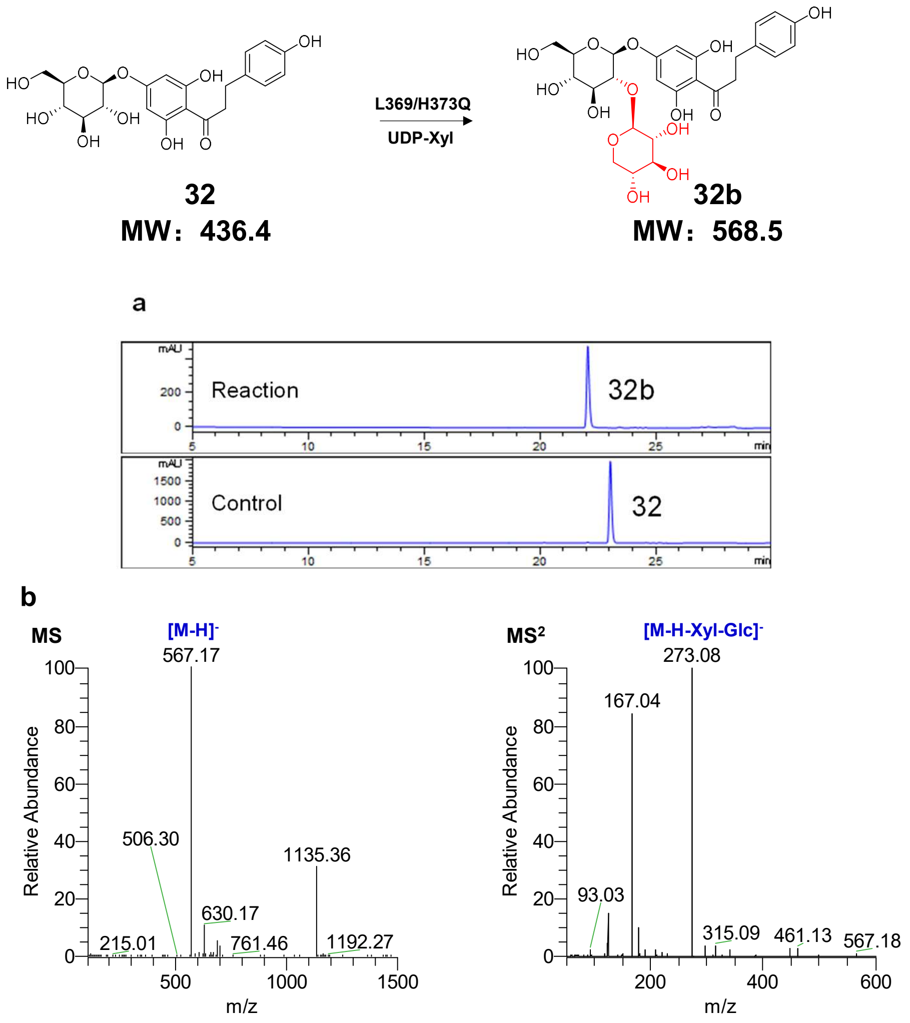

Supplementary Fig. 115 HPLC and LC/MS analyses of L369/H373Q catalytic reaction mixture for substrate **32**. **a**, HPLC analysis of L369/H373Q catalyzed product using **32** as the substrate. **b**, (-)-ESI-MS and MS^2 spectra of product **32b**. The analytical conditions are given in **Supplementary Table 3**.

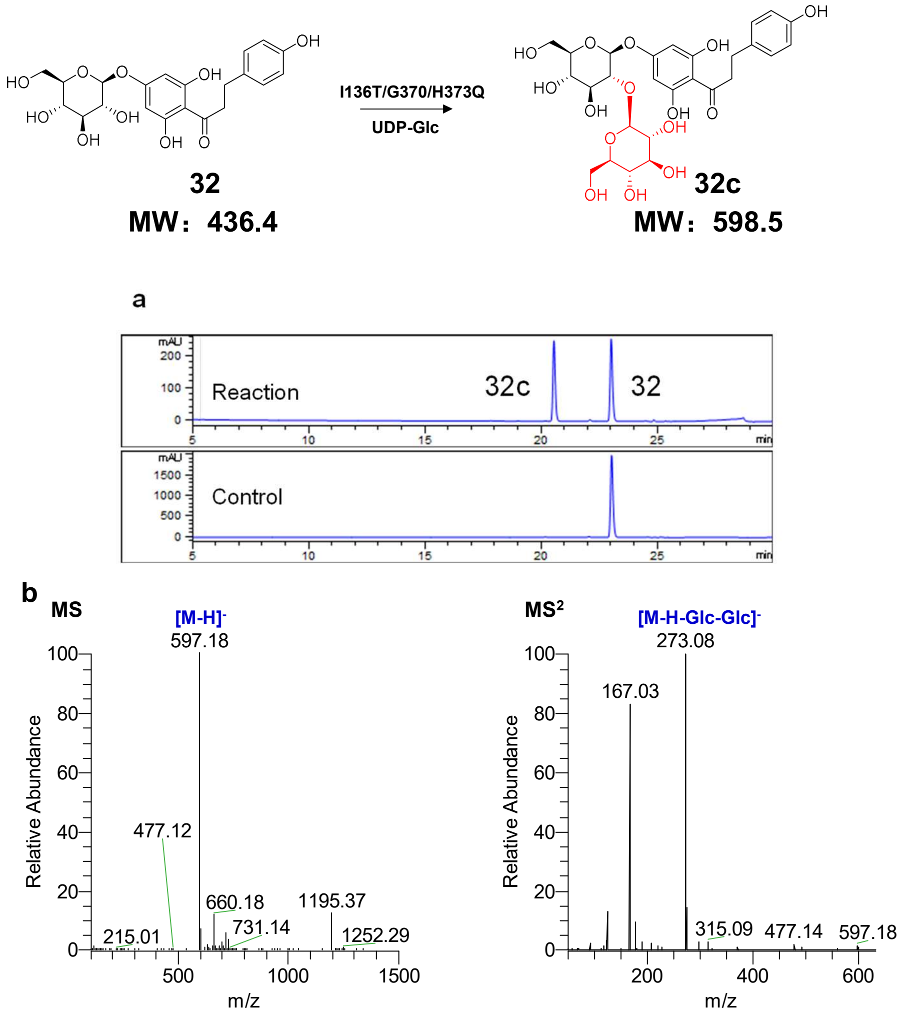

Supplementary Fig. 116 HPLC and LC/MS analyses of I136T/G370/H373Q catalytic reaction mixture for substrate **32**. **a**, HPLC analysis of I136T/G370/H373Q catalyzed product using **32** as the substrate. **b**, (-)-ESI-MS and MS^2 spectra of product **32c**. The analytical conditions are given in **Supplementary Table 3**.

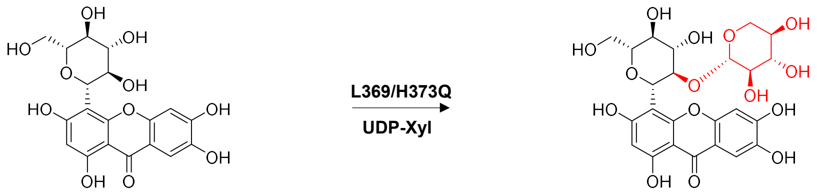

34
MW：422.3

34b
MW：538.5

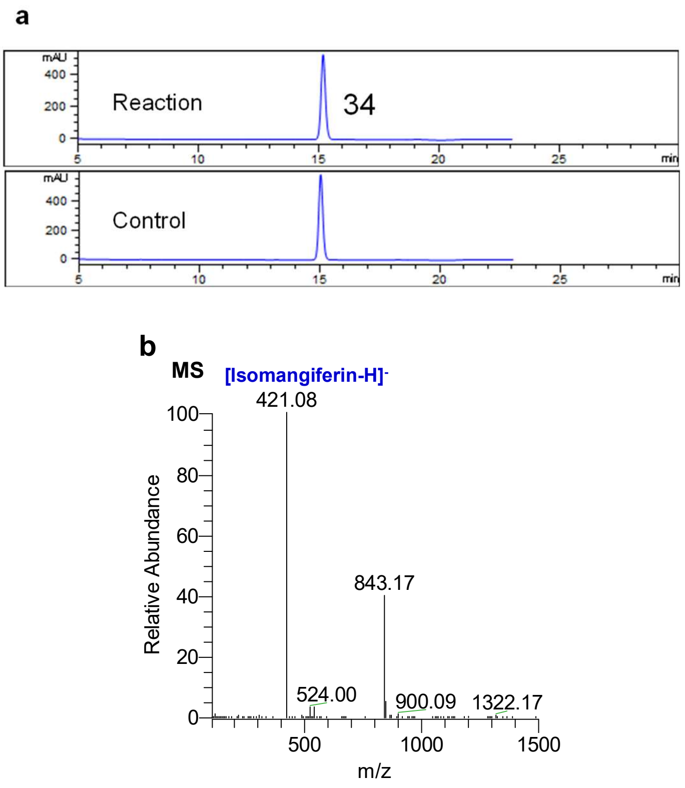

Supplementary Fig. 117 HPLC and LC/MS analyses of L369/H373Q catalytic reaction mixture for substrate **34**. **a**, HPLC analysis of L369/H373Q catalyzed product using **34** as the substrate. **b**, (-)-ESI-MS and MS^2 spectra of product **34b**. The analytical conditions are given in **Supplementary Table 3**.

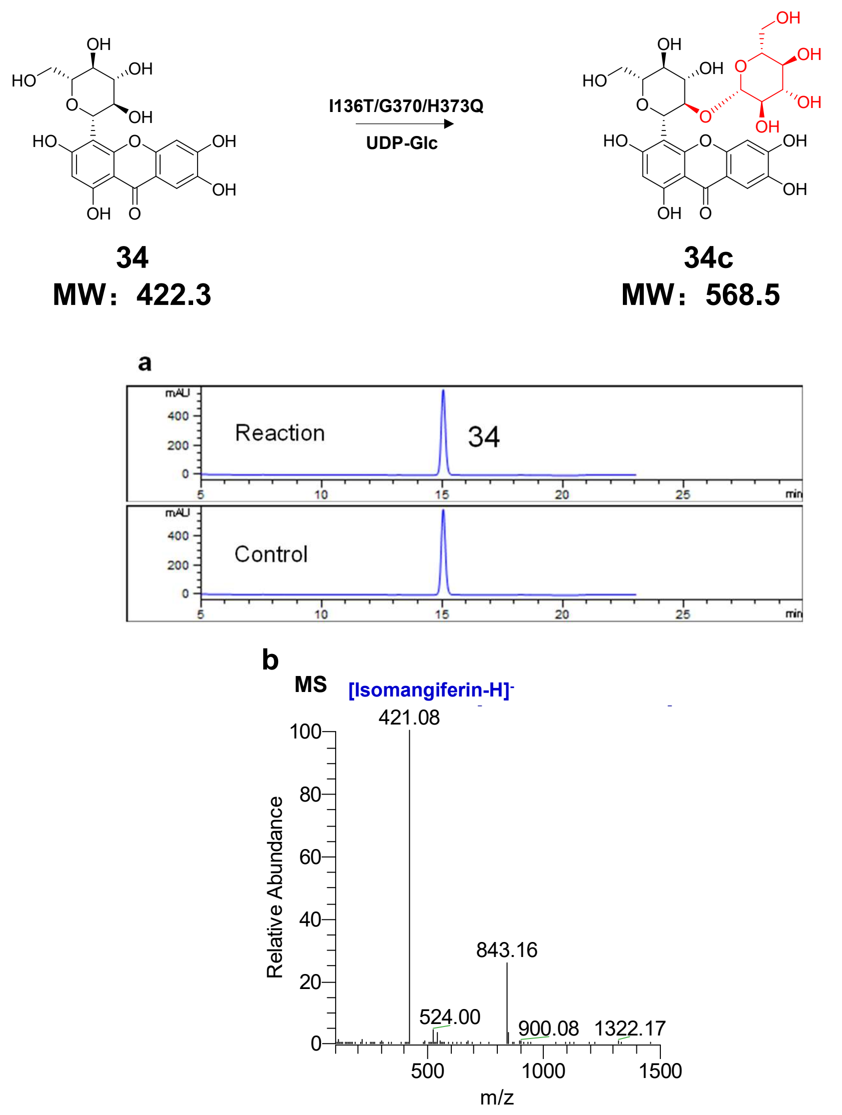

Supplementary Fig. 118 HPLC and LC/MS analyses of I136T/G370/H373Q catalytic reaction mixture for substrate **34**. **a**, HPLC analysis of I136T/G370/H373Q catalyzed product using **34** as the substrate. **b**, (-)-ESI-MS and MS^2 spectra of product **34c**. The analytical conditions are given in **Supplementary Table 3**.

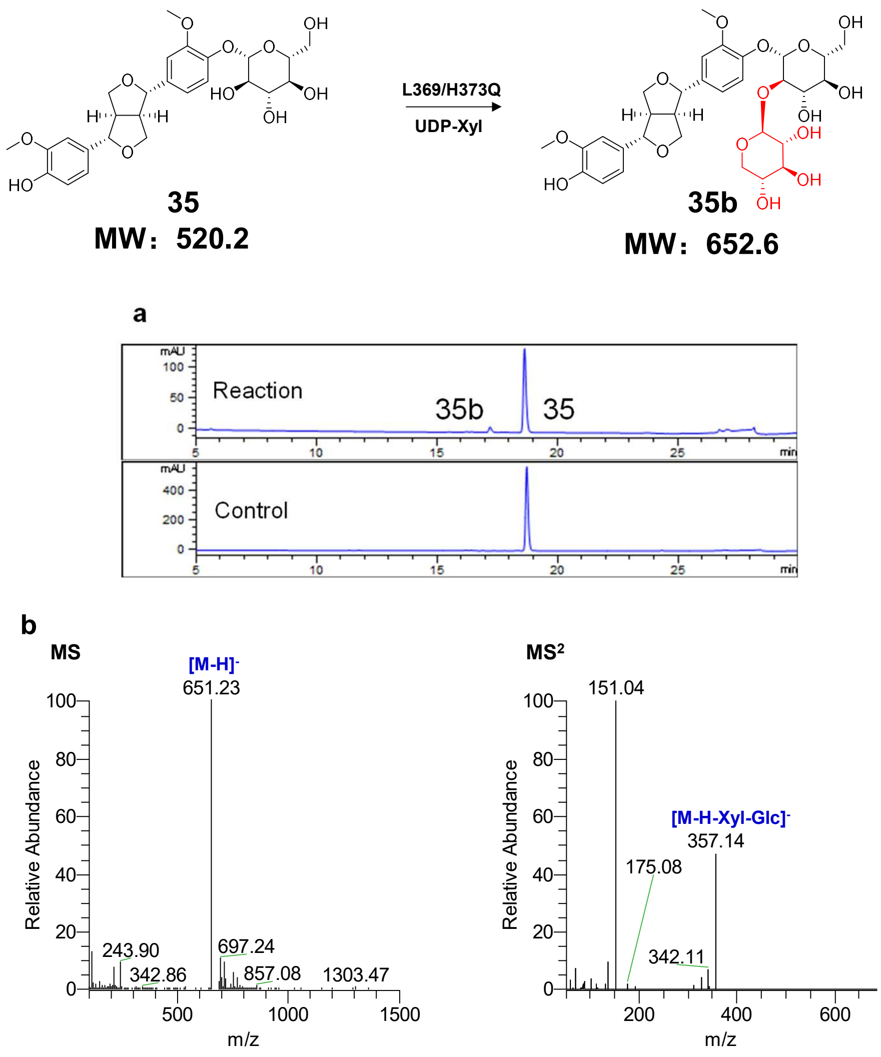

Supplementary Fig. 119 HPLC and LC/MS analyses of L369/H373Q catalytic reaction mixture for substrate **35**. **a**, HPLC analysis of L369/H373Q catalyzed product using **35** as the substrate. **b**, (-)-ESI-MS and MS^2 spectra of product **35b**. The analytical conditions are given in **Supplementary Table 3**.

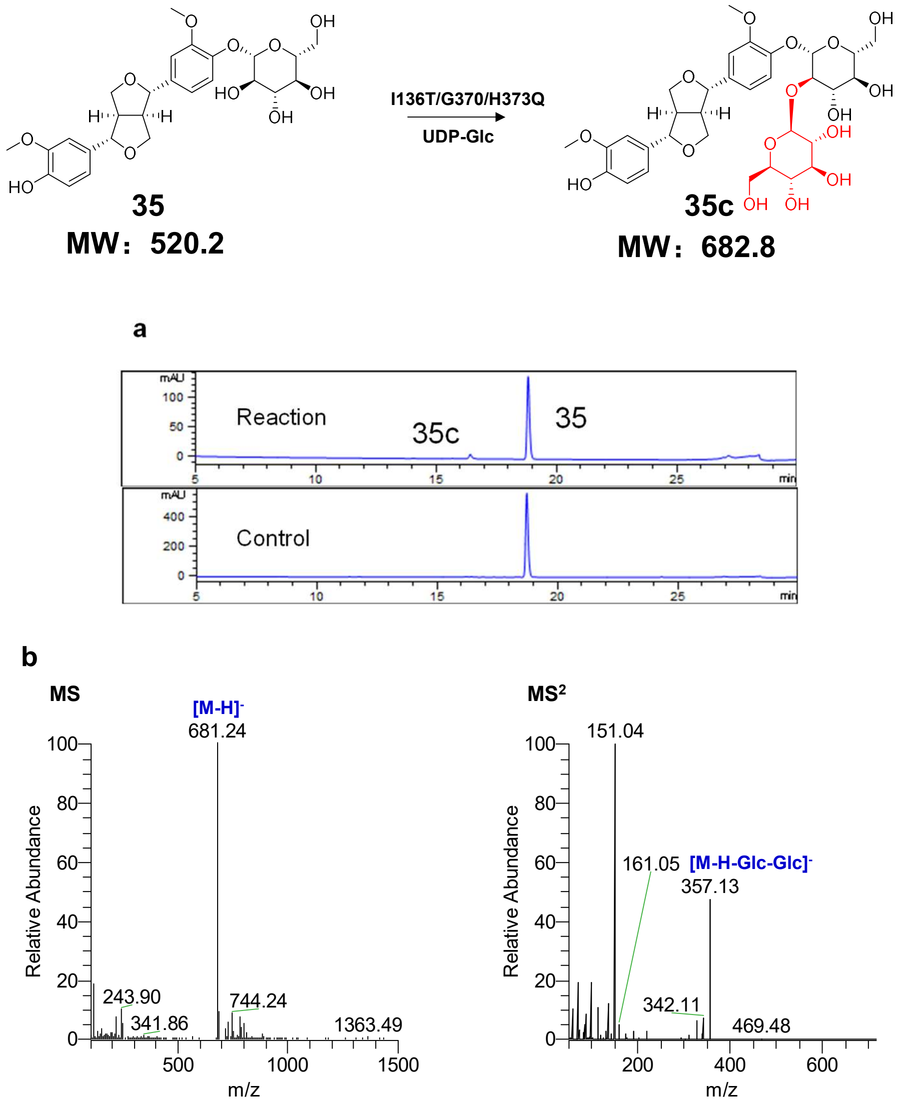

Supplementary Fig. 120 HPLC and LC/MS analyses of I136T/G370/H373Q catalytic reaction mixture for substrate **35**. **a**, HPLC analysis of I136T/G370/H373Q catalyzed product using **35** as the substrate. **b**, (-)-ESI-MS and MS^2 spectra of product **35c**. The analytical conditions are given in **Supplementary Table 3**.

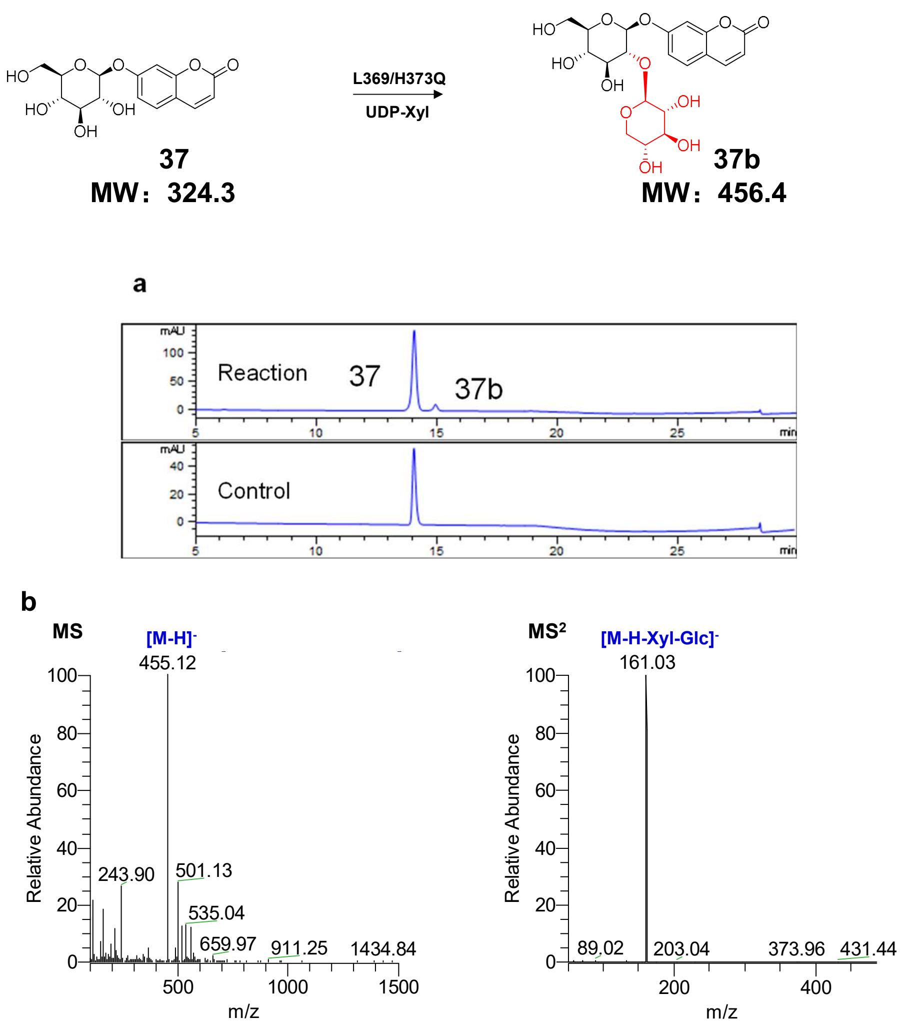

Supplementary Fig. 121 HPLC and LC/MS analyses of L369/H373Q catalytic reaction mixture for substrate **37**. **a**, HPLC analysis of L369/H373Q catalyzed product using **37** as the substrate. **b**, (-)-ESI-MS and MS^2 spectra of product **37b**. The analytical conditions are given in **Supplementary Table 3**.

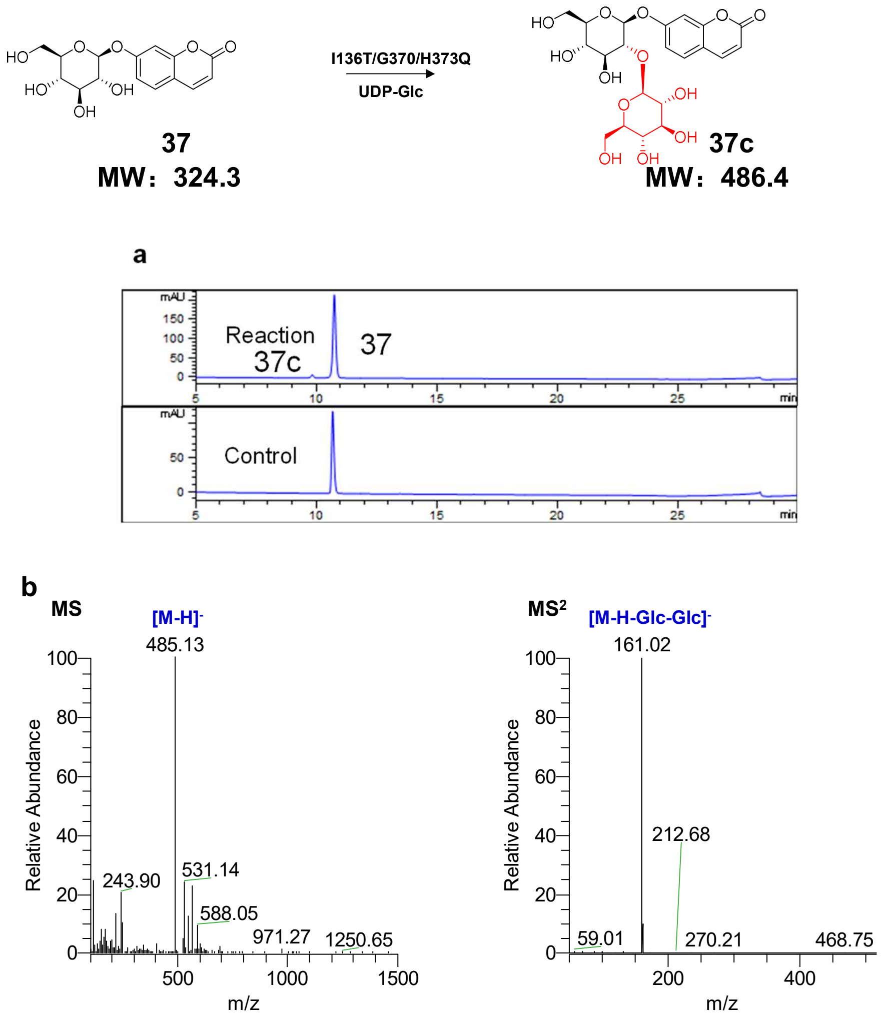

Supplementary Fig. 122 HPLC and LC/MS analyses of I136T/G370/H373Q catalytic reaction mixture for substrate **37**. **a**, HPLC analysis of I136T/G370/H373Q catalyzed product using **37** as the substrate. **b**, (-)-ESI-MS and MS^2 spectra of product **37c**. The analytical conditions are given in **Supplementary Table 3**.

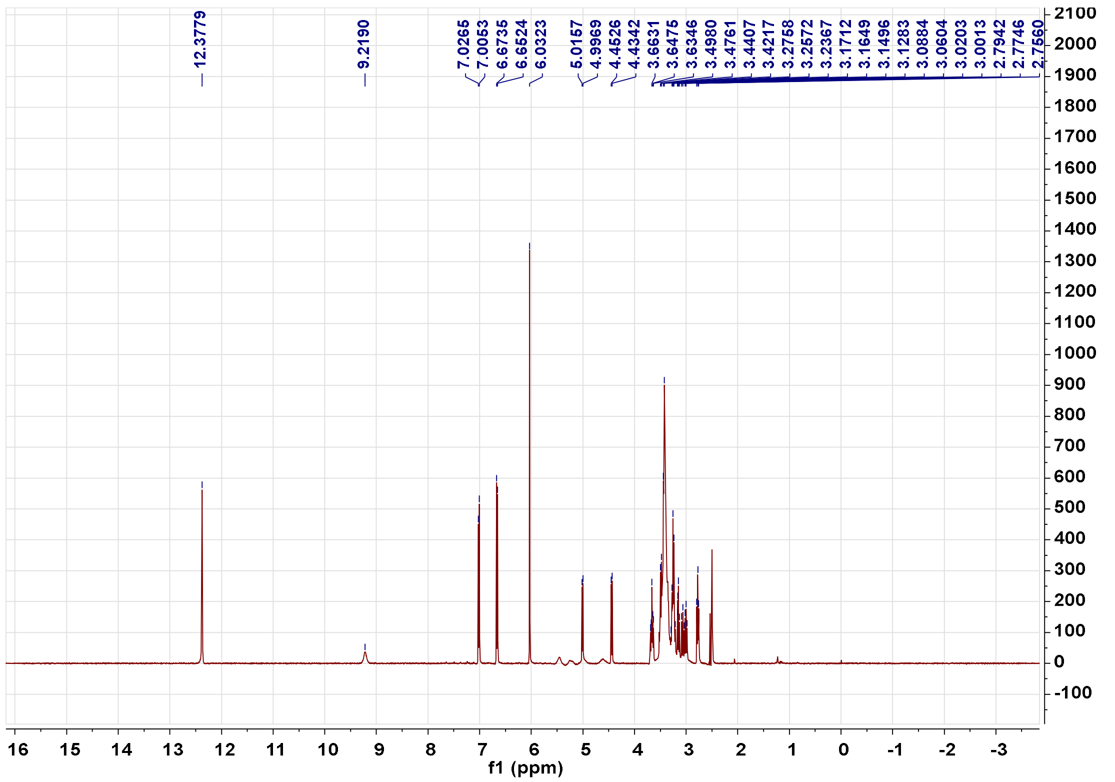

Supplementary Fig. 123 The ^{1}H NMR spectrum of **32b** in DMSO-d_6 (400 MHz).

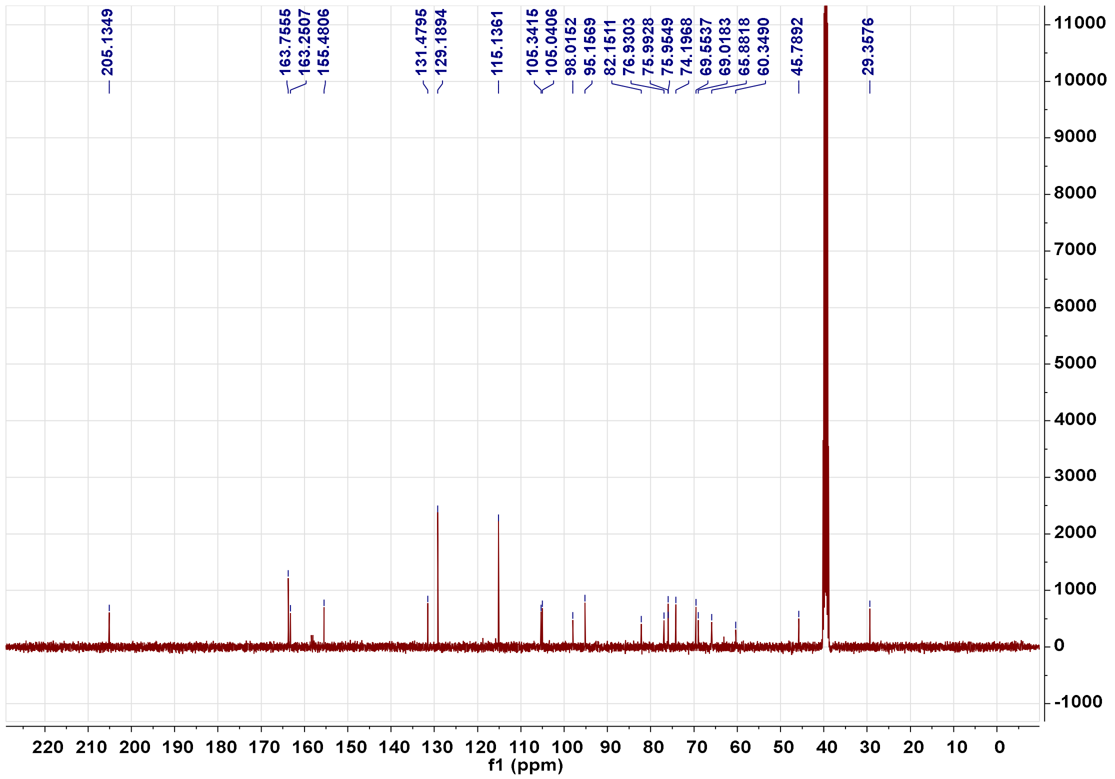

Supplementary Fig. 124 The ^{13}C NMR spectrum of **32b** in DMSO-d_6 (100 MHz).

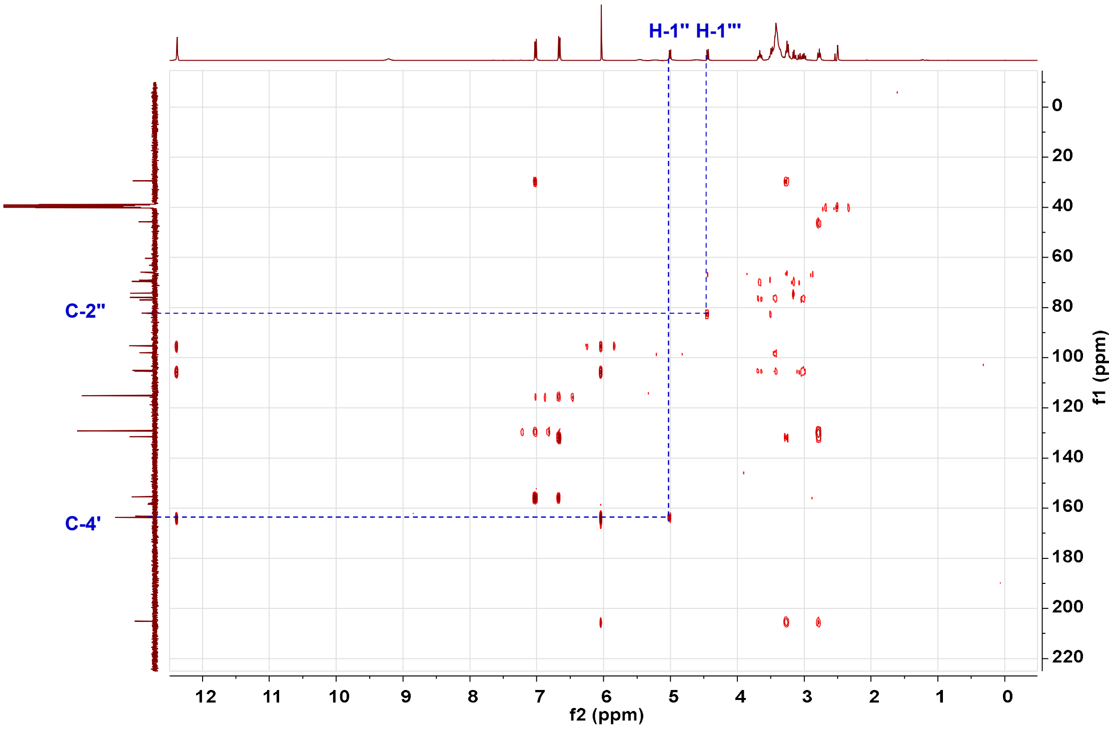

Supplementary Fig. 125 The HMBC spectrum of **32b** in DMSO-d_6 (400 MHz).

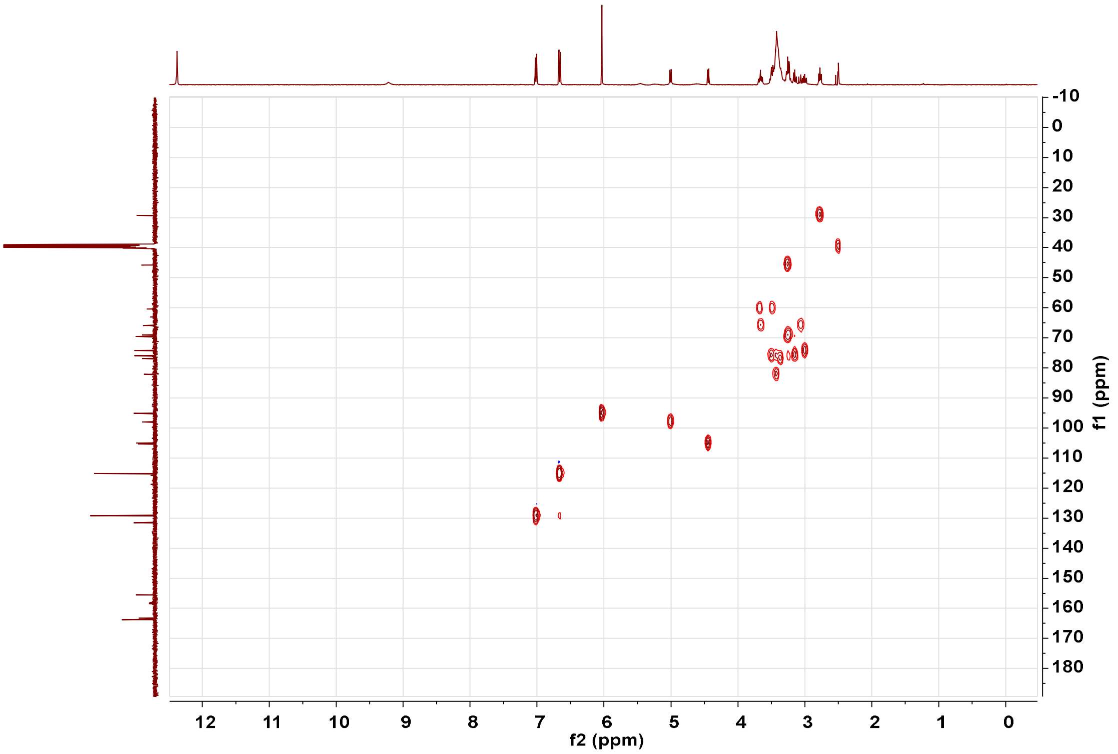

Supplementary Fig. 126 The HSQC spectrum of **32b** in DMSO-d_6 (400 MHz).

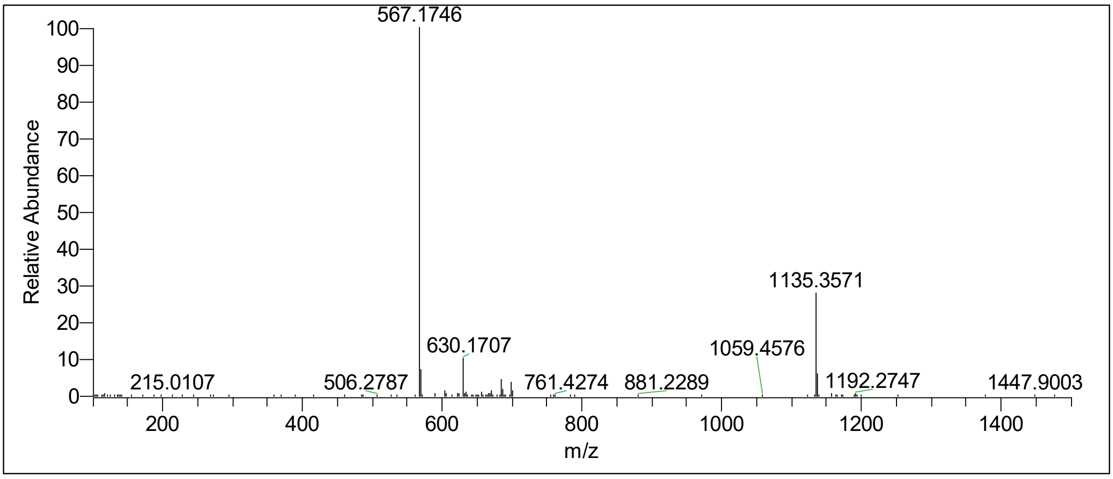

Supplementary Fig. 127 (-)-ESI-HRMS spectrum of **32b**.

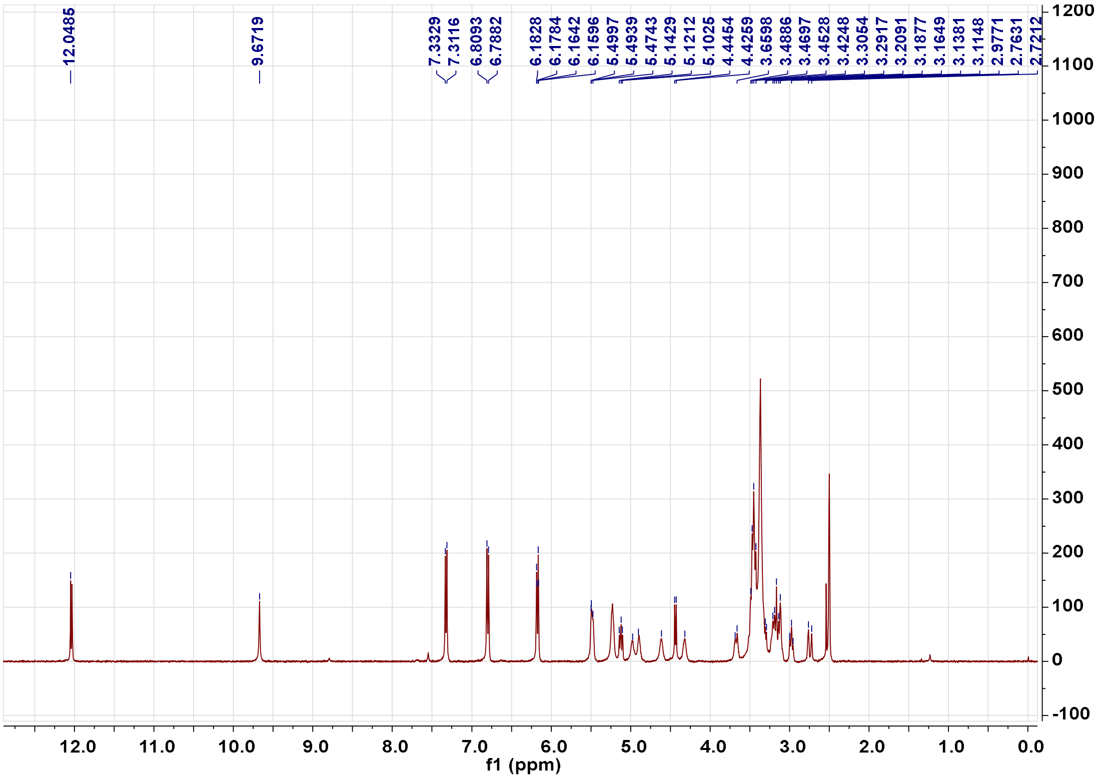

Supplementary Fig. 128 The ^{1}H NMR spectrum of **3c** in DMSO-d_6 (400 MHz).

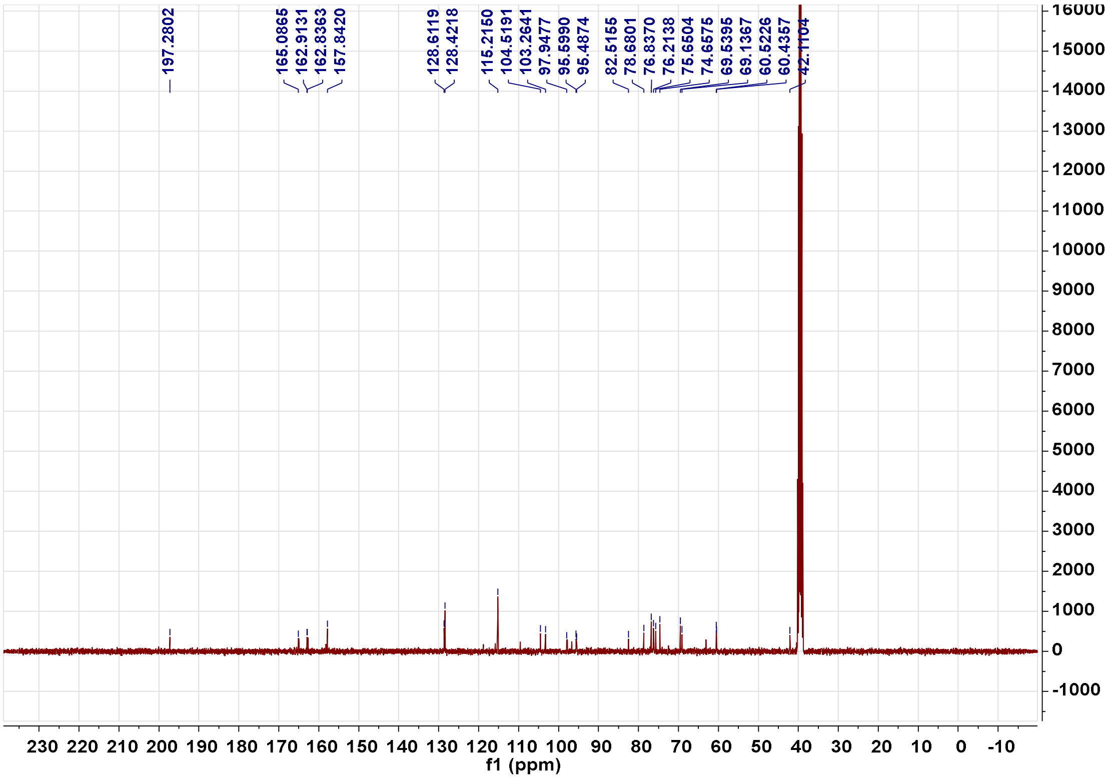

Supplementary Fig. 129 The ^{13}C NMR spectrum of **3c** in DMSO-d_6 (100 MHz).

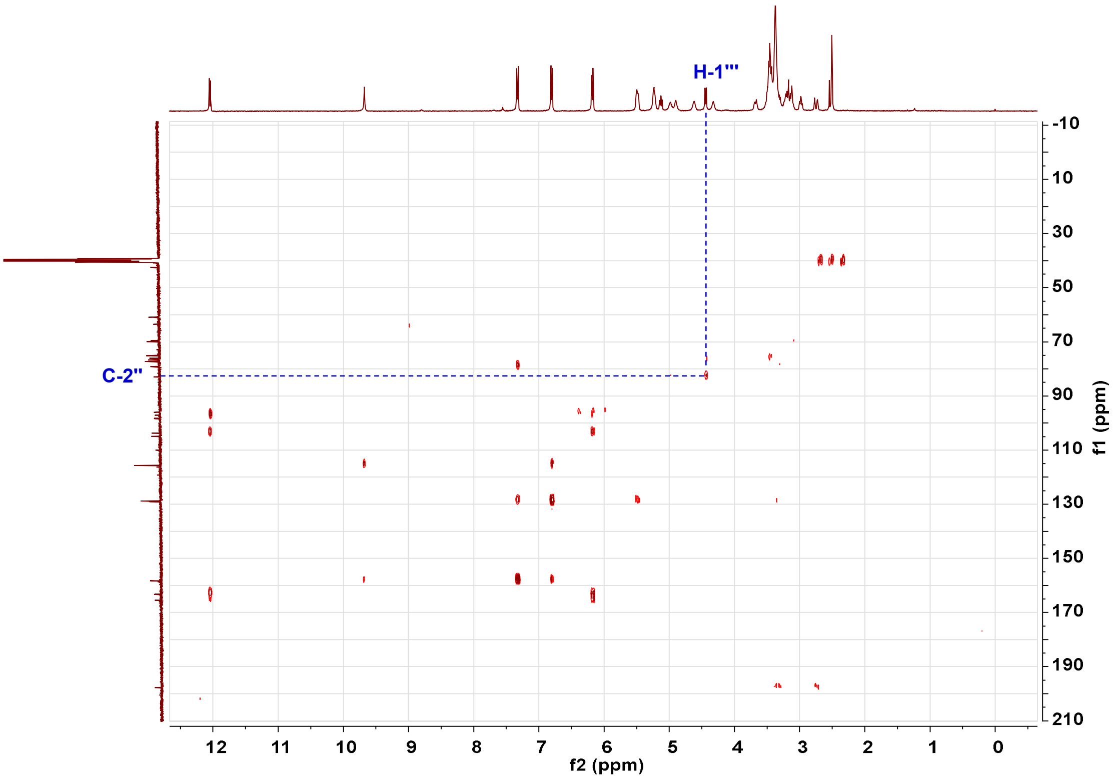

Supplementary Fig. 130 The HMBC spectrum of **3c** in DMSO-d_6 (400 MHz).

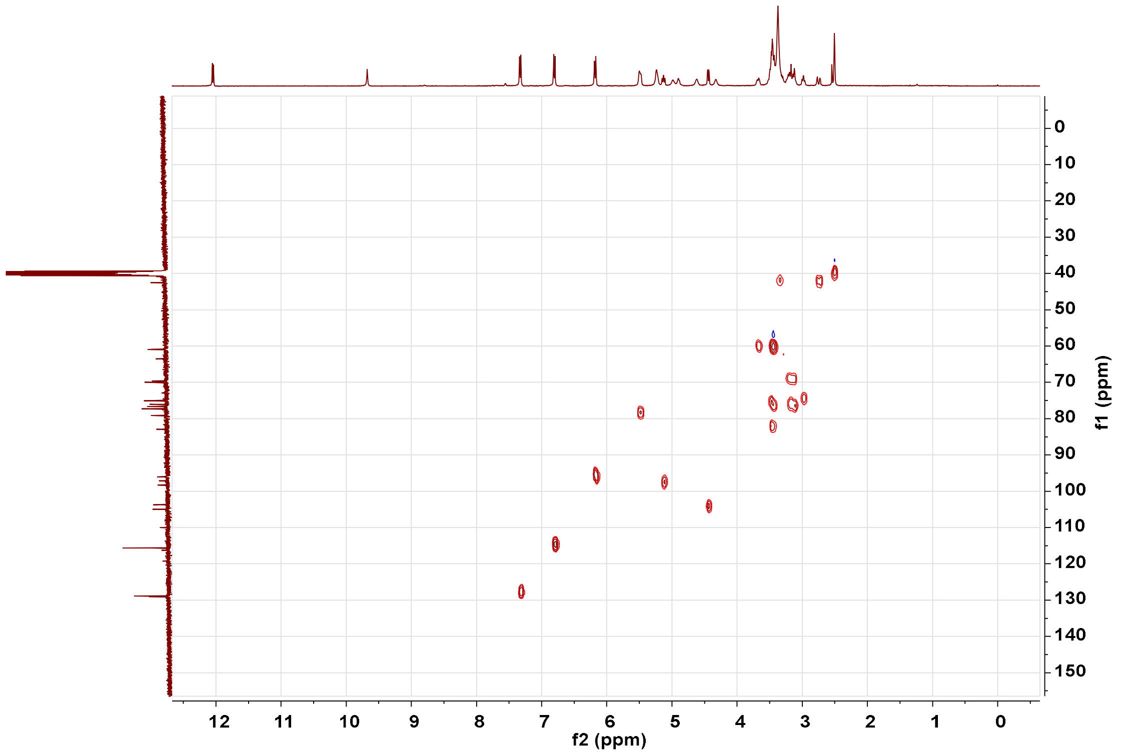

Supplementary Fig. 131 The HSQC spectrum of **3c** in DMSO-d_6 (400 MHz).

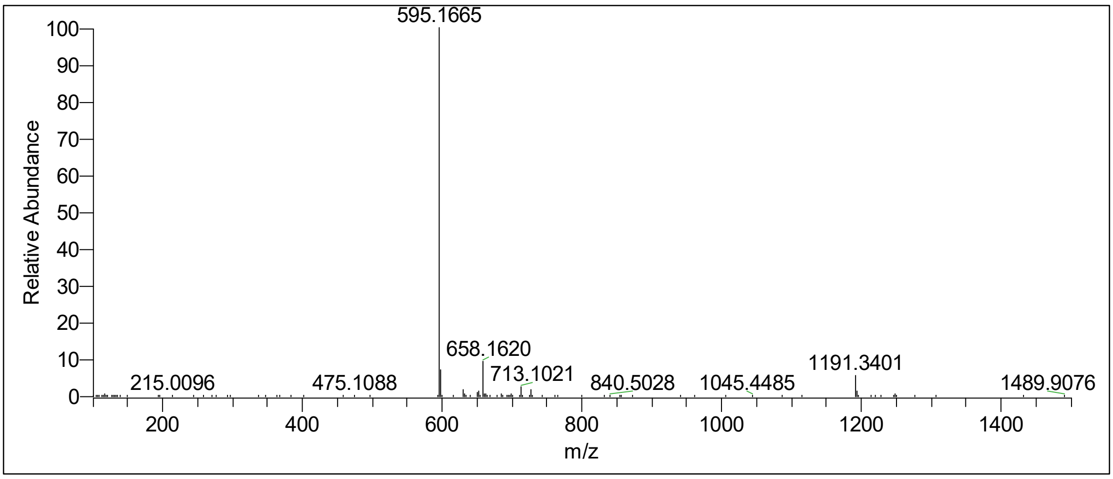

Supplementary Fig. 132 (-)-ESI-HRMS spectrum of **3c**.

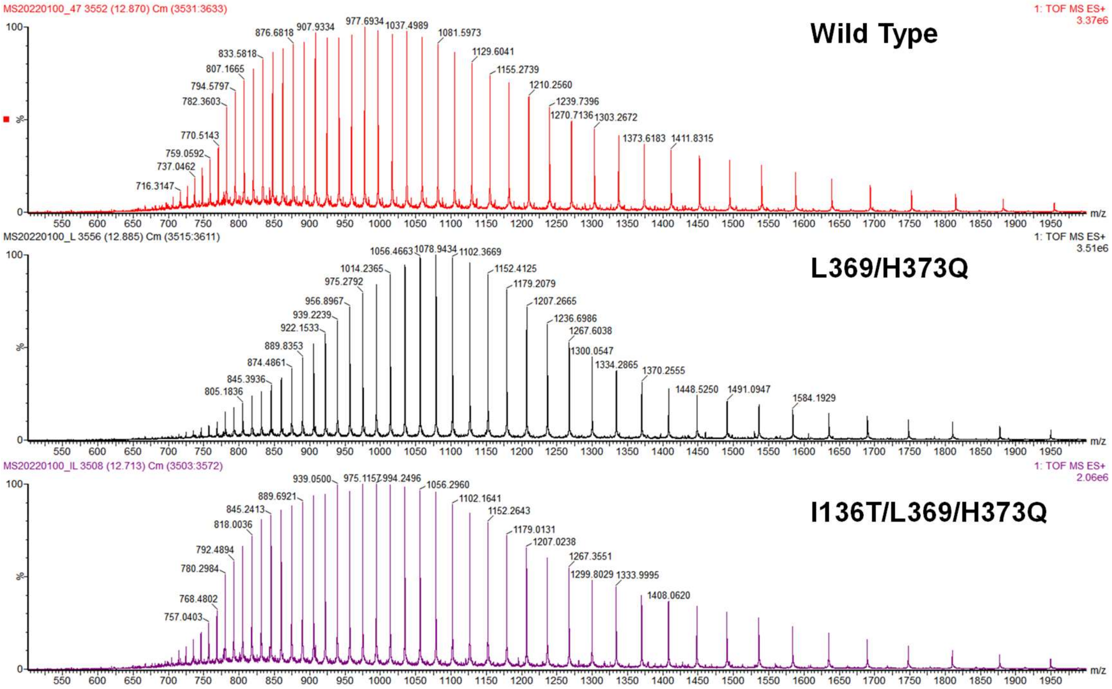

Supplementary Fig. 133 The HDX-MS spectra of GuApiGT (wild type), and L369/H373Q and I136T/L369/H373Q mutants.

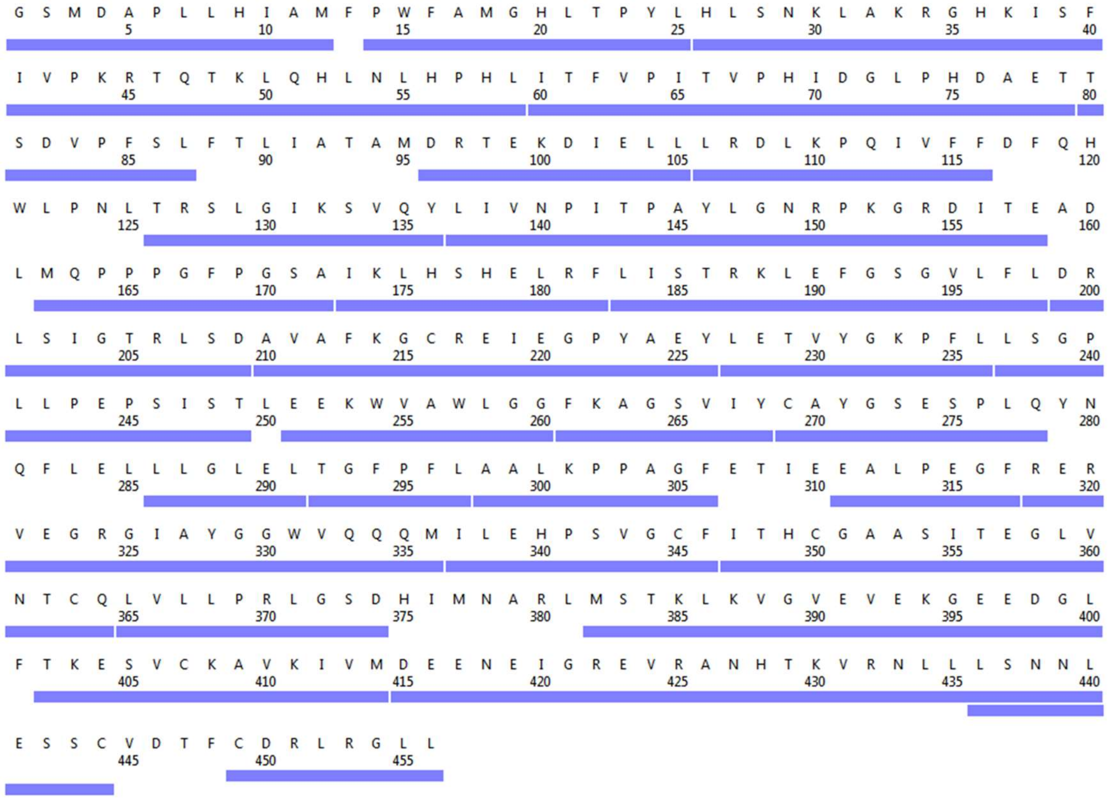

Total: 33 Peptides, 90.1% Coverage, 1.01 Redundancy

Supplementary Fig. 134 The peptide segment coverage of GuApiGT in HDX-MS analysis.

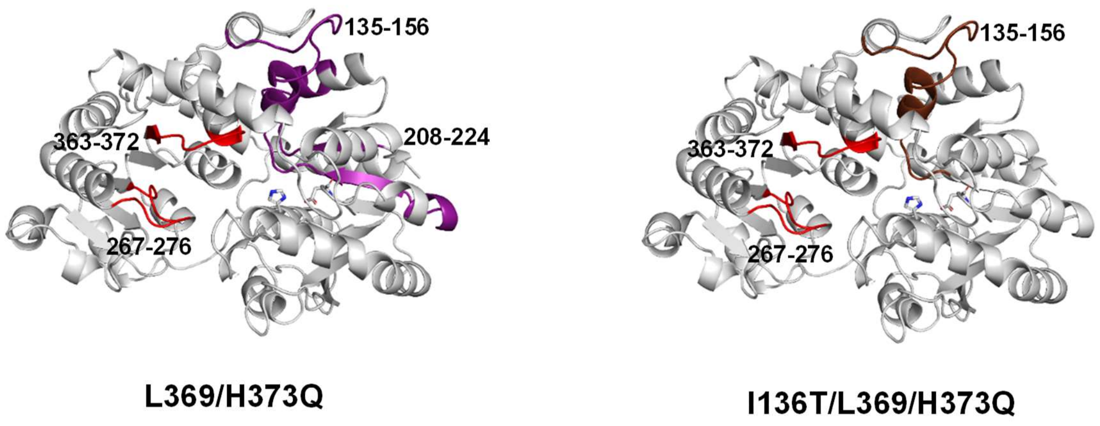

Supplementary Fig. 135 Differential peptides in the model structures of L369/H373Q and I136T/L369/H373Q, by comparing with WT. Peptides with decreased deuterium uptake were labeled in red, and those with increased uptake were labeled in purple and brown, respectively.

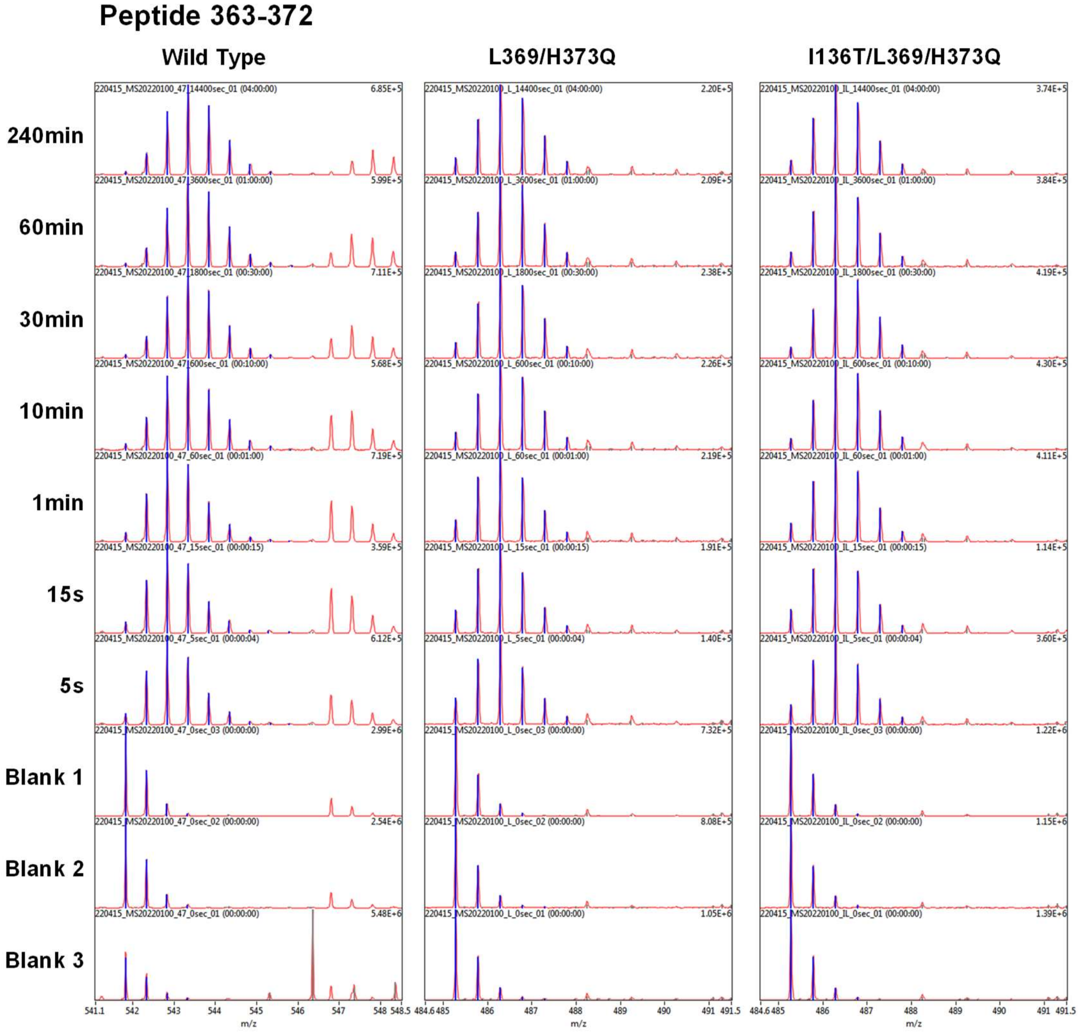

Supplementary Fig. 136 Deuterium uptake plots of peptide 363-372 at different time points.

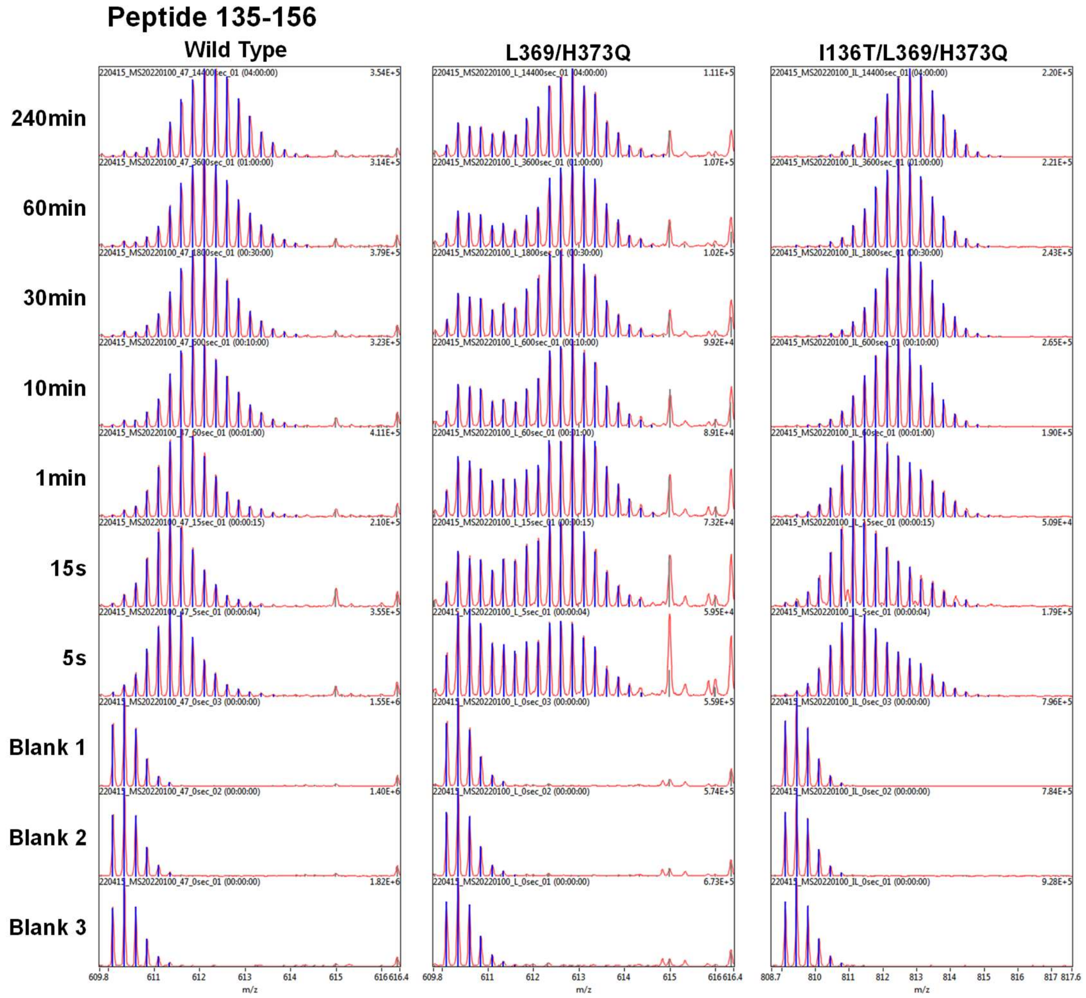

Supplementary Fig. 137. Deuterium uptake plots of peptide 135-156 at different time points.

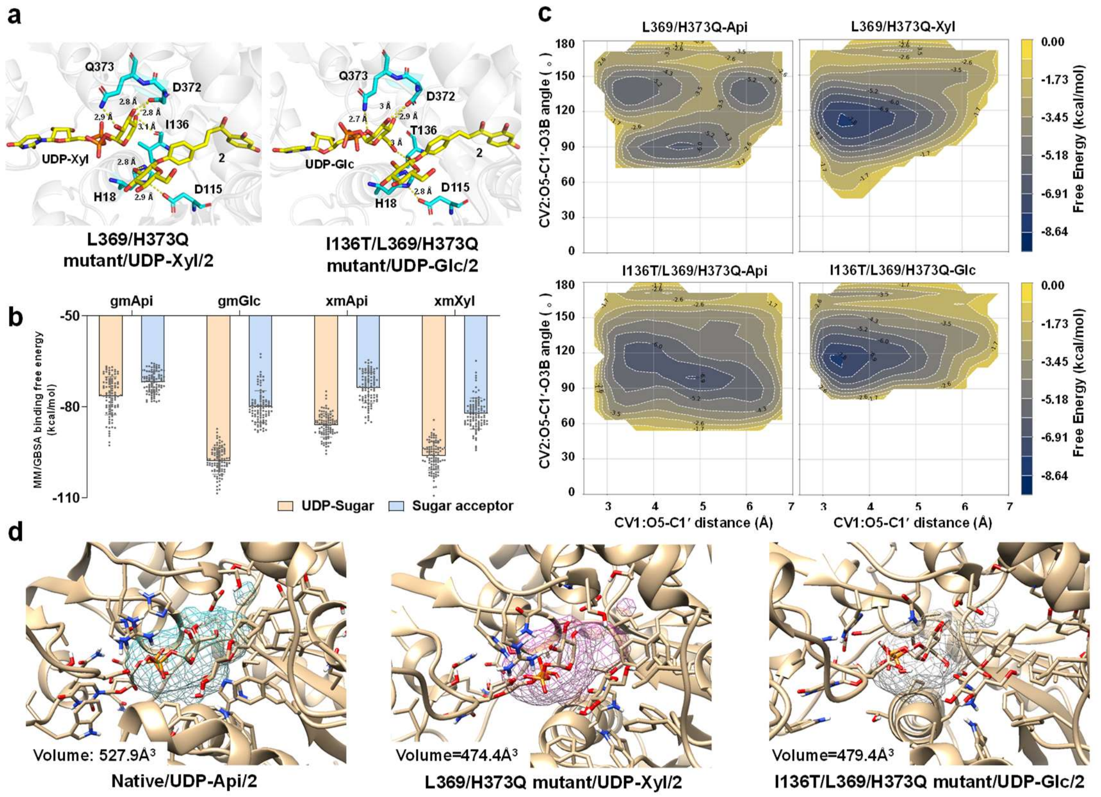

Supplementary Fig. 138 Sugar donor selectivity mechanisms of GuApiGT mutants. **a**, The complex models of L369/H373Q mutant/UDP-Xyl/**2** and I136T/L369/H373Q mutant/UDP-Glc/**2**. The representative configurations were extracted from MD simulations. The hydrogen-bond interactions are shown as yellow dashes. **b**, The relative MM/GBSA binding free energy for different systems. gmApi, I136T/L369/H373Q mutant/UDP-Api/**2**; gmGlc, I136T/L369/H373Q mutant/UDP-Glc/**2**; xmApi, L369/H373Q mutant/UDP-Api/**2**; xmXyl, L369/H373Q mutant/UDP-Xyl/**2**. **c**, Metadynamic simulations of L369/H373Q and I136T/L369/H373Q mutants with different sugar donors. **d**, Spatial blobs (mesh maps) demonstrating the sugar binding part of the active pocket for GuApiGT and its mutants. Grid interval = 0.9, distance cutoff = 10. Data are presented as mean values ± SD (n=100 biologically independent samples) (**b**). The source data underlying figures (**b-c**) are provided in a Source Data file.

Identity=19.83%

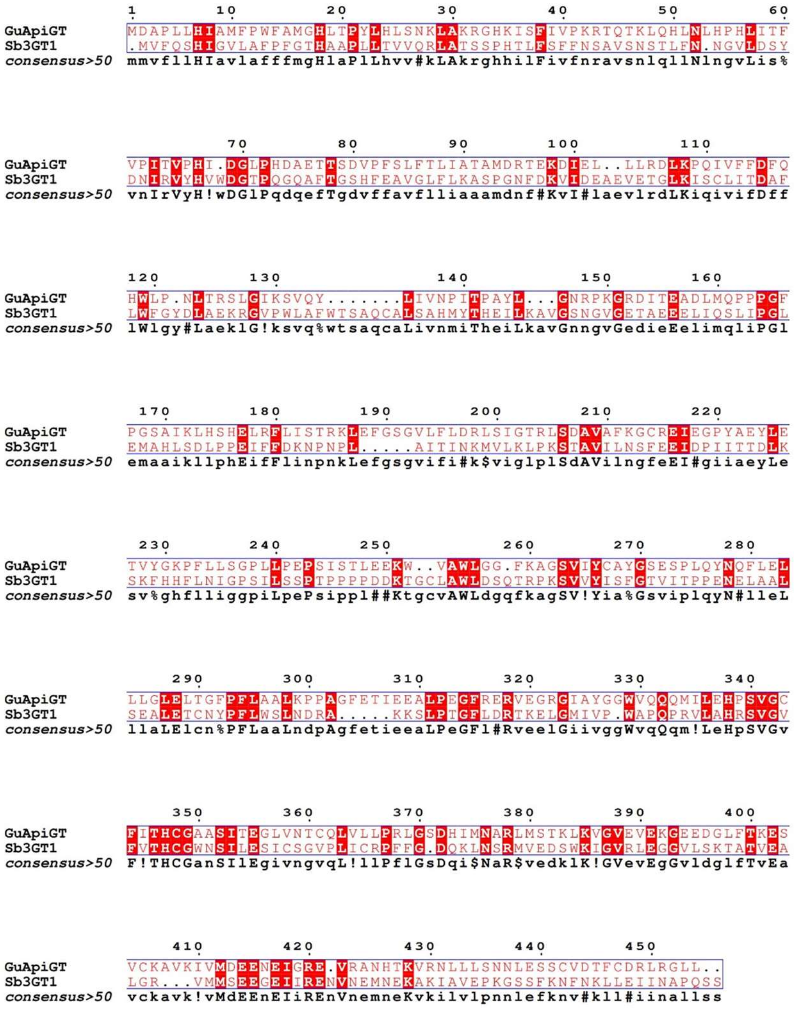

Supplementary Fig. 139 Sequence alignment of Sb3GT1 and GuApiGT. Sb3GT1 shares amino acid sequence identity with GuApiGT of 19.83%. This figure was produced with ENDscript (http://multalin.toulouse.inra.fr/multalin/).

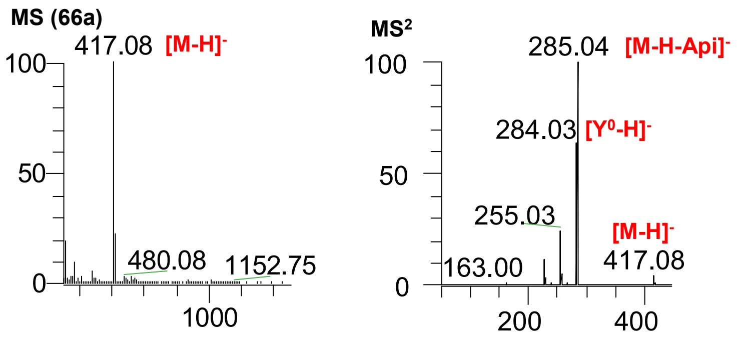

Supplementary Fig. 140 The MS and MS/MS spectra of **66a**. The peak exhibited an $[M-H]^-$ ion at *m*/*z* 417, which is 132 Da greater than that of kaempferol. In MS/MS analysis, the $[M-H]^-$ ion could yield an abundant $[Y^0-H]^-$ product ion at *m*/*z* 284, which was diagnostic for flavonol 3-*O*-glycosides[27]. Thus, product **66a** was tentatively characterized as kaempferol 3-*O*-apioside.

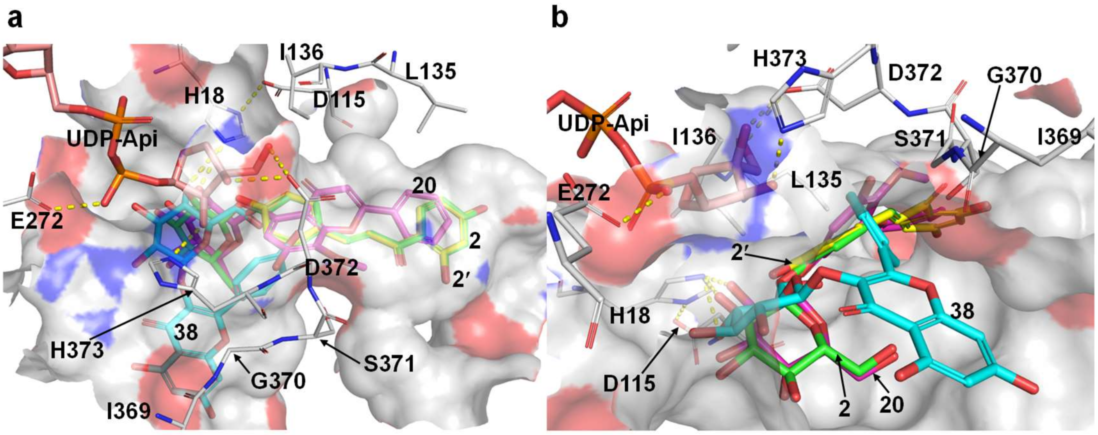

Supplementary Fig. 141 Binding modes of compounds **2** (thick green sticks), **2′** (thick yellow sticks), **20** (thick magenta sticks) and **38** (thick cyan sticks) in GuApiGT. UDP-Api and important residues are depicted as thick pink sticks and thin grey sticks, respectively. The shape of the binding pocket is depicted by surface with the transparency of 0.4. **a**, top view; **b**, front view.

The active site of GuApiGT shapes like a “hammer”. Compound **2** can fit this shape very well, but its aglycone isoliquiritigenin (**2′**) only fits the handle region of this “hammer”. Thus, **2′** is not stable in the active pocket. We further conducted 100-ns MD simulations of GuApiGT/UDP-Api/**2** and GuApiGT/UDP-Api/**2′**. The MM/GBSA binding free energy of **2′** (-58.7 ± 5.3 kcal/mol) in the active pocket is significant higher than **2** (-78.8 ± 4.2 kcal/mol). These data explained why GuApiGT could not accept free aglycones as sugar acceptor. Compound **20** (5-*O*-glycoside) shares a similar binding mode with compound **2**, while compound **38** (3-*O*-glycoside) exhibits great steric hindrance in fitting the handle region.

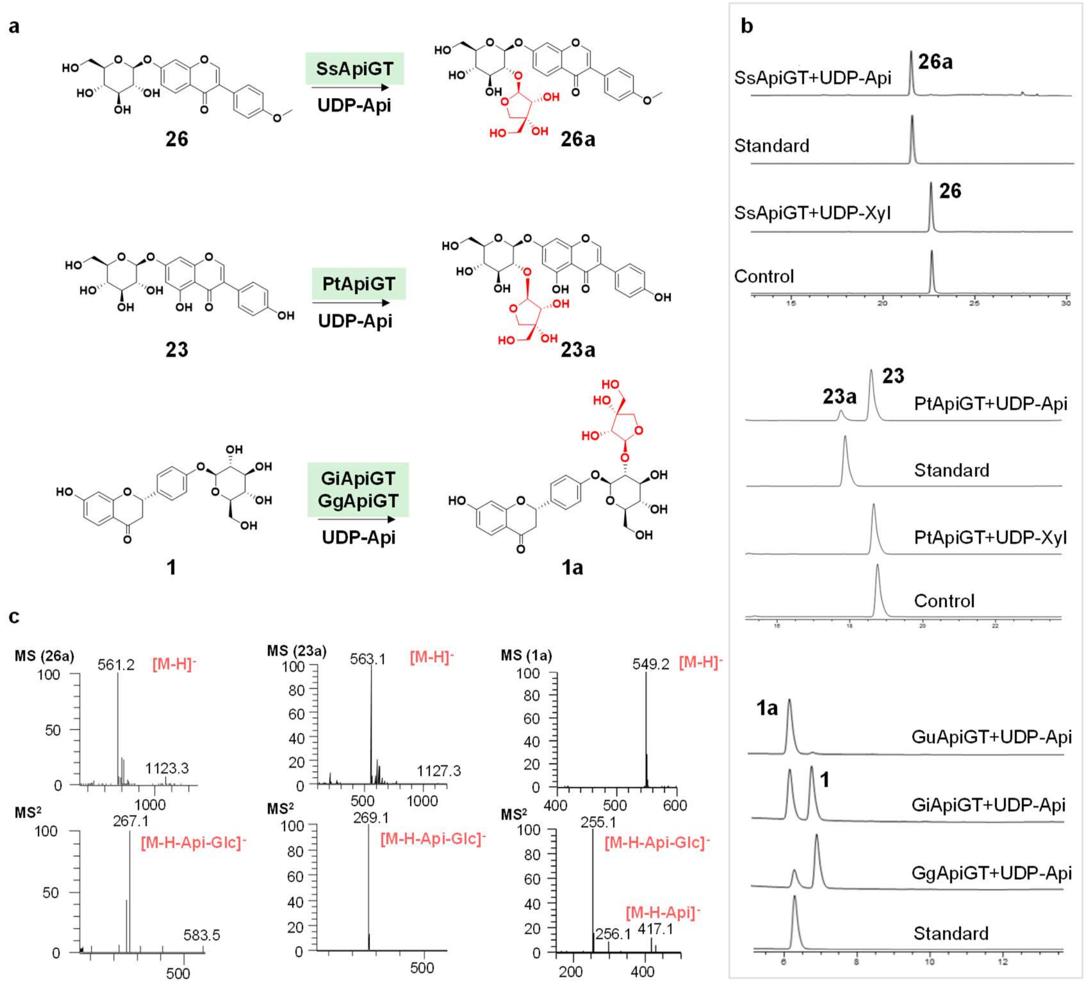

Supplementary Fig. 142 Functional characterization of SsApiGT, PtApiGT, GgApiGT, and GiApiGT. **a**, Catalytic reactions. **26**, **23**, and **1** were used as sugar acceptors, respectively. **b**, HPLC analysis of ApiGT catalyzed products. **c**, (-)-ESI-MS and MS^2 spectra of **26a**, **23a**, and **1a**.

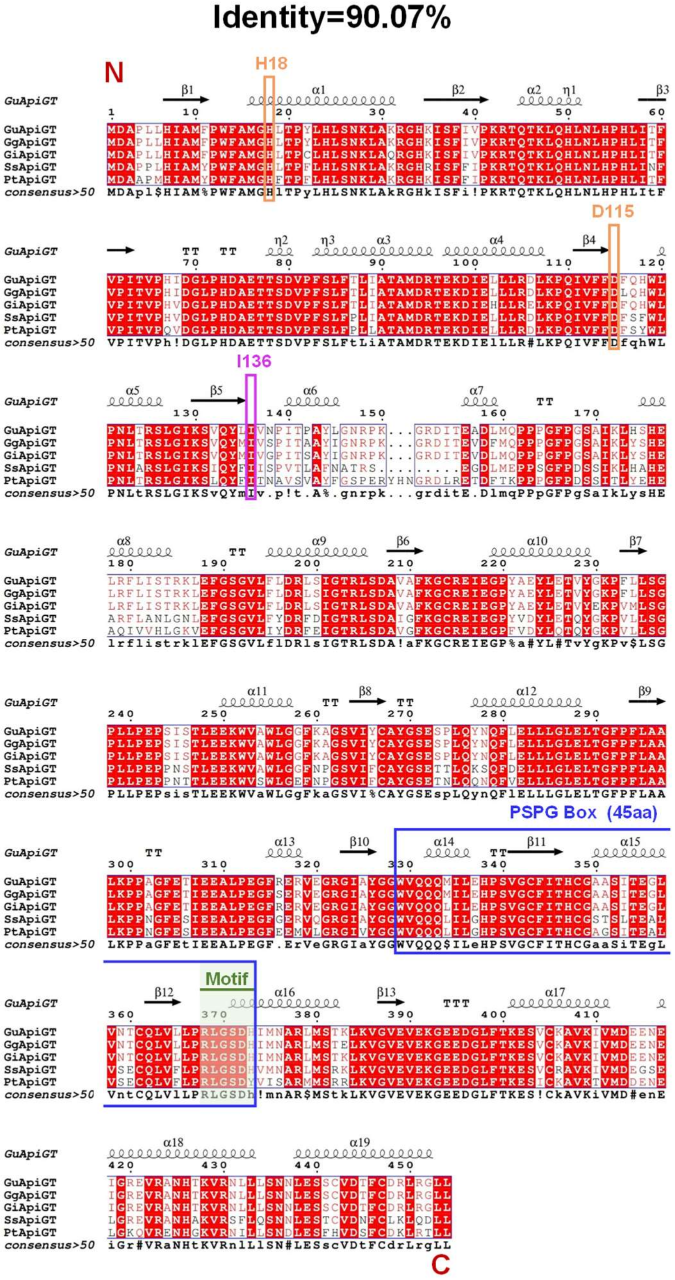

Supplementary Fig. 143 Amino acid sequence alignment of GuApiGT, GgApiGT, GiApiGT, SsApiGT, and PtApiGT. This figure was produced with ENDscript (http://multalin.toulouse.inra.fr/multalin/).

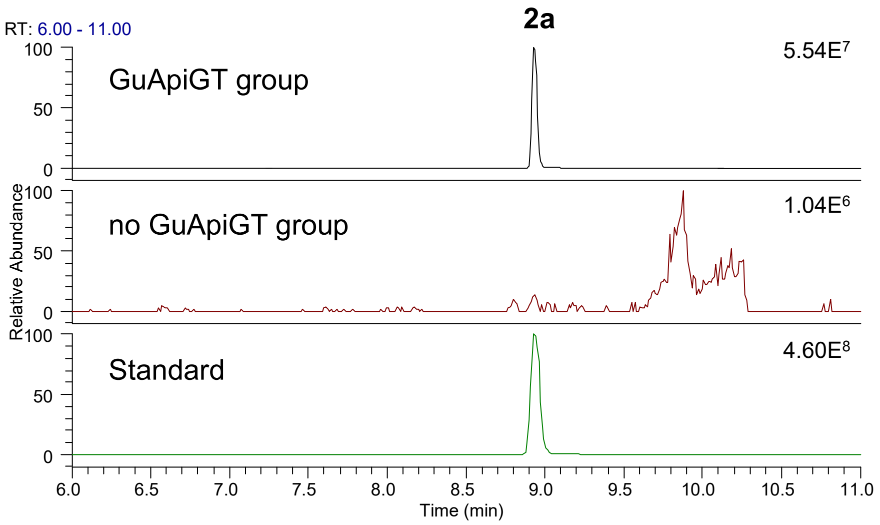

Supplementary Fig. 144 Extracted ion chromatograms (XICs) exhibiting the presence and absence of **2a** in the GuApiGT group and no GuApiGT group.

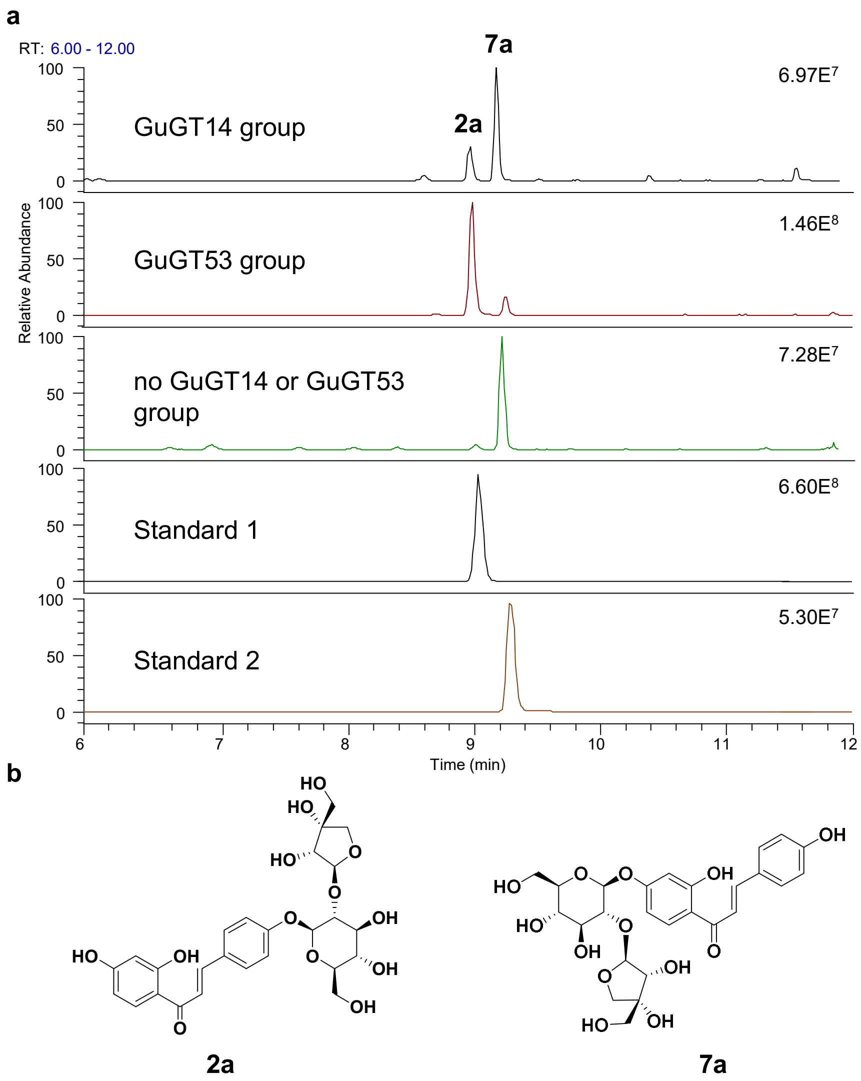

Supplementary Fig. 145 a, Extracted ion chromatograms (XICs) demonstrating the production of **2a** and **7a** in the GuGT14 group, GuGT53 group, and the endogenous GT group. GuGT14 group contained *UAXS*, *GuGT14*, and *GuApiGT* genes. GuGT53 group contained *UAXS*, *GuGT53*, and *GuApiGT* genes. The third group only contained *UAXS* and *GuApiGT* genes. Three days after infiltration, isoliquiritin and UDP-GlcA were supplemented to all the groups. **b**, Structures of **2a** and **7a**.

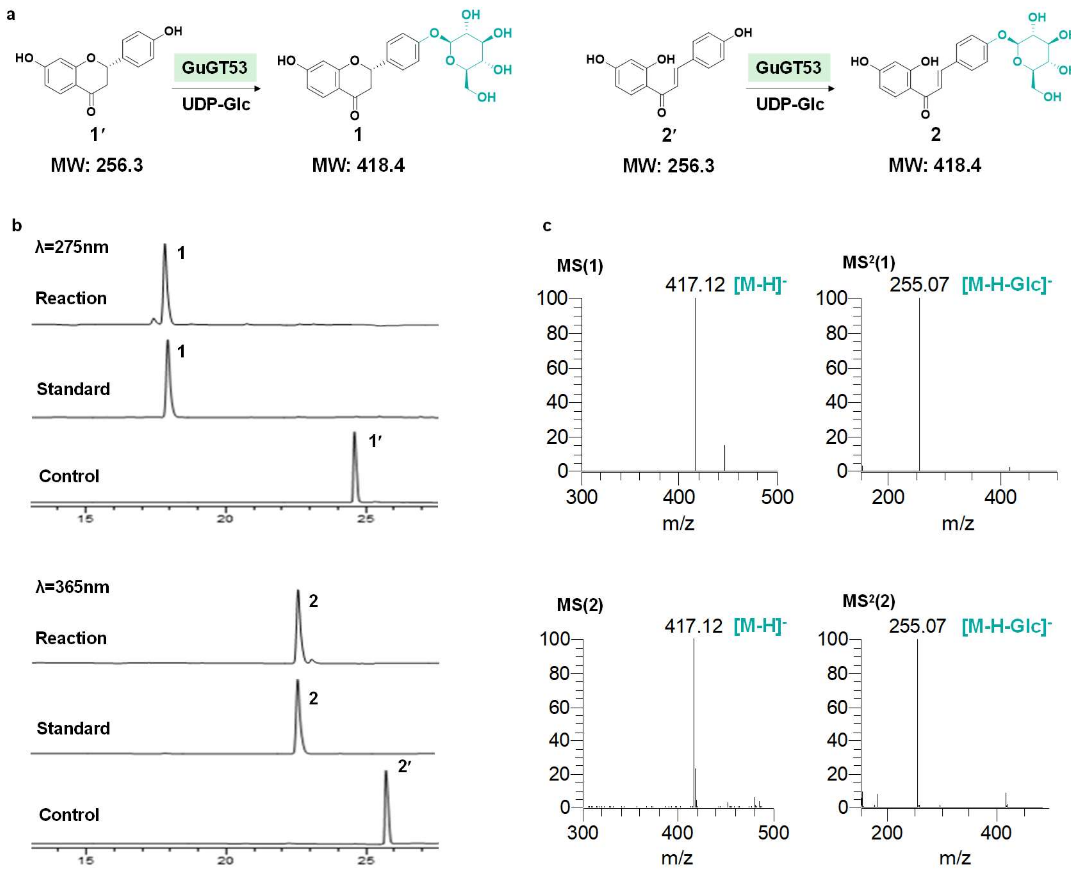

Supplementary Fig. 146 Functional characterization of GuGT53. **a**, Catalytic function of GuGT53 using **1′** and **2′** as sugar acceptors. **b**, HPLC chromatograms of reaction mixtures. **c**, (-)-ESI-MS and MS^2 spectra of **1** and **2**.

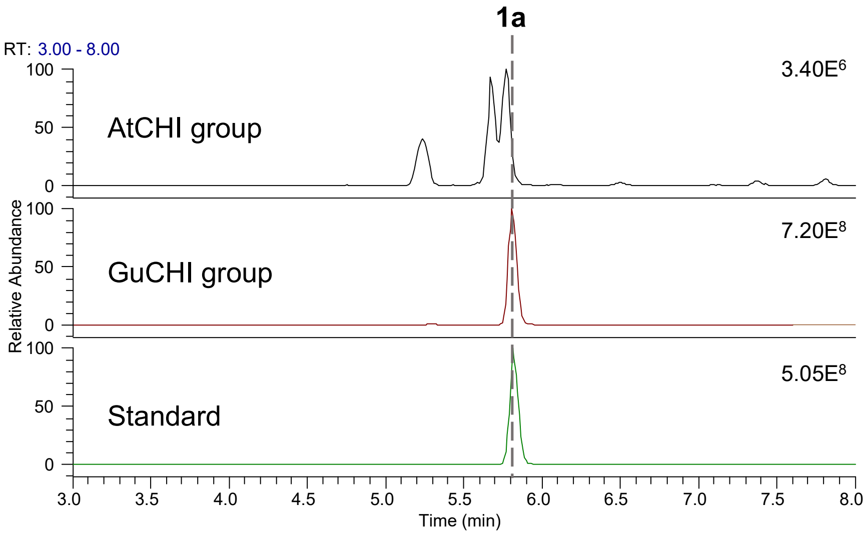

Supplementary Fig. 147 Extracted ion chromatograms (XICs) demonstrating the presence and absence of **1a** in the AtCHI group and GuCHI group.

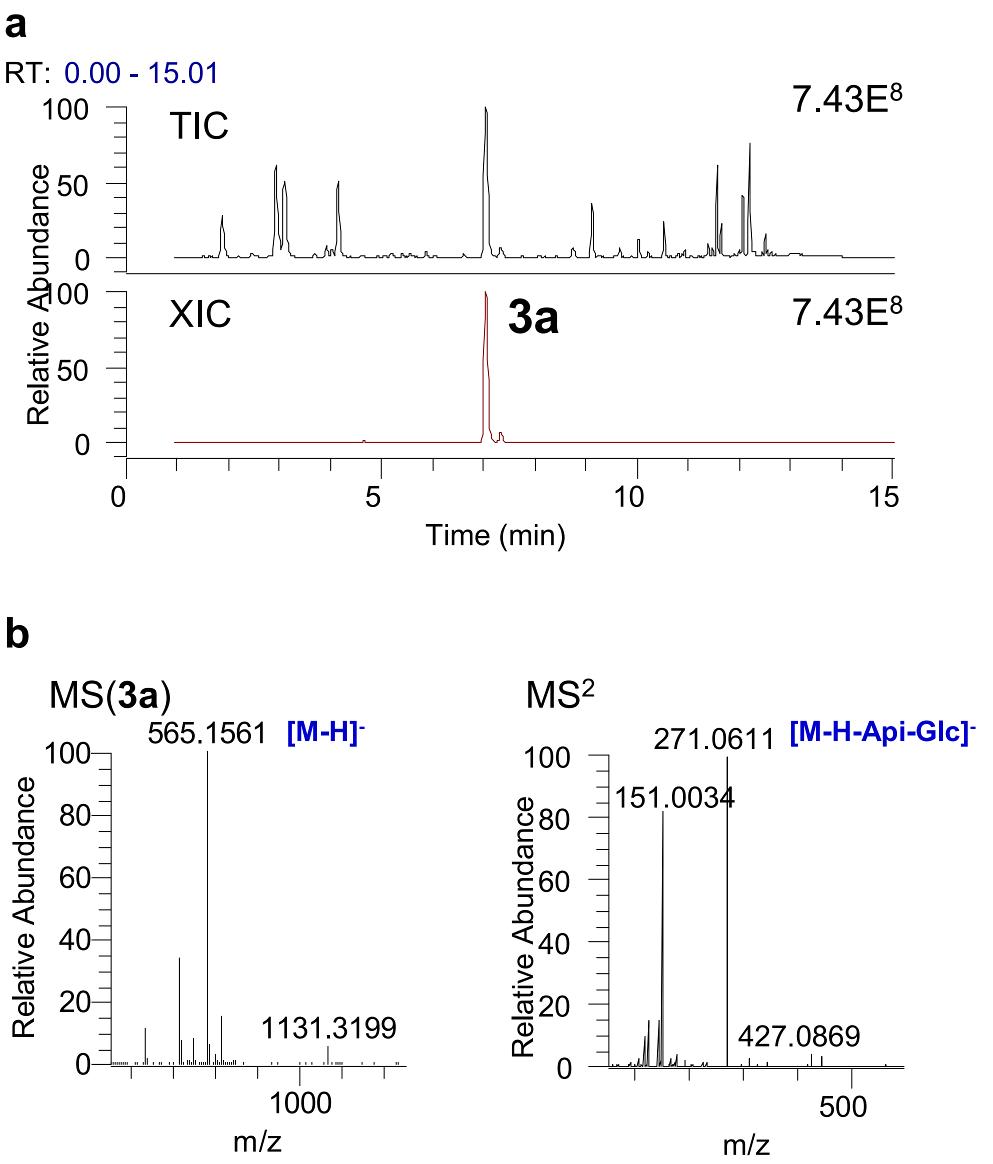

Supplementary Fig. 148 a, LC/MS chromatograms of **3a** in engineered tobacco extracts. **b**, The MS and MS/MS spectra of **3a**. The structure was identified by comparing with the catalytic product of GuApiGT.

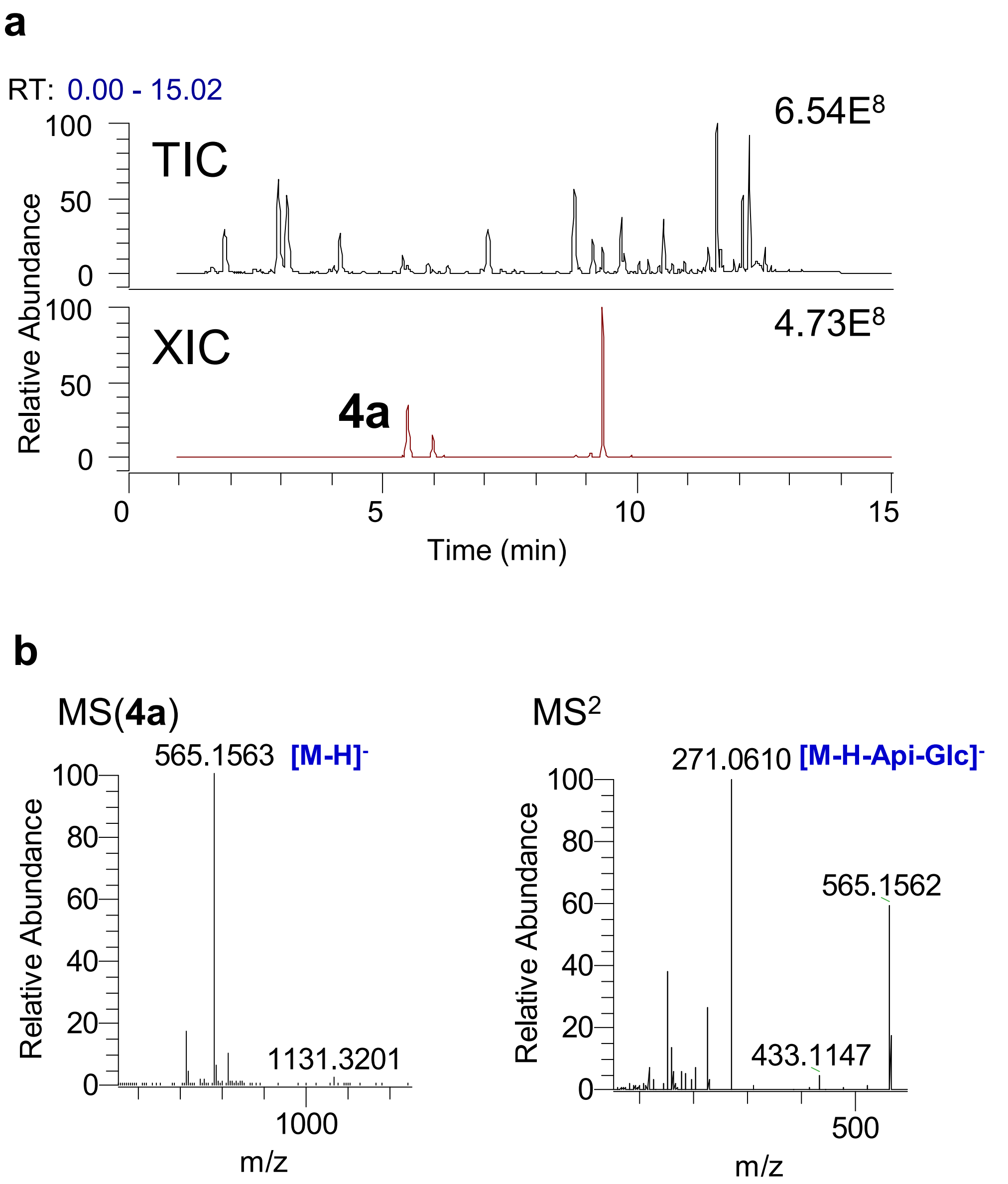

Supplementary Fig. 149 **a**, LC/MS chromatograms of **4a** in engineered tobacco extracts. **b**, The MS and MS/MS spectra of **4a**. The structure was identified by comparing with the catalytic product of GuApiGT.

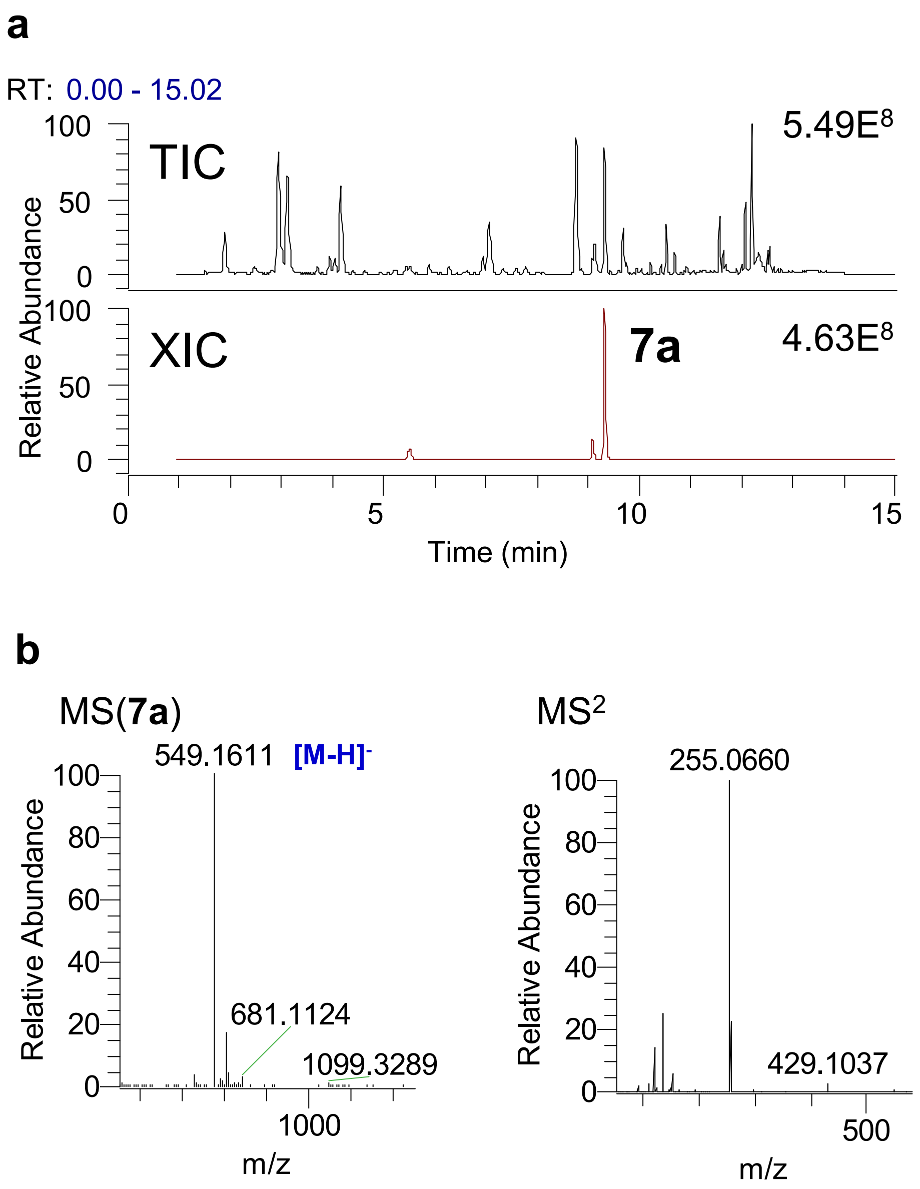

Supplementary Fig. 150 a, LC/MS chromatograms of **7a** in engineered tobacco extracts. **b**, The MS and MS/MS spectra of **7a**. The structure was identified by comparing with the catalytic product of GuApiGT.

a

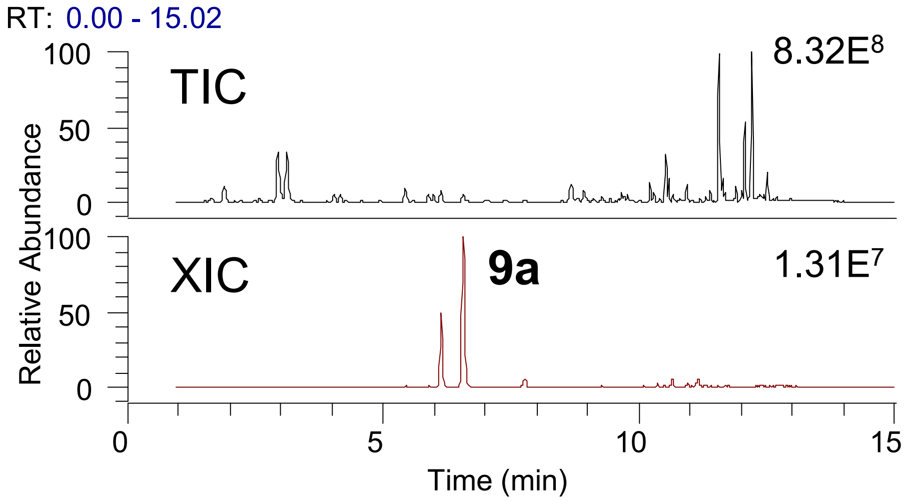

b

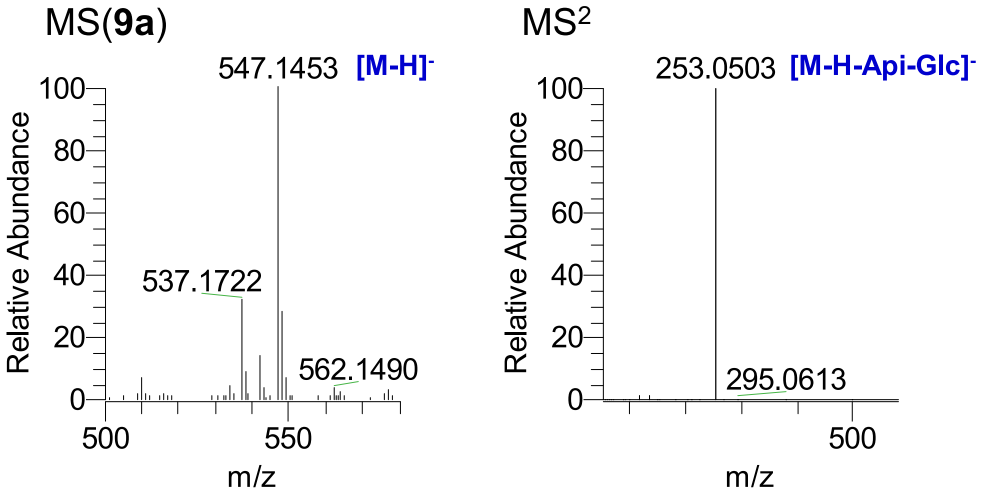

Supplementary Fig. 151 a, LC/MS chromatograms of **9a** in engineered tobacco extracts. **b**, The MS and MS/MS spectra of **9a**. The structure was identified by comparing with the catalytic product of GuApiGT.

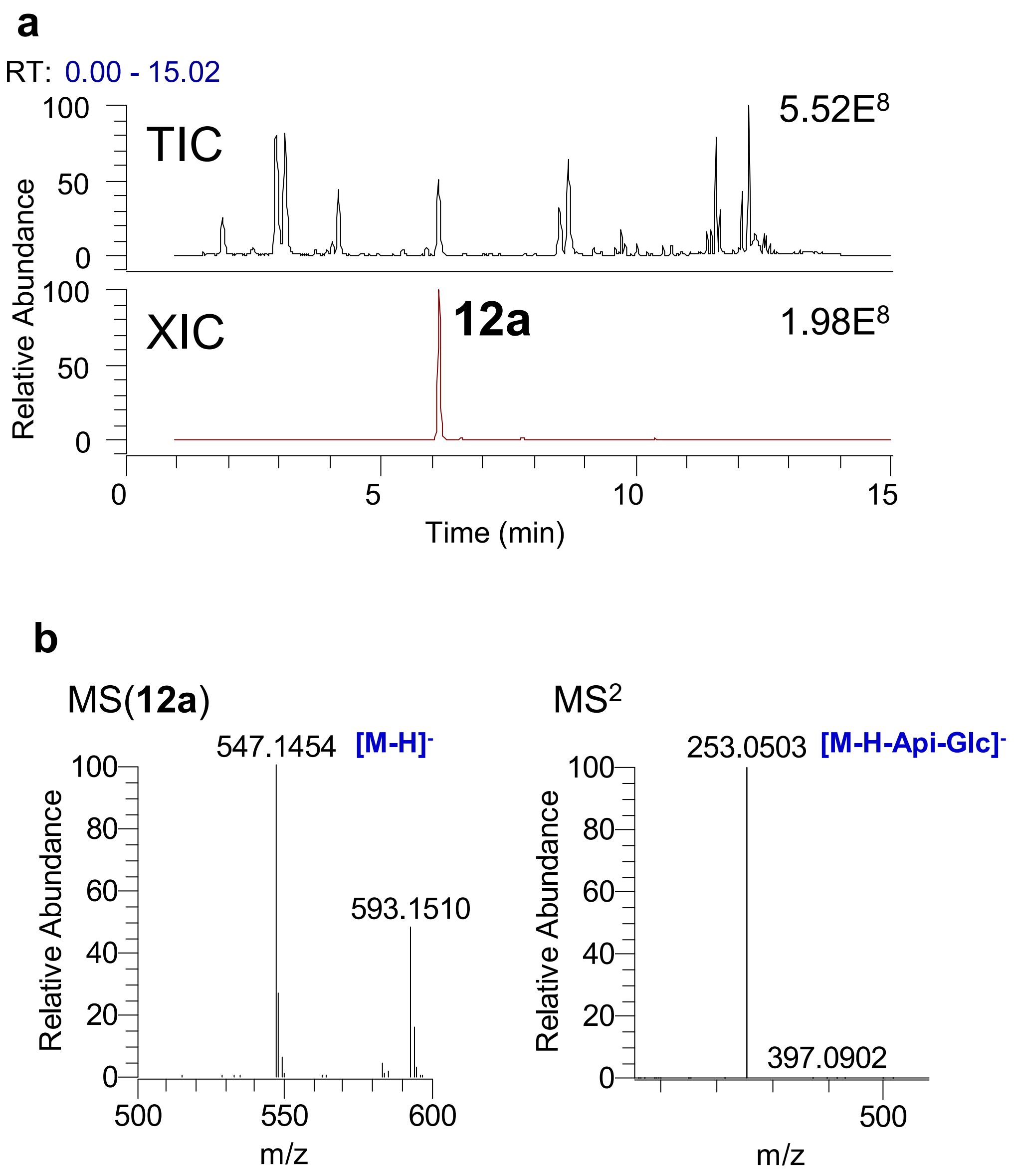

Supplementary Fig. 152 a, LC/MS chromatograms of **12a** in engineered tobacco extracts. **b**, The MS and MS/MS spectra of **12a**. The structure was identified by comparing with a reference standard.

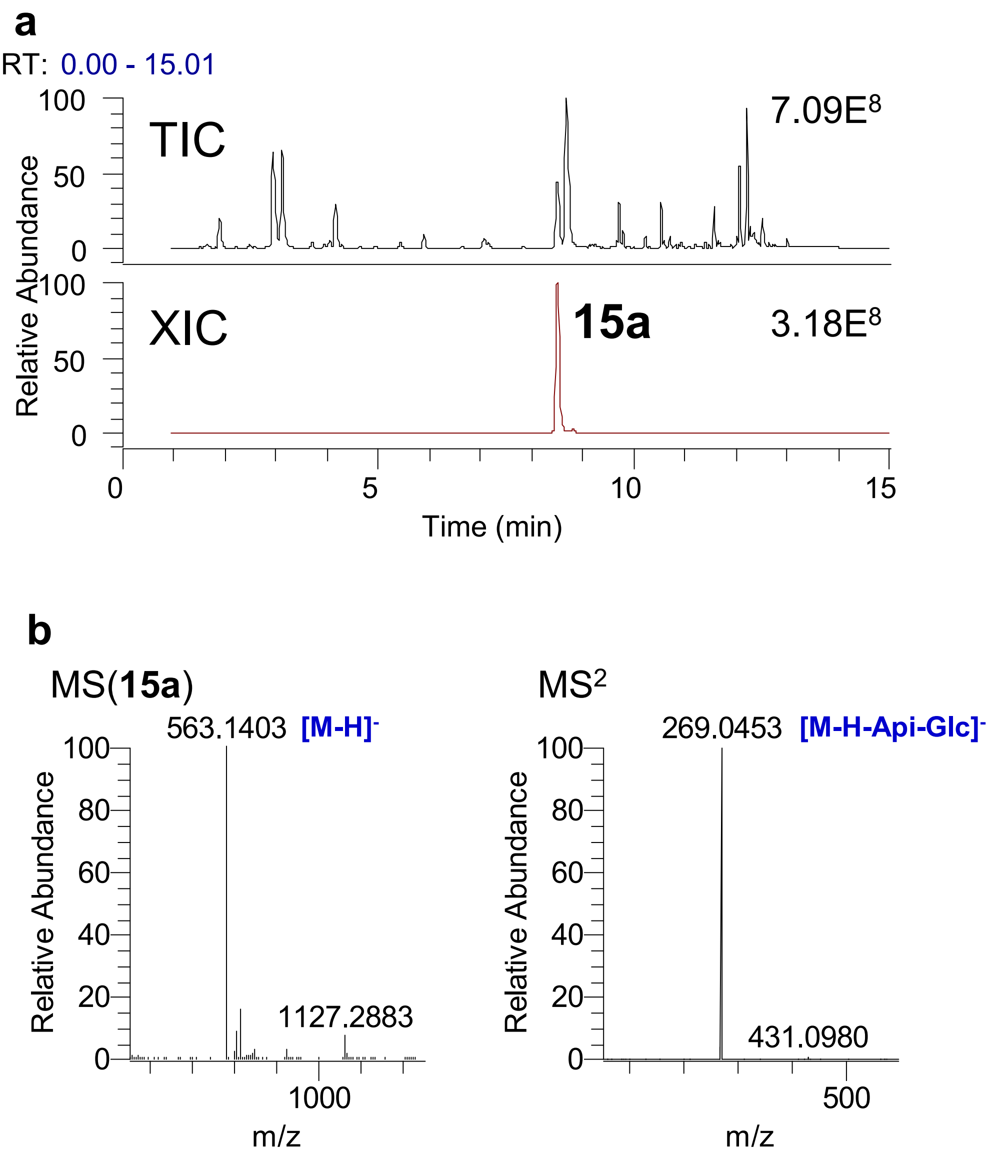

Supplementary Fig. 153 **a**, LC/MS chromatograms of **15a** in engineered tobacco extracts. **b**, The MS and MS/MS spectra of **15a**. The structure was identified by comparing with a reference standard.

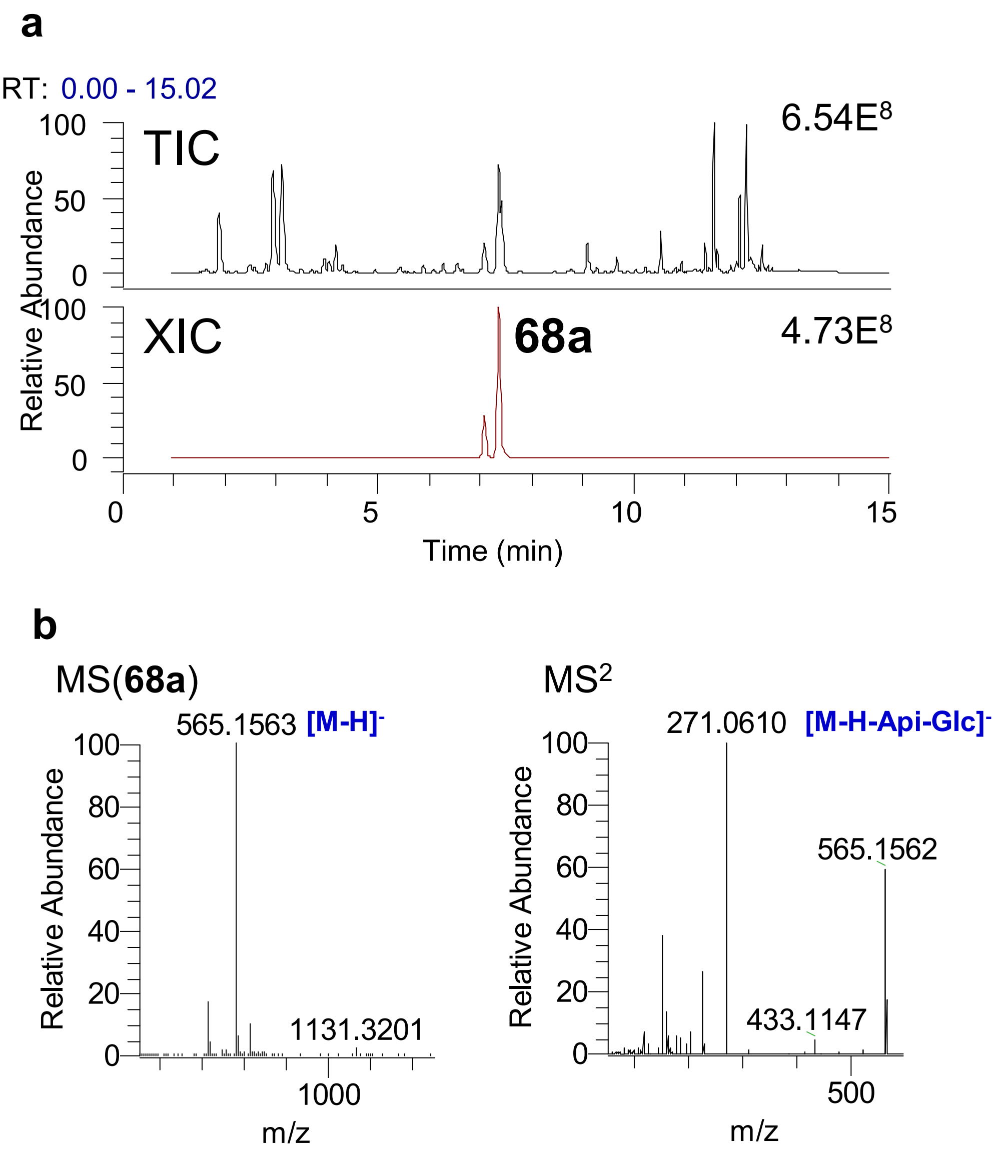

Supplementary Fig. 154 a, LC/MS chromatograms of **68a** in engineered tobacco extracts. **b**, The MS and MS/MS spectra of **68a**. The structure was tentatively characterized by mass spectrometry analysis.

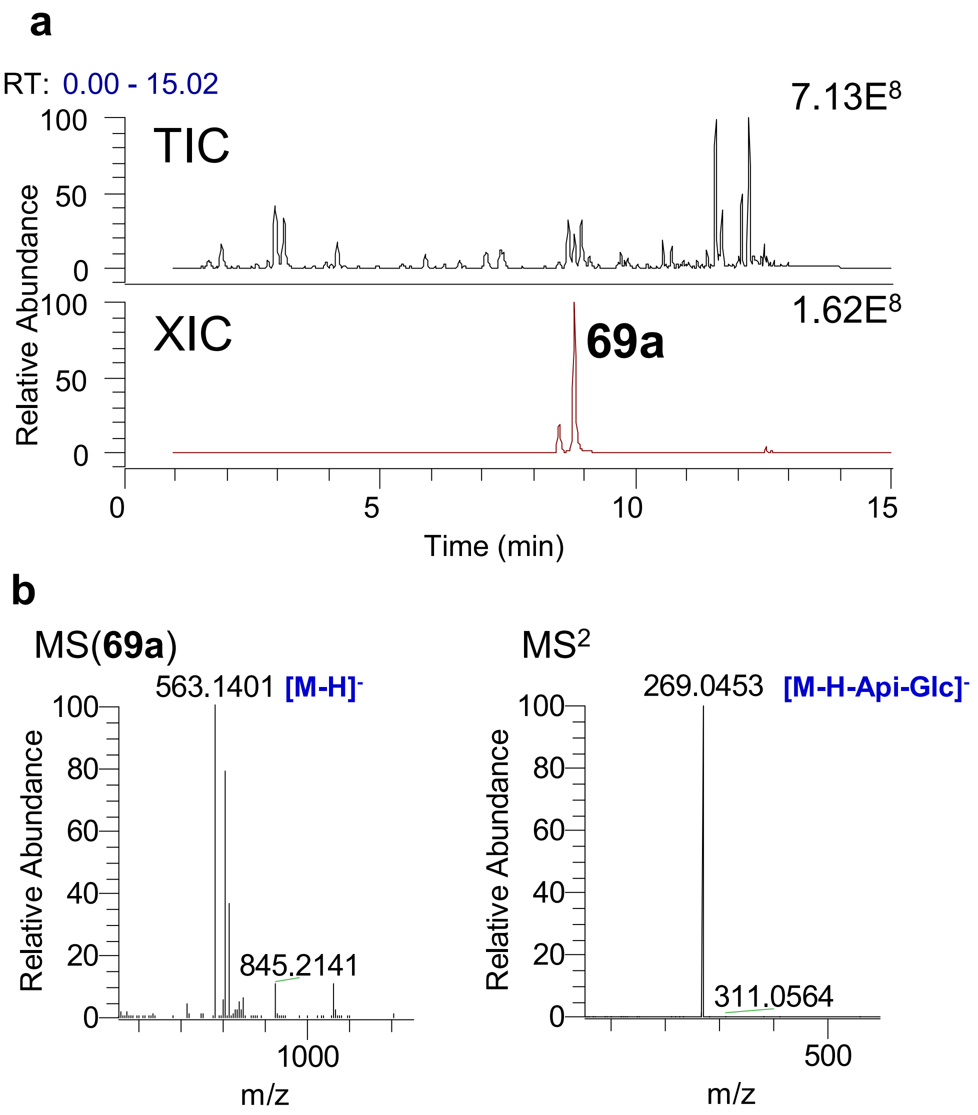

Supplementary Fig. 155 **a**, LC/MS chromatograms of **69a** in engineered tobacco extracts. **b**, The MS and MS/MS spectra of **69a**. The structure was tentatively characterized by mass spectrometry analysis.

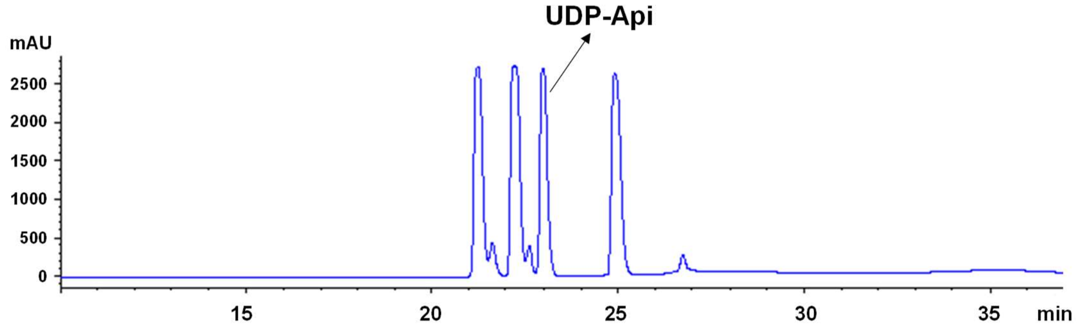

Supplementary Fig. 156 Purification of UDP-Api by HPLC. UV detection wavelength, 262 nm.

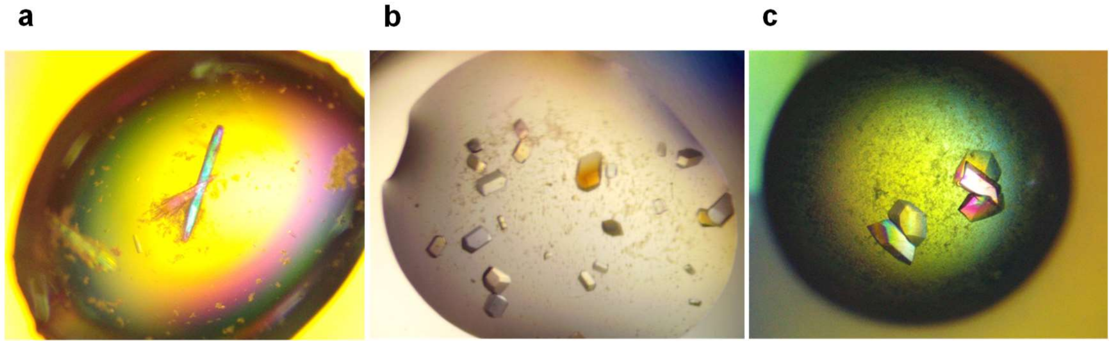

Supplementary Fig. 157 Crystals of GuApiGT (**a**), Sb3GT1 (**b**), and Sb3GT1-375S/Q377H (**c**).

4. Supplementary References

1) Zhang, C. *et al*. Extraction optimization, structural characterization and potential alleviation of hyperuricemia by flavone glycosides from celery seeds. *Food Funct.* **13**, 9832 (2022).

2) Shao, H. *et al*. Crystal structures of a multifunctional triterpene/flavonoid glycosyltransferase from *Medicago truncatula*. *Plant Cell* **17**, 3141-3154 (2005).

3) Offen, W. *et al*. Structure of a flavonoid glucosyltransferase reveals the basis for plant natural product modification. *EMBO J.* **25**, 1396-1405 (2006).

4) Li, L. *et al*. Crystal structure of *Medicago truncatula* UGT85H2 insights into the structural basis of a multifunctional (iso)flavonoid glycosyltransferase. *J. Mol. Biol.* **370**, 951-963 (2007).

5) Brazier-Hicks, M. *et al*. Characterization and engineering of the bifunctional *N*- and *O*-glucosyltransferase involved in xenobiotic metabolism in plants. *Proc. Natl. Acad. Sci. U. S. A.* **104**, 20238-20243 (2007).

6) Modolo, L. V. *et al*. Crystal structures of glycosyltransferase UGT78G1 reveal the molecular basis for glycosylation and deglycosylation of (iso)flavonoids. *J. Mol. Biol.* **392**, 1292-1302 (2009).

7) Hiromoto, T. *et al*. Structural basis for acceptor-substrate recognition of UDP-glucose: anthocyanidin 3-*O*-glucosyltransferase from *Clitoria ternatea*. *Protein Sci.* **24**, 395-407 (2015).

8) Wetterhorn, K. M. *et al*. Crystal structure of Os79 (Os04g0206600) from *Oryza sativa*: A UDP-glucosyltransferase involved in the detoxification of deoxynivalenol. *Biochemistry* **55**, 6175-6186 (2016).

9) Wetterhorn, K. M. *et al*. Determinants and expansion of specificity in a trichothecene UDP-glucosyltransferase from *Oryza sativa*. *Biochemistry* **56**, 6585-6596 (2017).

10) Thompson, A. M. G., Iancu, C. V., Neet, K. E., Dean, J. V., Choe, J. Y. Differences in salicylic acid glucose conjugations by UGT74F1 and UGT74F2 from

Arabidopsis thaliana. *Sci. Rep.* **7**, 46629 (2017).

11) Hsu, T. M. *et al*. Employing a biochemical protecting group for a sustainable indigo dyeing strategy. *Nat. Chem. Biol.* **14**, 256-261 (2018).

12) Zong, G. N. *et al*. Crystal structures of rhamnosyltransferase UGT89C1 from *Arabidopsis thaliana* reveal the molecular basis of sugar donor specificity for UDP-beta-L-rhamnose and rhamnosylation mechanism. *Plant J.* **99**, 257-269 (2019).

13) Lee, S. G., Salomon, E., Yu, O., Jez, J. M. Molecular basis for branched steviol glucoside biosynthesis. *Proc. Natl. Acad. Sci. U. S. A.* **116**, 13131-13136 (2019).

14) Yang, T. *et al*. Hydrophobic recognition allows the glycosyltransferase UGT76G1 to catalyze its substrate in two orientations. *Nat. Commun.* **10**, 3214 (2019).

15) Liu, Z. F., Li, J. X., Sun, Y. W., Zhang, P., Wang, Y. Structural insights into the catalytic mechanism of a plant diterpene glycosyltransferase SrUGT76G1. *Plant. Commun.* **1**, 100004 (2020).

16) He, J. B. *et al*. Molecular and structural characterization of a promiscuous *C*-glycosyltransferase from *Trollius chinensis*. *Angew. Chem. Int. Ed.* **58**, 11513-11520 (2019).

17) Li, J. *et al*. Near-perfect control of the regioselective glucosylation enabled by rational design of glycosyltransferases. *Green Synth. Catal.* **2**, 45-53 (2021).

18) Zhang, M. *et al*. Functional characterization and structural basis of an efficient di-*C*-glycosyltransferase from *Glycyrrhiza glabra*. *J. Am. Chem. Soc.* **142**, 3506-3512 (2020).

19) Liu, M. Z. *et al*. Crystal structures of the *C*-glycosyltransferase UGT708C1 from buckwheat provide insights into the mechanism of *C*-glycosylation. *Plant Cell* **32**, 2917-2931 (2020).

20) Wen, Z. X. *et al*. Directed evolution of a plant glycosyltransferase for chemo- and regioselective glycosylation of pharmaceutically significant flavonoids. *ACS Catal.* **11**, 14781-14790 (2021).

21) Maharjan, R. *et al*. Crown-ether-mediated crystal structures of the glycosyltransferase PaGT3 from *Phytolacca americana*. *Acta Crystallogr. D Struct. Biol.* **76**, 521-530 (2020).

22) Maharjan, R. *et al*. An ambidextrous polyphenol glycosyltransferase PaGT2 from *Phytolacca americana*. *Biochemistry* **59**, 2551-2561 (2020).

23) Li, J. *et al*. Efficient *O*-glycosylation of triterpenes enabled by protein engineering of plant glycosyltransferase UGT74AC1. *ACS Catal.* **10**, 3629-3639 (2020).

24) Wang, Z. L. *et al*. Dissection of the general two-step di-*C*-glycosylation pathway for the biosynthesis of (iso)schaftosides in higher plants. *Proc. Natl. Acad. Sci. U.S.A.* **117**, 30816-30823 (2020).

25) Zhang, J. Z. *et al*. Catalytic flexibility of rice glycosyltransferase OsUGT91C1 for the production of palatable steviol glycosides. *Nat. Commun.* **12**, 7030 (2021).

26) Huang, W., He, Y., Jiang, R. W., Deng, Z. X., Long, F. Functional and structural dissection of a plant steroid 3-*O*-glycosyltransferase facilitated the engineering enhancement of sugar donor promiscuity. *ACS Catal.* **12**, 2927-2937 (2022).

27) Yang, W. Z. *et al*. Low energy induced homolytic fragmentation of flavonol 3-*O*-glycosides by negative electrospray ionization tandem mass spectrometry. *Rapid Commun Mass Spectrom.* **28**, 385-395 (2014).
