# Supplementary material for: Insights into the missing apiosylation step in flavonoid apiosides biosynthesis of Leguminosae plants
Source: Nat Commun. 2023 Oct 20;14:6658. doi: 10.1038/s41467-023-42393-1 (PMC10589286; doi:10.1038/s41467-023-42393-1)
Supplement: Supplementary file 3 — Description of Additional Supplementary Files [file 41467_2023_42393_MOESM3_ESM.pdf]

Description of Additional Supplementary Files

File Name: Supplementary Data 1

Description: Primers used in this study.

File Name: Supplementary Data 2

Description: Gaussian optimized geometry for reactant complex (RC).

File Name: Supplementary Data 3

Description: Gaussian optimized geometry for transition state (TS).

File Name: Supplementary Data 4

Description: Gaussian optimized geometry for product complex (PC).
