# Supplementary material for: Insights into the missing apiosylation step in flavonoid apiosides biosynthesis of Leguminosae plants
Source: Nat Commun. 2023 Oct 20;14:6658. doi: 10.1038/s41467-023-42393-1 (PMC10589286; doi:10.1038/s41467-023-42393-1)
Supplement: Supplementary file 8 — Reporting Summary [file 41467_2023_42393_MOESM8_ESM.pdf]

nature portfolio

Corresponding author(s): Min Ye

Last updated by author(s): Sep 12, 2023

Reporting Summary

Nature Portfolio wishes to improve the reproducibility of the work that we publish. This form provides structure for consistency and transparency in reporting. For further information on Nature Portfolio policies, see our Editorial Policies and the Editorial Policy Checklist.

Statistics

For all statistical analyses, confirm that the following items are present in the figure legend, table legend, main text, or Methods section.

n/a	Confirmed	
[ ]	[x]	The exact sample size (*n*) for each experimental group/condition, given as a discrete number and unit of measurement
[ ]	[x]	A statement on whether measurements were taken from distinct samples or whether the same sample was measured repeatedly
[x]	[ ]	The statistical test(s) used AND whether they are one- or two-sided<br>*Only common tests should be described solely by name; describe more complex techniques in the Methods section.*
[x]	[ ]	A description of all covariates tested
[x]	[ ]	A description of any assumptions or corrections, such as tests of normality and adjustment for multiple comparisons
[ ]	[x]	A full description of the statistical parameters including central tendency (e.g. means) or other basic estimates (e.g. regression coefficient) AND variation (e.g. standard deviation) or associated estimates of uncertainty (e.g. confidence intervals)
[ ]	[x]	For null hypothesis testing, the test statistic (e.g. *F*, *t*, *r*) with confidence intervals, effect sizes, degrees of freedom and *P* value noted<br>*Give P values as exact values whenever suitable.*
[x]	[ ]	For Bayesian analysis, information on the choice of priors and Markov chain Monte Carlo settings
[x]	[ ]	For hierarchical and complex designs, identification of the appropriate level for tests and full reporting of outcomes
[x]	[ ]	Estimates of effect sizes (e.g. Cohen's *d*, Pearson's *r*), indicating how they were calculated

Our web collection on statistics for biologists contains articles on many of the points above.

Software and code

Policy information about availability of computer code

Data collection	LC data: OpenLab ChemStation C. 01. 05 (Agilent). RStudio 1.4.1103, MS data: Xcalibur 4.1 software (ThermoFisher). Computational modeling data: Autodock-4.2 (https://autodock.scripps.edu/download-autodock4/) for molecular docking; Desmond-v6.8 for molecular dynamics and well-tempered metadynamics simulations; Prime-v6.6 for binding free energy calculations; Jaguar, Desmond, and Prime are implemented in the commercial Schrödinger Software package version 2021-4 (https://www.schrodinger.com/); Gaussian 16 C.01 for QM/MM calculations.
Data analysis	LC data: OpenLab ChemStation C. 01. 05 (Agilent). MS data: Xcalibur 4.1 software (ThermoFisher). Kinetic parameters: GraphPad.Prism.v5.0. Crystallographic data: XDS version 1.13 for processing X-ray diffraction data; Phaser version 2.8 for model search; Phenix1.20.1-4487 and COOT version 0.8.9.2 for structural refinement; Phenix 1.20.1-4487 and PDB validation server (https://validate-rcsb-1.wwpdb.org/) for structural validation. Computational modeling: Pymol-open-source (https://github.com/schrodinger/pymol-open-source) and Maestro v13.0 (https://www.schrodinger.com/) for visualizations of structures; VMD (https://www.ks.uiuc.edu/Research/vmd/) tcl scripts for MD analysis; VMD molUP plugin (https://github.com/BioSIM-Research-Group/molUP) and python scripts (https://github.com/jhliTCB/jgot) for QM/MM analysis; Microsoft Powerpoint for combining the sub-figures; Matplotlib library implemented in Anaconda python 3 package for metadynamics analysis; and other in-house used python bash scripts.

For manuscripts utilizing custom algorithms or software that are central to the research but not yet described in published literature, software must be made available to editors and reviewers. We strongly encourage code deposition in a community repository (e.g. GitHub). See the Nature Portfolio guidelines for submitting code & software for further information.

Data

Policy information about availability of data

All manuscripts must include a data availability statement. This statement should provide the following information, where applicable:

- Accession codes, unique identifiers, or web links for publicly available datasets
- A description of any restrictions on data availability
- For clinical datasets or third party data, please ensure that the statement adheres to our policy

Data supporting the findings of this study are available in the article, supplementary materials, or public database. The sequence of genes data generated in this study have been deposited in the NCBI database under the following accession numbers: GuApiGT (OQ201607, https://www.ncbi.nlm.nih.gov/nuccore/OQ201607), SsApiGT/PtApiGT/GiApiGT/GgApiGT (OQ230794-OQ230797, https://www.ncbi.nlm.nih.gov/nuccore/OQ230794-OQ230797), GuGT53 (OQ266890, https://www.ncbi.nlm.nih.gov/nuccore/OQ266890) and other apiosyltransferase candidate genes from Leguminosae plants (OR372660-OR372775, https://www.ncbi.nlm.nih.gov/nuccore/OR372660-OR372775). The raw reads from the RNA-sequencing profiling analysis of G. uralensis have been deposited in the NCBI Sequence Read Archive (SRA) database under the BioProject accessions PRJNA945816 (https://www.ncbi.nlm.nih.gov/bioproject/PRJNA945816). The crystal structures in this study have been deposited in the RCSB PDB database under the following accession numbers: GuApiGT (8HZZ, https://doi.org/10.2210/pdb8HZZ/pdb), Sb3GT1 in complex with UDP (8IOE, https://doi.org/10.2210/pdb8I0E/pdb), and Sb3GT1-375S/Q377H in complex with UDP-Glc (8IOD, https://doi.org/10.2210/pdb8I0D/pdb). The primers and Gaussian optimized geometries (RC, TS and PC) are given in Supplementary Data 1-4. Source data are provided with this paper.

Research involving human participants, their data, or biological material

Policy information about studies with human participants or human data. See also policy information about sex, gender (identity/presentation), and sexual orientation and race, ethnicity and racism.

Reporting on sex and gender	not applicable
Reporting on race, ethnicity, or other socially relevant groupings	not applicable
Population characteristics	not applicable
Recruitment	not applicable
Ethics oversight	not applicable

Note that full information on the approval of the study protocol must also be provided in the manuscript.

Field-specific reporting

Please select the one below that is the best fit for your research. If you are not sure, read the appropriate sections before making your selection.

☒ Life sciences ☐ Behavioural & social sciences ☐ Ecological, evolutionary & environmental sciences

For a reference copy of the document with all sections, see nature.com/documents/nr-reporting-summary-flat.pdf

Life sciences study design

All studies must disclose on these points even when the disclosure is negative.

Sample size	Three independent biological samples were tested to obtain the mean values. This sample size was chosen according to literatures (Nat. Commun. 2023, 14, 4696. and Nature 2022, 607, 617-622.). In addition, two separate plants of Glycyrrhiza. uralensis were collected in Inner Mongolia Autonomous Region of China. The transcriptome data of three different parts (root, cortex, and leaf) of G. uralensis were acquired using Illumina sequencing platform, respectively. A total of six sets of transcriptome data were used for co-expression analysis to discover the candidate genes. The candidate genes were then confirmed by functional characterization. This strategy was similar to literature report (Nature 2022, 607, 617-622.).
Data exclusions	No data was excluded.
Replication	Most of the data presented in this study are derived from three biologically independent samples, and they show good reproducibility.
Randomization	The samples were randomly allocated into experimental groups.
Blinding	Blinding is not applicable in our study because it does not involve subjects receiving different treatments. All experiments were conducted by analyzing data derived from different biological replicates.

Reporting for specific materials, systems and methods

We require information from authors about some types of materials, experimental systems and methods used in many studies. Here, indicate whether each material, system or method listed is relevant to your study. If you are not sure if a list item applies to your research, read the appropriate section before selecting a response.

Materials & experimental systems

n/a	Involved in the study
☒	☐ Antibodies
☒	☐ Eukaryotic cell lines
☒	☐ Palaeontology and archaeology
☒	☐ Animals and other organisms
☒	☐ Clinical data
☒	☐ Dual use research of concern
☒	☐ Plants

Methods

n/a	Involved in the study
☒	☐ ChIP-seq
☒	☐ Flow cytometry
☒	☐ MRI-based neuroimaging
